# Total Synthesis and Structural Revision of Bonnevillamides B and C

**DOI:** 10.1021/acs.orglett.6c02331

**Published:** 2026-07-09

**Authors:** Emanuel Papadopoulos, Uli Kazmaier

**Affiliations:** Saarland University, Organic Chemistry I, Campus, Building C4.2, D-66123 Saarbrücken, Germany

## Abstract

The stereoselective synthesis of the unusual amino acid 5-methyl-4-hydroxyproline (MeHyPro) and its incorporation into the first synthesized bonnevillamides show that the originally assigned (2*S*,4*S*,5*R*) configuration of this amino acid is not correct and must be changed to (2*S*,4*R*,5*S*).

## Graphical abstract

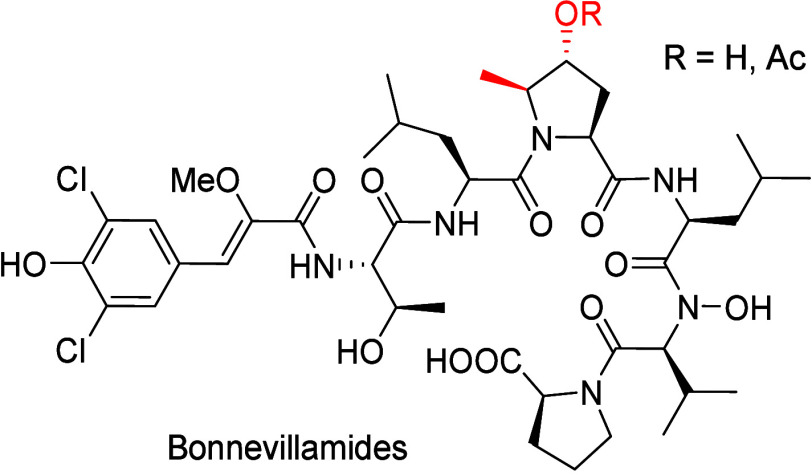

While searching for novel natural products from extremophilic microorganisms, the Winter group isolated bonnevillamides (Bv) A–C in 2017,^1^ linear hexapeptides with some rather unusual building blocks, from an actinobacterium of the Great Salt Lake in Utah, USA (Figure 1a). A striking feature of the Bv’s is the *N*-terminal-functionalized α-hydroxycinnamic acid, which was referred to as bonnevillic acid (Bva). In addition to an *N*-hydroxyvaline (HyVal), all bonnevillamides share a 5-methyl-4-hydroxyproline (MeHyPro), which exists as either the free alcohol or the corresponding acetate (MeAcPro). In BvA, another proline, located at the *C*-terminus, is also replaced by a 4-methylazetidine-2-carboxylic acid (Maz). The absolute configurations of all proteinogenic amino acid residues were determined by an advanced Marfey’s method,^2^ and for the unusual amino acids, only the relative orientation of the substituents was determined, not the absolute configuration.

**Figure 1:**
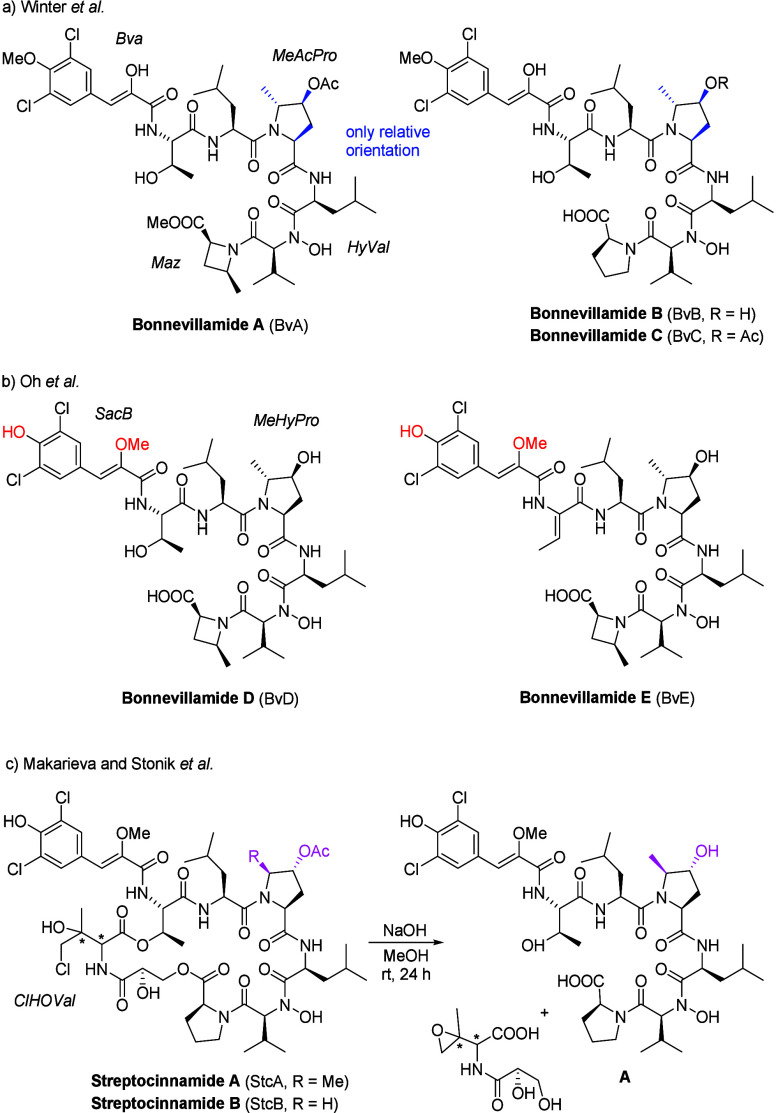
Bonnevillamides (Bv) and streptocinnamides (Stc).

Interestingly, in 2021 the Oh group succeeded in isolating analogous BvD and BvE (Figure 1b) from the actinobacterium *Streptomyces* sp. UTZ13, which was discovered close to Seoul in the exoskeleton of the carrion beetle *Nicrophorus concolor*.^3^ In principle, BvD is the saponification product of BvA, in which both the *C*-terminal methyl ester and the acetyl residue of MeAcPro have been cleaved. Furthermore, BvE features a dehydrated *N*-terminal threonine residue. In the course of the structural elucidation of BvD and BvE, Oh et al. were able to determine that the structural formulas of BvA-C, as originally postulated by Winter et al., required revision.^3^ In fact, the methyl group is located at the α-*O* atom of the cinnamic acid motif and not at the phenol-*O*. Accordingly, the *N*-terminal carboxylic acid is not Bva but saccharochlorine B (SacB), a modified cinnamic acid which was previously also isolated as a natural product.^4^ ROESY correlations were used to assign the relative configuration of the two unusual amino acids as (2S*,4S*)-Maz and (2S*,4S*,5R*)-MeAcPro, respectively.

Oh et al. were also able to elucidate the biosynthesis of the nonribosomally synthesized peptides.^3^ Two NRPS genes *bnvD* and *bnvO* encode a total of eight modules, with only the A domains of modules 1 to 6 matching the corresponding amino acids of the Bv’s. Moreover, the authors suspected that remaining modules 7 and 8 would be omitted due to this discrepancy. For example, in the A domain of module 7, some essential amino acids are missing in the conserved regions, whereas in module 8 the entire A domain is missing. This suggests that Bv’s may be “incomplete” natural products in which biosynthesis is interrupted prematurely.

This assumption is also supported by the discovery of the streptocinnamides (Stc) (Figure 1c), cyclic depsipeptides isolated by Makarieva and Stonik et al. from the marine actinomycete strain *Streptomyces* sp. KMM 9044.^5^ In contrast to the bonnevillamides, these natural products possess almost identical core structures but incorporate two additional building blocks: d-glyceric acid and a γ-chlorinated β-hydroxyvaline (ClHOVal) residue. In StcB, HyPro is not methylated at C-5. As part of the structure elucidation, StcA was saponified, whereby the two additional components were cleaved to form a linear hexapeptide **A**, which was supposed to be identical to BvB. Unfortunately, no NMR data have been published for **A**. Compared to the postulated revised structure of BvB,^3^ **A** differs only in the configuration of the two stereogenic centers of MeHyPro. The (*S*)-configurations of the proteinogenic amino acids were determined by Marfey’s method,^6^ whereby total hydrolysis of StcB clearly assigned the (2*S*,4*R*) configuration to HyPro.

On the basis of the *trans* orientation of the neighboring 5-methyl group, the streptocinnamides therefore contain (2*S*,4*R*,5*S*)-MeHyPro. This raises the question of whether different stereoisomers of MeHyPro are synthesized by the streptomycetes or whether one of the configurational elucidations may be incorrect.

For years, our research group has been working on the synthesis of complex peptide natural products that deserve special attention due to their biological activity and/or unusual structure.^7^ Therefore, we also turned our attention to BvB and BvC with the aim of confirming the configuration of MeHyPro and MeAcPro, respectively, by synthesis. To this end, it was necessary to develop an amino acid synthesis that, in principle, enables access to all stereoisomers (Scheme 1). Alongside a Sharpless dihydroxylation, a key step in our synthetic strategy was a chelate-enolate-Claisen rearrangement developed in our research group.^8^ Since the *trans* relation of the methyl and hydroxy groups of MeHyPro was undisputed on the basis of the previous structure elucidations, the proline ring should be obtainable by an intramolecular S_N_2 reaction from corresponding *cis-*diol **B**, which in turn should be accessible via Sharpless dihydroxylation from γ,δ-unsaturated amino acid **C**.^9^ Depending on the choice of the chiral ligand, both stereogenic centers at positions 4 and 5 can be adjusted as desired. **C**, in turn, is obtained by enolate-Claisen rearrangement from chiral allyl ester **D**, whereby the configuration of the allyl alcohol ultimately determines the configuration at the α-C.

**Scheme 1:**
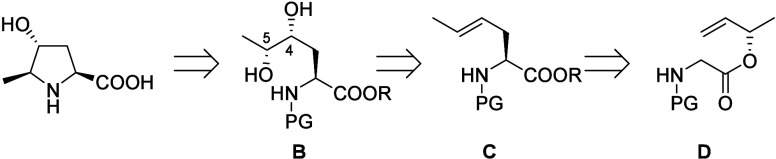
Retrosynthesis for MeHyPro

Our first attempt to obtain a suitably protected (2*S*,4*R*,5*S*)-MeHyPro derivative was made from Cbz-protected glycine propargyl ester **1**,^10^ available from commercially available (*S*)-3-butyn-2-ol (Scheme 2). Lindlar hydrogenation of **1** delivered desired allyl ester **2** in very good yield. Deprotonation with a slight excess of LDA in the presence of ZnCl_2_ leads to the formation of a chelate-bridged zinc ester enolate, which undergoes a Claisen rearrangement when heated to room temperature. Due to the fixed (*Z*)-enolate geometry and the chairlike transition state, the rearrangement takes place with perfect chirality transfer, providing enantiomerically pure **3**. Reaction with Boc_2_O and DMAP in *tert*-butanol^11^ yielded *tert*-butyl ester **4**, which was subjected to asymmetric Sharpless dihydroxylation.^12^ According to Sharpless’ model, the DHQD ligands should provide the desired stereoisomer. However, both (DHQD)_2_PHAL and (DHQD)_2_AQN provided an almost 1:1 diastereomeric mixture of diol **5** in moderate yield. In previous work, Sharpless reported that the presence of an NH bond can negatively affect stereoselectivity.^13^ Therefore, **4** was converted to biscarbamate **6**.^14^ In this case, while the Sharpless dihydroxylation was highly stereoselective, it did not yield the desired diol. Instead, cyclic carbamate **7** was obtained as the sole stereoisomer. Unfortunately, we were not able to convert **7** to desired diol **5**.

**Scheme 2:**
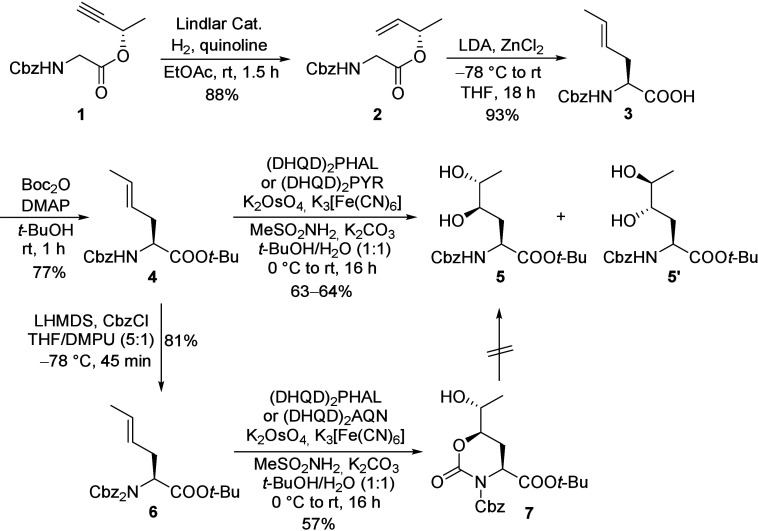
First Attempt to Get Access to MeHyPro

Since Boc-protected amino acids are less prone to the formation of oxazolidinones, allyl ester **8** ^15^ was used in a second attempt (Scheme 3). Instead of a *tert*-butyl ester, benzyl ester **9** was synthesized to ensure orthogonality to the two Boc-protecting groups in **10**. In this case, the Sharpless dihydroxylation provided an excellent *dr* value of >95:5 (determined via ^1^H NMR) and a good yield. Finally, diol **11** was converted with SOCl_2_ and pyridine to the corresponding cyclic sulfite, which was then oxidized with NaIO_4_ and catalytic amounts of RuCl_3_ to give cyclic sulfate **12**.^16^

**Scheme 3:**
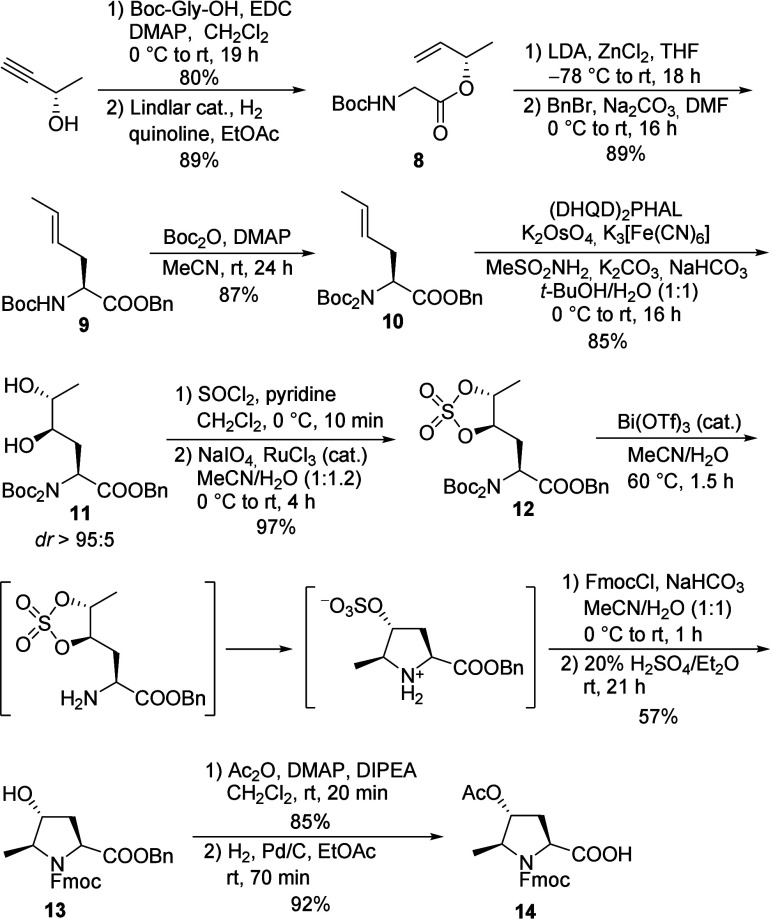
Successful Synthesis of Protected MeHyPro

Due to its limited stability, intermediate **12** was directly converted to desired proline derivative **13** by cleavage of the Boc protecting groups, in situ cyclization, and subsequent Fmoc protection. Several mild Lewis acids were tested to cleave the Boc protection groups,^17^ whereby Bi­(OTf)_3_ in MeCN/H_2_O proved to be the most effective.^18^ Boc cleavage and in situ cyclization primarily formed the monosulfuric acid ester, which could be cleaved with dilute sulfuric acid after Fmoc protection of the secondary amine. Under these conditions, a yield of 57% was obtained for **13** over the whole sequence. In order to finish desired *N*-protected amino acid **14**, the secondary hydroxyl group was acetylated and the benzyl ester was cleaved by catalytic hydrogenation. Starting from (*S*)-3-butyn-2-ol, the total yield of **14** was 18% over nine steps.

Next, we turned to the synthesis of a correspondingly protected side chain, SacB. We decided to use an allyl protection group on the phenol, which should ultimately be cleaved via Pd catalysis. Therefore, commercially available 3,5-dichloro-4-hydroxybenzaldehyde was first converted to corresponding allyl ether **15**, and the aldehyde was then subjected to a Wittig reaction to provide desired Sac derivative **17** (Scheme 4). Wittig reagent **16** was prepared in a two-step sequence starting from α-methoxyacetic acid methyl ester via radical α-bromination^19^ and substitution of the bromide by PPh_3_. Following a protocol by Seneci et al., this phosphonium salt was deprotonated with DBU in THF, which provides the best (*Z*)-selectivities with electron-rich aldehydes.^20^ Indeed, only traces of the (*E*)-isomer could be detected, whereby only the desired (*Z*)-isomer was isolated after column chromatography. Finally, the methyl ester in **17** was saponified, providing allyl-protected SacB **18** in quantitative yield.

**Scheme 4:**
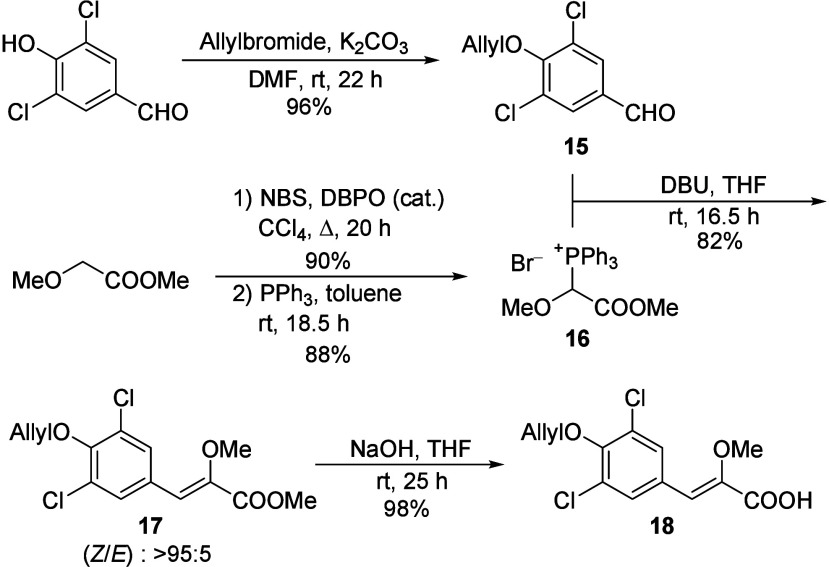
Synthesis of Protected SacB

The last remaining unusual amino acid was *N*-hydroxyvaline (HyVal), which, according to Sarnowski and Del Valle, is easily accessible from the *tert-*butyl ester of valine (Scheme 5).^21^ Since Fmoc-amino acid chlorides are usually employed for the coupling of *N*-hydroxyamino acids,^22^ **19** was coupled with Fmoc-Leu-Cl, which was freshly prepared from Fmoc-Leu-OH, oxalyl chloride, and catalytic amounts of DMF. To avoid side reactions of the free *N*–OH group of valine during further coupling steps, this group was also allylated in order to be cleaved together with the aryl allyl ether at the end of the synthesis. Pd-catalyzed allylation of **20** with allyl methyl carbonate^23^ was the method of choice. Acidic cleavage of the *tert*-butyl ester provided dipeptidic acid **21**, which was subsequently linked with H-Pro-O*t*-Bu to the *C*-terminal end of BvB and BvC. While a number of classic coupling reagents, such as HBTU, T3P, and HATU, delivered desired tripeptide **22** in low yield, EDC/Oxyma provided a yield of 69%. Therefore, the following peptide couplings to peptides **23**–**26** were mostly carried out with EDC/Oxyma, whereby consistently good yields were achieved for all steps. The respective cleavage of the Fmoc protecting groups was carried out with Et_2_NH in MeCN. The two allyl protecting groups of **26** were simultaneously cleaved and Pd-catalyzed with dimethylbarbituric acid (DMBA), and the *tert*-butyl ester was cleaved with trifluoroacetic acid, giving rise to bonnevillamide **27**. Methanolysis of the acetate residue on MeAcPro under basic conditions provided deacetylated derivative bonnevillamide **28**.

**Scheme 5:**
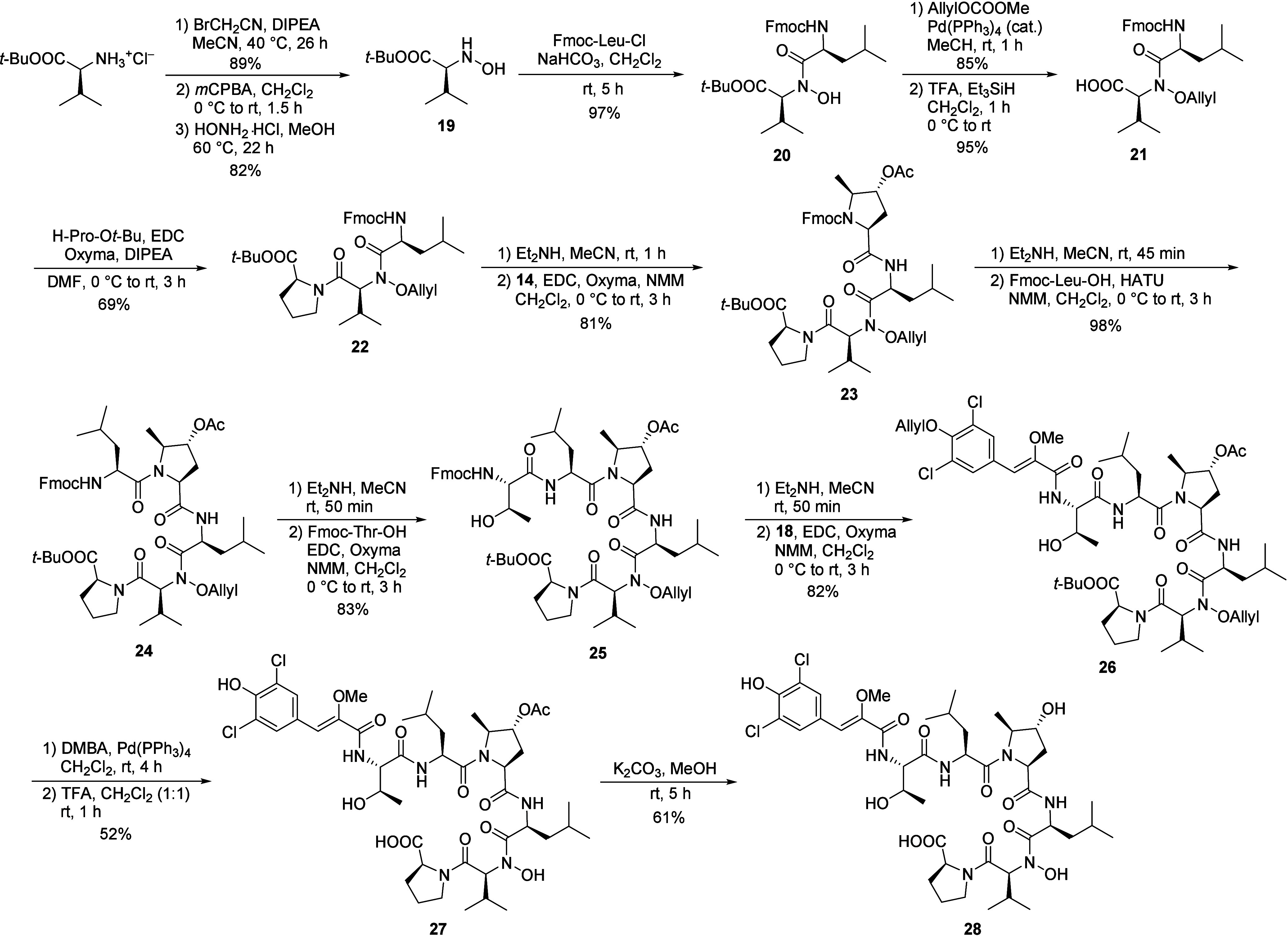
Synthesis of Bonnevillamide C (**27**) and B (**28**)

In order to check whether the two synthesized Bv’s are identical to those isolated by Winter et al., the optical rotation values of the synthesized compounds were compared with those of the isolated compounds. The [α]_D_^20^ of **27** = −40.1 (c = 0.5, CHCl_3_) was identical to that measured for BvC, [α]_D_^20^ = −40 (c = 0.1, CHCl_3_), and the value for **28** ([α]_D_^20^ = −26.0 (c = 0.1, CHCl_3_)) also closely matched that of BvB ([α]_D_^20^ = −25 (c = 0.1, CHCl_3_)).

Furthermore, the NMR spectra of the newly synthesized compounds were compared with those of the isolated compounds. The ^1^H NMR data of **27** differed by only 0.00–0.05 ppm from the corresponding signals of BvC, with most deviations below 0.02 ppm. The situation is similar when comparing the ^13^C NMR data. Here, the deviations range from 0.1 to 0.5 ppm, with a few outliers such as the methyl groups of leucine, which is located between HyVal and MeHyp. For both methyl groups, the deviation is 1.1 ppm.

This observation can be attributed to the fact that the chemical shifts of linear peptides, especially those containing a free carboxylic acid, are often pH- and concentration-dependent. Similar deviations can also be found in the original spectra of BvA–C. By acidifying the NMR sample with 0.4 vol % TFA, a better match between the reported signals could be obtained. However, the deviation of the two methyl carbons remained unchanged. This can be attributed to the steric crowding of the leucine residue between HyVal and MeHyp, which probably renders these protons sensitive to microenvironmental changes in side-chain rotameric populations.^24^

The ^1^H chemical shifts of **28** differed by only 0.01–0.04 ppm from the corresponding signals of BvB, except for the signal of some leucine CH groups. The same applies to the corresponding ^13^C data; in particular, the ^13^C carbonyl signals of the two leucine units differed strongly (Δδ = 3.1, 5.7 ppm). The NMR signals of the two leucines were probably swapped (Supporting Information). Under this assumption, the signals obtained agree very well with the published data.

In conclusion, based on the first total synthesis of BvB and BvC, the originally postulated structure must be revised. As previously reported, the natural products do not contain the proposed Bva but SacB, and the absolute configuration of (2*S*,4*R*,5*S*)-MeHyPro corresponds to that of the structurally related streptocinnamides.

## Supplementary Material



## Data Availability

The data underlying this study are available in the published article and its Supporting Information.

## Notes

Detailed experimental procedures, NMR data, and copies of ^1^H and ^13^C NMR spectra (PDF)
