# Supplementary material for: Total Synthesis and Structural Revision of Bonnevillamides B and C
Source: Org Lett. 2026 Jul 9;28(29):9244–9. doi: 10.1021/acs.orglett.6c02331 (PMC13411061; doi:10.1021/acs.orglett.6c02331)
Supplement: Supplementary file 1 [file ol6c02331_si_001.pdf]

Total Synthesis and Structural Revision of Bonnevillamides B and C

Emanuel Papadopoulos, Uli Kazmaier*

Saarland University, Institute for Organic Chemistry I, Building C4.2

P. O. Box 151150, 66041 Saarbruecken, Germany

Email: u.kazmaier@mx.uni-saarland.de

Supporting Information

List of Abbreviations 1

General Information 2

Studies on the Conversion of 7 to 5 3

Synthetic Procedures 4

NMR Spectra 29

NMR Data Comparison of Authentic and Synthetic Bonnevillamides B and C . 115

Bibliography 125

List of Abbreviations

$(DHQD)_2$ AQN	Hydroquinidine anthraquinone-1,4-diyl diether
$(DHQD)_2$ PHAL	Hydroquinidine 1,4-phthalazinediyl diether
$(DHQD)_2$ PYR	Hydroquinidine-2,5-diphenyl-4,6-pyrimidinediyl diether
Ac	Acetyl
All	Allyl
Bn	Benzyl
Boc	*tert*-Butyloxycarbonyl
Bu	Butyl
BvB	Bonnevillamide B
BvC	Bonnevillamide C
Cbz	Benzyloxycarbonyl
cHex	Cyclohexane
COSY	Correlation spectroscopy
DBPO	Benzoyl peroxide
DBU	1,8-Diazabicyclo(5.4.0)undec-7-ene
DCM	Dichloromethane
DIPA	Diisopropylamine
DIPEA	*N*,*N*-Diisopropylethylamine
DMAP	4-Dimethylaminopyridine
DMBA	1,3-Dimethylbarbituric acid
DMF	Dimethylformamide
DMPU	*N*,*N'*-Dimethylpropyleneurea
EDC	*N*-Ethyl-*N'*-(3-dimethyl-aminopropyl)carbodiimide hydrochloride
eq.	Equivalent
ESI	Electrospray ionization
Et	Ethyl
Fmoc	Fluorenylmethyloxycarbonyl
HMBC	Heteronuclear multiple-bond correlation spectroscopy
HSQC	Heteronuclear single-quantum correlation spectroscopy
HyVal	*N*-Hydroxy valine
LC/MS	Liquid chromatography–mass spectrometry
LDA	Lithium diisopropylamide
LHMDS	Lithium bis(trimethylsilyl)amide
Lit.	Literature
*m*CPBA	*m*-chloroperoxybenzoic acid
Me	Methyl
MeHyPro	5-methyl-4-hydroxyproline
NOESY	Nuclear Overhauser effect spectroscopy
NBS	*N*-Bromosuccinimide
NMM	*N*-Methylmorpholine
Oxyma	Ethyl cyanohydroxyimino-acetate
rt	Room temperature
SacCl	Saccharochlorine B
t-Bu	*tert*-Butyl
TESH	Triethylsilane
TFA	Trifluoroacetic acid
THF	Tetrahydrofuran
TMS	Tetramethylsilane
TOCSY	Total correlation spectroscopy

General Information

All air and moisture sensitive reactions were carried out in dried glassware (> 100 °C) under N_2 or Ar atmosphere. Anhydrous solvents were purchased from Acros Organics or dried before use (THF was distilled over sodium/benzophenone) and stored under N_2 atmosphere. The products were purified by column chromatography on a Grace *Reveleris PREP Chromatography* system or a Büchi *Pure C-815 Flash* system using prepacked columns *RediSep® Rf* from *Teledyne Isco*. For reverse-phase chromatography (indicated by C_{18}-SiO_2), a Grace *Reveleris PREP Chromatography* system was used with *Büchi FlashPure Select C18* columns and MeCN/H_2O solvents. Analytical TLC was performed on pre-coated silica gel plates (Machery-Nagel, Polygram Sil G/UV_{254}). Detection was accomplished with UV light (254 nm), $KMnO_4$ solution, ninhydrin solution or cerium(IV)/ ammonium molybdate solution. Melting points were determined with a MEL-TEMP II (Laboratory devices) apparatus and are uncorrected. ^1H and ^{13}C NMR spectra were recorded at 293 or 298 K on a Bruker Avance II 400 MHz spectrometer [^1H 400 MHz and ^{13}C 101 MHz], a Bruker Avance I 500 MHz spectrometer [^1H 500 MHz and ^{13}C 126 MHz] or a Bruker AV 500 Neo spectrometer [^1H 500 MHz and ^{13}C 126 MHz]. Chemical shifts (δ) are reported in parts per million (ppm) relative to TMS or internal solvent signal. Peaks were assigned using (^1H,^1H)-COSY, (^1H,^{13}C)-HSQC and (^1H,^{13}C)-HMBC spectra. Chemical shifts of C atoms marked with * were estimated from the HSQC/HMBC spectra. LC/MS measurements were performed on a Shimadzu system (system controller: SCL-10A, liquid chromatograph: LC-2030C ED Plus, autosampler: SCL-6B, mass spectrometer: LCMS-2020), using an Onyx $C_{18(2)}$ column (50×4.6 mm, 3 µm particle size) from Phenomenex as the stationary phase. Detection was performed using a diode array detector (190–300 nm) and a mass detector (Shimadzu LCMS-2020) via ESI. Mass spectra were recorded with a Bruker Daltonics maXis 4G (ESI). Optical rotations were measured with a Krüss P8000-T80 polarimeter in a thermostated (20 °C ± 1 °C) cuvette, using a sodium vapor lamp (λ = 589 nm) as radiation source. $[\alpha]_D^{20}$ values are given in 10^{-1}deg cm^2 g^{-1}.

Studies on the Conversion of 7 to 5

Table 1: Conducted studies on the conversion of cyclic carbamate **7** to acyclic mono carbamate **5**.

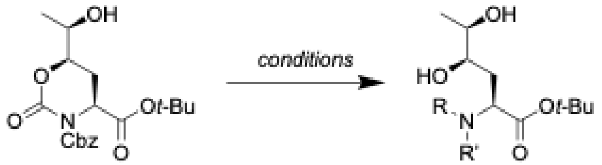

Entry	Conditions	Comment
**1**[1]	4.2 eq. LiCl, 5.0 eq. BnOH, 1.3 eq. $LiOH_{(aq.)}$, THF, 0 °C ⟶ rt, 24 h (R = R‘ = Cbz)	No conversion
**2**[2]	0.3 eq. Cs_2CO_3, MeOH, rt, 22 h (R = Cbz, R‘ = H)	Only partial Cbz- and *t*-Bu-ester cleavage
**3**[3]	1.1 eq. $LiOH_{(aq.)}$, MeOH/THF/H_2O (2:2:1.5), rt, 17 h (R = Cbz, R‘ = H)	Product in traces, partial Cbz- and *t*-Bu-ester cleavage
**4**	2.2 eq. K_2CO_3, MeOH/THF/H_2O (2:1:1), rt, 17 h (R = Cbz, R‘ = H)	Mainly *t*-Bu-ester cleavage

Synthetic Procedures

General Procedure (GP) for Fmoc Cleavage with Et_2NH followed by EDC/Oxyma coupling

Et_2NH (20 eq.) was added to a solution of the Fmoc-protected amine in MeCN (0.2–0.3 M) at rt. After complete conversion was confirmed via LC/MS (45–60 min), the mixture was concentrated under reduced pressure. Finally, the residue was co-evaporated twice with toluene and twice with DCM.

The resulting free amine was dissolved with the carboxylic acid component in DCM (0.1–0.3 M) and cooled to 0 °C. The solution was then treated with Oxyma, followed by EDC and NMM, and then slowly warmed to rt. After complete conversion was confirmed via LC/MS (3–4 h), the mixture was diluted with EtOAc and washed with 1 M $HCl_{(aq.)}$, H_2O, saturated $NaHCO_{3(aq.)}$ and saturated $NaCl_{(aq.)}$ solution. The organic phase was dried over $MgSO_4$, the solvent was removed *in vacuo* and the resulting residue was purified by column chromatography.

(*S*)-But-3-yn-2-yl-((benzyloxy)carbonyl)glycinate [1]

A solution of 687 µL (8.74 mmol, 1.0 eq., ρ = 0.891 g/mL) of (*S*)-3-butyn-2-ol and 2.01 g (9.61 mmol, 1.1 eq.) of Cbz-Gly-OH in a mixture of 18 mL anhydrous DCM and 6.0 mL anhydrous DMF was treated with 1.91 g (9.95 mmol, 1.1 eq.) EDC, followed by 112 mg (914 µmol, 0.1 eq.) DMAP. The colourless solution was stirred for 19 h before the now pale yellow solution was diluted with EtOAc. It was then washed with 1 M $KHSO_{4(aq.)}$, 1 M $LiCl_{(aq.)}$, saturated $NaHCO_{3(aq.)}$ and saturated $NaCl_{(aq.)}$ solution. The organic phase was dried over $MgSO_4$ and the solvent was removed *in vacuo.* Automated column chromatography of the crude product (SiO_2, *n*-pentane/EtOAc 10:0 ⟶ 7:3) yielded 1.94 g (7.43 mmol, 85 %) of ester **1** as a colourless oil.

$[\alpha]_D^{20}$ = +8.4 (c = 1.0, $CHCl_3$) **R$_f$** = 0.28 (*n*-Pentane/EtOAc 7:3)

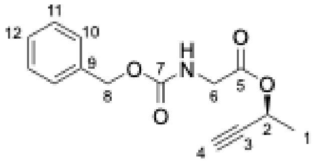

^{1}H NMR (500 MHz, $CDCl_3$): δ = 7.39–7.29 (m, 5 H, 10-H, 11-H, 12-H), 5.48 (qd, $^3J_{2,1}$ = 6.7 Hz, $^4J_{2,4}$ = 2.1 Hz, 1 H, 2-H), 5.29 (m, 1 H, NH), 5.13 (s, 2 H, 8-H), 4.03 (dd, $^2J_{6a,6b}$ = 18.3 Hz, $^3J_{6a,NH}$ = 5.8 Hz, 1 H, 6-H$_a$), 3.98 (dd, $^2J_{6b,6a}$ = 18.3 Hz, $^3J_{6b,NH}$ = 5.5 Hz, 1 H, 6-H$_b$), 2.48 (d, $^4J_{4,2}$ = 2.2 Hz, 1 H, 4-H), 1.52 (d, $^3J_{1,2}$ = 6.7 Hz, 3 H, 1-H).

^{13}C NMR (126 MHz, $CDCl_3$): δ = 169.1 (s, C-5), 156.3 (s, C-7), 136.3 (s, C-9), 128.7 (d, C-11), 128.3 (d, C-12), 128.2 (d, C-10), 81.5 (s, C-3), 73.7 (d, C-4), 67.3 (t, C-8), 61.4 (d, C-2), 42.9 (d, C-6), 21.3 (q, C-1).

HRMS (ESI) m/z: $[M+H]^+$ Calcd. for $C_{14}H_{16}NO_4^+$ 262.1074; Found 262.1074.

(S)-But-3-en-2-yl-((benzyloxy)carbonyl)glycinate [2]

First, 2.00 g (7.65 mmol, 1.0 eq.) of alkyne **1** was dissolved in 19 mL EtOAc and mixed with 454 µL (3.83 mmol, 0.5 eq., ρ = 1.090 g/mL) quinoline and 162 mg of Lindlar's catalyst. The greyish suspension was stirred under H_2 atmosphere (balloon) for 1.5 h, before being filtered through Celite and rinsed with EtOAc. The organic phase was then washed twice with 1 M $HCl_{(aq.)}$ and once with saturated $NaCl_{(aq.)}$ solution, dried over $MgSO_4$ and the solvent was removed *in vacuo*. Finally, the residue was purified by automated column chromatography (SiO_2, *n*-pentane/EtOAc 100:0 ⟶ 75:25). A total of 1.77 g (6.72 mmol, 88 %) of alkene **2** was isolated as a colourless oil.

$[\alpha]_D^{20}$ = −16.1 (c = 1.0, $CHCl_3$) **R_f** = 0.33 (*n*-Pentane/EtOAc 7:3)

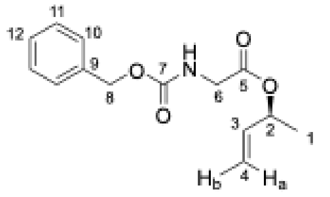

^1H NMR (500 MHz, $CDCl_3$): δ = 7.39–7.29 (m, 5 H, 10-H, 11-H, 12-H), 5.82 (ddd, $^3J_{3,4a}$ = 16.9 Hz, $^3J_{3,4b}$ = 10.5 Hz, $^3J_{3,2}$ = 6.1 Hz, 1 H, 3-H), 5.40 (quint, $^3J_{2,1} \approx {}^3J_{2,3}$ = 6.4 Hz, 1 H, 2-H), 5.30 (m, 1 H, NH), 5.25 (d, $^3J_{4a,3}$ = 17.3 Hz, 1 H, 4-H_a), 5.16 (d, $^3J_{4b,3}$ = 10.5 Hz, 1 H, 4-H_b), 5.12 (s, 2 H, 8-H), 3.97 (m, 2 H, 6-H), 1.33 (d, $^3J_{1,2}$ = 6.5 Hz, 3 H, 1-H).

^{13}C NMR (126 MHz, $CDCl_3$): δ = 169.4 (s, C-5), 156.3 (s, C-7), 137.1 (d, C-3), 136.3 (s, C-9), 128.6 (d, C-11), 128.3 (d, C-12), 128.2 (d, C-10), 116.6 (t, C-4), 72.6 (d, C-2), 67.2 (t, C-8), 43.1 (t, C-6), 20.0 (q, C-1).

HRMS (ESI) m/z: $[M+H]^+$ Calcd. for $C_{14}H_{18}NO_4^+$ 264.1230; Found 264.1231.

(S,E)-2-((Benzyloxycarbonyl)amino)hex-4-enoic acid [3][4]

<u>LDA solution:</u> Under N_2 atmosphere, 2.20 mL (15.6 mmol, 3.1 eq., ρ = 0.717 g/mL) DIPA was dissolved in 16 mL anhydrous THF and cooled to −83 °C (EtOAc/$N_{2(l)}$). Subsequently, 6.0 mL (2.5 M in *n*-hexane, 15.0 mmol, 3.0 eq.) *n*-BuLi was added dropwise, before the mixture was stirred for 15 min at rt.

<u>Claisen rearrangement:</u> A solution of 1.32 g (5.02 mmol, 1.0 eq.) of allyl ester **3** in 16 mL anhydrous THF was added under N_2 atmosphere to a suspension of 832 mg (6.10 mmol, 1.2 eq.) $ZnCl_2$ (dried under high vacuum by heating with a heat gun) in 16 mL anhydrous THF and cooled to −83 °C (EtOAc/$N_{2(l)}$). The previously prepared LDA solution was slowly added down the inner wall of the reaction flask before slowly heating to rt after complete addition.

<u>Work-up:</u> After 18 h, 1 M $KHSO_{4(aq.)}$ was added to the now yellow solution, before diluting with EtOAc. After the phases were separated, the aqueous phase was extracted twice with EtOAc and the combined organic phases were washed with saturated $NaCl_{(aq.)}$ solution. The mixture was then dried over $MgSO_4$ and the solvent was removed *in vacuo*. Automated column chromatography (SiO_2, *n*-pentane/EtOAc (+ 2 % AcOH) 10:0 ⟶ 5:5) of the residue yielded 1.23 g (4.68 mmol, 93 %) of carboxylic acid **3** as a pale yellow, highly viscous oil.

$[\alpha]_D^{20}$ = +26.3 (c = 1.0, $CHCl_3$) **R_f** = 0.41 (*n*-Pentane/EtOAc 1:1)

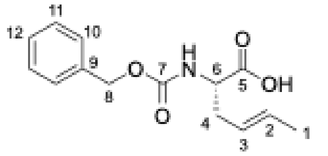

^{1}H NMR (500 MHz, $CDCl_3$): δ = 7.42–7.28 (m, 5 H, 10-H, 11-H, 12-H), 5.58 (dq, $^3J_{2,3}$ = 13.1 Hz, $^3J_{2,1}$ = 6.4 Hz, 1 H, 2-H), 5.32 (m, 1 H, 3-H), 5.25 (d, $^3J_{NH,6}$ = 8.1 Hz, 1 H, NH), 5.12 (m, 2 H, 8-H), 4.43 (dt, $^3J_{6,NH}$ = 8.1 Hz, $^3J_{6,4}$ = 5.6 Hz, 1 H, 6-H), 2.53 (m, 2 H, 4-H), 1.66 (d, $^3J_{1,2}$ = 6.4 Hz, 3 H, 1-H).

^{13}C NMR (126 MHz, $CDCl_3$): δ = 176.8 (s, C-5), 156.1 (s, C-7), 136.2 (s, C-9), 130.9 (d, C-2), 128.7 (d, C-11), 128.4 (d, C-10), 128.3 (d, C-12), 124.1 (d, C-3), 67.3 (t, C-8), 53.4 (d, C-6), 35.2 (t, C-4), 18.1 (q, C-1).

HRMS (ESI) m/z: $[M+H]^+$ Calcd. for $C_{14}H_{18}NO_4^+$ 264.1230; Found 264.1228.

***tert*-Butyl (*S*,*E*)-2-(((benzyloxy)carbonyl)amino)hex-4-enoate [4][5]**

First, 1.46 g (5.53 mmol, 1.0 eq.) of carboxylic acid **3** was dissolved in 55 mL *t*-BuOH and treated with 2.54 mL (11.1 mmol, 2.0 eq., ρ = 0.950 g/mL) Boc_2O and 201 mg (1.64 mmol, 0.3 eq.) DMAP. After stirring for 70 min, the mixture was concentrated *in vacuo* and the residue was purified by automated column chromatography (SiO_2, *n*-pentane/EtOAc 10:0 ⟶ 8:2). A total of 1.36 g (4.26 mmol, 77 %) of *tert*-butyl ester **4** was isolated as a colourless, viscous oil.

$[\alpha]_D^{20}$ = +17.0 (c = 1.0, $CHCl_3$) R_f = 0.48 (*n*-Pentane/EtOAc 7:3)

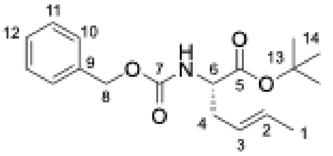

^{1}H NMR (400 MHz, $CDCl_3$): δ = 7.41–7.28 (m, 5 H, 10-H, 11-H, 12-H), 5.52 (dq, $^3J_{2,3}$ = 13.0 Hz, $^3J_{2,1}$ = 6.3 Hz, 1 H, 2-H), 5.34–5.23 (m, 2 H, NH, 3-H), 5.10 (m, 2 H, 8-H), 4.28 (dt, $^3J_{6,NH}$ = 8.3 Hz, $^3J_{6,4}$ = 5.5 Hz, 1 H, 6-H), 2.44 (m, 2 H, 4-H), 1.64 (d, $^3J_{1,2}$ = 6.2 Hz, 3 H, 1-H), 1.45 (s, 9 H, 14-H).

^{13}C NMR (101 MHz, $CDCl_3$): δ = 18.1 (q, C-1), 28.2 (q, C-14), 35.9 (t, C-4), 54.1 (d, C-6), 66.9 (t, C-8), 82.1 (s, C-13), 124.6 (d, C-3), 128.3 (d, C-10), 128.6 (d, C-11, C-12), 130.0 (d, C-2), 136.5 (s, C-9), 155.8 (s, C-7), 171.1 (s, C-5).

HRMS (ESI) m/z: $[M+H]^+$ Calcd. for $C_{18}H_{26}NO_4^+$ 320.1856; Found 320.1859.

***tert*-Butyl (2*S*)-2-[((benzyloxy)carbonyl)amino]-4,5-dihydroxyhexanoate [5/5’]**

To a solution of $(DHQD)_2PHAL$ in a mixture of 1.7 mL *t*-BuOH and 3.4 mL H_2O, 662 mg (2.01 mmol, 3.0 eq.) $K_3[Fe(CN)_6]$, 280 mg (2.03 mmol, 3.0 eq.) K_2CO_3, 0.9 mg (0.5 mol%, 2.44 µmol) K_2OsO_4 and 64.5 mg (678 µmol, 1.0 eq.) $MeSO_2NH_2$ were added and stirred vigorously. Next, the mixture was cooled to 0 °C and a solution of 214 mg (670 µmol, 1.0 eq.) of alkene **4** in 1.7 mL *t*-BuOH was added before slowly heating to rt. After 22 h, the mixture was quenched with saturated $Na_2S_2O_{3(aq.)}$ solution, before diluting with EtOAc. The phases

were then separated, the aqueous phase was extracted twice with EtOAc, and the combined EtOAc phases were washed with saturated $NaCl_{(aq.)}$ solution. The organic phase was then dried over $MgSO_4$, the solvent removed *in vacuo*, and the residue purified by automated column chromatography (SiO_2, *n*-pentane/EtOAc 10:0 ⟶ 4:6). A total of 149 mg (422 µmol, 63 %) of diols **5** and 5' was isolated as a colourless oil and a 1:1 mixture of diastereomers.

R_f = 0.21 (*n*-Pentane/EtOAc 4:6)

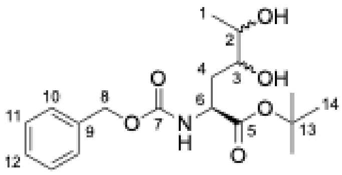

^{1}H NMR (500 MHz, DMSO-d_6, 373 K): δ = 7.44−7.27 (m, 5 H, 10-H, 11-H, 12-H), 6.97 (bs, 1 H, NH), 5.07 (m, 2 H, 8-H), 4.17 (td, $^3J_{6,NH}$ ≈ $^3J_{6,4a}$ = 7.9 Hz, $^3J_{6,4b}$ = 4.9 Hz, 0.5 H, 6-H), 4.12 (q, $^3J_{6,NH}$ ≈ $^3J_{6,4}$ = 7.1 Hz, 0.5 H, 6-H), 3.49 (m, 1 H, 2-H), 3.41 (dt, $^3J_{3,4b}$ = 8.6 Hz, $^3J_{3,2}$ ≈ $^3J_{3,4a}$ = 4.2 Hz, 0.5 H, 3-H), 3.36 (dt, $^3J_{3,4b}$ = 8.4 Hz, $^3J_{3,2}$ ≈ $^3J_{3,4a}$ 4.7 Hz, 0.5 H, 3-H), 1.91 (ddd, $^2J_{4a,4b}$ = 14.0 Hz, $^3J_{4a,6}$ = 7.3 Hz, $^3J_{4a,3}$ = 3.9 Hz, 0.5 H, 4-H$_a$), 1.73 (m, 1 H, 4-H$_a$, 4-H$_b$), 1.64 (ddd, $^2J_{4b,4a}$ = 14.0 Hz, $^3J_{4b,3}$ = 8.7 Hz, $^3J_{4b,6}$ = 6.5 Hz, 0.5 H, 4-H$_b$), 1.41 (s, 9 H, 14-H), 1.04 (d, $^3J_{1,2}$ = 6.3 Hz, 1.5 H, 1), 1.03 (d, $^3J_{1,2}$ = 6.2 Hz, 1.5 H, 1-H).

^{13}C NMR (126 MHz, DMSO-d_6, 373 K): δ = 171.4 (s, C-5), 170.9 (s, C-5), 155.2 (s, C-7), 155.1 (s, C-7), 136.7 (s, C-9), 136.5 (s, C-9), 127.8 (d, C-11), 127.7 (d, C-11), 127.2 (d, C-12), 127.1 (d, C-12), 127.1 (d, C-10), 127.0 (d, C-10), 79.9 (s, C-13), 79.8 (s, C-13), 71.3 (d, C-3), 70.6 (d, C-3), 68.9 (d, C-2), 68.4 (d, C-2), 65.3 (t, C-8), 65.0 (t, C-8), 52.3 (d, C-6), 52.0 (d, C-6), 34.2 (t, C-4), 33.5 (t, C-4), 27.3 (q, C-14), 18.3 (q, C-1), 18.1 (q, C-1).

HRMS (ESI) m/z: $[M+H]^+$ Calcd. for $C_{18}H_{28}NO_6^+$ 354.1913; Found 354.1911.

***tert*-Butyl (*S*,*E*)-2-(bis((benzyloxy)carbonyl)amino)hex-4-enoate [6][6]**

Under N_2 atmosphere, 386 mg (1.21 mmol, 1.0 eq.) of Cbz-protected amine **4** was dissolved in a mixture of 20 mL anhydrous THF and 4.0 mL anhydrous DMPU and cooled to −83 °C (EtOAc/$N_{2(l)}$). Subsequently, 1.7 mL (1.0 M in THF, 1.70 mmol, 1.4 eq.) of LHMDS was slowly added dropwise and the now yellow solution was stirred for 15 min at −83 °C before 276 µL (1.93 mmol, 1.6 eq., ρ = 1.195 g/mL) CbzCl was slowly added dropwise. After stirring for a further 45 min at −83 °C, the mixture was treated with saturated $NH_4Cl_{(aq.)}$ solution and diluted with EtOAc. The phases were then separated and the aqueous phase was extracted with EtOAc. The combined organic phases were washed with saturated $NaCl_{(aq.)}$ solution, dried over $MgSO_4$ and the solvent was removed *in vacuo*. Automated column chromatography (SiO_2, *n*-pentane/EtOAc 10:0 ⟶ 8:2; then C_{18}-SiO_2, H_2O/MeCN 90:10 ⟶ 5:95) of the residue yielded 447 mg (986 µmol, 81 %) of the bis-protected amine **6** as a colourless oil.

$[\alpha]_D^{20}$ = −24.4 (c = 1.0, $CHCl_3$) R_f = 0.41 (*n*-Pentane/EtOAc 8:2)

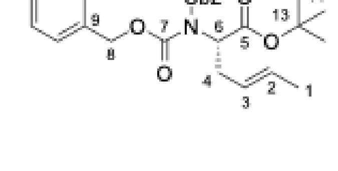

^1H NMR (500 MHz, $CDCl_3$): δ = 7.38–7.29 (m, 10 H, 10-H, 11-H, 12-H), 5.27 (d, $^3J_{8a,8b}$ = 12.3 Hz, 2 H, 8-H_a), 5.37–5.24 (m, 2 H, 2-H, 3-H), 5.21 (d, $^2J_{8b,8a}$ = 12.3 Hz, 2 H, 8-H_b), 4.91 (dd, $^3J_{6,4a/b}$ = 10.4 Hz, $^3J_{6,4b/a}$ = 5.1 Hz, 1 H, 6-H), 2.71 (m, 1 H, 4-H_a), 2.57 (m, 1 H, 4-H_b), 1.53 (m, 3 H, 1-H), 1.34 (s, 9 H, 14-H).

^{13}C NMR (126 MHz, $CDCl_3$): δ = 168.9 (s, C-5), 153.5 (s, C-7), 135.3 (s, C-9), 129.0 (d, C-2), 128.7 (d, C-12), 128.5 (d, C-11), 128.3 (d, C-10), 126.3 (d, C-3), 81.9 (s, C-13), 69.0 (t, C-8), 59.4 (d, C-6), 32.9 (t, C-4), 27.9 (q, C-14), 18.0 (q, C-1).

HRMS (ESI) m/z: $[M+H]^+$ Calcd. for $C_{26}H_{32}NO_6^+$ 454.2224; Found 454.2219.

3-Benzyl 4-(*tert*-butyl) (4*S*,6*R*)-6-((*R*)-1-hydroxyethyl)-2-oxo-1,3-oxazinane-3,4-dicarboxylate [7]

To a solution of 184 mg (405 µmol, 1.0 eq.) of alkene **6** in a mixture of 2.0 mL *t*-BuOH and 2.0 mL H_2O, 3.8 mg (4.43 µmol, 1 mol%) $(DHQD)_2AQN$, 169 mg (1.22 mmol, 3.0 eq.) K_2CO_3, 401 mg (1.22 mmol, 3.0 eq.) $K_3[Fe(CN)_6]$ and 40.4 mg (424 µmol, 1.05 eq.) $MeSO_2NH_2$ were added and stirred vigorously. Next, the mixture was cooled to 0 °C and 0.8 mg (0.5 mol%, 2.17 µmol) K_2OsO_4 was added before slowly warming to rt. After 15.5 h, 622 mg (3.27 mmol, 8.0 eq.) of $Na_2S_2O_5$ was added (gas evolution) and the mixture was diluted with water and EtOAc. The phases were then separated, the aqueous phase was extracted twice with EtOAc, and the combined EtOAc phases were washed with saturated $NaCl_{(aq.)}$ solution. The mixture was then dried over $MgSO_4$, the solvent removed *in vacuo*, and the residue purified by automated column chromatography (SiO_2, cHex/EtOAc 10:0 ⟶ 5:5). A total of 88.0 mg (232 µmol, 57 %) of cyclic carbamate **7** was isolated as a colourless oil.

$[\alpha]_D^{20}$ = +50.7 (c = 1.0, $CHCl_3$) R_f = 0.17 (*n*-Pentane/EtOAc 7:3)

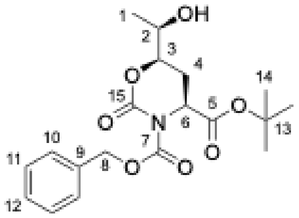

^1H NMR (400 MHz, $CDCl_3$): δ = 7.41–7.30 (m, 5 H, 10-H, 11-H, 12-H), 5.64 (d, $^3J_{OH,2}$ = 6.6 Hz, 1 H, OH), 5.11 (m, 2 H, 8-H), 4.42 (m, 1 H, 2-H), 4.36–4.28 (m, 2 H, 3-H, 6-H), 2.30 (dt, $^2J_{4a,4b}$ = 14.5 Hz, $^3J_{4a,3} \approx {}^3J_{4b,6}$ = 4.6 Hz, 1 H, 4-H_a), 2.17 (ddd, $^2J_{4b,4a}$ = 14.5 Hz, $^3J_{4b,3/6}$ = 8.5 Hz, $^3J_{4b,6/3}$ = 5.9 Hz, 1 H, 4-H_b), 1.48 (s, 9 H, 14-H), 1.42 (d, $^3J_{1,2}$ = 6.2 Hz, 3 H, 1-H).

^{13}C NMR (101 MHz, $CDCl_3$): δ = 169.7 (s, C-5), 155.9 (s, C-7), 154.0 (s, C-15), 136.1 (s, C-9), 128.7 (d, C-11), 128.5 (d, C-12), 128.2 (d, C-10), 83.8 (s, C-13), 79.5 (d, C-3), 78.2 (d, C-2), 67.3 (t, C-8), 51.4 (d, C-6), 36.0 (t, C-4), 28.0 (q, C-14), 19.1 (q, C-1).

HRMS (ESI) m/z: $[M+H]^+$ Calcd. for $C_{19}H_{26}NO_7^+$ 380.1704; Found 380.1709.

(S)-But-3-yn-2-yl-(*tert*-butoxycarbonyl)-glycinat [SI-1]

A solution of 836 µL (11.7 mmol, 1.0 eq., ρ = 0.891 g/mL) of (S)-but-3-yn-2-ol and 2.05 g (10.6 mmol, 1.1 eq.) Boc-Gly-OH in 29 mL anhydrous DCM was treated under N_2 atmosphere at 0 °C with 2.26 g (11.8 mmol, 1.1 eq.) EDC, followed by 131 mg (1.07 mmol, 0.1 eq.) DMAP. The mixture was then slowly warmed to rt and the colourless solution was stirred for 19 h before the now pale yellow solution was diluted with EtOAc. It was subsequently washed with 1 M $HCl_{(aq.)}$, H_2O, saturated $NaHCO_{3(aq.)}$ and saturated $NaCl_{(aq.)}$ solution. The organic phase was dried over $MgSO_4$ and the solvent was removed *in vacuo*. Automated column chromatography (SiO_2, *n*-pentane/EtOAc 10:0 ⟶ 7:3) of the crude product yielded 1.93 g (8.48 mmol, 80 %) of ester **SI-1** as a colourless oil.

$[\alpha]_D^{20}$ = −95.2 (c = 1.0, $CHCl_3$) R_f = 0.34 (*n*-Pentane/EtOAc 7:3)

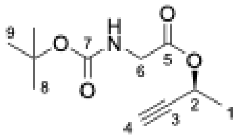

^{1}H NMR (500 MHz, $CDCl_3$): δ = 5.48 (qd, $^3J_{2,1}$ = 6.7 Hz, $^4J_{2,4}$ = 2.1 Hz, 1 H, 2-H), 5.00 (bs, 1 H, NH), 3.96 (dd, $^2J_{6a,6b}$ = 18.4 Hz, $^3J_{6a,NH}$ = 5.8 Hz, 1 H, 6-H_a), 3.90 (dd, $^2J_{6b,6a}$ = 18.4 Hz, $^3J_{6b,NH}$ = 5.5 Hz, 1 H, 6-H_b), 2.47 (d, $^4J_{4,2}$ = 2.1 Hz, 1 H, 4-H), 1.52 (d, $^3J_{1,2}$ = 6.7 Hz, 3 H, 1-H), 1.45 (s, 9 H, 9-H).

^{13}C NMR (126 MHz, $CDCl_3$): δ = 169.5 (s, C-5), 155.8 (s, C-7), 81.6 (s, C-3), 80.2 (s, C-8), 73.6 (d, C-4), 61.2 (d, C-2), 42.6 (t, C-6), 28.4 (q, C-9), 21.3 (q, C-1).

HRMS (ESI) m/z: $[M+H]^+$ Calcd. for $C_{11}H_{18}NO_4^+$ 228.1230; Found 228.1231.

(S)-But-3-en-2-yl-(*tert*-butoxycarbonyl)glycinate [8]

First, 1.90 g (8.38 mmol, 1.0 eq.) of alkyne **SI-1** was dissolved in 22 mL EtOAc and mixed with 497 µL (4.19 mmol, 0.5 eq., ρ = 1.090 g/mL) quinoline and 178 mg of Lindlar's catalyst. The pale greyish suspension was stirred under H_2 atmosphere (balloon) for 70 min before being filtered through Celite and rinsed with EtOAc. The organic phase was then washed twice with 1 M $HCl_{(aq.)}$ and once with saturated $NaCl_{(aq.)}$ solution, dried over $MgSO_4$ and the solvent was removed *in vacuo*. Finally, the residue was purified by automated column chromatography (SiO_2, *n*-pentane/EtOAc 10:0 ⟶ 8:2). A total of 1.71 g (7.46 mmol, 89 %) of alkene **8** was isolated as a colourless oil.

$[\alpha]_D^{20}$ = −27.7 (c = 1.0, $CHCl_3$) R_f = 0.40 (*n*-Pentan/EtOAc 7:3)

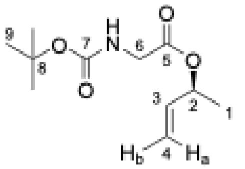

^{1}H NMR (500 MHz, $CDCl_3$): δ = 5.82 (m, 1 H, 3-H), 5.40 (quint, $^3J_{2,1} \approx {}^3J_{2,3}$ = 6.3 Hz, 1 H, 2-H), 5.25 (dt, $^3J_{4a,3}$ = 17.2 Hz, $^2J_{4a,4b} \approx {}^4J_{4a,2}$ = 11.3 Hz, 1 H, 4-H_a), 5.15 (dt, $^3J_{4b,3}$ = 10.5 Hz, $^2J_{4b,4a} \approx {}^4J_{4b,2}$ = 1.2 Hz, 1 H, 4-H_b), 5.01 (bs, 1 H, NH), 3.89 (m, 2 H, 6-H), 1.44 (s, 9 H, 9-H), 1.33 (d, $^3J_{1,2}$ = 6.5 Hz, 3 H, 1-H).

^{13}C NMR (126 MHz, $CDCl_3$): δ = 169.8 (s, C-5), 155.8 (s, C-7), 137.2 (d, C-3), 116.5 (t, C-4), 80.1 (s, C-8), 72.4 (d, C-2), 42.8 (t, C-6), 28.4 (q, C-9), 20.0 (q, C-1).

HRMS (ESI) m/z: $[M+H]^+$ Calcd. for $C_{11}H_{20}NO_4^+$ 230.1387; Found 230.1386.

Benzyl (*S*,*E*)-2-((*tert*-butoxycarbonyl)amino)hex-4-enoate [9]

LDA solution:[4] Under N_2 atmosphere, 3.40 mL (24.1 mmol, 3.2 eq., ρ = 0.717 g/mL) of DIPA was dissolved in 24 mL anhydrous THF and cooled to −83 °C (EtOAc/$N_{2(l)}$). Subsequently, 6.0 mL (2.5 M in *n*-hexane, 22.5 mmol, 3.0 eq.) of *n*-BuLi was added dropwise, before the colourless solution was warmed to rt and stirred for 20 min.

Claisen rearrangement:[4] A solution of 1.75 g (7.62 mmol, 1.0 eq.) of allyl ester **8** in 24 mL anhydrous THF was added under N_2 atmosphere to a suspension of 1.27 g (9.32 mmol, 1.2 eq.) of $ZnCl_2$ (dried under high vacuum by heating with a heat gun) in 24 mL anhydrous THF and cooled to −83 °C (EtOAc/$N_{2(l)}$). The previously prepared LDA solution was slowly run down the inner wall of the reaction flask, before the resulting mixture was slowly warmed to rt.

Work-up:[4] After 6 h, 1 M $KHSO_{4(aq.)}$ solution was added to the yellow, slightly turbid reaction mixture and diluted with EtOAc. After the phases were separated, the aqueous phase was extracted twice with EtOAc and the combined organic phases were washed with saturated $NaCl_{(aq.)}$ solution. The organic phase was then dried over $MgSO_4$ and the solvent removed *in vacuo*.

Esterification:[7] Under Ar atmosphere, the previously obtained residue was dissolved in 24 mL anhydrous DMF and cooled to 0 °C. Subsequently, 2.42 g (22.9 mmol, 3.0 eq.) Na_2CO_3 were added and 1.81 mL (15.2 mmol, 2.0 eq., ρ = 1.438 g/mL) of BnBr were added dropwise. The pale yellow suspension was then heated to rt and stirred for 5 h before being treated with saturated $NH_4Cl_{(aq.)}$ solution and diluted with EtOAc. After the phases had been separated, the aqueous phase was extracted twice with EtOAc and the combined organic phases were washed twice with saturated $NaCl_{(aq.)}$ solution. Next, the organic phase was dried over $MgSO_4$ and the solvent was removed *in vacuo*. Automated column chromatography (SiO_2, *n*-pentane/EtOAc 10:0 ⟶ 8:2) of the residue yielded 2.17 g (6.79 mmol, 89 %) of benzyl ester **9** as a colourless oil.

$[\alpha]_D^{20}$ = −26.4 (c = 1.0, $CHCl_3$) R_f = 0.63 (*n*-Pentane/EtOAc 1:1)

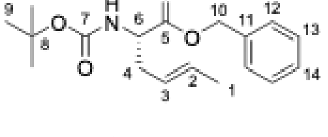

^{1}H NMR (500 MHz, $CDCl_3$): δ = 7.41−7.29 (m, 5 H, 12-H, 13-H, 14-H), 5.48 (dq, $^3J_{2,3}$ = 13.2 Hz, $^3J_{2,1}$ = 6.4 Hz, 1 H, 2-H), 5.26 (m, 1 H, 3-H), 5.22 (d, $^2J_{10a,10b}$ = 11.8 Hz, 1 H, 10-H_a), 5.11 (d, $^2J_{10b,10a}$ = 12.3 Hz, 1 H, 10-H_b), 5.01 (d, $^3J_{NH,6}$ = 8.3 Hz, 1 H, NH), 4.36 (dt, $^3J_{6,NH}$ = 8.4 Hz, $^3J_{6,4}$ = 5.6 Hz, 1 H, 6-H), 2.44 (m, 2 H, 4-H), 1.61 (d, $^3J_{1,2}$ = 6.6 Hz, 3 H, 1-H), 1.44 (s, 9 H, 9-H).

^{13}C NMR (126 MHz, $CDCl_3$): δ = 172.3 (s, C-5), 155.4 (s, C-7), 135.6 (s, C-11), 130.2 (d, C-2), 128.7 (d, C-13), 128.52 (d, C-14), 128.48 (d, C-12), 124.6 (d, C-3), 80.0 (s, C-8), 67.1 (t, C-10), 53.4 (d, C-6), 35.7 (t, C-4), 28.5 (q, C-9), 18.1 (q, C-1).

HRMS (ESI) m/z: $[M+H]^+$ Calcd. for $C_{18}H_{26}NO_4^+$ 320.1856; Found 320.1859.

Benzyl (*S*,*E*)-2-(bis(*tert*-butoxycarbonyl)amino)hex-4-enoate [10][8]

Under Ar atmosphere, 2.17 g (6.78 mmol, 1.0 eq.) of the Boc-protected amine **9** was dissolved in 23 mL anhydrous MeCN and 1.76 mL (7.68 mmol, 1.1 eq., ρ = 0.950 g/mL) of Boc_2O were added, followed by 196 mg (1.60 mmol, 0.2 eq.) DMAP. After 7 h, a further 1.60 mL (6.96 mmol, 1.0 eq.) of Boc_2O was added to the now red solution, before the solvent was removed *in vacuo* after stirring for 16.5 h. Automated column chromatography (SiO_2, *n*-pentane/EtOAc 10:0 ⟶ 9:1) of the obtained residue yielded 2.48 g (5.91 mmol, 87 %) of the bis-protected amine **10** as a colourless oil.

$[\alpha]_D^{20}$ = −32.3 (c = 1.0, $CHCl_3$) R_f = 0.27 (*n*-Pentane/EtOAc 9:1)

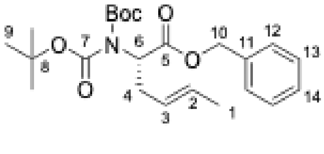

^{1}H NMR (500 MHz, $CDCl_3$): δ = 7.38–7.27 (m, 5 H, 12-H, 13-H, 14-H), 5.50 (m, 1 H, 2-H), 5.38 (m, 1 H, 3-H), 5.17 (d, $^2J_{10a,10b}$ = 12.5 Hz, 1 H, 10-H_a), 5.13 (d, $^2J_{10b,10a}$ = 12.5 Hz, 1 H, 10-H_b), 4.94 (dd, $^3J_{6,4a/b}$ = 10.1 Hz, $^3J_{6,4b/a}$ = 5.1 Hz, 1 H, 6-H), 2.79 (m, 1 H, 4-H_a), 2.62 (m, 1 H, 4-H_b), 1.63 (m, 3 H, 1-H), 1.44 (s, 18 H, 9-H).

^{13}C NMR (126 MHz, $CDCl_3$): δ = 170.6 (s, C-5), 152.2 (s, C-7), 135.8 (s, C-11), 128.8 (d, C-2), 128.6 (d, C-13), 128.3 (d, C-14), 128.2 (d, C-12), 126.5 (d, C-3), 83.0 (s, C-8), 66.9 (t, C-10), 58.2 (d, C-6), 33.2 (t, C-4), 28.1 (q, C-9), 18.2 (q, C-1).

HRMS (ESI) m/z: $[M+H]^+$ Calcd. for $C_{23}H_{34}NO_6^+$ 420.2384; Found 420.2381.

Benzyl (2*S*,4*R*,5*R*)-2-(bis(*tert*-butoxycarbonyl)amino)-4,5-dihydroxyhexanoate [11]

To a solution of 581 mg (1.51 mmol, 1.0 eq.) of alkene **10** in a mixture of 7.6 mL *t*-BuOH and 7.6 mL H_2O, 12.0 mg (15.4 µmol, 1 mol%) $(DHQD)_2PHAL$, 1.49 g (4.53 mmol, 3.0 eq.) $K_3[Fe(CN)_6]$, 146 mg (1.53 mmol, 1.0 eq.) $MeSO_2NH_2$, 384 mg (4.57 mmol, 3.0 eq.) $NaHCO_3$ and 625 mg (4.53 mmol, 3.0 eq.) K_2CO_3 were added and stirred vigorously. Next, the mixture was cooled to 0 °C and 2.6 mg (0.5 mol%, 7.06 µmol) K_2OsO_4 was added before slowly heating to rt. After 16 h, 2.29 g (12.0 mmol, 8.0 eq.) $Na_2S_2O_5$ was carefully added (gas evolution) and the mixture was diluted with H_2O and EtOAc. The phases were then separated, the aqueous phase was extracted twice with EtOAc, and the combined EtOAc phases were washed with saturated $NaCl_{(aq.)}$ solution. The organic phase was then dried over $MgSO_4$, the solvent was removed *in vacuo*, and the residue was purified by automated column chromatography (SiO_2, cHex/EtOAc 10:0 ⟶ 5:5). A total of 583 mg (1.29 mmol, 85 %) of diol **11** was isolated as a colourless oil.

$[\alpha]_D^{20}$ = −22.0 (c = 1.0, $CHCl_3$) R_f = 0.20 (*n*-Pentane/EtOAc 1:1)

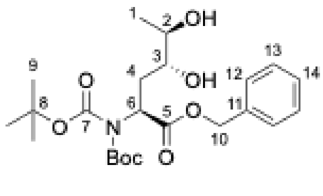

^{1}H NMR (500 MHz, $CDCl_3$): δ = 7.39–7.28 (m, 5 H, 12-H, 13-H, 14-H), 5.16 (m, 2 H, 10-H), 5.12 (m, 1 H, 6-H), 3.67–3.58 (m, 2 H, 2-H, 3-H), 2.56 (ddd, $^{2}J_{4a,4b}$ = 14.4 Hz, $^{3}J_{4a,3/6}$ = 7.3 Hz, $^{3}J_{4a,6/3}$ = 3.2 Hz, 1 H, 4-H_a), 1.78 (ddd, $^{2}J_{4b,4a}$ = 14.6 Hz, $^{3}J_{4b,3/6}$ = 8.6 Hz, $^{3}J_{4b,6/3}$ = 5.0 Hz, 1 H, 4-H_b), 1.45 (s, 18 H, 9-H), 1.22 (d, $^{3}J_{1,2}$ = 6.0 Hz, 3 H, 1-H).

^{13}C NMR (126 MHz, $CDCl_3$): δ = 171.2 (s, C-5), 152.4 (s, C-7), 135.6 (s, C-11), 128.6 (d, C-13), 128.4 (d, C-14), 128.2 (d, C-12), 83.6 (s, C-8), 74.2 (d, C-3), 70.4 (d, C-2), 67.2 (t, C-10), 55.8 (d, C-6), 35.2 (t, C-4), 28.1 (q, C-9), 19.6 (q, C-1).

HRMS (ESI) m/z: $[M+H]^+$ Calcd. for $C_{23}H_{36}NO_8^+$ 454.2436; Found 454.2435.

Benzyl (*S*)-2-(bis(*tert*-butoxycarbonyl)amino)-3-((4*R*,5*R*)-5-methyl-2,2-dioxido-1,3,2-dioxathiolan-4-yl)propanoate [12][9]

Under N_2 atmosphere, 1.16 g (2.57 mmol, 1.0 eq.) of diol **11** was dissolved in 24 mL anhydrous DCM and cooled to 0 °C. To this solution, 642 µL (7.95 mmol, 3.1 eq., ρ = 0.980 g/mL) pyridine was added, followed by the dropwise addition of 317 µL (3.85 mmol, 1.5 eq., ρ = 1.638 g/mL) thionyl chloride. After stirring for 15 min at 0 °C, the mixture was diluted with DCM and washed with water. The aqueous phase was extracted twice with DCM and the combined DCM phases were dried over $MgSO_4$ before removing the solvent *in vacuo.*

The obtained residue was dissolved in a mixture of 11 mL MeCN and 13 mL H_2O and cooled to 0 °C. Next, 828 mg (3.87 mmol, 1.5 eq.) $NaIO_4$ and 13.6 mg $RuCl_3 \cdot xH_2O$ were added and after addition, the brown reaction mixture was stirred vigorously and slowly warmed to rt. After 3.5 h, the mixture was diluted with EtOAc, filtered through Celite and rinsed with EtOAc. The filtrate was then washed with H_2O, saturated $NaHCO_{3(aq.)}$ and saturated $NaCl_{(aq.)}$ solution, dried over $MgSO_4$ and concentrated *in vacuo.* Automated column chromatography (SiO_2, *n*-pentane/EtOAc 10:0 ⟶ 7:3) of the crude product yielded 1.29 g (2.50 mmol, 97 %) of the cyclic sulphate **12** as a colourless oil.

$[\alpha]_D^{20}$ = +0.2 (c = 1.0, $CHCl_3$) R_f = 0.55 (*n*-Pentane/EtOAc 1:1)

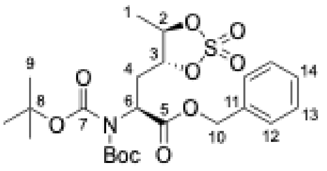

^{1}H NMR (400 MHz, DMSO-*d*$_6$): δ = 7.39–7.31 (m, 5 H, 12-H, 13-H, 14-H), 5.26 (dd, $^{3}J_{6,4b}$ = 9.2 Hz, $^{3}J_{6,4a}$ = 4.5 Hz, 1 H, 6-H), 5.15 (m, 2 H, 10-H), 5.10 (dq, $^{2}J_{2,3}$ = 8.1 Hz, $^{3}J_{2,1}$ = 5.6 Hz, 1 H, 2-H), 4.87 (ddd, $^{3}J_{3,4b}$ = 10.0 Hz, $^{3}J_{3,2}$ = 8.2 Hz, $^{3}J_{3,4a}$ = 1.7 Hz, 1 H, 3-H), 2.59 (ddd, $^{2}J_{4a,4b}$ = 15.7 Hz, $^{3}J_{4a,6}$ = 4.5 Hz, $^{3}J_{4a,3}$ = 1.7 Hz, 1 H, 4-H_a), 2.35 (dt, $^{2}J_{4b,4a}$ = 15.7 Hz, $^{3}J_{4b,3} \approx {}^{3}J_{4b,6}$ = 9.6 Hz, 1 H, 4-H_a), 1.50 (d, $^{3}J_{1,2}$ = 6.2 Hz, 3 H, 1-H), 1.40 (s, 18 H, 9-H).

^{13}C NMR (101 MHz, DMSO-*d*$_6$): δ = 174.4 (s, C-5), 156.6 (s, C-7), 140.8 (s, C-11), 133.6 (d, C-13), 133.3 (d, C-14), 133.0 (d, C-12), 93.2 (d, C-3), 90.0 (d, C-2), 88.2 (s, C-8), 71.7 (t, C-10), 61.0 (d, C-6), 36.3 (t, C-4), 32.7 (q, C-9), 21.7 (q, C-1).

HRMS (ESI): Decomposition.

Fmoc-MeHyPro-OBn [13]

Boc cleavage:[10] A solution of 529 mg (1.03 mmol, 1.0 eq.) of cyclic sulphate **12** in a mixture of 10 mL MeCN and 0.5 mL H_2O was treated with 138 mg (204 µmol, 0.2 eq.) $Bi(OTf)_3$, heated to 60 °C (oil bath) and stirred for 1.5 h.

Fmoc protection: The now colourless suspension was cooled to rt and diluted with 9.5 mL H_2O before being cooled to 0 °C. Subsequently, 260 mg (3.09 mmol, 3.0 eq.) $NaHCO_3$ and 292 mg (1.13 mmol, 1.1 eq.) FmocCl were added. The colourless suspension was then reheated to rt and concentrated *in vacuo* after 1.5 h.

Sulphuric acid ester cleavage:[9] The obtained residue was suspended in 42 mL Et_2O and 8.4 mL of 20 % (v/v) $H_2SO_{4(aq.)}$ was added. Next, the biphasic mixture was stirred vigorously for 21 h. Subsequently, the mixture was diluted with Et_2O and the phases were separated, with the aqueous phase being extracted once with Et_2O. The combined organic phases were washed with saturated $NaHCO_{3(aq.)}$ and saturated $NaCl_{(aq.)}$ solution, dried over $MgSO_4$ and concentrated *in vacuo*. The resulting residue was purified by automated column chromatography (SiO_2, *n*-pentane/EtOAc 10:0 → 4:6), after which 267 mg (583 µmol, 57 %) of proline derivative **13** was isolated as a colourless foam.

$[\alpha]_D^{20}$ = −60.4 (c = 1.0, $CHCl_3$) R_f = 0.21 (*n*-Pentane/EtOAc 1:1)

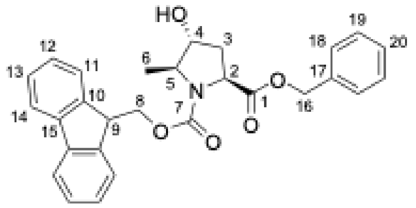

^{1}H NMR (500 MHz, DMSO-*d*$_6$, 373 K): δ = 7.85 (d, $^3J_{14,13}$ = 7.6 Hz, 2 H, 14-H), 7.62 (m, 2 H, 11-H), 7.40 (t, $^3J_{13,12} \approx {}^3J_{13,14}$ = 7.5 Hz, 2 H, 13-H), 7.35−7.26 (m, 7 H, 12-H, 18-H, 19-H, 20-H), 5.12 (s, 2 H, 16-H), 4.87 (bs, 1 H, OH), 4.41 (t, $^3J_{2,3}$ = 8.3 Hz, 1 H, 2-H), 4.35 (m, 2 H, 8-H), 4.19 (bs, 1 H, 9-H), 3.90 (m, 1 H, 4-H), 3.70 (bs, 1 H, 5-H), 2.15 (m, 1 H, 3-H$_a$), 2.06 (m, 1 H, 3-H$_b$), 1.08 (bs, 3 H, 6-H).

^{13}C NMR (126 MHz, DMSO-*d*$_6$, 373 K): δ = 171.6 (s, C-1), 153.6 (s, C-7), 143.5 (s, C-10), 140.4 (s, C-15), 135.5 (s, C-17), 127.8 (d, C-19), 127.4 (d, C-20), 127.1 (d, C-18), 127.0 (d, C-13), 126.5 (d, C-12), 124.3 (d, C-11), 119.4 (d, C-14), 74.2 (d, C-4), 66.1 (t, C-8), 65.5 (t, C-16), 62.1 (d, C-5), 58.0 (d, C-2), 46.4 (d, C-9), 35.9 (t, C-3), 17.4 (q, C-6)-

HRMS (ESI) m/z: $[M+H]^+$ Calcd. for $C_{28}H_{28}NO_5^+$ 458.1966; Found 458.1962.

Fmoc-MeHyPro(*O*Ac)-OBn [SI-2]

First, 298 mg (651 µmol, 1.0 eq.) of alcohol **13** was dissolved in 6.5 mL DCM and treated with 123 µL (1.30 mmol, 2.0 eq., *ρ* = 1.080 g/mL) Ac_2O, 277 µL (1.63 mmol, 2.5 eq., *ρ* = 0.742 g/mL) DIPEA and 8.1 mg (66.3 µmol, 0.1 eq.) DMAP. After stirring for 20 min, the mixture was diluted with Et_2O and washed with 1 M $HCl_{(aq.)}$, H_2O and saturated $NaHCO_{3(aq.)}$ solution. The organic phase was then dried over $MgSO_4$ and the solvent was removed *in vacuo*. Automated column chromatography (SiO_2, *n*-pentane/EtOAc 10:0 → 6:4) of the crude product yielded 276 mg (553 µmol, 85 %) of acetate **SI-2** as a colourless foam.

$[\alpha]_D^{20}$ = −54.6 (c = 1.0, $CHCl_3$) R_f = 0.54 (*n*-Pentane/EtOAc 1:1)

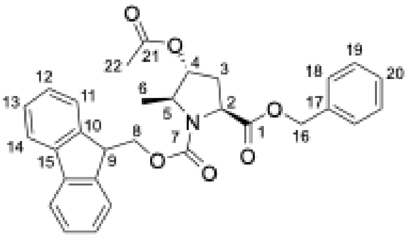

Rotamer 1 (rotamer ratio ≈ 1:1):

^{1}H NMR (500 MHz, $CDCl_3$): δ = 7.76 (m, 2 H, 14-H), 7.55 (m, 2 H, 11-H), 7.39 (m, 2 H, 13-H), 7.36–7.22 (m, 7 H, 12-H, 18-H, 19-H, 20-H), 5.13 (d, $^2J_{16a,16b}$ = 12.2 Hz, 1 H, 16-H_a), 5.07 (d, $^2J_{16b,16a}$ = 12.2 Hz, 1 H, 16-H_b), 4.92 (m, 1 H, 4-H), 4.51 (m, 1 H, 2-H), 4.35 (dd, $^2J_{8a,8b}$ = 10.6 Hz, $^3J_{8a,9}$ = 6.2 Hz, 1 H, 8-H_a), 4.22 (dd, $^2J_{8b,8a}$ = 10.6 Hz, $^3J_{8b,9}$ = 7.8 Hz, 1 H, 8-H_b), 3.96 (t, $^3J_{9,8}$ = 7.0 Hz, 1 H, 9-H), 3.91 (m, 1 H, 5-H), 2.37 (m, 1 H, 3-H_a), 2.20 (ddd, $^2J_{3b,3a}$ = 14.2 Hz, $^3J_{3b,2/4}$ = 9.9 Hz, $^3J_{3b,4/2}$ = 4.4 Hz, 1 H, 3-H_a), 2.04 (s, 3 H, 22-H), 1.19 (d, $^3J_{6,5}$ = 6.8 Hz, 3 H, 6-H).

^{13}C NMR (126 MHz, $CDCl_3$): δ = 172.1 (s, C-1), 170.5 (s, C-21), 154.2 (s, C-7), 143.6 (s, C-10), 141.3 (s, C-15), 135.4 (s, C-17), 128.7 (d, C-19), 128.6 (d, C-20), 128.2 (d, C-18), 127.9 (d, C-13), 127.8 (d, C-13), 127.1 (d, C-12), 125.0 (d, C-11), 120.1 (d, C-14), 77.5 (d, C-4), 67.6 (t, C-8), 67.2 (t, C-16), 60.4 (d, C-5), 58.7 (d, C-2), 47.2 (d, C-9), 33.6 (t, C-3), 21.3 (q, C-22), 18.0 (q, C-6).

Rotamer 2 (selected signals):

^{1}H NMR (500 MHz, $CDCl_3$): δ = 5.22 (d, $^2J_{16a,16b}$ = 12.4 Hz, 1 H, 16-H_a), 5.17 (d, $^2J_{16b,16a}$ = 12.4 Hz, 1 H, 16-H_b), 4.95 (m, 1 H, 4-H), 4.28 (t, $^3J_{9,8}$ = 6.6 Hz, 1 H, 9-H), 4.14 (m, 1 H, 5-H), 2.44 (m, 1 H, 3-H_a), 2.29 (ddd, $^2J_{3b,3a}$ = 14.3 Hz, $^3J_{3b,2/4}$ = 9.7 Hz, $^3J_{3b,4/2}$ = 4.7 Hz, 1 H, 3-H_b), 2.06 (s, 3 H, 22-H), 1.34 (d, $^3J_{6,5}$ = 6.8 Hz, 3 H, 6-H).

^{13}C NMR (126 MHz, $CDCl_3$): δ = 172.3 (s, C-1), 154.8 (s, C-7), 144.0 (s, C-10), 141.4 (s, C-15), 135.6 (s, C-17), 128.5 (d, C-18), 127.2 (d, C-12), 125.4 (d, C-11), 78.4 (d, C-4), 67.8 (t, C-8), 61.1 (d, C-5), 58.4 (d, C-2), 47.3 (d, C-9), 35.0 (t, C-3), 18.4 (q, C-6).

HRMS (ESI) m/z: $[M+H]^+$ Calcd. for $C_{30}H_{30}NO_6^+$ 500.2068; Found 500.2069.

Fmoc-MeHyPro(*O*Ac)-OH [14]

A solution of 463 mg (926 µmol, 1.0 eq.) of benzyl ester **SI-2** in 9.0 mL EtOAc was treated with 52.3 mg (10 wt%) Pd/C. The resulting black suspension was stirred vigorously under H_2 atmosphere (balloon) for 70 min before being filtered through Celite and rinsed with EtOAc. The solvent was then removed *in vacuo* and the residue was purified by automated column chromatography (SiO_2, *n*-pentane/EtOAc (+2 % AcOH) 10:0 ⟶ 5:5), with the isolated fraction being co-evaporated three times with toluene to remove residual AcOH. A total of 349 mg (852 µmol, 92 %) of carboxylic acid **14** was isolated as a colourless foam.

$[\alpha]_D^{20}$ = −45.8 (c = 1.0, $CHCl_3$) R_f = 0.22 (*n*-Pentane/EtOAc 1:1 + 1 % AcOH)

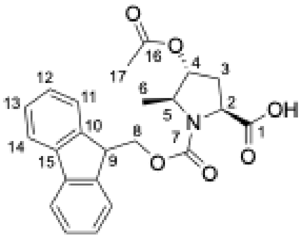

^{1}H NMR (500 MHz, DMSO-d_6, 373 K): δ = 12.26 (bs, 1 H, COOH), 7.85 (d, $^3J_{14,13}$ = 7.6 Hz, 2 H, 14-H), 7.64 (d, $^3J_{11,12}$ = 7.4 Hz, 2 H, 11-H), 7.41 (t, $^3J_{13,12} \approx {}^3J_{13,14}$ = 7.5 Hz, 2 H, 13-H), 7.33 (t, $^3J_{12,11} \approx {}^3J_{12,13}$ = 7.4 Hz, 2 H, 12-H), 4.88 (bs, 1 H, 4-H), 4.38 (bs, 2 H, 8-H), 4.30 (m, 1 H, 5-H), 4.26 (t, $^3J_{9,8}$ = 6.5 Hz, 1 H, 9-H), 3.80 (bs, 1 H, 2-H), 2.33 (m, 1 H, 3-H$_a$), 2.26 (m, 1 H, 3-H$_b$), 2.02 (s, 3 H, 17-H), 1.13 (bs, 3 H, 6-H).

^{13}C NMR (126 MHz, DMSO-d_6, 373 K): δ = 173.3 (s, C-1), 170.1 (s, C-16), 154.3 (s, C-7), 144.4 (s, C-10), 141.3 (s, C-15), 128.0 (d, C-13), 127.5 (d, C-12), 125.3 (d, C-11), 120.4 (d, C-14), 78.3 (d, C-4), 67.2 (t, C-8), 60.5 (d, C-2), 58.6 (d, C-5), 47.5 (d, C-9), 34.1 (t, C-3), 21.2 (q, C-17), 18.2 (q, C-6).

HRMS (ESI) m/z: [M+H]$^+$ Calcd. for $C_{23}H_{24}NO_6^+$ 410.1598; Found 410.1598.

4-(Allyloxy)-3,5-dichlorobenzaldehyde [15]

A solution of 2.31 g (11.7 mmol, 1.0 eq.) of 3,5-dichloro-4-hydroxybenzaldehyde in 30 mL DMF was treated with 4.86 g (35.2 mmol, 3.0 eq.) K_2CO_3 and 1.52 mL (17.6 mmol, 1.5 eq., ρ = 1.398 g/mL) allyl bromide. After 22 h, the yellow suspension was diluted with Et_2O and washed three times with H_2O. The organic phase was dried over $MgSO_4$ and the solvent was removed *in vacuo*. Finally, the crude product was purified by automated column chromatography (SiO_2, *n*-pentane/EtOAc 100:0 ⟶ 95:5), yielding 2.59 g (11.2 mmol, 96 %) of allyl ether **15** as a pale yellow solid. Melting range: 74–75 °C

R_f = 0.50 (*n*-Pentane/EtOAc 8:2)

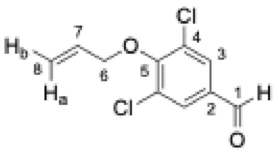

^{1}H NMR (500 MHz, $CDCl_3$): δ = 9.87 (s, 1 H, 1-H), 7.83 (s, 2 H, 3-H), 6.14 (ddt, $^3J_{7,8a}$ = 17.1 Hz, $^3J_{7,8b}$ = 10.3 Hz, $^3J_{7,6}$ = 6.0 Hz, 1 H, 7-H), 5.44 (dq, $^3J_{8b,7}$ = 17.2 Hz, $^2J_{8a,8b} \approx {}^4J_{8a,6}$ = 1.5 Hz, 1 H, 8-H$_a$), 5.32 (dq, $^3J_{8b,7}$ = 10.4 Hz, $^2J_{8b,8a} \approx {}^4J_{8b,6}$ = 1.2 Hz, 1 H, 8-H$_b$), 4.67 (dt, $^3J_{6,7}$ = 6.0 Hz, $^3J_{6,8}$ = 1.3 Hz, 2 H, 6-H).

^{13}C NMR (126 MHz, $CDCl_3$): δ = 188.9 (d, C-1), 156.3 (s, C-5), 133.2 (s, C-2), 132.5 (d, C-7), 131.0 (s, C-4), 130.1 (d, C-3), 119.7 (t, C-8), 74.9 (t, C-6).

HRMS (ESI) m/z: [M+H]$^+$ Calcd. for $C_{10}H_9Cl_2O_2^+$ 230.9974; Found 230.9971.

Methyl 2-bromo-2-methoxyacetate [SI-3][11]

To a solution of 2.18 g (20.9 mmol, 1.0 eq.) of methyl methoxyacetate in 11 mL CCl_4, 3.72 g (20.9 mmol, 1.0 eq.) NBS was added, followed by 10.2 mg (42.1 µmol, 0.2 mol%) DBPO. The mixture was slowly heated to reflux (oil bath, 95 °C; at just under 90 °C, the reaction mixture turned red and suddenly began to reflux vigorously). After 20 h, the suspension was cooled to rt, filtered and the filter cake was washed with DCM before the filtrate was concentrated *in vacuo*. Finally, the residue was distilled (12.1 mbar, 78 °C), yielding 3.43 g (18.9 mmol, 90 %) of the α-brominated ester **SI-3** as a colourless liquid.

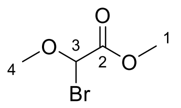

^{1}H NMR (500 MHz, $CDCl_3$): δ = 6.02 (s, 1 H, 3-H), 3.87 (s, 3 H, 1-H), 3.59 (s, 3 H, 4-H).

^{13}C NMR (101 MHz, $CDCl_3$): δ = 166.0 (s, C-2), 83.2 (d, C-3), 58.9 (q, C-4), 53.4 (q, C-1).

(1,2-Dimethoxy-2-oxoethyl)triphenylphosphonium bromide [16][11]

First, 3.35 g (18.3 mmol, 1.0 eq.) of bromide **SI-3** was dissolved in 26 mL toluene and 4.82 g (18.4 mmol, 1.0 eq.) of PPh_3 was added. After 18.5 h, the solid was filtered off and washed with Et_2O before being dried under vacuum. A total of 7.15 g (16.1 mmol, 88 %) of Wittig salt **16** was isolated as a colourless solid.

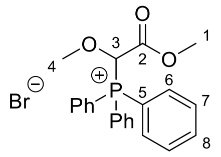

^{1}H NMR (500 MHz, $CDCl_3$): δ = 8.29 (d, $^{2}J_{3,P}$ = 13.1 Hz, 1 H, 3-H), 7.95 (m, 6 H, 7-H), 7.77 (m, 3 H, 8-H), 7.65 (m, 6 H, 6-H), 3.89 (s, 3 H, 4-H), 3.57 (s, 3 H, 1-H).

^{13}C NMR (126 MHz, $CDCl_3$): δ = 167.13 (s, C-2), 167.08 (s, C-2'), 135.2 (d, C-8), 134.9 (d, C-7), 134.8 (d, C-7'), 130.2 (d, C-6), 130.1 (d, C-6'), 117.3 (s, C-5), 116.6 (s, C-5'), 76.3 (d, C-3), 62.7 (q, C-4), 62.6 (q, C-4'), 53.4 (q, C-1).

Methyl (*Z*)-3-(4-(allyloxy)-3,5-dichlorophenyl)-2-methoxyacrylate [17][12]

Under N_2 atmosphere, 1.22 g (2.74 mmol, 1.5 eq.) of Wittig salt **16** was suspended in 6.0 mL anhydrous THF and then 438 µL (2.91 mmol, 1.6 eq., ρ = 1.010 g/mL) DBU was added. After stirring the now deep-yellow suspension for 10 min, 420 mg (1.82 mmol, 1.0 eq.) of aldehyde **15** was added and stirred for 16.5 h. The mixture was then concentrated *in vacuo*, the residue taken up in Et_2O and washed with saturated $NH_4Cl_{(aq.)}$ solution, followed by saturated $NaCl_{(aq.)}$ solution. The organic phase was then dried over $MgSO_4$ and the solvent removed *in vacuo*, before the residue was purified by automated column chromatography (SiO_2, cHex/EtOAc 10:0 ⟶ 8:2). A total of 474 mg (1.49 mmol, 82 %) of enol ether **17** was isolated as a yellow solid. Melting range: 82–83 °C.

R_f = 0.31 (*n*-Pentane/EtOAc 9:1)

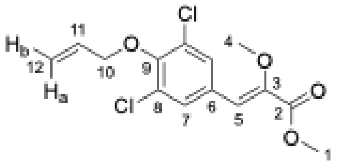

^{1}H NMR (500 MHz, $CDCl_3$): δ = 7.68 (s, 2 H, 7-H), 6.75 (s, 1 H, 5-H), 6.14 (ddt, $^3J_{11,12a}$ = 17.2 Hz, $^3J_{11,12b}$ = 10.4 Hz, $^3J_{11,10}$ = 6.0 Hz, 1 H, 11-H), 5.43 (dq, $^3J_{12a,11}$ = 17.2 Hz, $^2J_{12a,12b} \approx {}^4J_{12a,10}$ = 1.5 Hz, 1 H, 12-H_a), 5.29 (dq, $^3J_{12b,11}$ = 10.3 Hz, $^2J_{12b,12a} \approx {}^4J_{12b,10}$ = 1.2 Hz, 1 H, 12-H_b), 4.58 (dt, $^3J_{10,11}$ = 6.0 Hz, $^4J_{10,12}$ = 1.3 Hz, 2 H, 10-H), 3.85 (s, 3 H, 1-H), 3.81 (s, 3 H, 4-H).

^{13}C NMR (126 MHz, $CDCl_3$): δ = 164.3 (s, C-2), 151.3 (s, C-9), 146.5 (s, C-3), 133.0 (d, C-11), 131.0 (s, C-6), 130.3 (d, C-7), 129.7 (s, C-8), 120.5 (d, C-5), 119.1 (t, C-12), 74.6 (t, C-10), 59.5 (q, C-4), 52.5 (q, C-1).

HRMS (ESI) m/z: $[M+H]^+$ Calcd. for $C_{14}H_{15}Cl_2O_4^+$ 317.0342; Found 317.0344.

(*Z*)-3-(4-(Allyloxy)-3,5-dichlorophenyl)-2-methoxyacrylic acid [18][13]

To a solution of 761 mg (2.40 mmol, 1.0 eq.) of methyl ester **17** in 8.0 mL THF, 4.0 mL of a 2.0 M $NaOH_{(aq.)}$ solution was added. The colourless emulsion was stirred vigorously for 26.5 h before being acidified with 1 M $HCl_{(aq.)}$ (pH = 1) and diluted with EtOAc. The phases were then separated and the aqueous phase was extracted twice with EtOAc. The combined EtOAc phases were dried over $MgSO_4$ and the solvent was removed *in vacuo*. A total of 714 mg (2.35 mmol, 98 %) of carboxylic acid **18** was isolated as a colourless solid, which was used without further purification. Melting range: 173–176 °C.

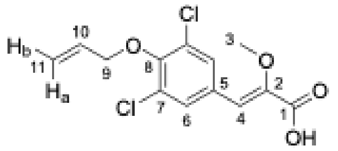

^{1}H NMR (500 MHz, DMSO-d_6): δ = 7.88 (s, 2 H, 6-H), 6.84 (s, 1 H, 4-H), 6.10 (ddt, $^3J_{10,11a}$ = 17.2 Hz, $^3J_{10,11b}$ = 10.3 Hz, $^3J_{10,9}$ = 5.8 Hz, 1 H, 10-H), 5.42 (dq, $^3J_{11a,10}$ = 17.2 Hz, $^2J_{11a,11b} \approx {}^4J_{11a,10}$ = 1.6 Hz, 1 H, 11-H_a), 5.28 (dq, $^3J_{11b,10}$ = 10.4 Hz, $^2J_{11b,11a} \approx {}^4J_{11b,10}$ = 1.2 Hz, 1 H, 11-H_b), 4.57 (dt, $^3J_{9,10}$ = 5.8 Hz, $^4J_{9,11}$ = 1.3 Hz, 2 H, 9-H), 3.76 (s, 3 H, 3-H).

^{13}C NMR (126 MHz, DMSO-d_6): δ = 164.4 (s, C-1), 150.1 (s, C-8), 147.4 (s, C-2), 133.1 (d, C-10), 131.6 (s, C-5), 129.9 (d, C-6), 128.6 (s, C-7), 118.9 (t, C-11), 118.1 (d, C-4), 74.2 (t, C-9), 58.8 (q, C-3).

HRMS (ESI) m/z: $[M+H]^+$ Calcd. for $C_{13}H_{13}Cl_2O_4^+$ 303.0185; Found 303.0187.

***tert*-Butyl *N*-cyanomethyl-L-valinate [SI-4][14]**

A solution of 1.02 g (4.89 mmol, 1.0 eq.) of H-Val-O*t*-Bu·HCl and 2.64 mL (15.2 mmol, 3.1 eq., ρ = 0.742 g/mL) DIPEA in 16 mL MeCN was treated dropwise with 409 µL (5.88 mmol, 1.2 eq., ρ = 1.722 g/mL) bromoacetonitrile at rt and then stirred for 26 h at 40 °C (oil bath). After cooling to rt, the mixture was concentrated *in vacuo*. Next, the obtained residue was dissolved in DCM and washed with saturated $NaHCO_{3(aq.)}$ solution. The aqueous phase was extracted twice with DCM and the combined organic phases were dried over $MgSO_4$ before the solvent

was removed *in vacuo*. Automated column chromatography (SiO_2, cHex/EtOAc 10:0 ⟶ 7:3) of the crude product yielded 922 mg (4.34 mmol, 89 %) of the *N*-alkylated amino acid ester **SI-4** as a colourless oil.

$[\alpha]_D^{20}$ = −16.5 (c = 1.0, $CHCl_3$) R_f = 0.29 (*n*-Pentane/EtOAc 8:2)

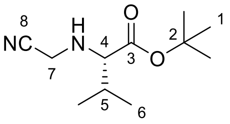

^{1}H NMR (500 MHz, $CDCl_3$): δ = 3.58 (d, $^3J_{7a,7b}$ = 17.2 Hz, 1 H, 7-H_a), 3.53 (d, $^3J_{7b,7a}$ = 17.2 Hz, 1 H, 7-H_b), 3.01 (d, $^3J_{4,5}$ = 4.9 Hz, 1 H, 4-H), 1.98 (septd, $^3J_{5,6}$ = 6.8 Hz, $^3J_{5,4}$ = 4.9 Hz, 1 H, 5-H), 1.88 (bs, 1 H, NH), 1.49 (s, 9 H, 1-H), 0.97 (d, $^3J_{6,5}$ = 6.8 Hz, 3 H, 6-H), 0.89 (d, $^3J_{6',5}$ = 6.9 Hz, 3 H, 6-H').

^{13}C NMR (126 MHz, $CDCl_3$): δ = 173.0 (s, C-3), 117.9 (s, C-8), 82.1 (s, C-2), 66.6 (d, C-4), 36.9 (t, C-7), 31.8 (d, C-5), 28.2 (q, C-1), 19.3 (q, C-6), 7.9 (q, C-6').

HRMS (ESI) m/z: $[M+H]^+$ Calcd. for $C_{11}H_{21}N_2O_2^+$ 213.1598; Found 213.1599.

***tert*-Butyl *N*-hydroxy-L-valinate [19][14]**

To a solution of 3.22 g (15.2 mmol, 1.0 eq.) of the *N*-alkylated valine derivative **SI-4** in 74 mL DCM, 8.99 g (36.5 mmol, 2.4 eq., ≈ 70 wt%) of *m*CPBA was added in two portions over 30 min at 0 °C. The resulting pink suspension was stirred for 1.5 h at room temperature before the now colourless suspension was cooled back to 0 °C and treated with approximately 50 mL each of saturated $NaHCO_{3(aq.)}$ and saturated $Na_2S_2O_{3(aq.)}$ solutions. After stirring for 5 min, the mixture was transferred to a separating funnel, prefilled with a 1:1 mixture of saturated $NaHCO_{3(aq.)}$ and saturated $Na_2S_2O_{3(aq.)}$ solution. The phases were then separated and the aqueous phase was extracted twice with DCM, before the combined organic phases were dried over $MgSO_4$ and the solvent was removed *in vacuo*.

Next, the obtained residue was dissolved in 82 mL MeOH, before 5.27 g (5.0 eq., 75.8 mmol) of hydroxylamine hydrochloride was added, and the mixture was stirred for 21 h at 60 °C (oil bath). After cooling to rt, the mixture was concentrated *in vacuo* and the residue was taken up in DCM. It was then washed with saturated $NaHCO_{3(aq.)}$ solution and the aqueous phase was extracted twice with DCM, before the combined organic phases were dried over $MgSO_4$ and the solvent was removed *in vacuo*. Automated column chromatography (SiO_2, cHex/EtOAc 10:0 ⟶ 5:5) of the residue yielded 2.34 g (12.4 mmol, 82 %) of the *N*-hydroxylated amino acid ester **19** as a colourless solid. Melting range: 68–69 °C (Lit.: 66–67 °C).[15]

$[\alpha]_D^{20}$ = −4.7 (c = 1.0, $CHCl_3$) R_f = 0.41 (*n*-Pentane/EtOAc 1:1)

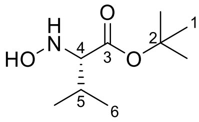

^{1}H NMR (400 MHz, $CDCl_3$): δ = 5.59 (bs, 2 H, NH, OH), 3.32 (d, $^3J_{4,5}$ = 6.5 Hz, 1 H, 4-H), 1.88 (oct, $^3J_{5,4} \approx {}^3J_{5,6}$ = 6.9 Hz, 1 H, 5-H), 1.49 (s, 9 H, 1-H), 0.96 (d, $^3J_{6,5}$ = 6.8 Hz, 6 H, 6-H, 6-H').

^{13}C NMR (126 MHz, $CDCl_3$): δ = 172.9 (s, C-3), 81.7 (s, C-2), 71.8 (d, C-4), 29.2 (d, C-5), 28.3 (q, C-1), 19.41 (q, C-6), 19.36 (q, C-6').

HRMS (ESI) m/z: $[M+H]^+$ Calcd. for $C_9H_{20}NO_3^+$ 190.1438; Found 190.1438.

Fmoc-Leu-HyVal-O*t*-Bu [20]

Under N_2 atmosphere, 2.61 g (7.39 mmol, 1.4 eq.) Fmoc-Leu-OH was suspended in 13 mL anhydrous DCM and 647 µL (7.39 mmol, 1.4 eq., ρ = 1.450 g/mL) oxalyl chloride was added. Next, seven drops of anhydrous DMF (1 drop/mmol) were added, resulting in vigorous gas evolution. The now clear, yellow solution was stirred for 1 h and then added dropwise to a suspension of 1.01 g (5.37 mmol, 1.0 eq.) H-HyVal-O*t*-Bu (**19**) and 4.50 g (53.6 mmol, 10 eq.) $NaHCO_3$ in 13 mL anhydrous DCM. After 5 h, the mixture was filtered and the filtrate was diluted with EtOAc. Next, the organic phase was washed with 1 M $HCl_{(aq.)}$, H_2O, saturated $NaHCO_{3(aq.)}$ and saturated $NaCl_{(aq.)}$ solution. The organic phase was then dried over $MgSO_4$ and the solvent was removed *in vacuo*. Automated column chromatography (SiO_2, cHex/EtOAc 10:0 ⟶ 8:2) of the crude product yielded 2.76 g (5.26 mmol, 97 %) of dipeptide **20** as a reddish foam.

$[\alpha]_D^{20}$ = −5.4 (c = 1.0, $CHCl_3$) R_f = 0.17 (*n*-Pentane/EtOAc 8:2)

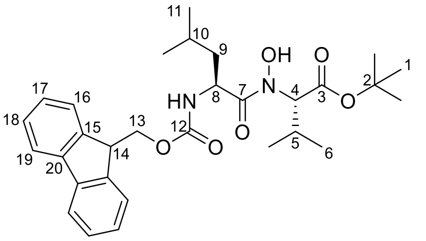

^1H NMR (500 MHz, $CDCl_3$): δ = 7.76 (m, 2 H, 19-H), 7.60 (m, 2 H, 16-H), 7.40 (m, 2 H, 18-H), 7.31 (m, 2 H, 17-H), 5.40 (d, $^3J_{NH,8}$ = 9.1 Hz, 1 H, NH), 5.05 (td, $^3J_{8,NH} \approx {}^3J_{8,9a/b}$ = 9.1 Hz, $^3J_{8,9b/a}$ = 4.5 Hz, 1 H, 8-H), 4.88 (d, $^3J_{4,5}$ = 7.7 Hz, 1 H, 4-H), 4.37 (d, $^3J_{13,14}$ = 7.1 Hz, 2 H, 13-H), 4.22 (t, $^3J_{14,13}$ = 7.2 Hz, 1 H, 14-H), 2.37 (oct, $^3J_{5,4} \approx {}^3J_{5,6}$ = 6.9 Hz, 1 H, 5-H), 1.73 (m, 1 H, 10-H), 1.61 (m, 1 H, 9-H_a), 1.57 (m, 1 H, 9-H_b), 1.50 (s, 9 H, 1-H), 1.04 (d, $^3J_{6,5}$ = 6.8 Hz, 3 H, 6-H), 1.01 (d, $^3J_{6',5}$ = 6.7 Hz, 3 H, 6-H'), 1.00 (d, $^3J_{11,10}$ = 6.1 Hz, 3 H, 11-H), 0.96 (d, $^3J_{11',10}$ = 6.6 Hz, 3 H, 11-H').

^{13}C NMR (101 MHz, $CDCl_3$): δ = 172.8 (s, C-7), 171.6 (s, C-3), 156.5 (s, C-12), 143.9 (s, C-15), 141.4 (s, C-20), 127.8 (d, C-18), 127.2 (d, C-17), 125.3 (d, C-16), 120.1 (d, C-19), 83.4 (s, C-2), 67.2 (t, C-13), 63.2 (d, C-4), 49.4 (d, C-8), 47.3 (d, C-14), 41.8 (t, C-9), 29.2 (d, C-5), 28.1 (q, C-1), 24.9 (d, C-10), 23.4 (q, C-11'), 22.0 (q, C-11), 19.62 (q, C-6), 19.57 (q, C-6').

HRMS (ESI) m/z: $[M+H]^+$ Calcd. for $C_{30}H_{41}N_2O_6^+$ 525.2959; Found 525.2953.

Fmoc-Leu-HyVal(*O*Allyl)-O*t*-Bu [SI-5][16]

Under N_2 atmosphere, 2.67 g (5.08 mmol, 1.0 eq.) of dipeptide **20** and 720 µL (6.10 mmol, 1.2 eq., ρ = 1.024 g/mL) allyl methyl carbonate were dissolved in 13 mL anhydrous MeCN and 58.7 mg (50.8 µmol, 1 mol%) $Pd(PPh_3)_4$ was added. After 1 h, the yellow solution was concentrated *in vacuo* and the residue was purified by automated column chromatography (SiO_2 *n*-pentane/EtOAc 10:0 ⟶ 8:2). A total of 2.43 g (4.31 mmol, 85%) of *O*-allylated dipeptide **SI-5** was isolated as a colourless foam.

$[\alpha]_D^{20}$ = −47.3 (c = 1.0, $CHCl_3$) R_f = 0.41 (*n*-Pentane/EtOAc 8:2)

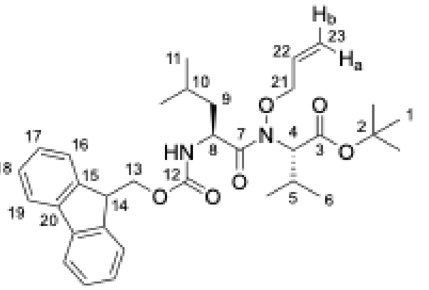

^{1}H NMR (500 MHz, $CDCl_3$): δ = 7.76 (d, $^3J_{19,18}$ = 7.7 Hz, 2 H, 19-H), 7.60 (m, 2 H, 16-H), 7.40 (tt, $^3J_{18,17} \approx {}^3J_{18,19}$ = 7.5 Hz, $^4J_{18,16} \approx {}^5J_{18,14}$ = 1.6 Hz, 2 H, 18-H), 7.31 (tt, $^3J_{17,16} \approx {}^3J_{17,18}$ = 7.4 Hz, $^4J_{17,19} \approx {}^5J_{17,14}$ = 1.4 Hz, 2 H, 17-H), 5.94 (ddt, $^3J_{22,23a}$ = 16.8 Hz, $^3J_{22,23b}$ = 10.4 Hz, $^3J_{22,21}$ = 6.2 Hz, 1 H, 22-H), 5.43 (dq, $^3J_{23a,22}$ = 17.3 Hz, $^2J_{23a,23b} \approx {}^4J_{23a,21}$ = 1.5 Hz, 1 H, 23-H_a), 5.37 (d, $^3J_{NH,8}$ = 9.6 Hz, 1 H, NH), 5.32 (d, $^3J_{23b,22}$ = 10.6 Hz, 1 H, 23-H_b), 4.83 (td, $^3J_{8,9a/b} \approx {}^3J_{8,NH}$ = 10.2 Hz, $^3J_{8,9b/a}$ = 3.4 Hz, 1 H, 8-H), 4.62 (dd, $^2J_{21a,21b}$ = 10.8 Hz, $^3J_{21a,22}$ = 6.5 Hz, 1 H, 21-H_a), 4.56 (dd, $^2J_{21b,21a}$ = 10.9 Hz, $^3J_{21b,22}$ = 6.0 Hz, 1 H, 21-H_b), 4.38 (d, $^3J_{4,5}$ = 10.8 Hz, 1 H, 4-H), 4.36 (d, $^3J_{14,13}$ = 7.7 Hz, 2 H, 14-H), 4.23 (t, $^3J_{14,13}$ = 7.3 Hz, 1 H, 14-H), 2.39 (dsept, $^3J_{5,4}$ = 10.3 Hz, $^3J_{5,6}$ = 6.6 Hz, 1 H, 5-H), 1.76 (m, 1 H, 10-H), 1.60 (ddd, $^2J_{9a,9b}$ = 13.4 Hz, $^3J_{9a,8/10}$ = 9.9 Hz, $^3J_{9a,10/8}$ = 3.4 Hz, 1 H, 9-H_a), 1.48 (m, 1 H, 9-H_b), 1.44 (s, 9 H, 1-H), 1.03 (d, $^3J_{6,5}$ = 6.5 Hz, 3 H, 6-H), 0.98 (d, $^3J_{6',5}$ = 7.3 Hz, 3 H, 6-H'), 0.97 (d, $^3J_{11,10}$ = 4.7 Hz, 3 H, 11-H), 0.95 (d, $^3J_{11',10}$ = 4.5 Hz, 3 H, 11-H').

^{13}C NMR (126 MHz, $CDCl_3$): δ = 175.1 (s, C-7), 167.2 (s, C-3), 156.4 (s, C-12), 143.9 (s, C-15), 141.4 (s, C-20), 130.7 (d, C-22), 127.8 (d, C-18), 127.2 (d, C-17), 125.3 (d, C-16), 121.0 (t, C-23), 120.1 (d, C-19), 81.9 (s, C-2), 78.1 (t, C-21), 68.0 (d, C-4), 67.1 (t, C-13), 50.3 (d, C-8), 47.3 (d, C-14), 41.5 (t, C-9), 28.4 (d, C-5), 28.1 (q, C-1), 24.8 (d, C-10), 23.7 (q, C-11'), 21.6 (q, C-11), 20.5 (q, C-6), 19.4 (q, C-6')

HRMS (ESI) m/z: $[M+H]^+$ Calcd. for $C_{33}H_{45}N_2O_6^+$ 565.3272; Found 565.3265.

Fmoc-Leu-HyVal(*O*Allyl)-OH [21]

2.43 g (4.31 mmol, 1.0 eq.) of *tert*-butyl ester **SI-5** was dissolved at 0 °C in 10.5 mL of a 95:2.5:2.5 mixture of TFA/TESH/DCM. The yellow solution was then heated to rt and concentrated *in vacuo* after stirring for 1 h. The residue was then co-evaporated thrice with $CHCl_3$ and subsequently purified by automated column chromatography (cHex/EtOAc (+2 % AcOH) 10:0 ⟶ 8:2). After lyophilisation, a total of 2.09 g (4.11 mmol, 95 %) of dipeptide **21** was obtained as a colourless lyophilisate.

$[\alpha]_D^{20}$ = −14.5 (c = 1.0, $CHCl_3$) R_f = 0.50 (*n*-Pentane/EtOAc 1:1 + 1 % AcOH)

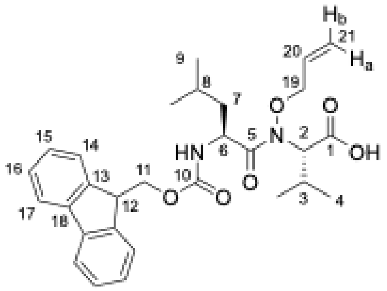

^{1}H NMR (400 MHz, $CDCl_3$): δ = 7.76 (d, $^3J_{17,16}$ = 7.6 Hz, 2 H, 17-H), 7.60 (m, 2 H, 14-H), 7.40 (t, $^3J_{16,17}$ ≈ $^3J_{16,15}$ = 7.5 Hz, 2 H, 16-H), 7.31 (t, $^3J_{15,14}$ ≈ $^3J_{15,16}$ = 7.4 Hz, 2 H, 15-H), 5.96 (ddt, $^3J_{20,21a}$ = 16.9 Hz, $^3J_{20,21b}$ = 10.3 Hz, $^3J_{22,19}$ = 6.4 Hz, 1 H, 20-H), 5.45 (d, $^3J_{21a,22}$ = 17.2 Hz, 1 H, 21-H_a), 5.40 (d, $^3J_{NH,6}$ = 9.6 Hz, 1 H, NH), 5.36 (d, $^3J_{21b,20}$ = 10.3 Hz, 1 H, 21-H_b), 4.84 (td, $^3J_{6,NH}$ ≈ $^3J_{6,7a/b}$ = 9.9 Hz, $^3J_{6,7b/a}$ = 4.0 Hz, 1 H, 6-H), 4.57 (d, $^3J_{19,20}$ = 6.5 Hz, 2 H, 19-H), 4.40 (m, 2 H, 11-H), 4.23 (t, $^3J_{12,11}$ = 7.0 Hz, 1 H, 12-H), 4.12 (d, $^3J_{2,3}$ = 10.4 Hz, 1 H, 2-H), 2.53 (dsept, $^3J_{3,2}$ = 10.2 Hz, $^3J_{3,4}$ = 6.5 Hz, 1 H, 3-H), 1.75 (m, 1 H, 8-H), 1.52 (m, 2 H, 7-H), 1.07 (d, $^3J_{4,3}$ = 6.6 Hz, 3 H, 4-H), 1.01 (d, $^3J_{4',3}$ = 6.6 Hz, 3 H, 4-H'), 0.95 (d, $^3J_{9,8}$ = 5.8 Hz, 6 H, 9-H, 9-H').

^{13}C NMR (101 MHz, $CDCl_3$): δ = 176.6 (s, C-7), 170.4 (s, C-3), 156.4 (s, C-12), 144.0 (s, C-15), 143.8 (s, C-15), 141.4 (s, C-20), 130.0 (d, C-22), 127.8 (d, C-18), 127.2 (d, C-17), 125.2 (d, C-16), 122.7 (t, C-23), 120.1 (d, C-19), 77.8 (t, C-21), 72.3 (d, C-4), 67.1 (t, C-13), 50.4 (d, C-8), 47.3 (d, C-14), 41.1 (t, C-9), 27.3 (d, C-5), 24.8 (d, C-10), 23.5 (q, C-11), 21.3 (q, C-11'), 19.84 (q, C-6), 19.75 (q, C-6').

HRMS (ESI) m/z: $[M+H]^+$ Calcd. for $C_{29}H_{37}N_2O_6^+$ 509.2646; Found 509.2646.

Fmoc-Leu-HyVal(*O*All)-Pro-O*t*-Bu [22]

First, 676 mg (1.33 mmol, 1.1 eq.) of dipeptide **21** was dissolved in 4.4 mL anhydrous DMF under N_2 atmosphere and cooled to 0 °C. Subsequently, 189 mg (1.33 mmol, 1.1 eq.) Oxyma, 258 mg (1.35 mmol, 1.1 eq.) EDC and 463 µL (2.66 mmol, 2.2 eq., ρ = 0.742 g/mL) DIPEA were added. The resulting yellow solution was stirred for 10 min at 0 °C before 213 µL (1.21 mmol, 1.0 eq., ρ = 0.971 g/mL) of H-Pro-O*t*-Bu was added and the mixture was slowly warmed to rt. After 3 h, the now dark orange solution was diluted with EtOAc and washed with 1 M $HCl_{(aq.)}$, 1 M $LiCl_{(aq.)}$, saturated $NaHCO_{3(aq.)}$ and saturated $NaCl_{(aq.)}$ solution. The organic phase was then dried over $MgSO_4$ and concentrated *in vacuo*. Automated column chromatography (SiO_2, cHex/EtOAc 10:0 ⟶ 7:3) of the crude product yielded 555 mg (838 µmol, 69 %) of tripeptide **22** as a colourless foam.

$[\alpha]_D^{20}$ = −84.5 (c = 1.0, $CHCl_3$) R_f = 0.31 (*n*-Pentane/EtOAc 7:3)

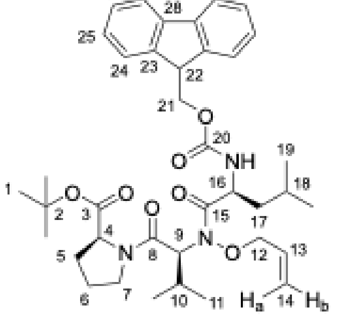

^{1}H NMR (500 MHz, DMSO-d_6, 373 K): δ = 7.85 (d, $^{3}J_{27,26}$ = 7.6 Hz, 2 H, 27-H), 7.68 (d, $^{3}J_{24,25}$ = 7.5 Hz, 2 H, 24-H), 7.41 (t, $^{3}J_{26,25}$ ≈ $^{3}J_{26,27}$ = 7.5 Hz, 2 H, 26-H), 7.31 (tdd, $^{3}J_{25,24}$ ≈ $^{3}J_{25,26}$ = 7.4 Hz, $^{4}J_{25,27}$ = 3.0 Hz, $^{5}J_{25,22}$ = 1.2 Hz, 2 H, 25-H), 7.09 (bs, 1 H, NH), 5.93 (ddt, $^{3}J_{13,14a}$ = 16.6 Hz, $^{3}J_{13,14b}$ = 11.0 Hz, $^{3}J_{13,12}$ = 5.9 Hz, 1 H, 13-H), 5.34 (d, $^{3}J_{14a,13}$ = 17.3 Hz, 1 H, 14-H$_a$), 5.26 (d, $^{3}J_{14b,13}$ = 10.5 Hz, 1 H, 14-H$_b$), 4.75 (d, $^{3}J_{9,10}$ = 10.5 Hz, 1 H, 9-H), 4.72 (bs, 1 H, 16-H), 4.61 (bs, 1 H, 12-H$_a$), 4.42 (dd, $^{2}J_{12b,12a}$ = 11.3 Hz, $^{3}J_{12b,13}$ = 6.0 Hz, 1 H, 12-H$_b$), 4.35 (m, 1 H, 21-H$_a$), 4.29 (dd, $^{2}J_{21b,21a}$ = 10.5 Hz, $^{3}J_{21b,22}$ = 6.9 Hz, 1 H, 21-H$_b$), 4.23–4.17 (m, 2 H, 4-H, 22-H), 3.51 (m, 2 H, 7-H), 2.43 (dsept, $^{3}J_{10,9}$ = 10.4 Hz, $^{3}J_{10,11}$ = 6.6 Hz, 1 H, 10-H), 2.16 (m, 1 H, 5-H$_a$), 1.96 (m, 1 H, 6-H$_a$), 1.88–1.79 (m, 2 H, 5-H$_b$, 6-H$_b$), 1.71 (m, 1 H, 18-H), 1.57 (m, 1 H, 17-H$_a$), 1.41 (s, 9 H, 1-H), 1.37 (ddd, $^{2}J_{17b,17a}$ = 13.3 Hz, $^{3}J_{17b,16/18}$ = 8.7 Hz, $^{3}J_{17b,18/16}$ = 3.4 Hz, 1 H, 17-H$_b$), 0.92 (d, $^{3}J_{11,10}$ = 7.3 Hz, 3 H, 11-H), 0.91 (d, $^{3}J_{19,18}$ = 6.9 Hz, 3 H, 19-H), 0.88 (d, $^{3}J_{11',10}$ = 6.7 Hz, 3 H, 11-H'), 0.87 (d, $^{3}J_{19',18}$ = 6.6 Hz, 3 H, 19-H').

^{13}C NMR (126 MHz, DMSO-d_6, 373 K): δ = 174.3 (s, C-8), 170.2 (s, C-3), 165.9 (s, C-15), 155.3 (s, C-20), 143.4 (s, C-23), 140.3 (s, C-28), 131.2 (d, C-13), 127.0 (d, C-26), 126.4 (d, C-25), 124.6 (d, C-24), 119.4 (d, C-27), 119.0 (t, C-14), 79.9 (s, C-2), 76.7 (t, C-12), 65.3 (t, C-21), 63.3 (d, C-9), 59.3 (d, C-4), 49.3 (d, C-16), 46.5 (d, C-22), 46.0 (t, C-7), 28.2 (t, C-5), 27.3 (d, C-10), 27.2 (q, C-1), 23.8 (t, C-6; d, C-18), 22.5 (q, C-19), 20.7 (q, C-19'), 18.9 (q, C-11), 18.2 (q, C-11').

The signal of C-17 lies beneath the solvent peak.

HRMS (ESI) m/z: [M+H]$^+$ Calcd. for $C_{38}H_{52}N_3O_7^+$ 662.3800; Found 662.3801.

Fmoc-MeHyPro(*O*Ac)-Leu-HyVal(*O*All)-Pro-O*t*-Bu [23]

According to **GP**, for Fmoc cleavage 420 mg (635 µmol, 1.0 eq.) of tripeptide **22** in 2.1 mL MeCN was reacted with 1.31 mL Et_2NH (12.7 mmol, 20 eq., ρ = 0.707 g/mL) for 1 h. For coupling, the resulting free amine was reacted with 280 mg (684 µmol, 1.1 eq.) of Fmoc-MeHyPro(*O*Ac)-OH (**14**), 101 mg (711 µmol, 1.2 eq.) Oxyma, 170 mg (708 µmol, 1.1 eq.) EDC and 173 µL (1.55 mmol, 2.5 eq., ρ = 0.920 g/mL) NMM in 6.2 mL DCM for 3 h. Following automated column chromatography (SiO_2, cHex/EtOAc 10:0 ⟶ 4:6), 419 mg (504 µmol, 81 %) of tetrapeptide **23** was isolated as a pale pink foam.

$[\alpha]_D^{20}$ = −111.2 (c = 1.0, $CHCl_3$) R_f = 0.23 (*n*-Pentane/EtOAc 1:1)

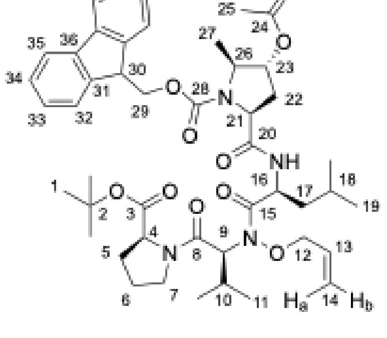

^{1}H NMR (500 MHz, DMSO-d_6, 373 K): δ = 7.85 (d, $^{3}J_{35,34}$ = 7.5 Hz, 2 H, 35-H), 7.83 (bs, 1 H, NH), 7.63 (d, $^{3}J_{32,33}$ = 7.4 Hz, 2 H, 32-H), 7.41 (t, $^{3}J_{34,33}$ ≈ $^{3}J_{34,35}$ = 7.4 Hz, 2 H, 34-H), 7.32 (td, $^{3}J_{33,32}$ ≈ $^{3}J_{33,34}$ = 7.5 Hz, $^{4}J_{33,32}$ = 1.2 Hz, 2 H, 33-H), 5.93 (ddt, $^{3}J_{13,14a}$ = 16.6 Hz, $^{3}J_{13,14b}$ = 10.4 Hz, $^{3}J_{13,12}$ = 6.0 Hz, 1 H, 13-H), 5.35 (dq, $^{3}J_{14a,13}$ = 17.3 Hz, $^{2}J_{14a,14b}$ ≈ $^{4}J_{14a,12}$ = 1.6 Hz, 1 H, 14-H$_a$), 5.25 (dq, $^{3}J_{14b,13}$ = 10.4 Hz, $^{2}J_{14b,14a}$ ≈ $^{4}J_{14b,12}$ = 1.2 Hz, 1 H, 14-H$_b$), 5.01 (td, $^{3}J_{16,NH}$ ≈ $^{3}J_{16,17a/b}$ =

9.3 Hz, $^{3}J_{16,17b/a}$ = 4.0 Hz, 1 H, 16-H), 4.83 (m, 1 H, 23-H), 4.73 (d, $^{3}J_{9,10}$ = 10.4 Hz, 1 H, 9-H), 4.66 (dd, $^{2}J_{12a,12b}$ = 11.2 Hz, $^{3}J_{12a,13}$ = 6.0 Hz, 1 H, 12-H$_a$), 4.48 (bs, 1 H, 21-H), 4.43 (ddt, $^{2}J_{12b,12a}$ = 11.2 Hz, $^{3}J_{12b,13}$ = 6.0 Hz, $^{4}J_{12b,14}$ = 1.3 Hz, 1 H, 12-H$_b$), 4.34 (bs, 1 H, 29-H$_a$), 4.30 (m, 1 H, 29-H$_b$), 4.24 (t, $^{3}J_{30,29}$ = 6.4 Hz, 1 H, 30-H), 4.22 (bs, 1 H, 4-H), 3.81 (bs, 1 H, 26-H), 3.48 (m, 2 H, 7-H), 2.44 (dsept, $^{3}J_{10,9}$ = 10.4 Hz, $^{3}J_{10,11}$ = 6.6 Hz, 1 H, 10-H), 2.24 (bs, 1 H, 22-H$_a$), 2.19–2.09 (m, 2 H, 5-H$_a$, 22-H$_b$), 2.02 (s, 3 H, 25-H), 1.93 (m, 1 H, 6-H$_a$), 1.88–1.79 (m, 2 H, 5-H$_b$, 6-H$_b$), 1.68 (m, 1 H, 18-H), 1.52 (ddd, $^{2}J_{17a,17b}$ = 13.7 Hz, $^{3}J_{17a,16/18}$ = 10.2 Hz, $^{3}J_{17a,18/16}$ = 4.9 Hz, 1 H, 17-H$_a$), 1.41 (s, 9 H, 1-H), 1.40 (m, 1 H, 17-H$_b$), 1.13 (bs, 3 H, 27-H), 0.92 (d, $^{3}J_{11,10}$ = 6.5 Hz, 3 H, 11-H), 0.86 (d, $^{3}J_{11',10}$ = 6.7 Hz, 3 H, 11-H'), 0.82 (d, $^{3}J_{19,18}$ = 6.6 Hz, 3 H, 19-H), 0.79 (d, $^{3}J_{19',18}$ = 6.6 Hz, 3 H, 19-H').

^{13}C NMR (126 MHz, DMSO-d_6, 373 K): δ = 173.9 (s, C-15), 170.4 (s, C-20), 170.2 (s, C-3), 169.1 (s, C-24), 165.8 (s, C-8), 153.4 (s, C-28), 143.5 (s, C-31), 140.3 (s, C-36), 131.2 (d, C-13), 127.0 (d, C-34), 126.5 (d, C-33), 124.4 (d, C-32), 119.4 (d, C-35), 119.0 (t, C-14), 79.9 (s, C-2), 77.4 (d, C-23), 76.6 (t, C-12), 66.3 (t, C-29), 63.2 (d, C-9), 59.6 (d, C-26), 59.3 (d, C-4), 58.2 (d, C-21), 47.1 (d, C-16), 46.5 (d, C-30), 46.0 (t, C-7), 33.4 (t, C-22), 28.1 (t, C-5), 27.3 (d, C-10), 27.2 (q, C-1), 23.8 (t, C-6), 23.7 (d, C-18), 22.4 (q, C-19), 20.9 (q, C-19'), 20.2 (q, C-25), 18.9 (q, C-11), 18.1 (q, C-11'), 17.1 (q, C-27).

HRMS (ESI) m/z: $[M+H]^+$ Calcd. for $C_{46}H_{63}N_4O_{10}^+$ 831.4539; Found 831.4539.

Fmoc-Leu-MeHyPro(*O*Ac)-Leu-HyVal(*O*All)-Pro-O*t*-Bu [24]

According to **GP**, for Fmoc cleavage 423 mg (509 µmol, 1.0 eq.) of tetrapeptide **23** in 2.0 mL MeCN was reacted with 1.05 mL Et_2NH (10.2 mmol, 20 eq., ρ = 0.707 g/mL) for 45 min. For coupling, the resulting free amine was reacted with 219 mg (621 µmol, 1.2 eq.) Fmoc-Leu-OH, 235 mg (618 µmol, 1.2 eq.) HATU (in place of Oxyma and EDC) and 141 µL (1.27 mmol, 2.5 eq., ρ = 0.920 g/mL) NMM in 5.1 mL DCM for 4 h. Following automated column chromatography (SiO_2, cHex/EtOAc 10:0 ⟶ 4:6), 469 mg (497 µmol, 98 %) of pentapeptide **24** was isolated as a colourless foam.

$[\alpha]_D^{20}$ = −108.7 (c = 1.0, $CHCl_3$) R_f = 0.23 (*n*-Pentane/EtOAc 1:1)

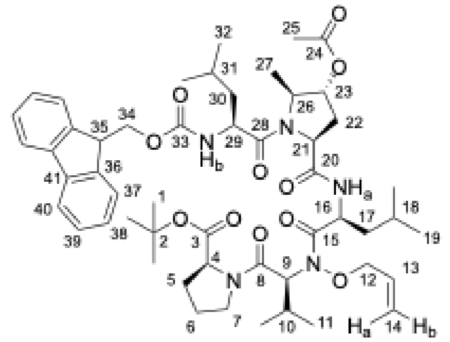

^{1}H NMR (500 MHz, DMSO-d_6, 373 K): δ = 7.85 (d, $^{3}J_{40,39}$ = 7.6 Hz, 2 H, 40-H), 7.68 (d, $^{3}J_{37,38}$ = 7.5 Hz, 2 H, 37-H), 7.41 (td, $^{3}J_{39,38}$ ≈ $^{3}J_{39,40}$ = 7.5 Hz, $^{4}J_{39,37}$ = 1.1 Hz, 2 H, 39-H), 7.32 (tdd, $^{3}J_{38,37}$ ≈ $^{3}J_{38,39}$ = 7.5 Hz, $^{4}J_{38,40}$ = 3.0 Hz, $^{5}J_{38,35}$ = 1.2 Hz, 2 H, 38-H), 5.94 (m, 1 H, 13-H), 5.35 (d, $^{3}J_{14a,13}$ = 17.1 Hz, 1 H, 14-H$_a$), 5.26 (d, $^{3}J_{14b,13}$ = 10.3 Hz, 1 H, 14-H$_b$), 5.01 (bs, 1 H, 16-H), 4.88 (bs, 1 H, 23-H), 4.74 (d, $^{3}J_{9,10}$ = 10.4 Hz, 1 H, 9-H), 4.64 (dd, $^{2}J_{12a,12b}$ = 11.5 Hz, $^{3}J_{12a,13}$ = 6.4 Hz, 1 H, 12-H$_a$), 4.59 (m, 1 H, 21-H), 4.42 (dd, $^{2}J_{12b,12a}$ = 11.2 Hz, $^{3}J_{12b,13}$ = 6.1 Hz, 1 H, 12-H$_b$), 4.39 (m,

1 H, 26-H), 4.34 (dd, $^{2}J_{34a,34b}$ = 10.3 Hz, $^{3}J_{34a,35}$ = 7.1 Hz, 1 H, 34-H_a), 4.28−4.17 (m, 4 H, 4-H, 29-H, 34-H_b, 35-H), 3.51 (bs, 2 H, 7-H), 2.44 (m, 1 H, 10-H), 2.30 (m, 1 H, 22-H_a), 2.19−2-09 (m, 2 H, 5-H_a, 22-H_b), 1.95 (m, 1 H, 6-H_a), 1.88 (s, 3 H, 25-H), 1.86−1.79 (m, 2 H, 5-H_b, 6-H_b), 1.75−1.66 (m, 2 H, 18-H, 31-H), 1.62 (bs, 1 H, 30-H_a), 1.52 (m, 1 H, 17-H_a), 1.41 (s, 9 H, 1-H), 1.44−1.33 (m, 2 H, 17-H_b, 30-H_b), 1.19 (d, $^{3}J_{27,26}$ = 6.9 Hz, 3 H, 27-H), 0.93 (d, $^{3}J_{11,10}$ = 6.5 Hz, 3 H, 11-H), 0.92 (m, 3 H, 32-H), 0.91 (d, $^{3}J_{32',31}$ = 6.6 Hz, 3 H, 32-H'), 0.90 (d, $^{3}J_{19,18}$ = 6.6 Hz, 3 H, 19-H), 0.87 (d, $^{3}J_{11,10} \approx {}^{3}J_{32,31}$ = 6.6 Hz, 6 H, 11-H, 32-H'), 0.86 (d, $^{3}J_{19',18}$ = 6.6 Hz, 3 H, 19-H').

^{13}C NMR (126 MHz, DMSO-d_6, 373 K): δ = 173.9 (s, C-15), 171.1 (s, C-20), 170.2 (s, C-3), 169.8 (s, C-28), 169.2 (s, C-24), 165.9 (s, C-8), 155.3 (s, C-33), 143.4 (s, C-36), 140.3 (s, C-41), 131.2 (d, C-13), 127.0 (d, C-39), 126.4 (d, C-38), 124.6 (d, C-37), 119.4 (d, C-40), 119.1 (t, C-14), 79.9 (s, C-2), 77.8 (d, C-23), 76.7 (t, C-12), 65.4 (t, C-34), 63.2 (d, C-9), 59.3 (d, C-4), 59.1 (d, C-26), 57.6 (d, C-21), 50.2 (d, C-29), 46.9 (d, C-16), 46.5 (d, C-35), 46.0 (t, C-7), 40.4 (t, C-17, C-30), 30.8 (t, C-22), 28.1 (t, C-5), 27.3 (d, C-10), 27.2 (q, C-1), 23.8 (t, C-6), 23.7 (d, C-18), 23.6 (d, C-31), 22.6 (q, C-32), 22.4 (q, C-19), 21.0 (q, C-32'), 20.8 (q, C-19'), 20.0 (q, C-25), 18.9 (q, C-11), 18.1 (q, C-11'), 18.0 (q, C-27)

HRMS (ESI) m/z: [M+H]$^+$ Calcd. for $C_{52}H_{74}N_5O_{11}^+$ 944.5379; Found 944.5379.

Fmoc-Thr-Leu-MeHyPro(*O*Ac)-Leu-HyVal(*O*All)-Pro-O*t*-Bu [25]

According to **GP**, for Fmoc cleavage 469 mg (497 µmol, 1.0 eq.) of pentapeptide **24** in 2.0 mL MeCN was reacted with 1.03 mL Et_2NH (9.94 mmol, 20 eq., ρ = 0.707 g/mL) for 50 min. For coupling, the resulting free amine was reacted with 208 mg (609 µmol, 1.2 eq.) Fmoc-Thr-OH, 85.8 mg (604 µmol, 1.2 eq.) Oxyma, 116 mg (605 µmol, 1.2 eq.) EDC and 138 µL (1.24 mmol, 2.5 eq., ρ = 0.920 g/mL) NMM in 5.0 mL DCM for 3 h. Following automated column chromatography (SiO_2, cHex/EtOAc 10:0 ⟶ 2:8), 432 mg (414 µmol, 83 %) of hexapeptide **25** was isolated as a pink foam.

$[\alpha]_D^{20}$ = −112.4 (c = 1.0, $CHCl_3$) R_f = 0.33 (*n*-Pentane/EtOAc 2:8)

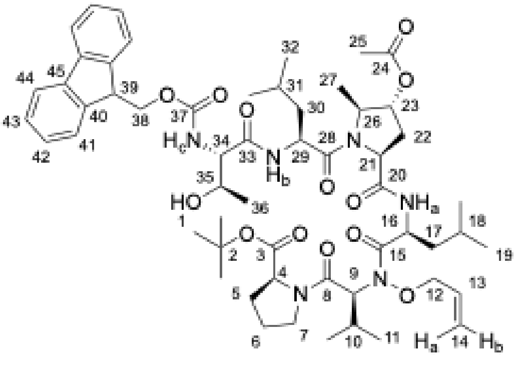

^{1}H NMR (500 MHz, DMSO-d_6): δ = 8.10 (d, $^{3}J_{NHa,16}$ = 8.6 Hz, 1 H, NH_a), 8.08 (d, $^{3}J_{NHb,29}$ = 8.1 Hz, 1 H, NH_b), 7.89 (d, $^{3}J_{44,43}$ = 7.5 Hz, 2 H, 44-H), 7.73 (m, 2 H, 41-H), 7.42 (t, $^{3}J_{43,42} \approx {}^{3}J_{43,44}$ = 7.5 Hz, 2 H, 43-H), 7.33 (t, $^{3}J_{42,41} \approx {}^{3}J_{42,43}$ = 7.5 Hz, 2 H, 42-H), 7.08 (d, $^{3}J_{NHc,34}$ = 8.9 Hz, 1 H, NH_c), 5.91 (ddt, $^{3}J_{13,14a}$ = 16.7 Hz, $^{3}J_{13,14b}$ = 10.5 Hz, $^{3}J_{13,12}$ = 6.1 Hz, 1 H, 13-H), 5.34 (dq, $^{3}J_{14a,13}$ = 17.4 Hz, $^{2}J_{14a,14b} \approx {}^{4}J_{14a,12}$ = 1.6 Hz, 1 H, 14-H_a), 5.27 (dq, $^{3}J_{14b,13}$ = 10.4 Hz, $^{2}J_{14b,14a} \approx {}^{4}J_{14b,12}$ = 1.4 Hz, 1 H, 14-H_b), 4.96 (ddd, $^{3}J_{16,17a/b}$ = 11.4 Hz, $^{3}J_{16,NHa}$ = 8.5 Hz, $^{3}J_{16,17b/a}$ = 3.6 Hz, 1 H, 16-H), 4.87 (m, 1 H, 23-H), 4.73 (d, $^{3}J_{OH,35}$ = 5.8 Hz, 1 H, OH), 4.71 (d, $^{3}J_{9,10}$ = 10.7 Hz, 1 H, 9-H), 4.60 (dd, $^{2}J_{12a,12b}$ = 11.0 Hz, $^{3}J_{12a,13}$ = 6.0 Hz, 1 H, 12-H_a), 4.53−4.43 (m, 3 H, 21-H, 26-H,

29-H), 4.39 (dd, $^2J_{12b,12a}$ = 11.1 Hz, $^3J_{12b,13}$ = 6.3 Hz, 1 H, 12-H$_b$), 4.34 (dd, $^2J_{38a,38b}$ = 9.9 Hz, $^3J_{38a,39}$ = 6.8 Hz, 1 H, 38-H$_a$), 4.29–4.19 (m, 2 H, 38-H$_b$, 39-H), 4.15 (dd, $^3J_{4,5a/b}$ = 8.6 Hz, $^3J_{4,5b/a}$ = 4.7 Hz, 1 H, 4-H), 3.91 (dd, $^3J_{34,NHc}$ = 8.9 Hz, $^3J_{34,35}$ = 5.1 Hz, 1 H, 34-H), 3.82 (m, 1 H, 35-H), 3.52 (dt, $^2J_{7a,7b}$ = 10.1 Hz, $^3J_{7a,6}$ = 6.7 Hz, 1 H, 7-H$_a$), 3.41 (dt, $^2J_{7b,7a}$ = 10.0 Hz, $^3J_{7b,6}$ = 6.7 Hz, 1 H, 7-H$_b$), 2.37 (m, 1 H, 10-H), 2.16 (m, 1 H, 5-H$_a$), 2.10 (m, 2 H, 22-H), 1.96 (s, 3 H, 25-H), 1.92 (m, 1 H, 6-H$_a$), 1.86–1.76 (m, 2 H, 5-H$_b$, 6-H$_b$), 1.73 (m, 1 H, 18-H), 1.69–1.55 (m, 2 H, 30-H$_a$, 31-H), 1.51 (m, 1 H, 17-H$_a$), 1.38 (s, 9 H, 1-H), 1.36–1.23 (m, 2 H, 17-H$_a$, 30-H$_a$), 1.19 (d, $^3J_{27,26}$ = 6.9 Hz, 3 H, 27-H), 0.98 (d, $^3J_{36,35}$ = 6.3 Hz, 3 H, 36-H), 0.90–0.86 (m, 9 H, 11-H, 19-H, 32-H), 0.84–0.82 (m, 6 H, 19-H', 32-H'), 0.80 (d, $^3J_{11',10}$ = 6.7 Hz, 3 H, 11-H').

^{13}C NMR (126 MHz, DMSO-d_6): δ = 174.5 (s, C-15), 170.8 (s, C-3), 170.6 (s, C-20), 170.5 (s, C-28), 170.1 (s, C-33), 170.0 (s, C-24), 166.3 (s, C-8), 155.9 (s, C-37), 143.8 (s, C-40), 140.7 (s, C-45), 131.6 (d, C-13), 127.6 (d, C-43), 127.1 (d, C-42), 125.3 (d, C-41), 120.1 (d, C-44), 120.0 (t, C-14), 80.3 (s, C-2), 78.0 (d, C-23), 77.3 (t, C-12), 66.8 (d, C-35), 65.7 (t, C-38), 63.1 (d, C-9), 60.5 (d, C-34), 59.7 (d, C-4), 59.6 (d, C-26), 57.9 (d, C-21), 48.0 (d, C-29), 46.9 (d, C-16), 46.7 (d, C-39), 46.5 (t, C-7), 40.8 (t, C-30), 40.3 (t, C-17), 31.9 (t, C-22), 28.7 (t, C-5), 27.6 (q, C-1), 24.4 (t, C-6), 23.9 (d, C-18), 23.8 (d, C-31), 23.4 (q, C-32), 23.3 (q, C-19), 21.6 (q, C-32'), 21.2 (q, C-19'), 20.8 (q, C-25), 19.9 (q, C-36), 19.4 (q, C-11), 18.7 (q, C-27), 18.5 (q, C-11').

HRMS (ESI) m/z: [M+H]$^+$ Calcd. for $C_{56}H_{81}N_6O_{13}^+$ 1045.5856; Found 1045.5855.

SacCl(*O*All)-Thr-Leu-MeHyPro(*O*Ac)-Leu-HyVal(*O*All)-Pro-O*t*-Bu [26]

According to **GP**, for Fmoc cleavage 77.9 mg (74.5 µmol, 1.0 eq.) of hexapeptide **25** in 0.3 mL MeCN was reacted with 154 µL Et_2NH (1.49 mmol, 20 eq., ρ = 0.707 g/mL) for 50 min. For coupling, the resulting free amine was reacted with 44.8 mg (148 µmol, 2.0 eq.) of carboxylic acid **18**, 21.5 mg (151 µmol, 2.0 eq.) Oxyma, 32.6 mg (170 µmol, 2.3 eq.) EDC and 29.0 µL (261 µmol, 3.5 eq., ρ = 0.910 g/mL) NMM in 0.75 mL DCM for 3 h. Following automated column chromatography (SiO_2, cHex/EtOAc 10:0 ⟶ 1:9), 68.1 mg (61.5 µmol, 82 %) of hexapeptide **26** was isolated as a pale yellow foam.

$[\alpha]_D^{20}$ = −108.6 (c = 1.0, $CHCl_3$)

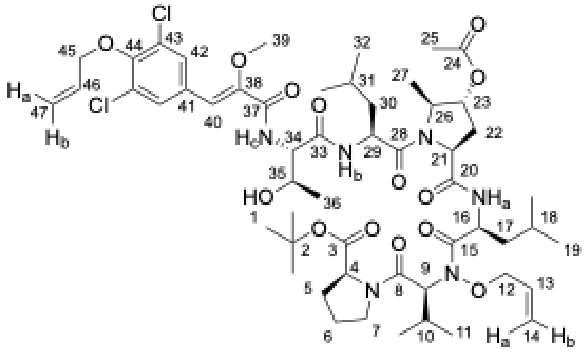

^{1}H NMR (500 MHz, DMSO-d_6, 373 K): δ = 7.89 (d, $^3J_{NHb,29}$ = 7.9 Hz, 1 H, NH$_b$), 7.74 (s, 2 H, 42-H), 7.67 (d, $^3J_{NHa,16}$ = 8.2 Hz, 1 H, NH$_a$), 7.60 (d, $^3J_{NHc,34}$ = 8.5 Hz, 1 H, NH$_c$), 6.58 (s, 1 H, 40-H), 6.12 (ddt, $^3J_{46,47a}$ = 17.2 Hz, $^3J_{46,47b}$ = 10.4 Hz, $^3J_{46,45}$ = 5.9 Hz, 1 H, 46-H), 5.94 (ddt, $^3J_{13,14a}$ = 16.8 Hz, $^3J_{13,14b}$ = 10.5 Hz, $^3J_{13,12}$ = 6.0 Hz, 1 H, 13-H), 5.43 (dq, $^3J_{47a,46}$ = 17.1 Hz, $^2J_{47a,47b}$ ≈ $^4J_{47a,45}$ = 1.6 Hz, 1 H, 47-H$_a$), 5.36 (dq, $^3J_{14a,13}$ = 17.4 Hz, $^2J_{14a,14b}$ ≈ $^4J_{14a,12}$ = 1.6 Hz, 1 H, 14-H$_a$), 5.29 (dq, $^3J_{47b,46}$ = 10.5 Hz, $^2J_{47b,47a}$ ≈ $^4J_{47b,45}$ = 1.3 Hz, 1 H, 47-H$_b$), 5.27 (m, 1 H, 14-H$_b$), 5.02 (m,

1 H, 16-H), 4.91 (bs, 1 H, 23-H), 4.74 (d, $^3J_{9,10}$ = 10.5 Hz, 1 H, 9-H), 4.64 (m, 1 H, 12-H$_a$), 4.61 (dt, $^3J_{45,46}$ = 5.8 Hz, $^4J_{45,47}$ = 1.4 Hz, 2 H, 45-H), 4.54–4.60 (m, 2 H, 21-H, 29-H), 4.48–4.40 (m, 2 H, 12-H$_b$, 26-H), 4.34 (dd, $^3J_{34,NHc}$ = 8.4 Hz, $^3J_{34,35}$ = 4.7 Hz, 1 H, 34-H), 4.21 (bs, 1 H, 4-H), 4.04 (bs, 1 H, 35-H), 3.72 (s, 3 H, 39-H), 3.51 (m, 2 H, 7-H), 2.44 (dsept, $^3J_{10,9}$ = 10.4 Hz, $^3J_{10,11}$ = 6.6 Hz, 1 H, 10-H), 2.28 (m, 1 H, 22-H$_a$), 2.20–2.10 (m, 2 H, 5-H$_a$, 22-H$_b$), 1.99 (s, 3 H, 25-H), 1.97 (m, 1 H, 6-H$_a$), 1.89–1.79 (m, 2 H, 5-H$_b$, 6-H$_b$), 1.76–1.67 (m, 2 H, 18-H, 31-H), 1.63 (m, 1 H, 30-H$_a$), 1.52 (m, 1 H, 17-H$_a$), 1.41 (s, 9 H, 1-H), 1.46–1.38 (m, 2 H, 17-H$_b$, 30-H$_b$), 1.24 (d, $^3J_{27,26}$ = 6.8 Hz, 3 H, 27-H), 1.11 (d, $^3J_{36,35}$ = 6.3 Hz, 3 H, 36-H), 0.93 (d, $^3J_{11,10}$ = 6.6 Hz, 3 H, 11-H), 0.87 (d, $^3J_{11',10}$ = 6.6 Hz, 3 H, 11-H), 0.96–0.85 (m, 12 H, 19-H, 19-H', 32-H, 32-H').

^{13}C NMR (126 MHz, DMSO-d_6, 373 K): δ = 173.9 (s, C-15), 170.6 (s, C-20), 170.2 (s, C-3), 169.7 (s, C-28), 169.2 (s, C-24), 169.0 (s, C-33), 165.9 (s, C-8), 162.2 (s, C-37), 150.4 (s, C-38), 149.4 (s, C-44), 132.6 (d, C-46), 131.2 (s, C-41), 131.1 (d, C-13), 128.9 (d, C-42), 128.1 (s, C-43), 119.1 (t, C-14), 118.1 (t, C-47), 113.0 (d, C-40), 79.9 (s, C-2), 77.7 (d, C-23), 76.7 (t, C-12), 73.6 (t, C-45), 66.1 (d, C-35), 63.2 (d, C-9), 59.3 (d, C-4), 59.2 (d, C-26), 58.5 (q, C-39), 58.2 (d, C-34), 57.6 (d, C-21), 48.0 (d, C-29), 46.9 (d, C-16), 46.0 (t, C-7), 40.8 (t, C-30), 40.4 (t, C-17), 31.1 (t, C-22), 28.1 (t, C-5), 27.3 (d, C-10), 27.2 (q, C-1), 23.8 (t, C-6), 23.6 (d, C-18, C-31), 22.6 (q, C-32), 22.5 (q, C-19), 21.2 (q, C-32'), 20.9 (q, C-19'), 20.1 (q, C-25), 19.3 (q, C-36), 18.9 (q, C-11), 18.2 (q, C-27), 18.1 (q, C-11').

HRMS (ESI) m/z: [M+H]$^+$ Calcd. for $C_{54}H_{81}Cl_2N_6O_{14}^+$ 1107.5182; Found 1107.5181.

Bonnevillamide C [27]

Under N_2 atmosphere, 29.8 mg (26.9 µmol, 1.0 eq.) of hexapeptide **26** was dissolved in 1.0 mL anhydrous DCM and 22.1 mg (142 µmol, 6.6 eq.) of DMBA and 0.7 mg (0.61 µmol, 2 mol%) of $Pd(PPh_3)_4$ were added. After 4 h, the now orange solution was diluted with EtOAc and washed three times with saturated $NaHCO_{3(aq.)}$ solution. The organic phase was then dried over $MgSO_4$ and concentrated *in vacuo*. The obtained residue was dissolved in 0.5 mL DCM and treated with 50 µL TESH, followed by 450 µL TFA, and stirred for 1 h. The now brown reaction mixture was dried *in vacuo*, and the residue was co-evaporated thrice with $CHCl_3$ to remove residual TFA. Finally, the crude product was purified by automated column chromatography (C_{18}-SiO_2, H_2O/MeCN +0.1 % HCOOH 9:1 → 1:9). After lyophilisation, 13.5 mg (13.9 µmol, 52 %) of bonnevillamide C (**27**) was isolated as a colourless lyophilisate.

$[\alpha]_D^{20}$ = −40.1 (c = 0.5, $CHCl_3$), Lit.: $[\alpha]_D^{20}$ = −40 (c = 0.1, $CHCl_3$)[17]

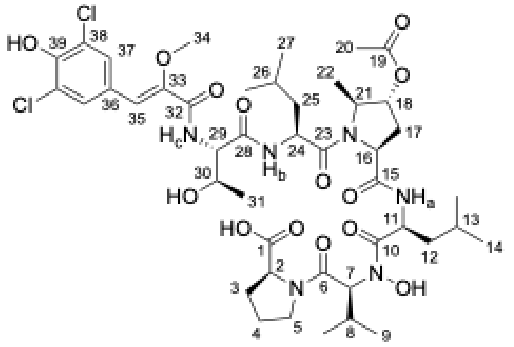

^{1}H NMR (500 MHz, DMSO-d_6): δ = 12.36 (bs, 1 H, COOH), 10.51 (bs, 1 H, OH_{Ar}), 9.55 (s, 1 H, NOH), 8.26 (d, $^{3}J_{NHb,24}$ = 8.0 Hz, 1 H, NH_b), 7.82 (d, $^{3}J_{NHa,11}$ = 8.7 Hz, 1 H, NH_a), 7.71 (s, 2 H, 37-H), 7.65 (d, $^{3}J_{NHc,29}$ = 8.7 Hz, 1 H, NH_c), 6.66 (s, 1 H, 35-H), 4.96 (ddd, $^{3}J_{11,12b}$ = 11.5 Hz, $^{3}J_{11,NHa}$ = 8.6 Hz, $^{3}J_{11,12a}$ = 3.4 Hz, 1 H, 11-H), 4.92 (d, $^{3}J_{OHsec,30}$ = 5.7 Hz, 1 H, OH), 4.88 (m, 1 H, 18-H), 4.69 (d, $^{3}J_{7,8}$ = 10.4 Hz, 1 H, 7-H), 4.51 (t, $^{3}J_{16,17}$ = 8.8 Hz, 1 H, 16-H), 4.49–4.42 (m, 2 H, 21-H, 24-H), 4.26 (dd, $^{3}J_{29,NHc}$ = 8.6 Hz, $^{3}J_{29,30}$ = 4.9 Hz, 1 H, 29-H), 4.20 (dd, $^{3}J_{2,3a/b}$ = 8.6 Hz, $^{3}J_{2,3b/a}$ = 4.5 Hz, 1 H, 2-H), 3.95 (h, $^{3}J_{30,29}$ ≈ $^{3}J_{30,31}$ ≈ $^{3}J_{30,OHsec}$ = 5.9 Hz, 1 H, 30-H), 3.66 (s, 3 H, 34-H), 3.49 (m, 2 H, 5-H), 2.32 (m, 1 H, 8-H), 2.16 (m, 3 H, 3-H_a, 17-H), 1.99 (s, 3 H, 20-H), 1.90 (m, 1 H, 4-H_a), 1.86–1.78 (m, 2 H, 3-H_b, 4-H_b), 1.70–1.57 (m, 3 H, 13-H, 25-H_a, 26-H), 1.49 (ddd, $^{3}J_{12a,12b}$ = 13.2 Hz, $^{3}J_{12a,13}$ = 9.6 Hz, $^{3}J_{12a,11}$ = 3.5 Hz, 1 H, 12-H_a), 1.37–1.27 (m, 2 H, 12-H_b, 25-H_b), 1.18 (d, $^{3}J_{22,21}$ = 6.8 Hz, 3 H, 22-H), 1.05 (d, $^{3}J_{31,30}$ = 6.2 Hz, 3 H, 31-H), 0.90 (d, $^{3}J_{27,26}$ = 6.4 Hz, 3 H, 27-H), 0.87 (d, $^{3}J_{9,8}$ = 6.7 Hz, 3 H, 9-H), 0.85 (d, $^{3}J_{14,13}$ = 5.8 Hz, 3 H, 14-H), 0.84–0.82 (m, 6 H, 14-H', 27-H'), 0.79 (d, $^{3}J_{9',8}$ = 6.7 Hz, 3 H, 9-H').

^{13}C NMR (126 MHz, DMSO-d_6): δ = 173.2 (s, C-1), 172.9 (s, C-10), 170.8 (s, C-23), 170.2 (s, C-15), 170.1 (s, C-19), 169.7 (s, C-28), 166.9 (s, C-6), 162.7 (s, C-32), 149.9 (s, C-39)*, 148.7 (s, C-33)*, 129.3 (s, C-36; d, C-37), 122.3 (s, C-38), 115.8 (d, C-35), 78.0 (d, C-18), 66.7 (d, C-30), 62.0 (d, C-7), 59.6 (d, C-21), 58.9 (q, C-34), 58.7 (d, C-2), 58.6 (d, C-29), 57.9 (d, C-16), 48.1 (d, C-24), 47.6 (d, C-11), 46.5 (t, C-5), 40.8 (t, C-25), 40.4 (t, C-12), 31.7 (t, C-17), 28.7 (t, C-3), 26.3 (d, C-8), 24.5 (t, C-4), 24.2 (d, C-13), 23.9 (d, C-26), 23.4 (q, C-14), 23.2 (q, C-27), 21.6 (q, C-27'), 21.0 (q, C-14'), 20.9 (q, C-20), 20.3 (q, C-31), 19.0 (q, C-9), 18.7 (q, C-22), 18.6 (q, C-9').

HRMS (ESI) m/z: $[M+H]^+$ Calcd. for $C_{44}H_{65}Cl_2N_6O_{14}^+$ 971.3930; Found 971.3930.

Bonnevillamide B [28]

A solution of 7.2 mg (7.41 µmol, 1.0 eq.) bonnevillamide C (**27**) in 0.7 mL MeOH was treated with 4.2 mg (30.4 µmol, 4.1 eq.) K_2CO_3 and stirred for 5 h. The mixture was then diluted with EtOAc and washed with 1 M $KHSO_{4(aq.)}$ solution. The aqueous phase was extracted twice with EtOAc before the combined organic phases were dried over $MgSO_4$ and the solvent was removed *in vacuo*. Finally, the residue was purified by automated column chromatography (C_{18}-SiO_2, H_2O/MeCN +0.1 % HCOOH 9:1 ⟶ 4:6), followed by preparative HPLC (C_{18}-SiO_2, H_2O/MeCN +0.1 % HCOOH 9:1 ⟶ 4:6). Following lyophilisation of the isolated fraction, 4.2 mg (4.52 µmol, 61 %) of bonnevillamide B (**28**) was obtained as a colourless lyophilisate.

$[\alpha]_D^{20}$ = −26.0 (c = 0.1, $CHCl_3$), Lit.: $[\alpha]_D^{20}$ = −25 (c = 0.1, $CHCl_3$)[17]

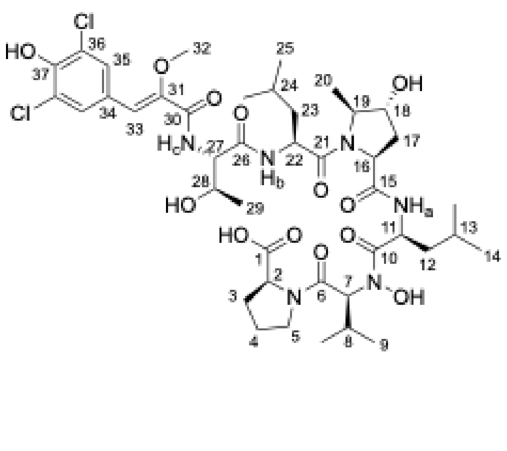

^{1}H NMR (500 MHz, DMSO-d_6): δ = 12.36 (bs, 1 H, COOH), 10.51 (bs, 1 H, OH_{Ar}), 9.56 (s, 1 H, NOH), 8.22 (t, $^{3}J_{NHb,22}$ = 8.4 Hz, 1 H, NH_b), 7.78 (d, $^{3}J_{NHa,11}$ = 8.3 Hz, 1 H, NH_a), 7.71 (s, 2 H, 35-H), 7.66 (d, $^{3}J_{NHc,27}$ = 8.9 Hz, 1 H, NH_c), 6.68 (s, 1 H, 33-H), 5.02 (d, $^{3}J_{OHMeHyPro,18}$ = 2.7 Hz, 1 H, $OH_{MeHyPro}$), 4.99 (d, $^{3}J_{OHThr,28}$ = 6.2 Hz, 1 H, OH), 4.94 (m, 1 H, 11-H), 4.69 (d, $^{3}J_{7,8}$ = 10.4 Hz, 1 H, 7-H), 4.66 (m, 1 H, 22-H), 4.46 (t, $^{3}J_{16,17}$ = 8.6 Hz, 1 H, 16-H), 4.32 (dd, $^{3}J_{27,NHc}$ = 8.8 Hz, $^{3}J_{27,28}$ = 4.2 Hz, 1 H, 27-H), 4.20 (dd, $^{3}J_{2,3a/b}$ = 8.5 Hz, $^{3}J_{2,3b/a}$ = 4.6 Hz, 1 H, 2-H), 4.05 (m, 1 H, 19-H), 4.00 (m, 1 H, 28-H), 3.92 (bs, 1 H, 18-H), 3.67 (s, 3 H, 32-H), 3.49 (m, 2 H, 5-H), 2.33 (m, 1 H, 8-H), 2.17−2.06 (m, 1 H, 3-H_a), 1.99 (m, 2 H, 17-H), 1.90 (m, 1 H, 4-H_a), 1.87−1.74 (m, 2 H, 3-H_b, 4-H_b), 1.65 (m, 1 H, 13-H), 1.60 (m, 1 H, 24-H), 1.56−1.45 (m, 2 H, 12-H_a, 23-H_a), 1.43−1.31 (m, 2 H, 12-H_b, 23-H_b), 1.12 (d, $^{3}J_{20,19}$ = 6.7 Hz, 3 H, 20-H), 1.05 (d, $^{3}J_{29,28}$ = 6.3 Hz, 3 H, 29-H), 0.93−0.82 (m, 12 H, 9-H, 14-H, 25-H, 25-H'), 0.83 (d, $^{3}J_{14',13}$ = 6.1 Hz, 3 H, 14-H'), 0.78 (d, $^{3}J_{9',8}$ = 6.6 Hz, 3 H, 9-H').

^{13}C NMR (126 MHz, DMSO-d_6): δ = 173.2 (s, C-1), 173.0 (s, C-10), 170.9 (s, C-15), 170.6 (s, C-21), 169.3 (s, C-26), 166.9 (s, C-6), 162.7 (s, C-30), 149.6 (s, C-37), 148.6 (s, C-31), 129.4 (d, C-35), 129.3 (s, C-34), 122.3 (s, C-36), 115.9 (d, C-33), 74.9 (d, C-18), 67.0 (d, C-28), 61.9 (d, C-7, C-19), 59.0 (q, C-32), 58.7 (d, C-2), 58.3 (d, C-16, C-27), 47.8 (d, C-22), 47.4 (d, C-11), 46.4 (t, C-5), 41.5 (t, C-23), 40.4 (t, C-12), 34.8 (t, C-17), 28.7 (t, C-3), 26.3 (d, C-8), 24.5 (t, C-4), 24.2 (d, C-13), 24.0 (d, C-24), 23.4 (s, C-14), 23.2 (s, C-25), 21.8 (s, C-25'), 21.1 (q, C-14'), 20.3 (q, C-29), 19.0 (q, C-9), 18.8 (q, C-20), 18.5 (q, C-9').

HRMS (ESI) m/z: $[M+H]^+$ Calcd. for $C_{42}H_{62}Cl_2N_6O_{13}^+$ 929.3825; Found 929.3791.

NMR Spectra

(*S*)-But-3-yn-2-yl-((benzyloxy)carbonyl)glycinate [1]

^{1}H NMR (500 MHz, $CDCl_3$, **1**):

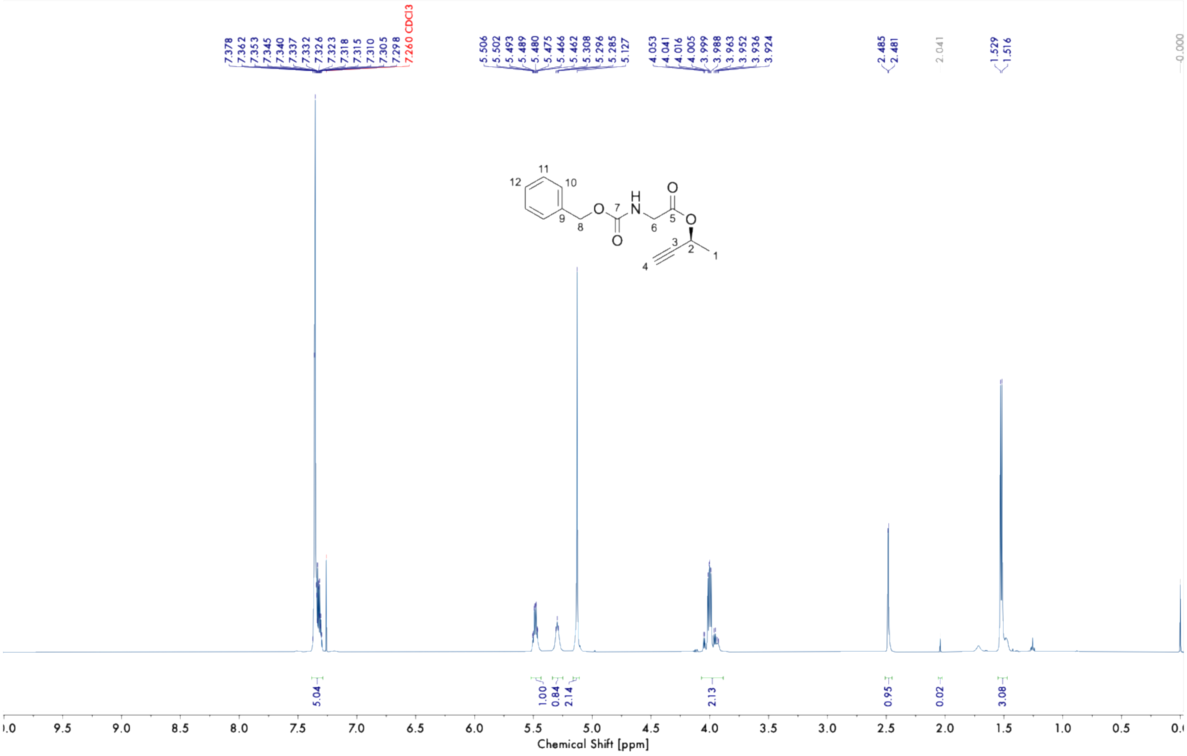

^{13}C NMR (126 MHz, $CDCl_3$, **1**):

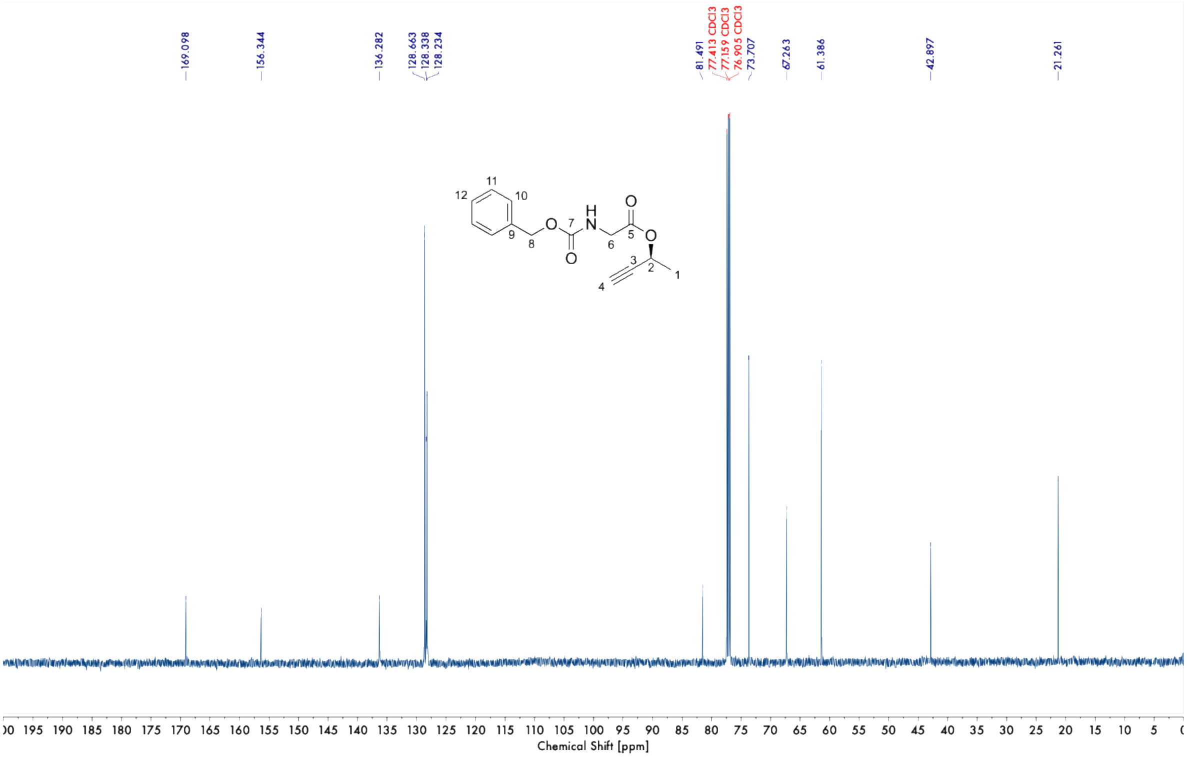

(^{1}H,^{1}H)-COSY (CDCl$_3$, **1**):

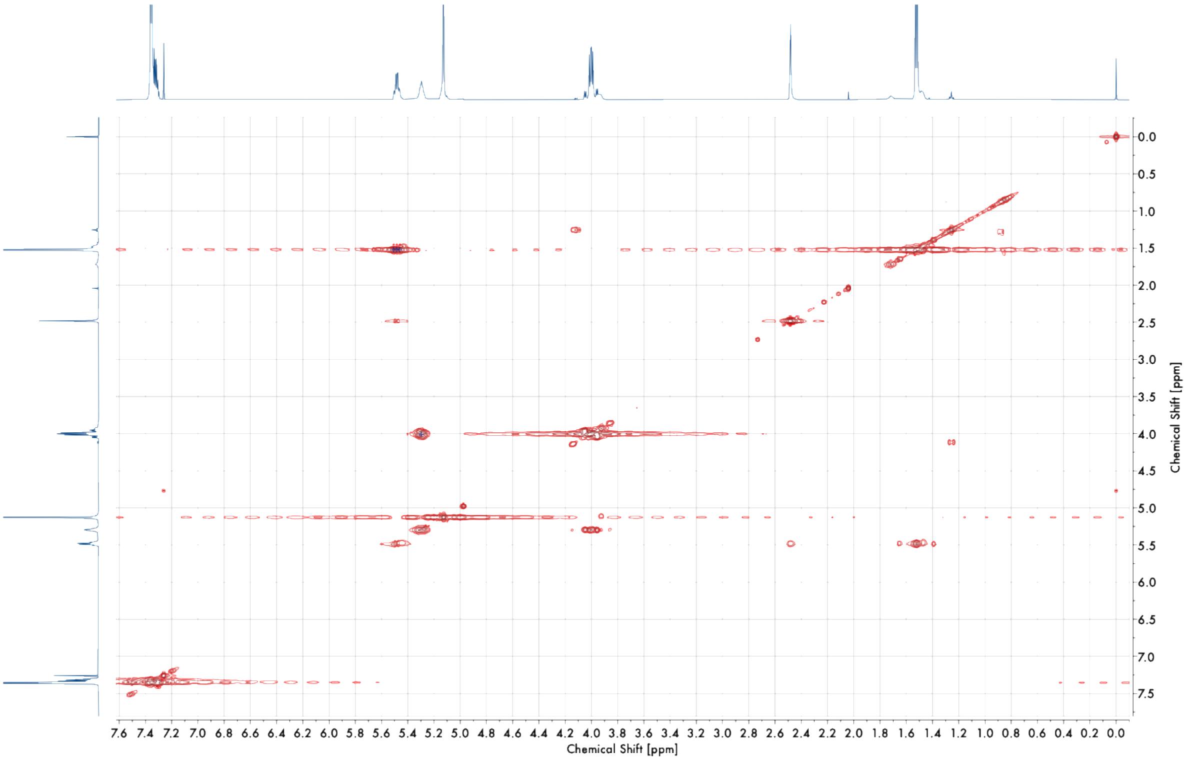

(^{1}H,^{13}C)-HSQC (CDCl$_3$, **1**):

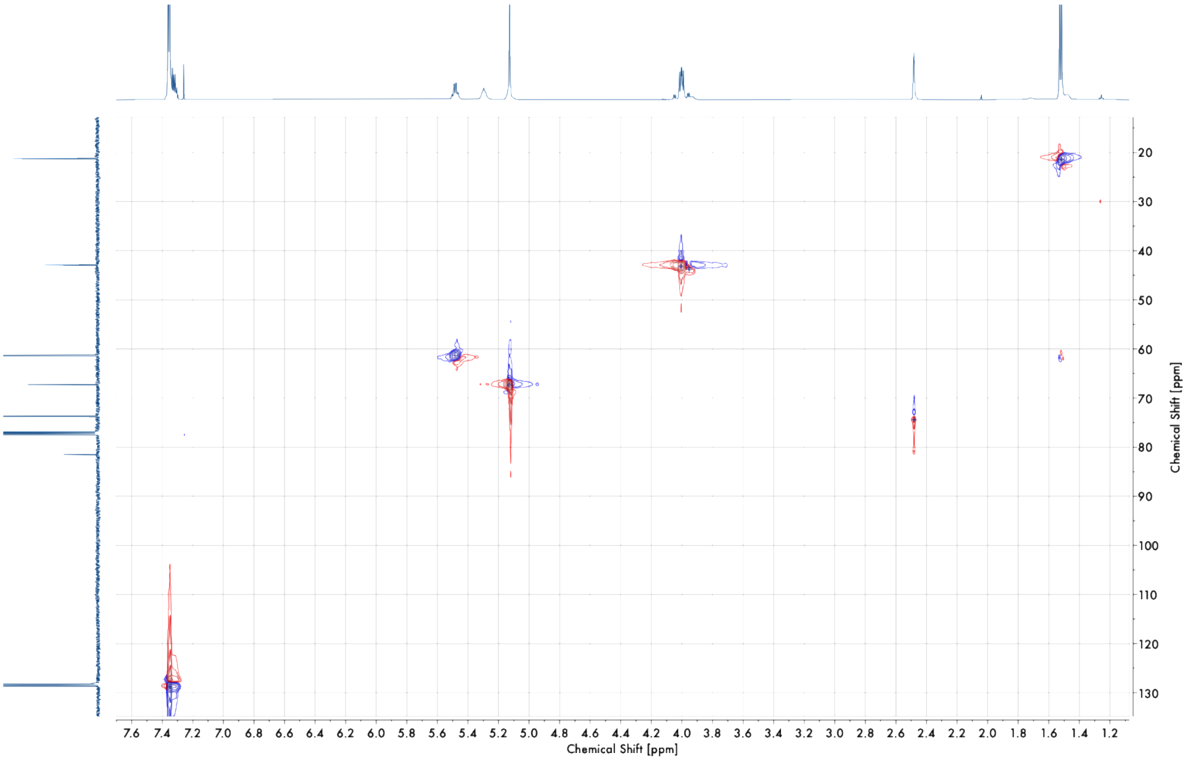

(^{1}H,^{13}C)-HMBC (CDCl$_3$, **1**):

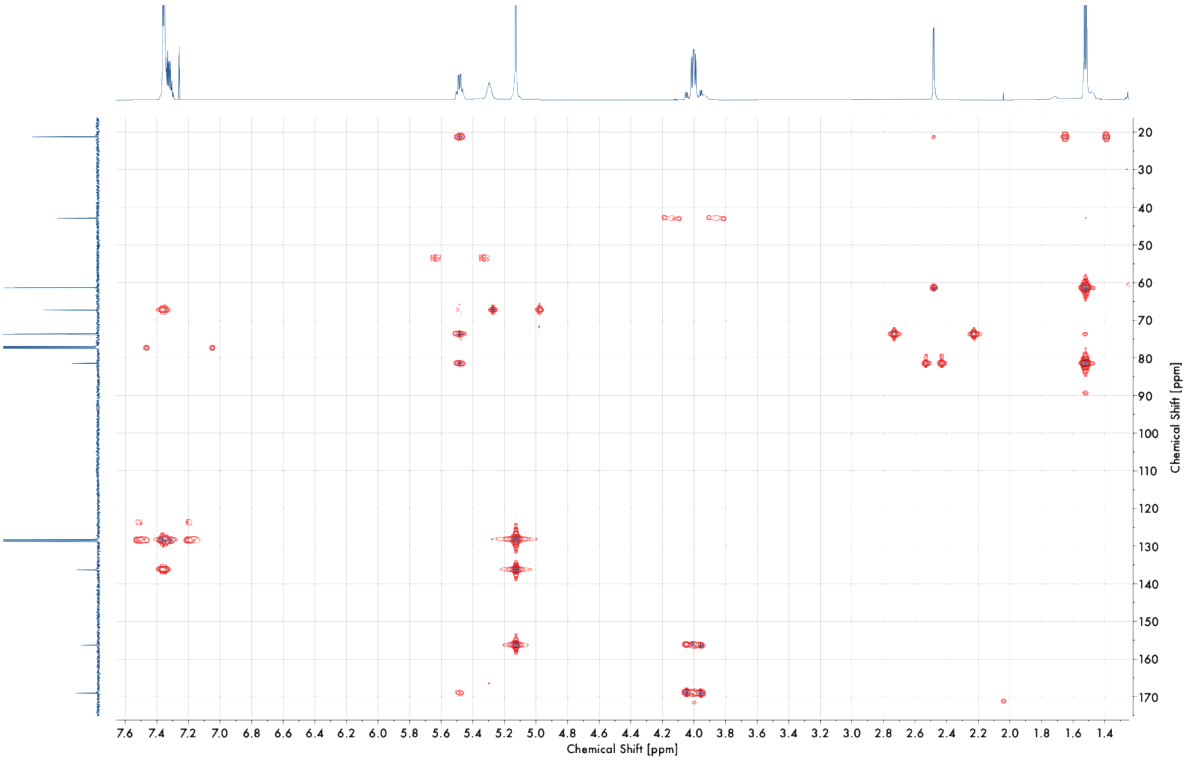

(*S*)-But-3-en-2-yl-((benzyloxy)carbonyl)glycinate [2]

^{1}H NMR (500 MHz, CDCl$_3$, **2**):

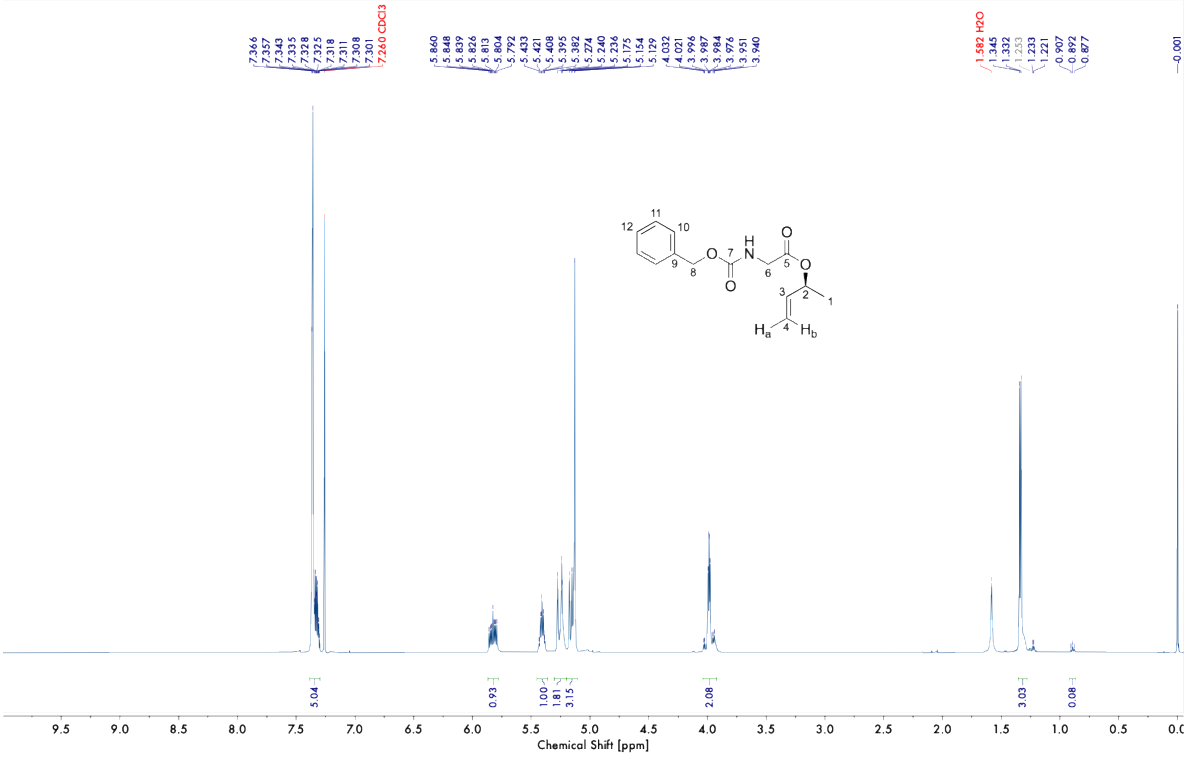

^{13}C NMR (126 MHz, CDCl$_3$, **2**):

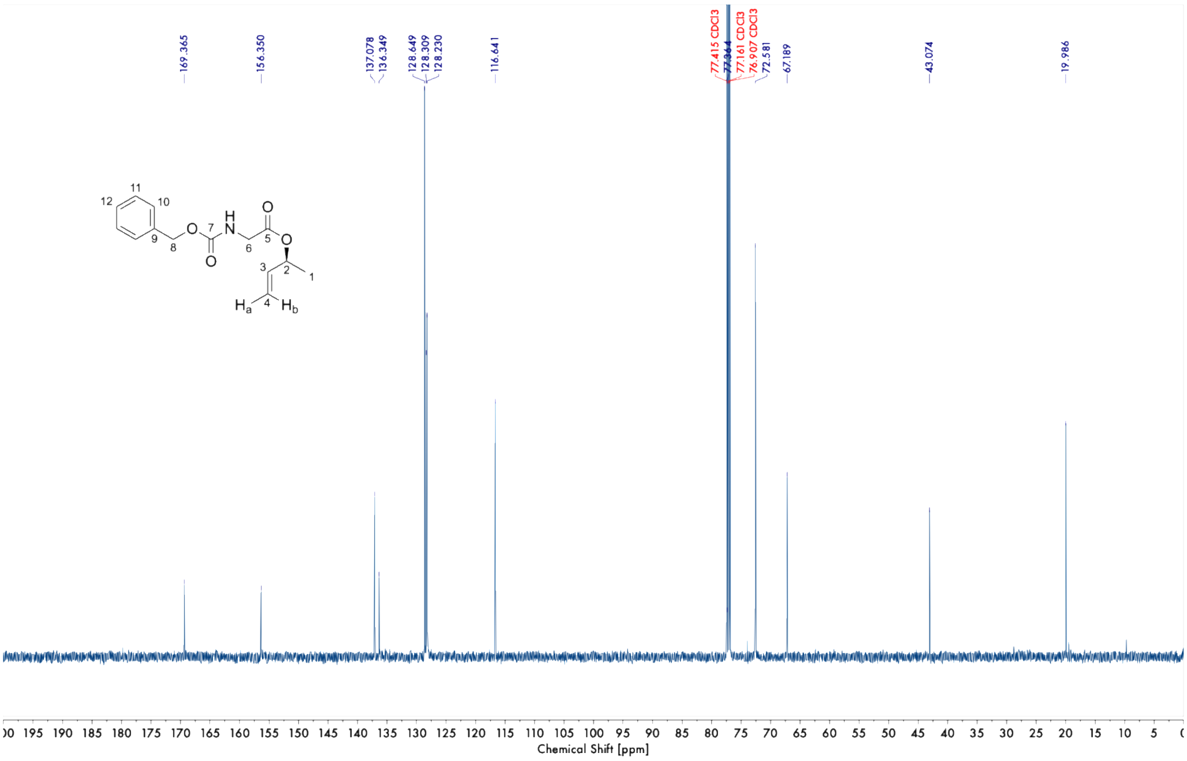

(^{1}H,^{1}H)-COSY (CDCl$_3$, **2**):

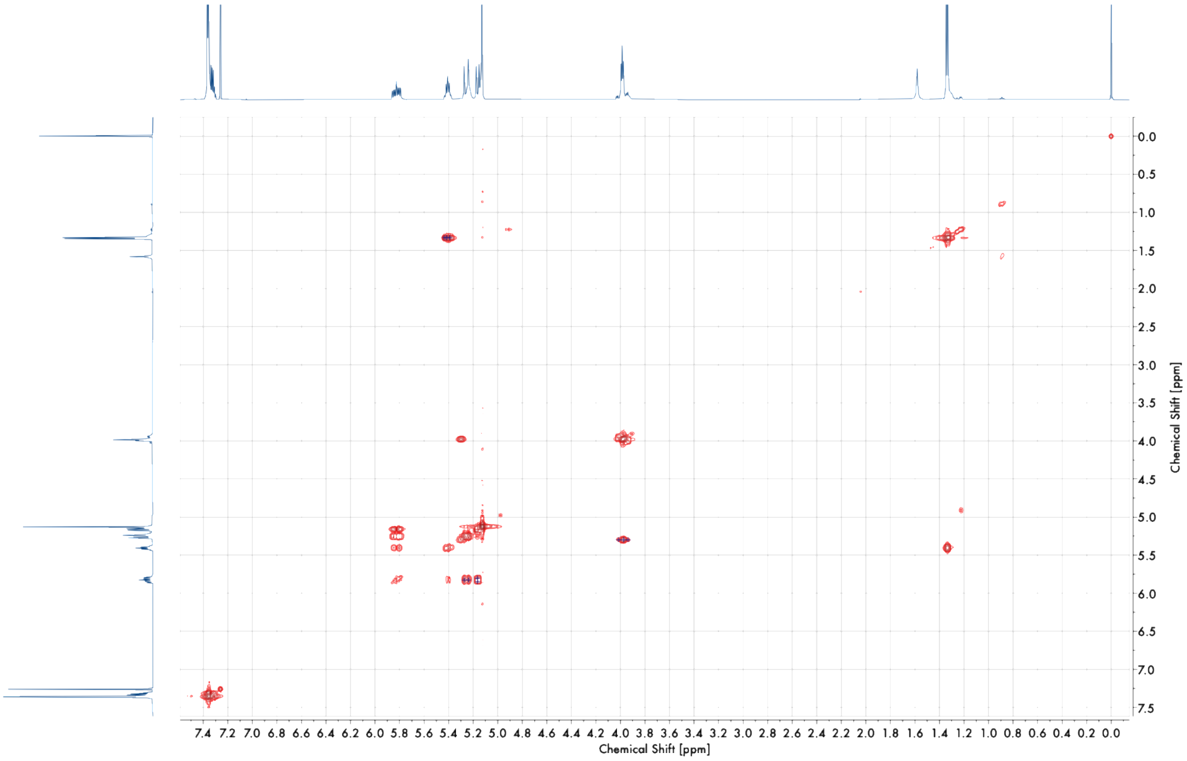

(¹H,¹³C)-HSQC (CDCl$_3$, **2**):

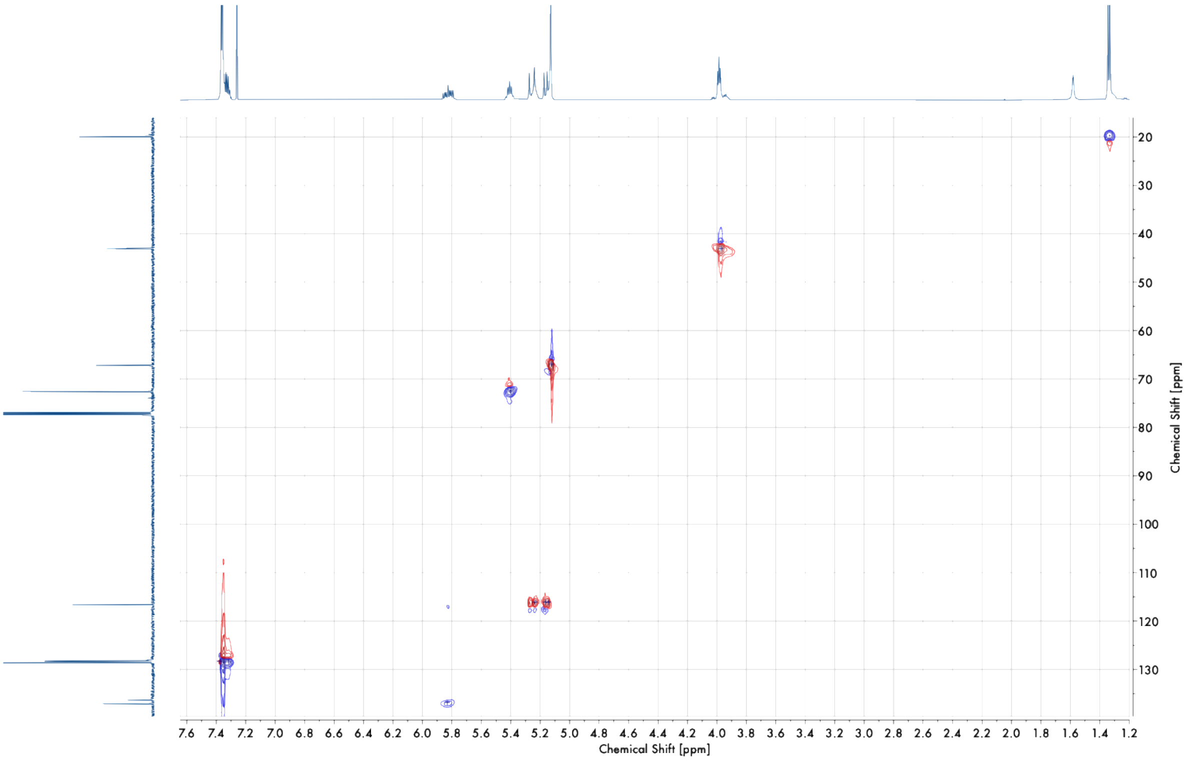

(¹H,¹³C)-HMBC (CDCl$_3$, **2**):

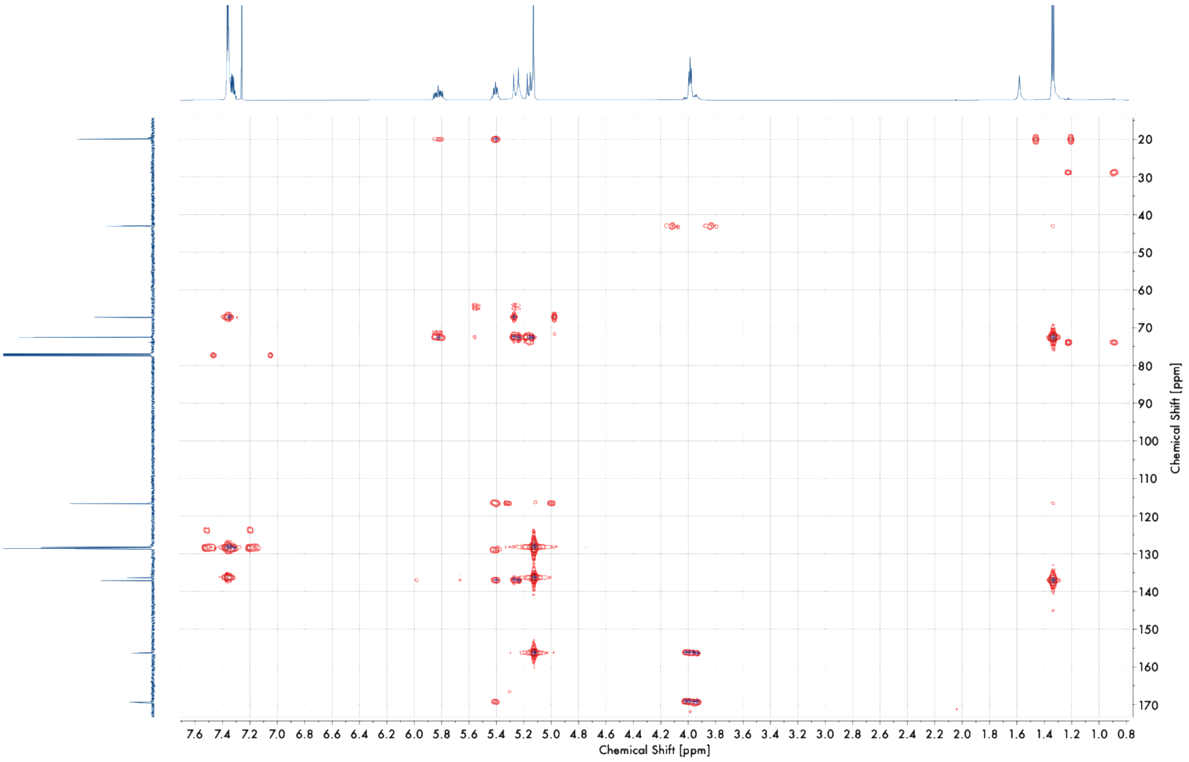

(*S*,*E*)-2-((Benzyloxycarbonyl)amino)hex-4-enoic acid [3]

^{1}H NMR (500 MHz, $CDCl_3$, **3**):

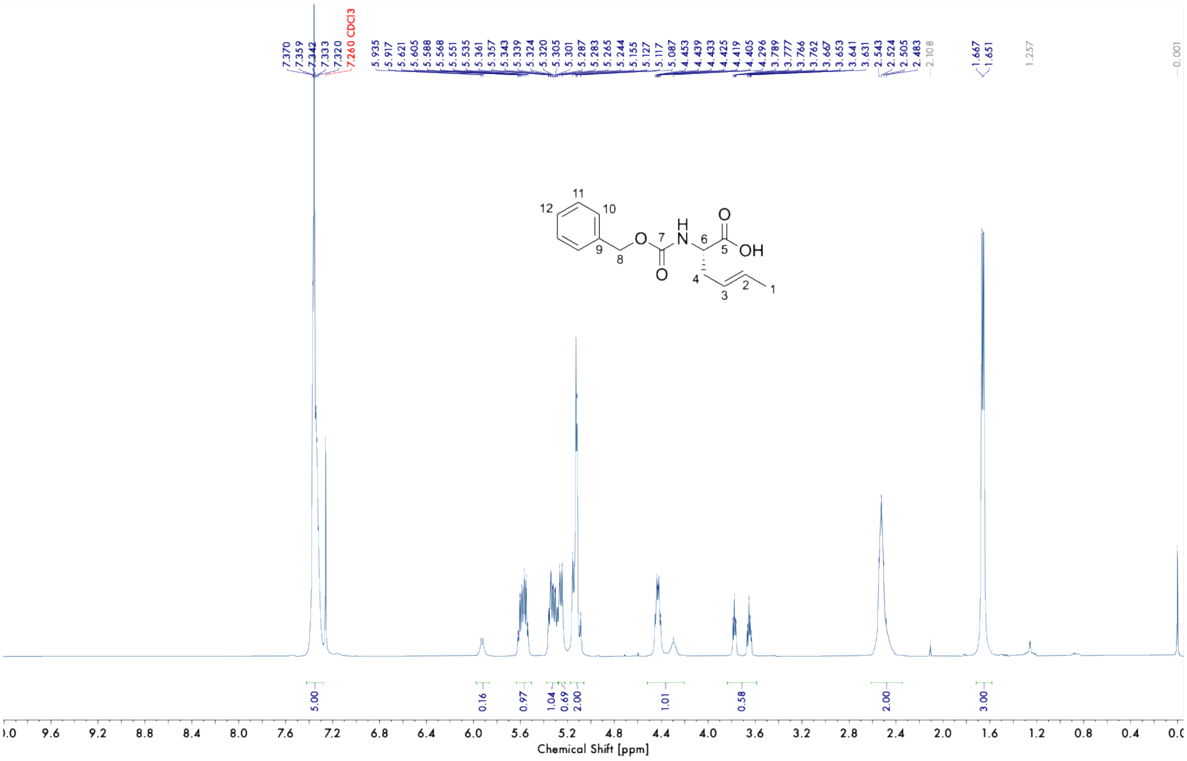

^{13}C NMR (126 MHz, $CDCl_3$, **3**):

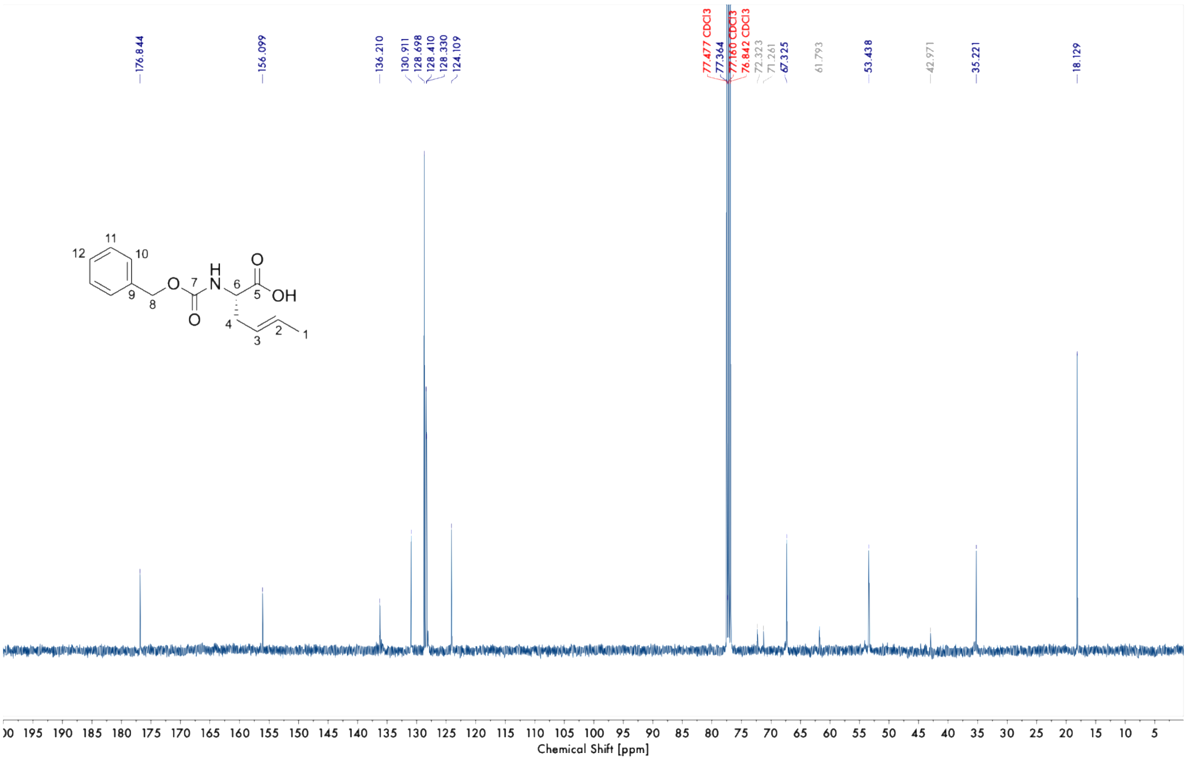

(^{1}H,^{1}H)-COSY (CDCl$_3$, **3**):

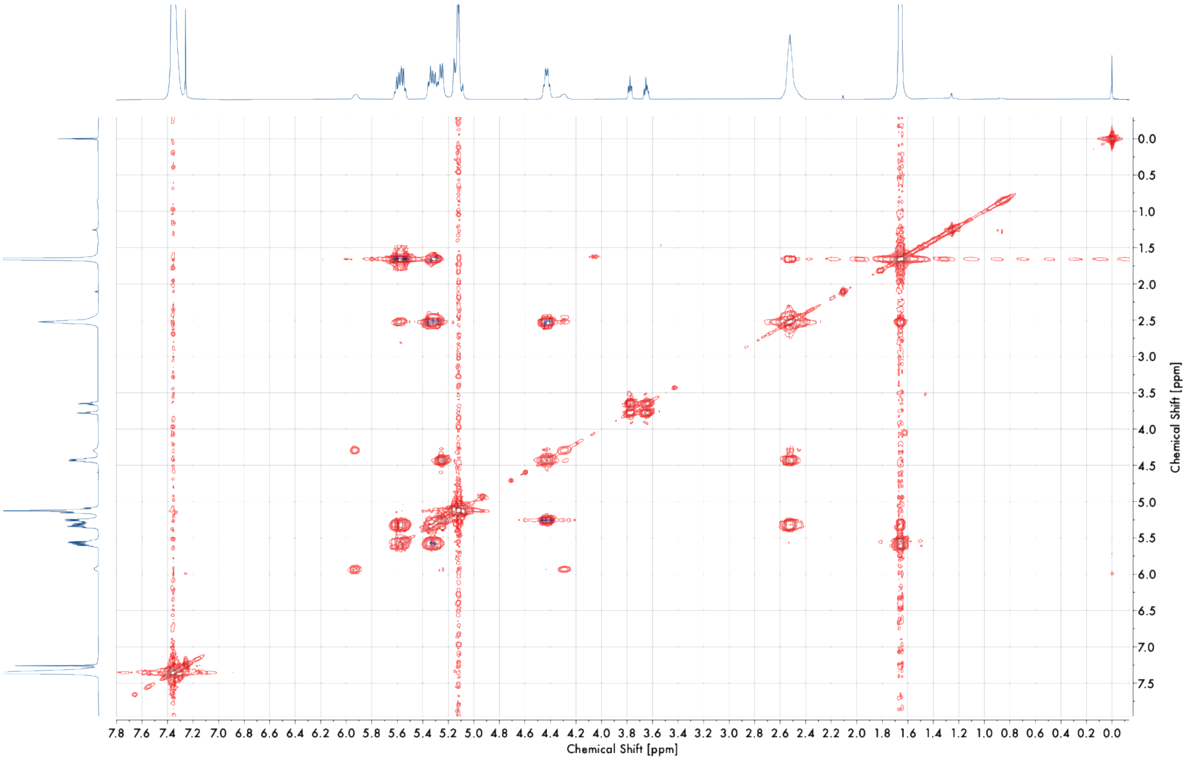

(^{1}H,^{13}C)-HSQC (CDCl$_3$, **3**):

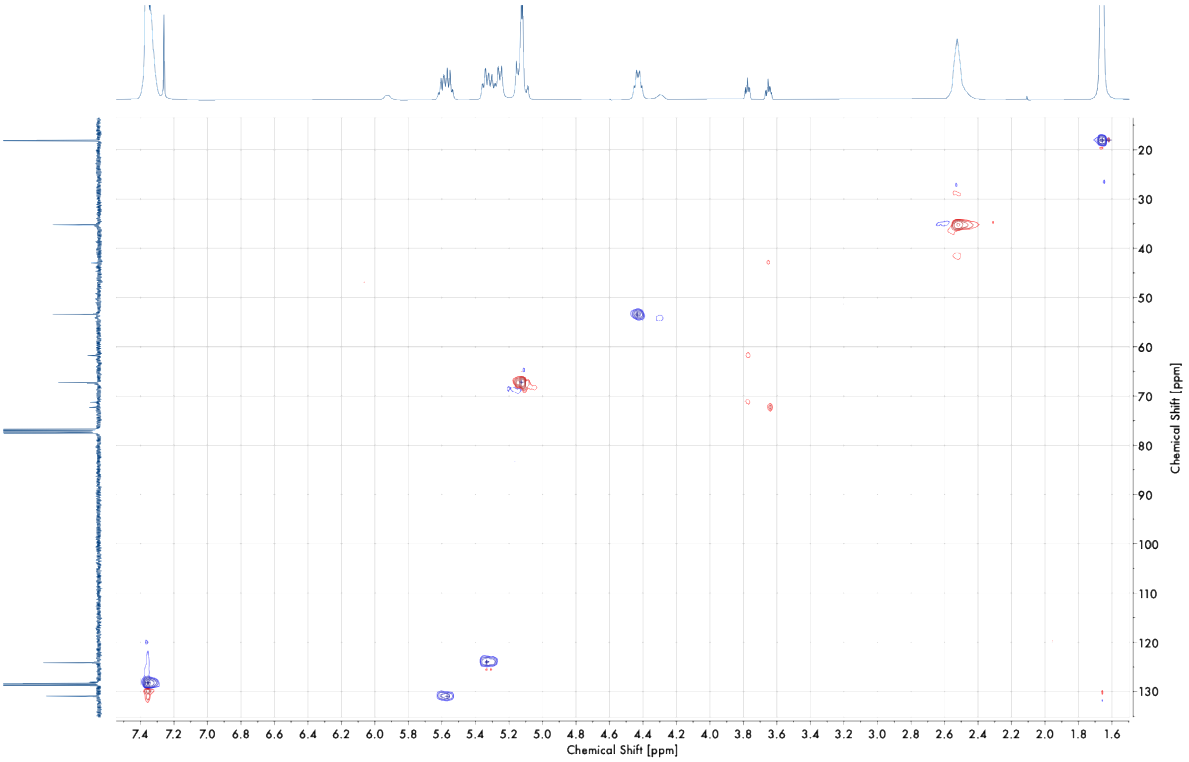

(^{1}H,^{13}C)-HMBC (CDCl$_3$, **3**):

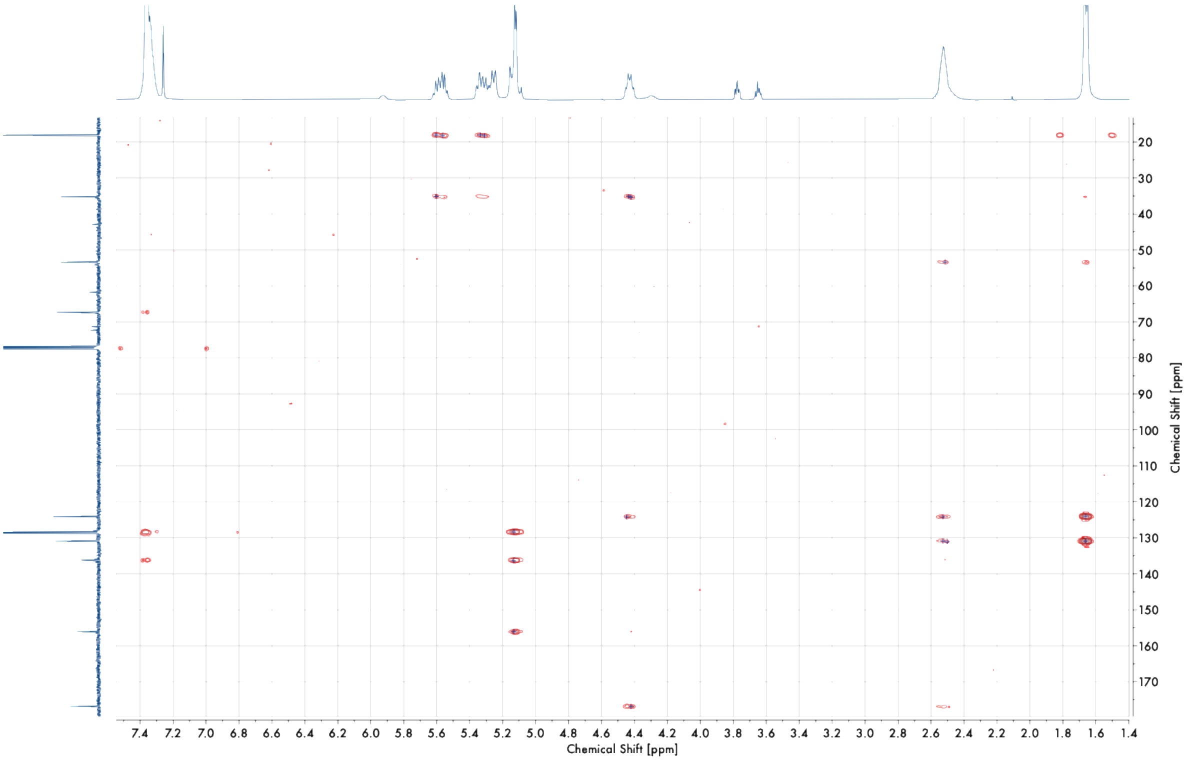

***tert*-Butyl (*S*,*E*)-2-(((benzyloxy)carbonyl)amino)hex-4-enoate [4]**

^{1}H NMR (400 MHz, CDCl$_3$, **4**):

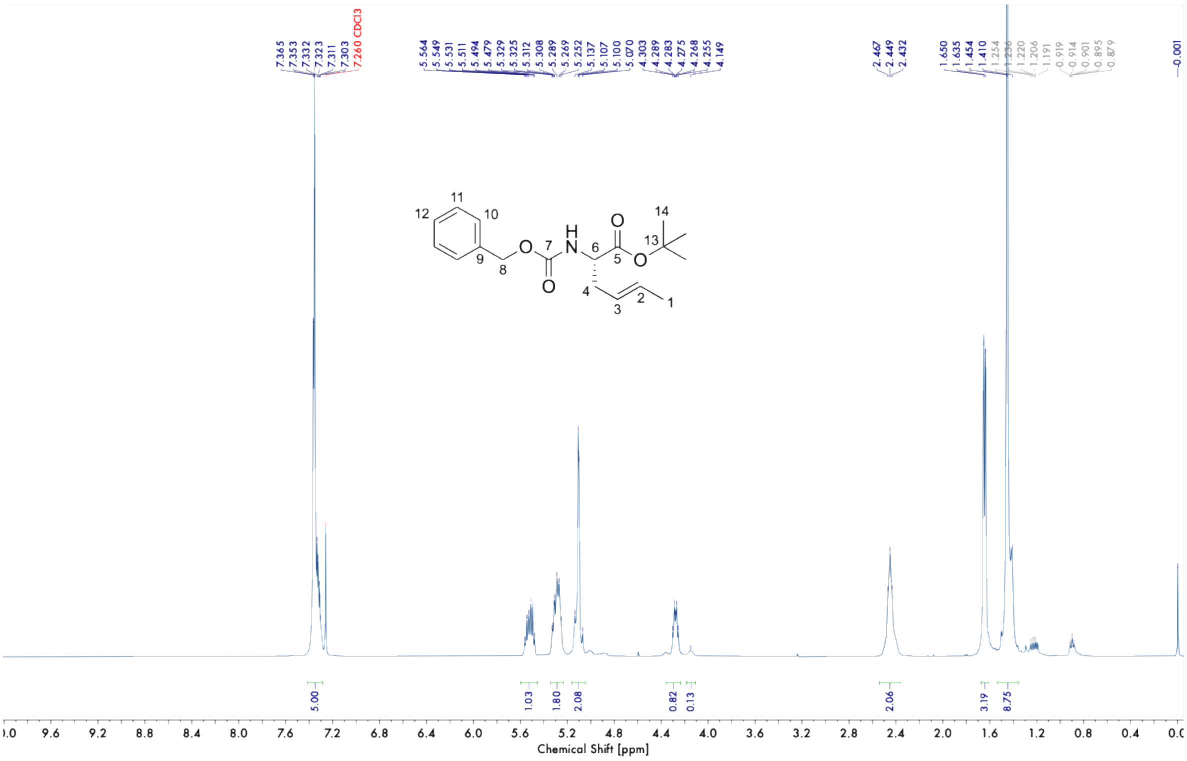

^{13}C NMR (101 MHz, CDCl$_3$, **4**):

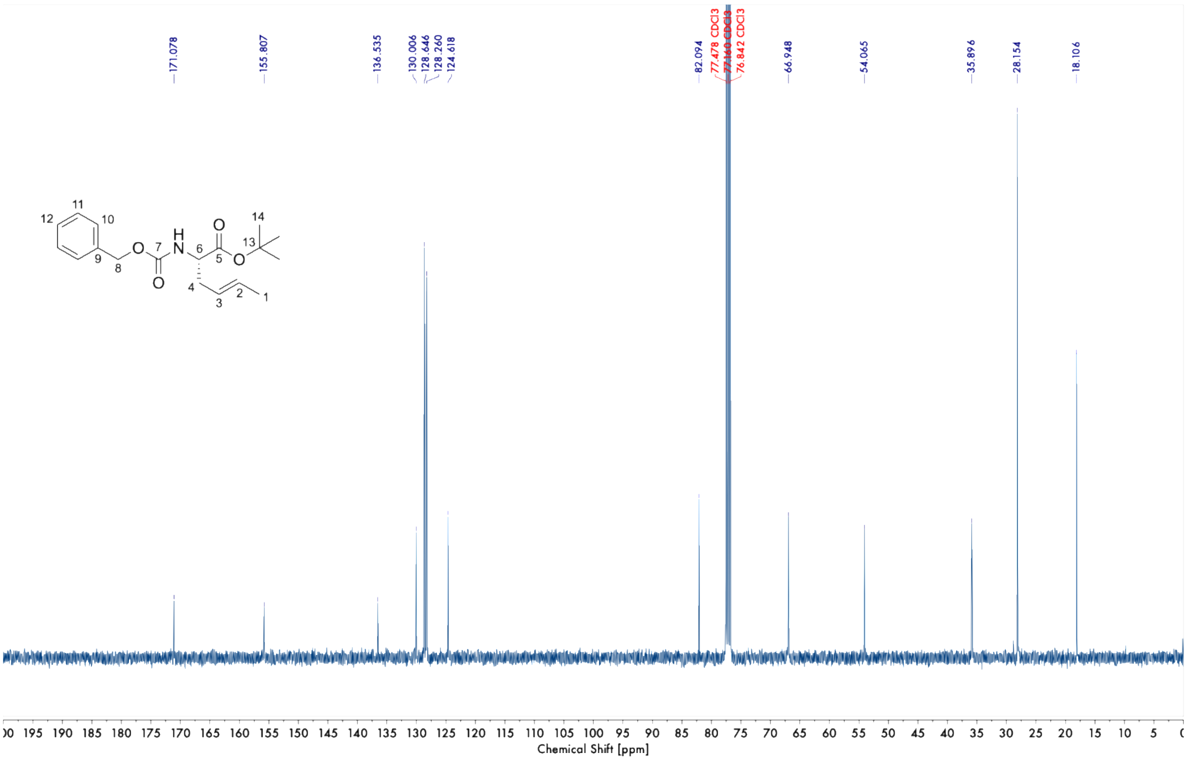

(^{1}H,^{1}H)-COSY (CDCl$_3$, **4**):

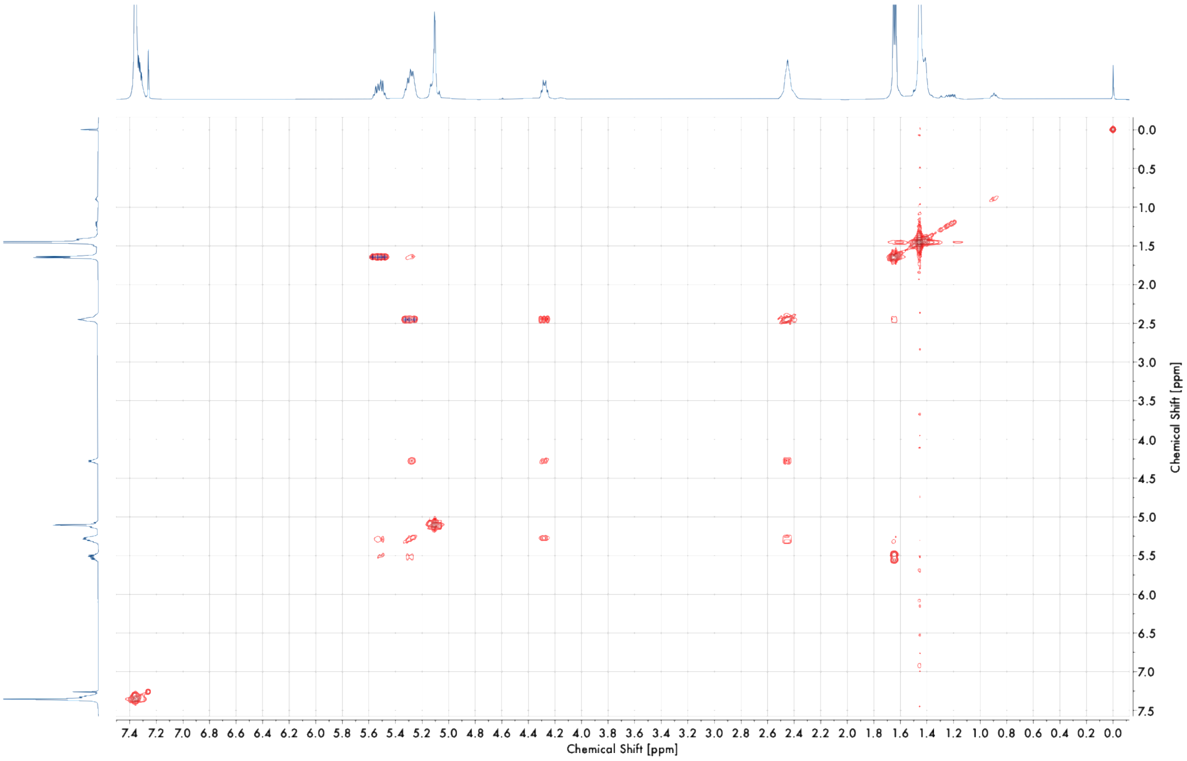

(^{1}H,^{13}C)-HSQC (CDCl$_3$, **4**):

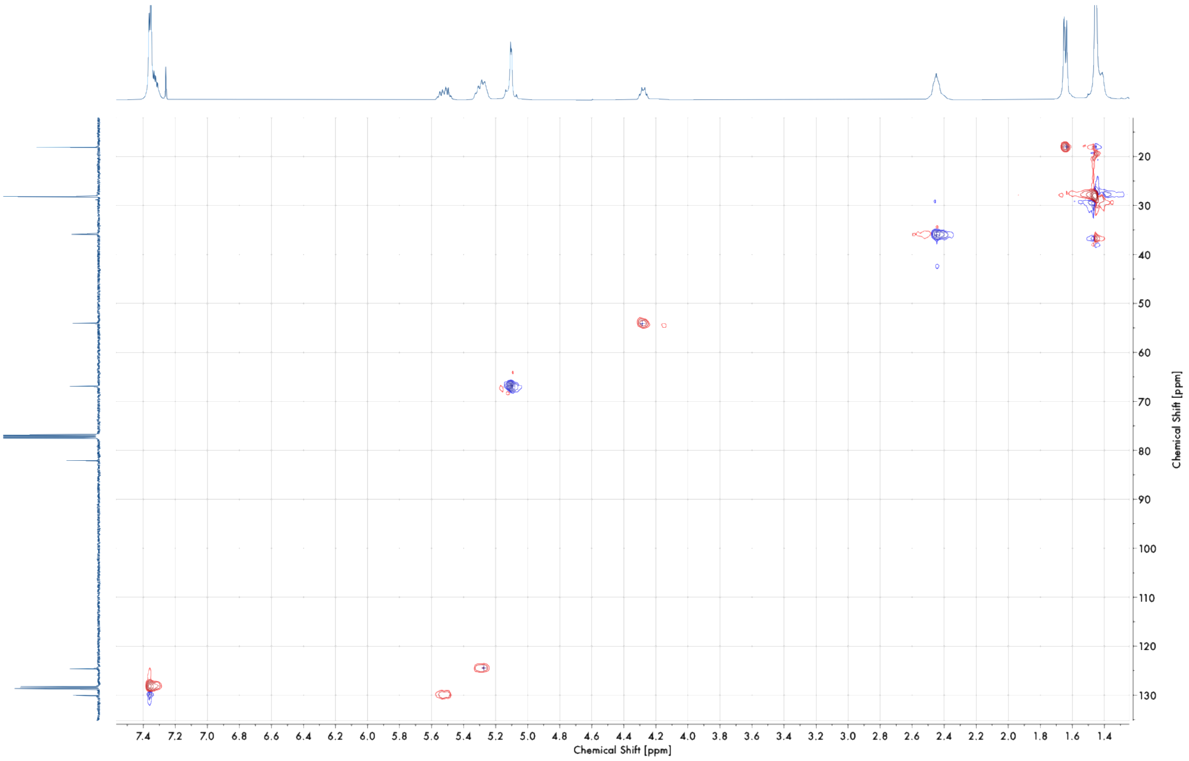

(^{1}H,^{13}C)-HMBC (CDCl$_3$, **4**):

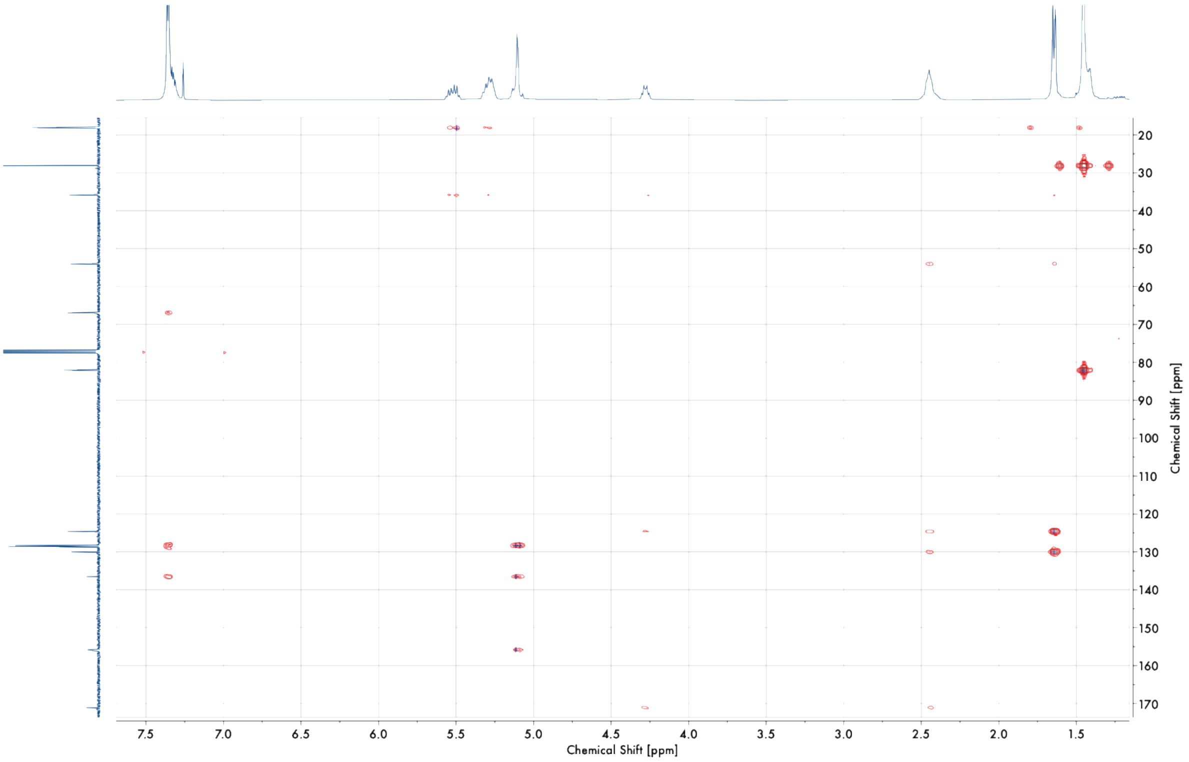

***tert*-butyl (2*S*)-2-[((benzyloxy)carbonyl)amino]-4,5-dihydroxyhexanoate [5]**

^{1}H NMR (500 MHz, DMSO-d_6, 373 K, **5**):

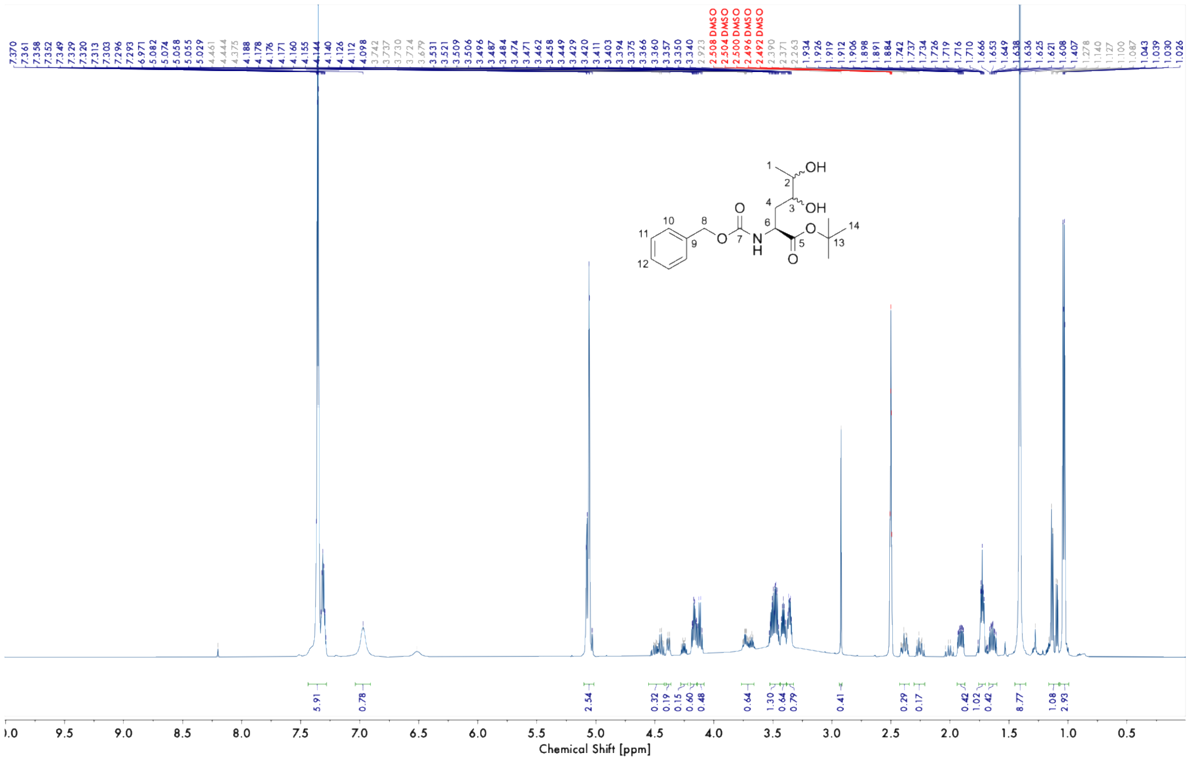

^{13}C NMR (126 MHz, DMSO-d_6, 373 K, **5**):

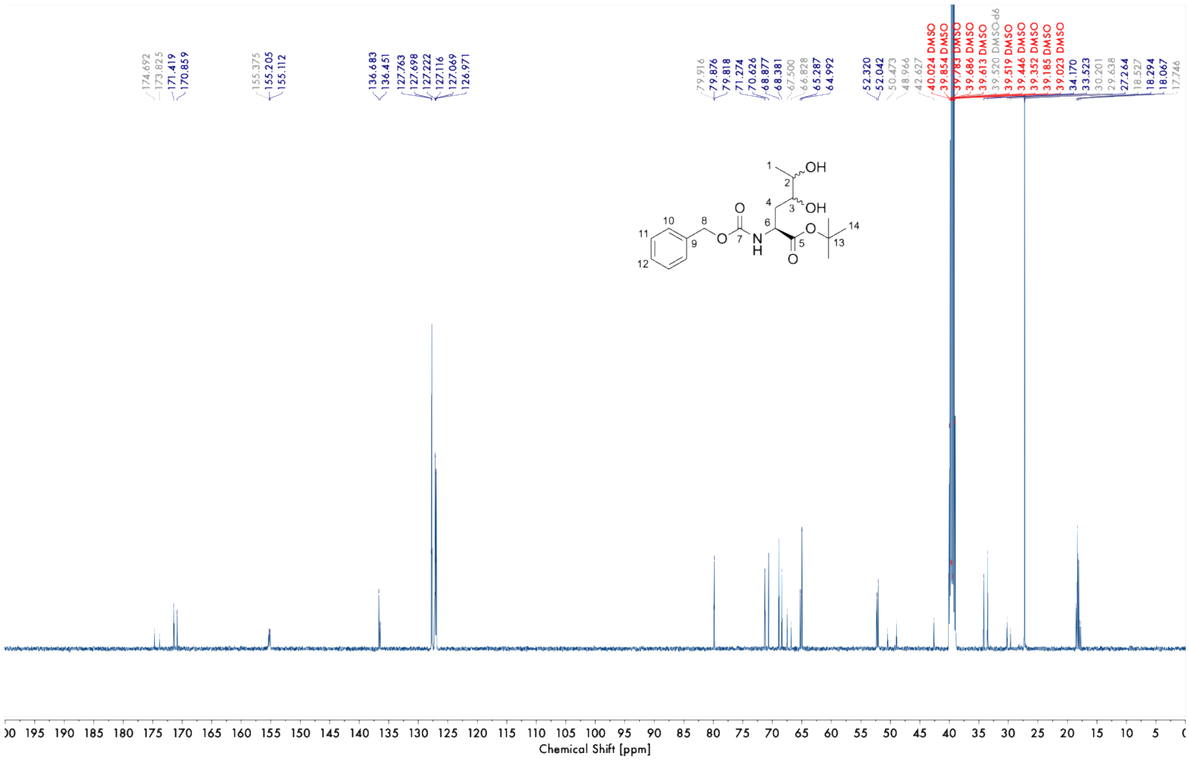

(^{1}H,^{1}H)-COSY (DMSO-d_6, 373 K, **5**):

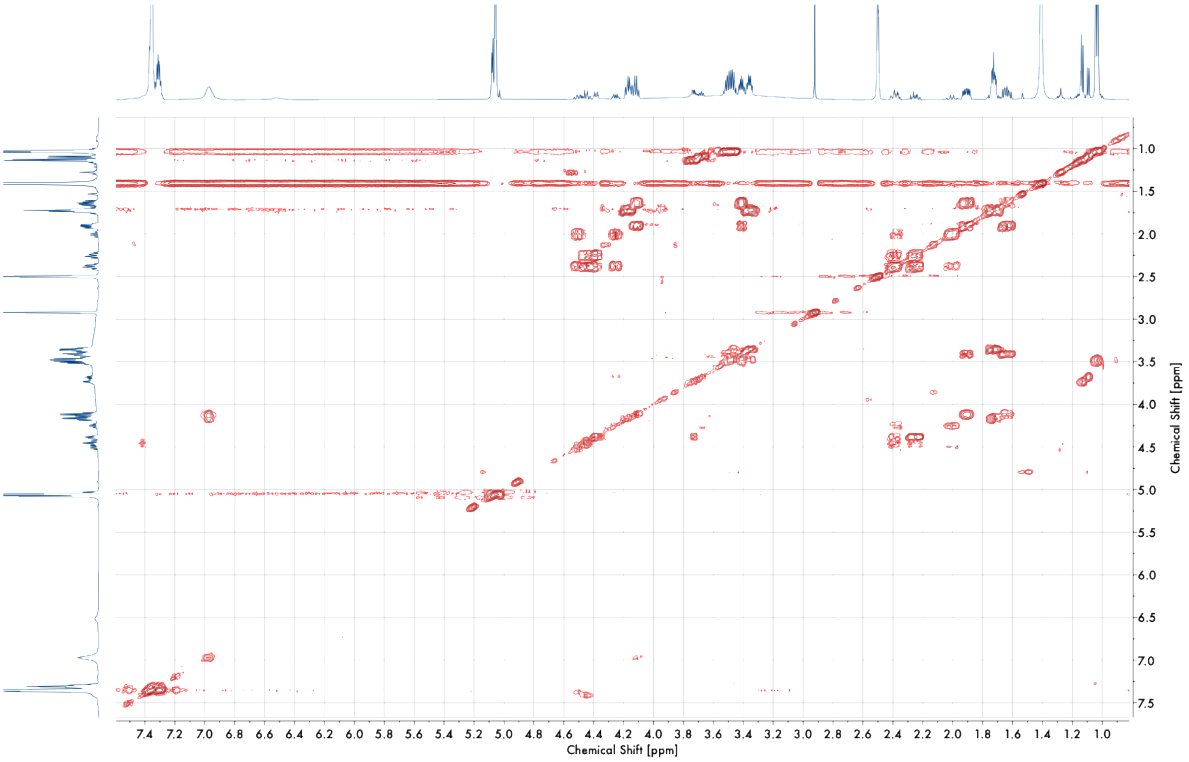

(^{1}H,^{13}C)-HSQC (DMSO-d_6, 373 K, **5**):

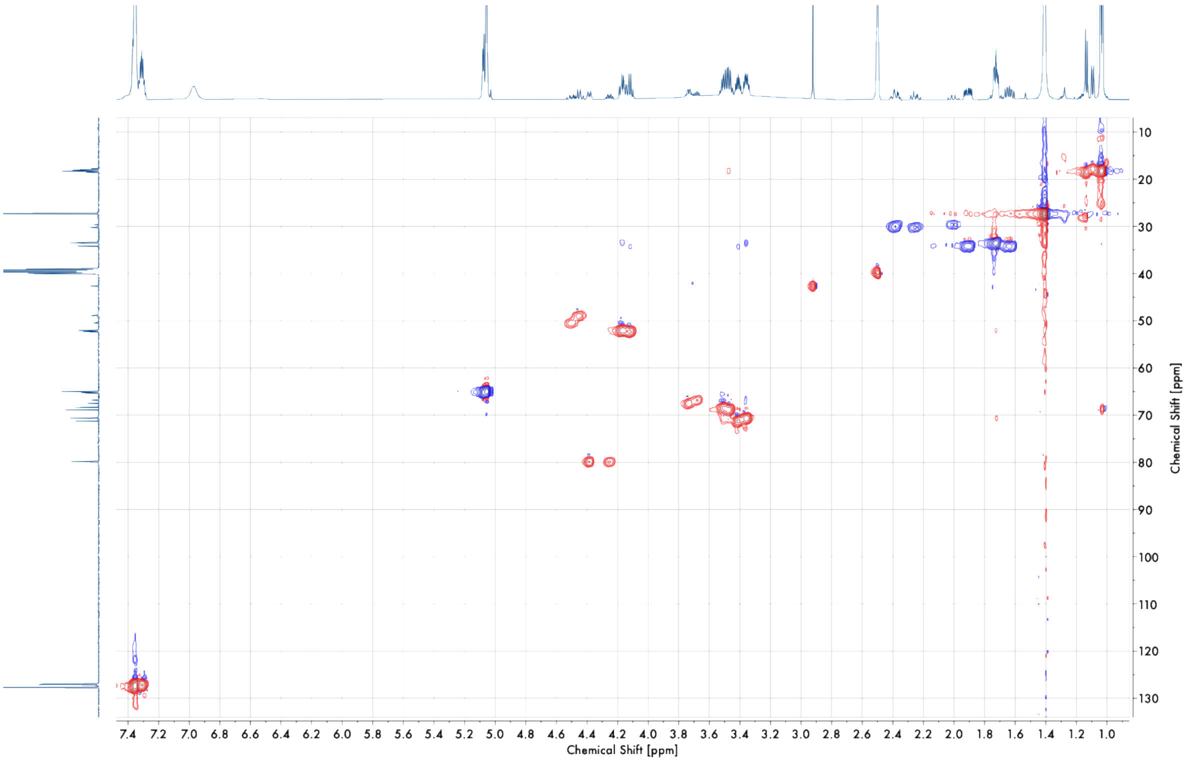

(^{1}H,^{13}C)-HMBC (DMSO-d_6, 373 K, **5**):

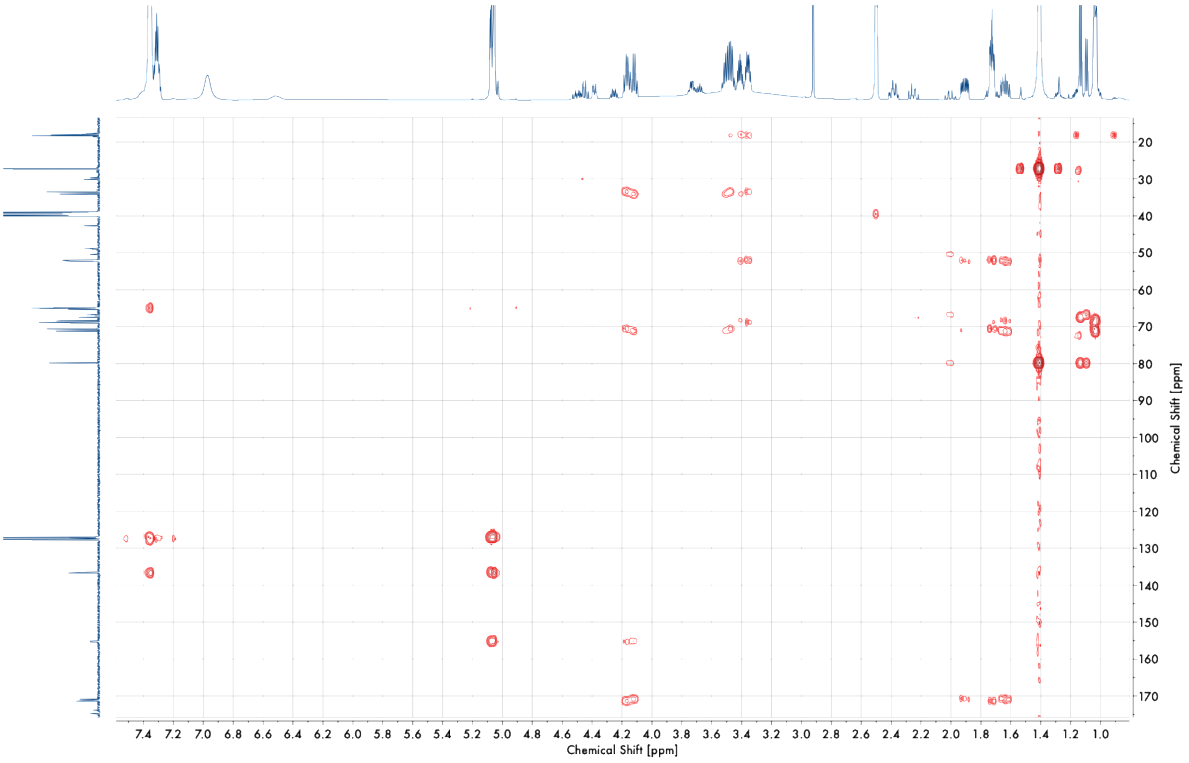

***tert*-butyl (*S*,*E*)-2-(bis((benzyloxy)carbonyl)amino)hex-4-enoate [6]**

^{1}H NMR (500 MHz, $CDCl_3$, **6**):

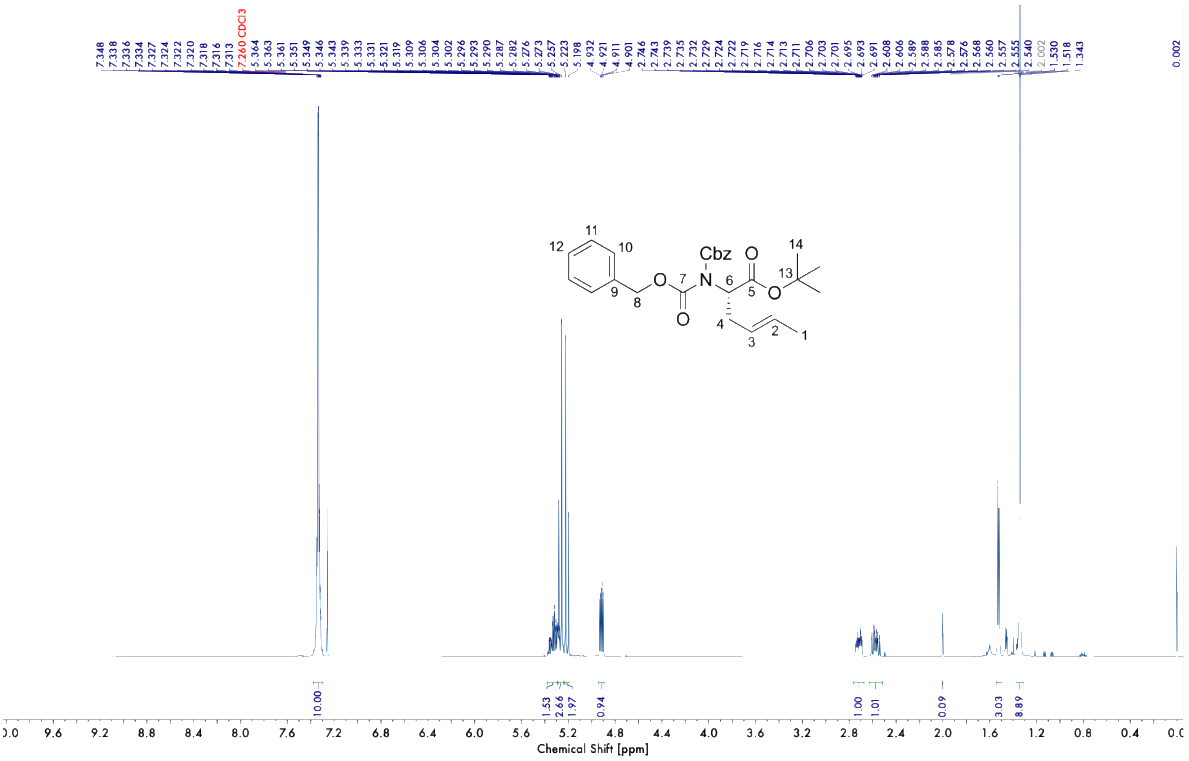

^{13}C NMR (126 MHz, $CDCl_3$, **6**):

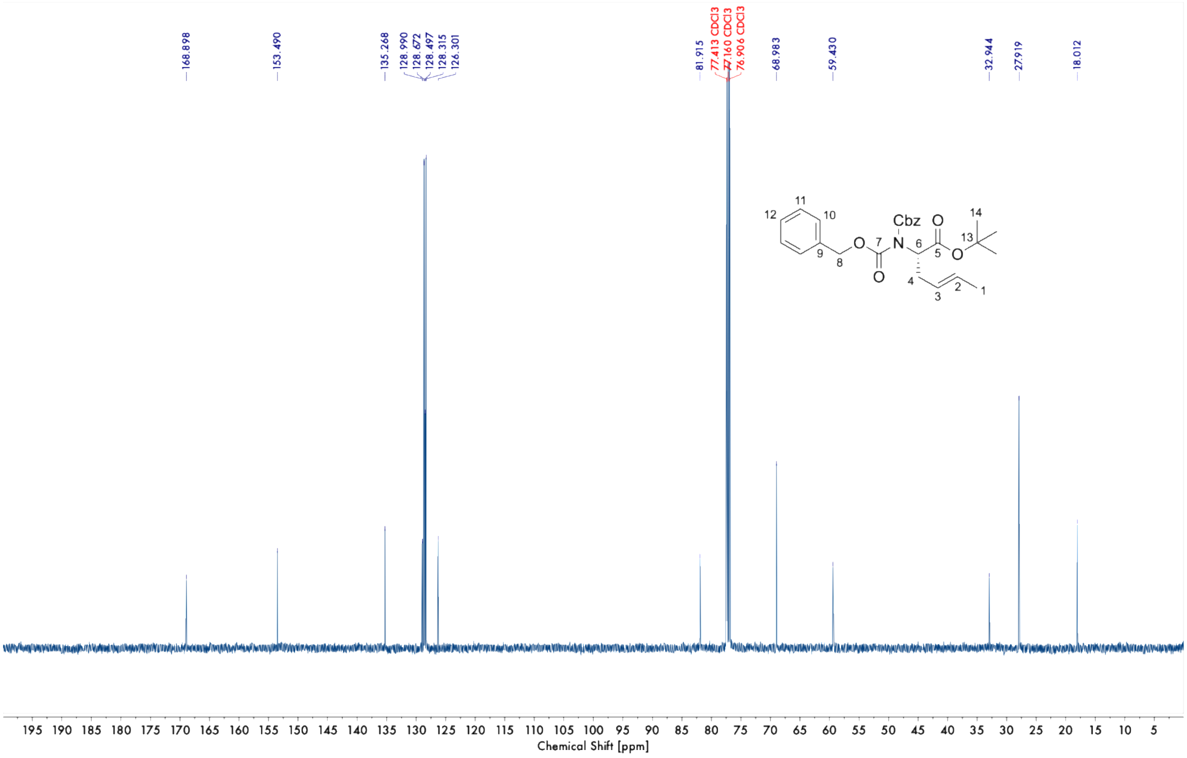

(^{1}H,^{1}H)-COSY ($CDCl_3$, **6**):

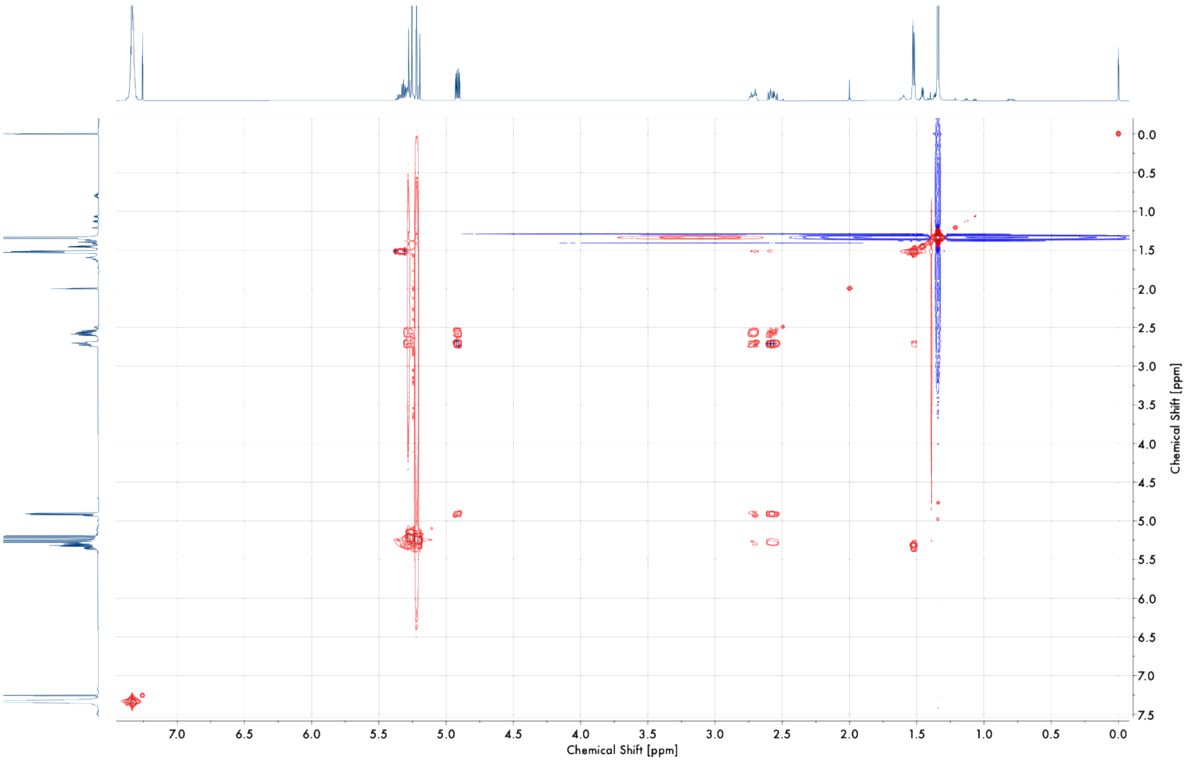

(^{1}H,^{13}C)-HSQC (CDCl$_3$, **6**):

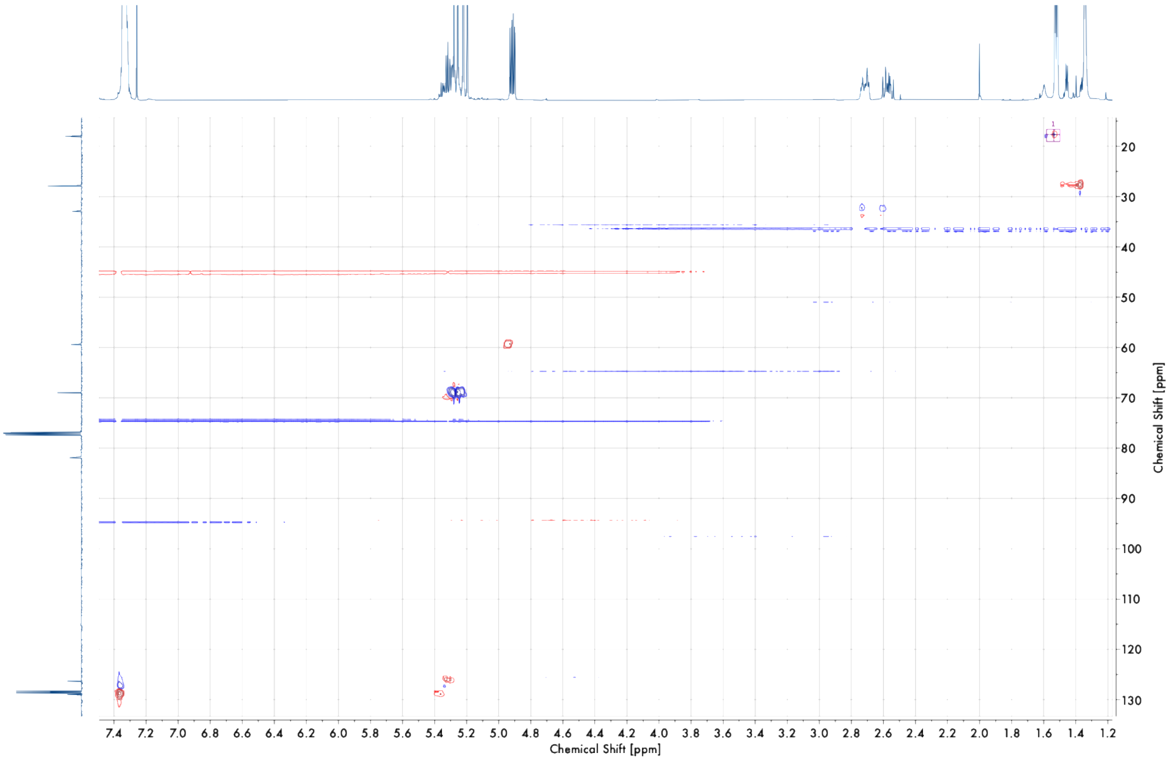

(^{1}H,^{13}C)-HMBC (CDCl$_3$, **6**):

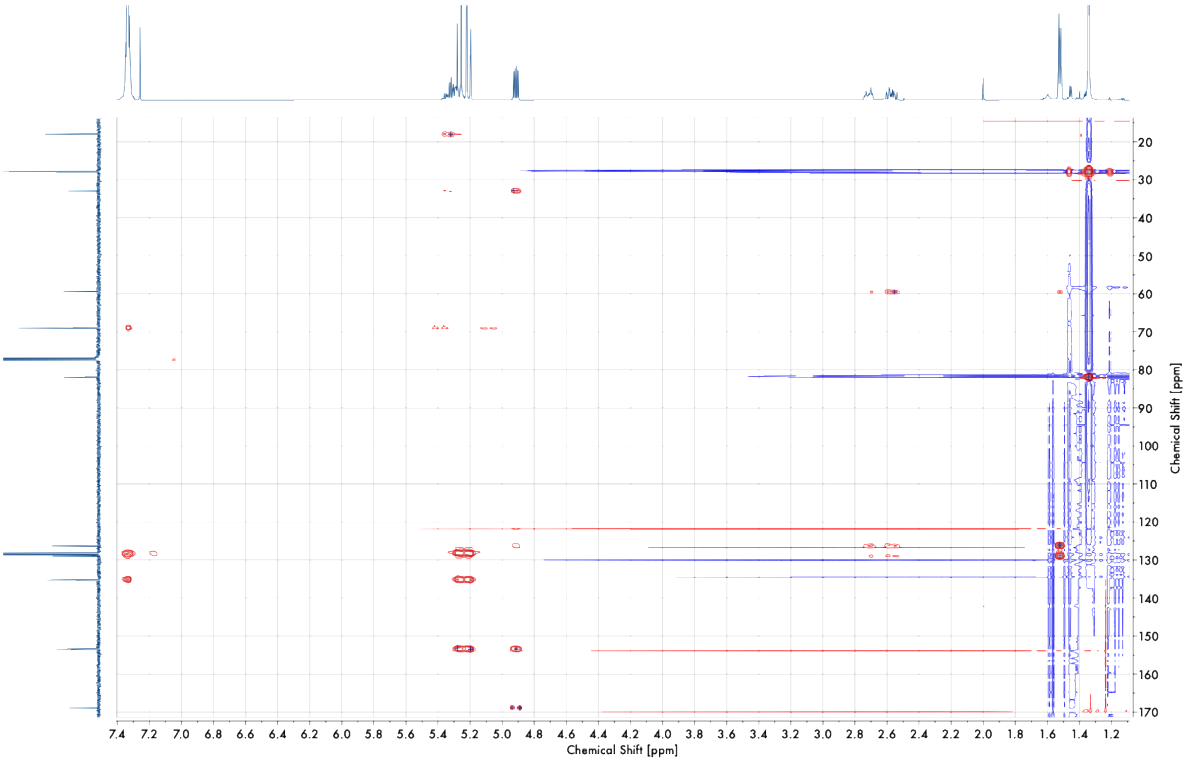

3-benzyl 4-(*tert*-butyl) (4*S*,6*R*)-6-((*R*)-1-hydroxyethyl)-2-oxo-1,3-oxazi-nane-3,4-dicarboxylate [7]

^{1}H NMR (400 MHz, $CDCl_3$, **7**):

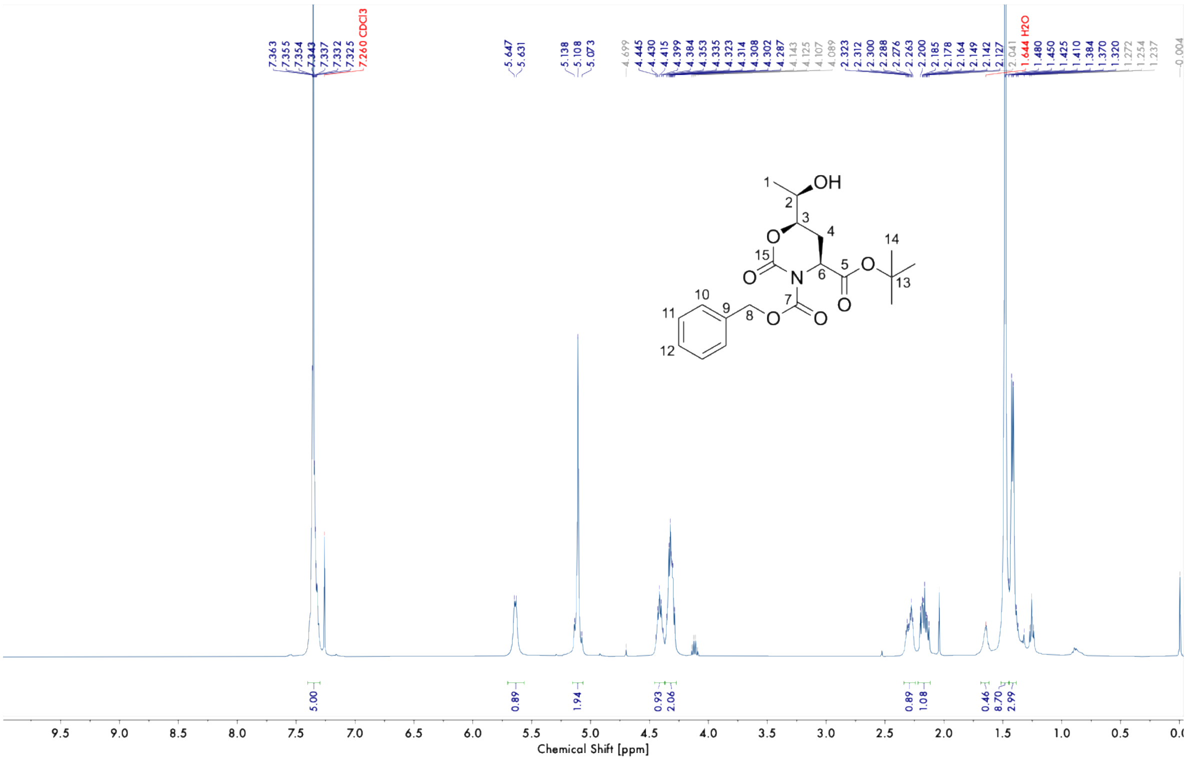

^{13}C NMR (101 MHz, $CDCl_3$, **7**):

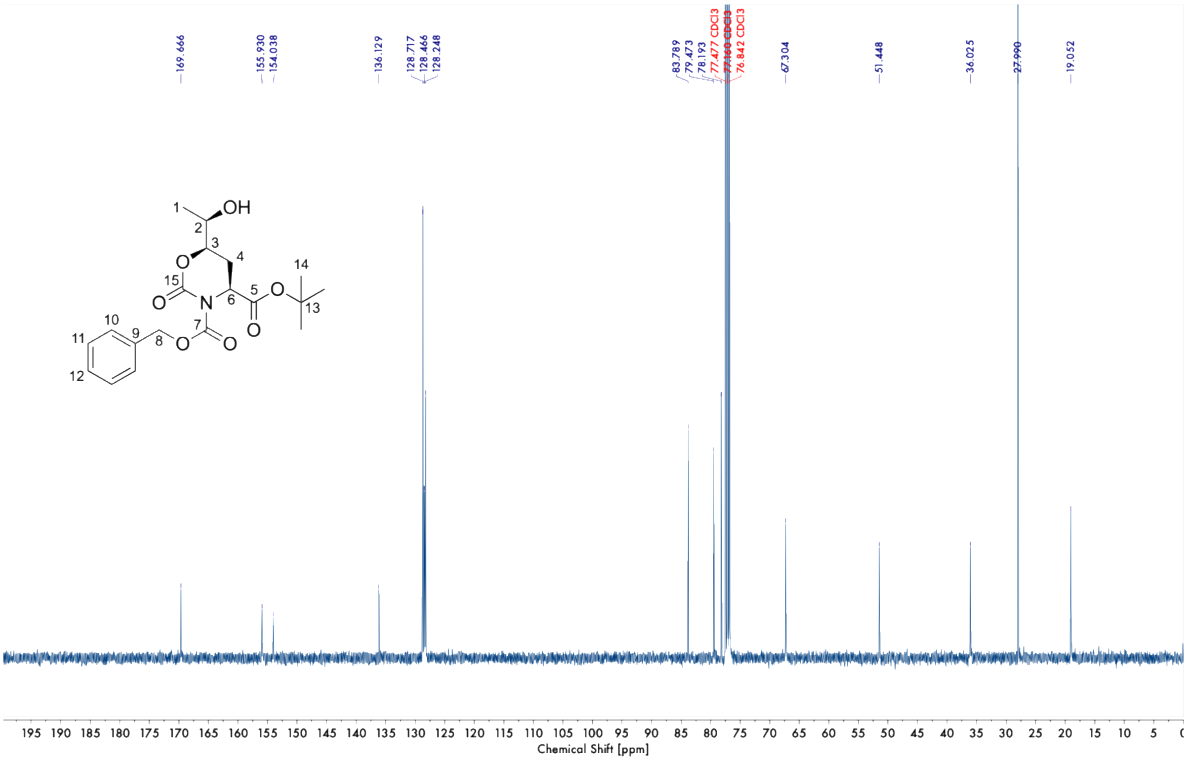

(^{1}H,^{1}H)-COSY (CDCl$_3$, **7**):

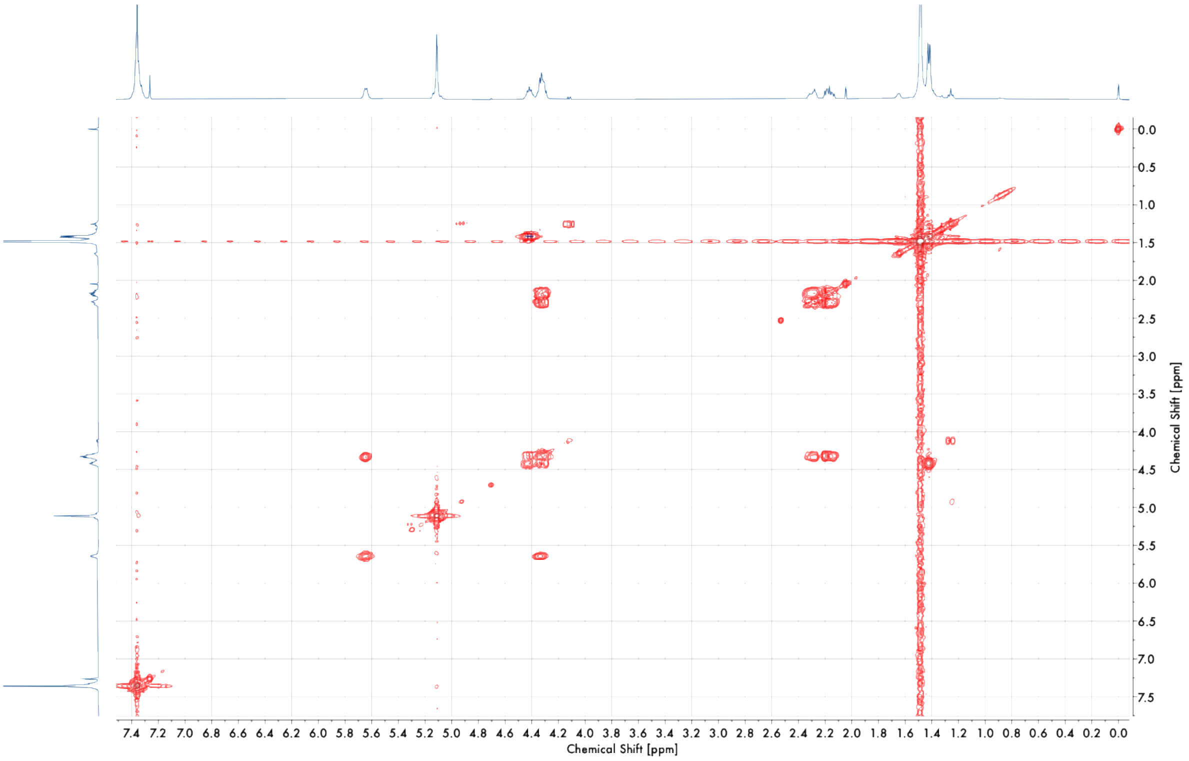

(^{1}H,^{13}C)-HSQC (CDCl$_3$, **7**):

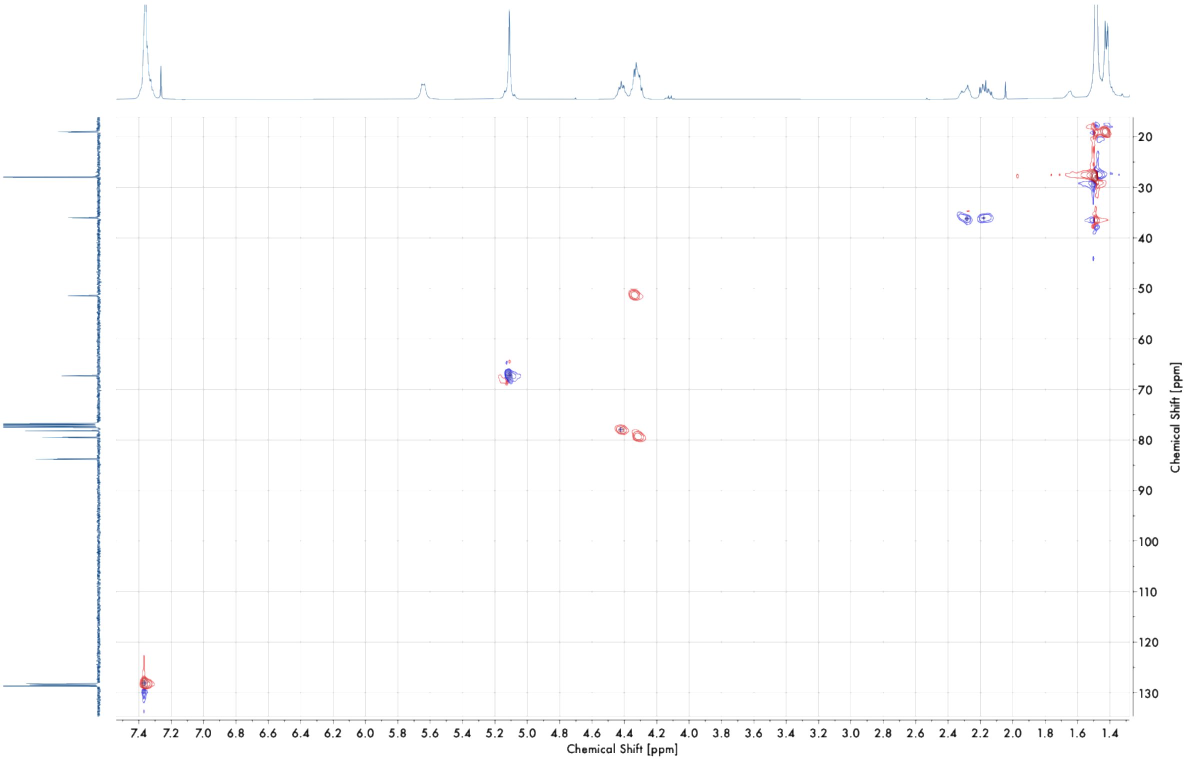

(^{1}H,^{13}C)-HMBC (CDCl$_3$, **7**):

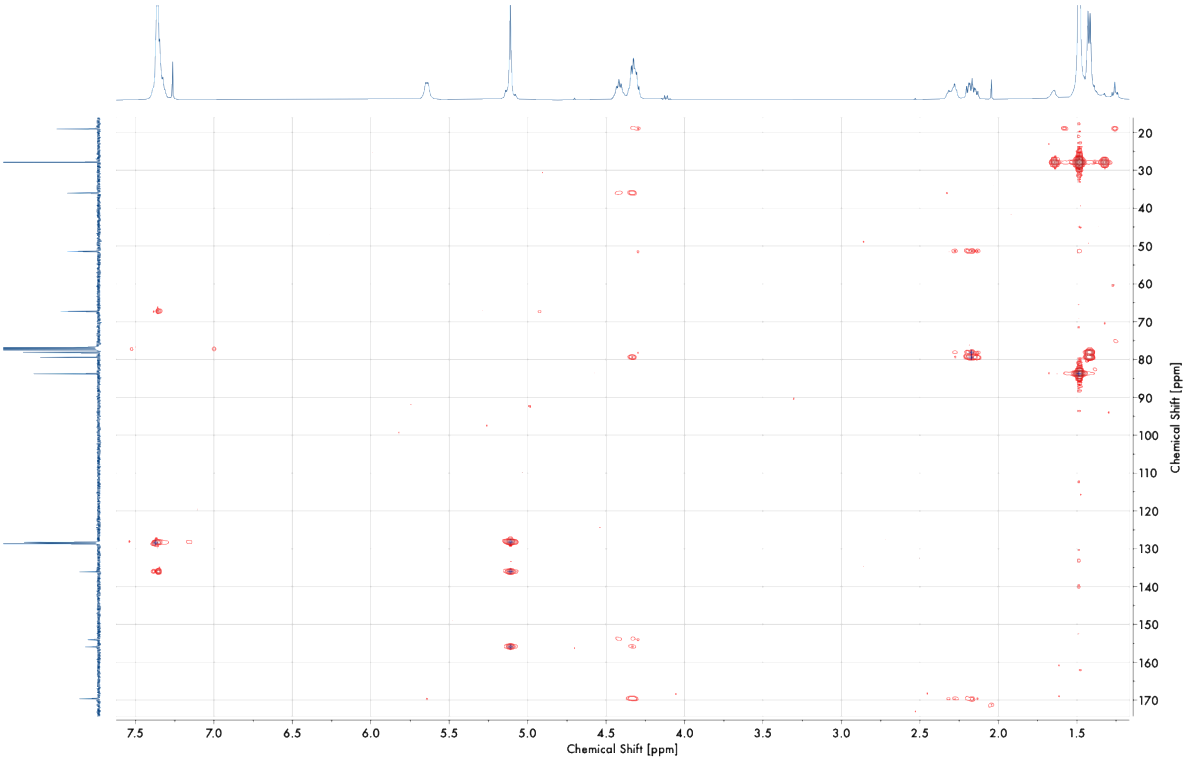

(S)-But-3-yn-2-yl-(*tert*-butoxycarbonyl)-glycinat [SI-1]

^{1}H NMR (500 MHz, CDCl$_3$, **SI-1**):

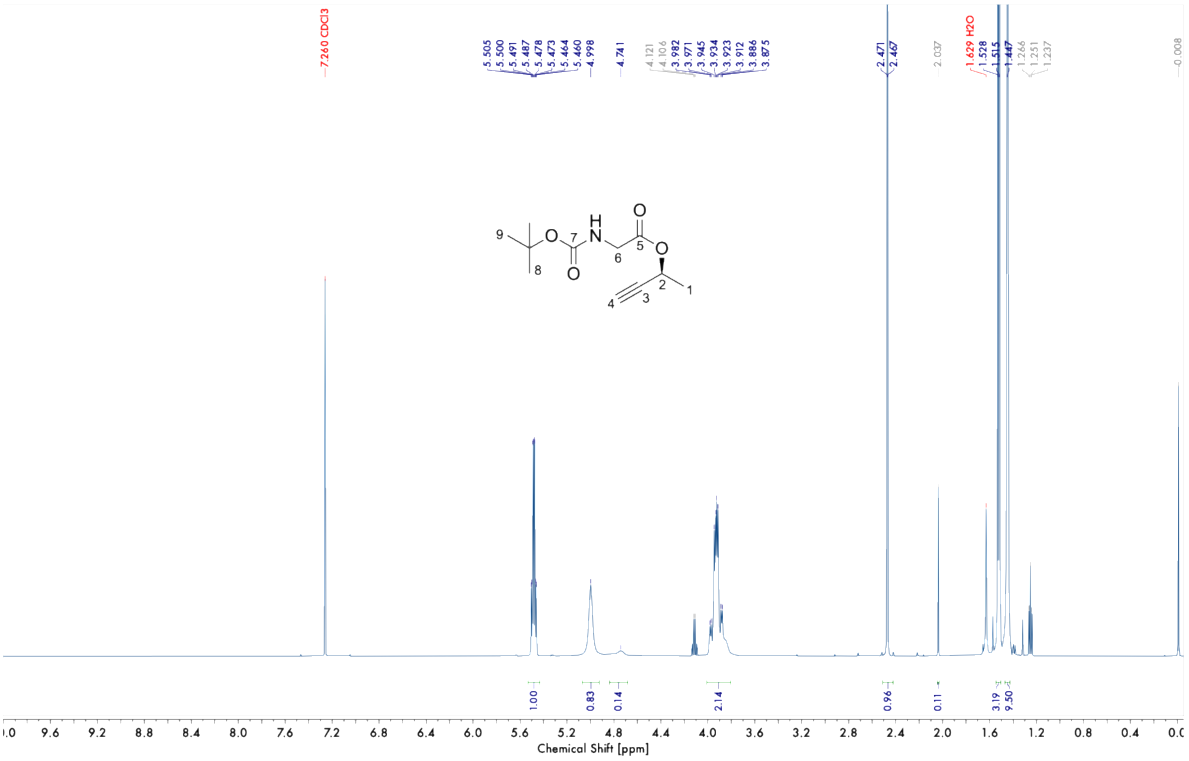

^{13}C NMR (126 MHz, $CDCl_3$, **SI-1**):

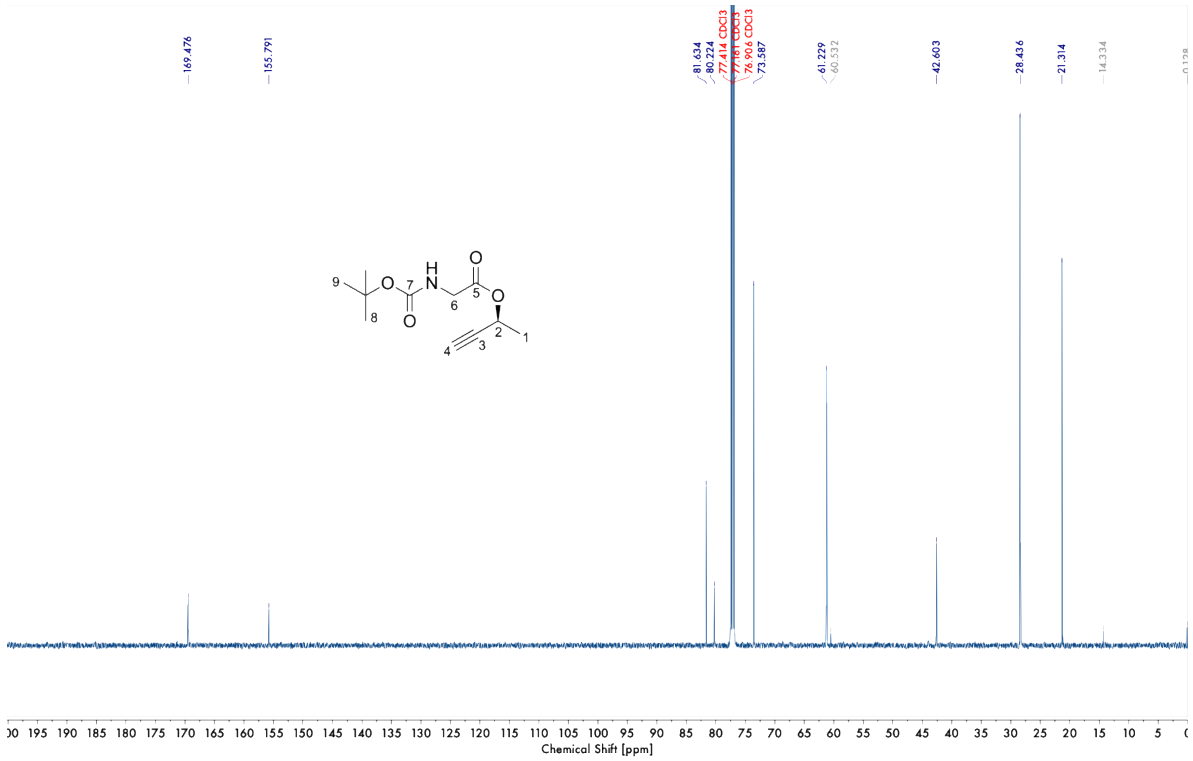

(^{1}H,^{1}H)-COSY ($CDCl_3$, **SI-1**):

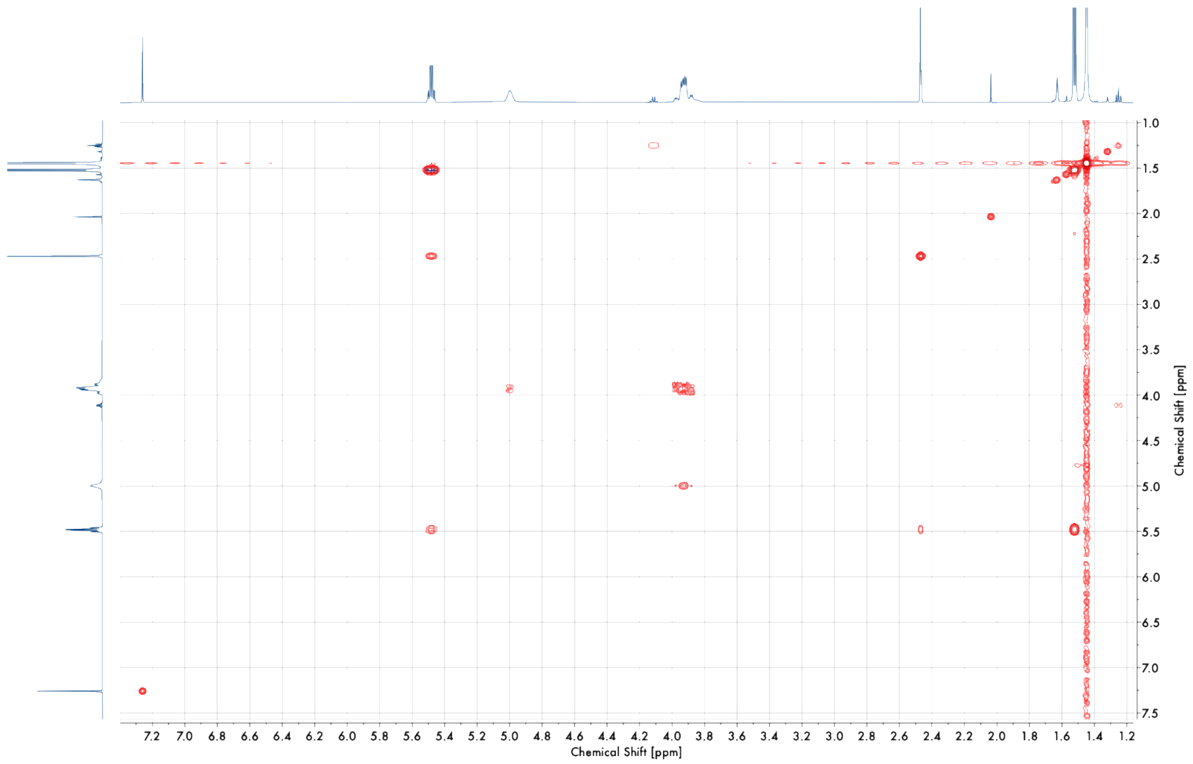

(^1H,^13C)-HSQC (CDCl$_3$, **SI-1**):

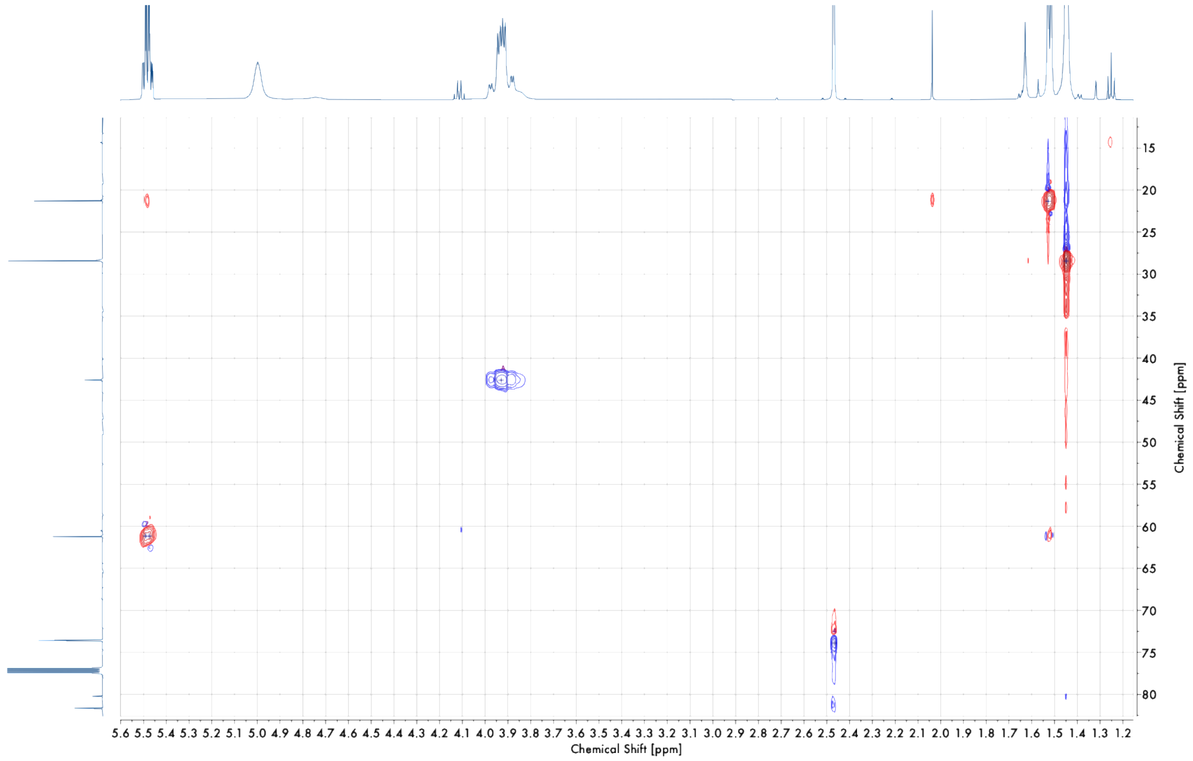

(^1H,^13C)-HMBC (CDCl$_3$, **SI-1**):

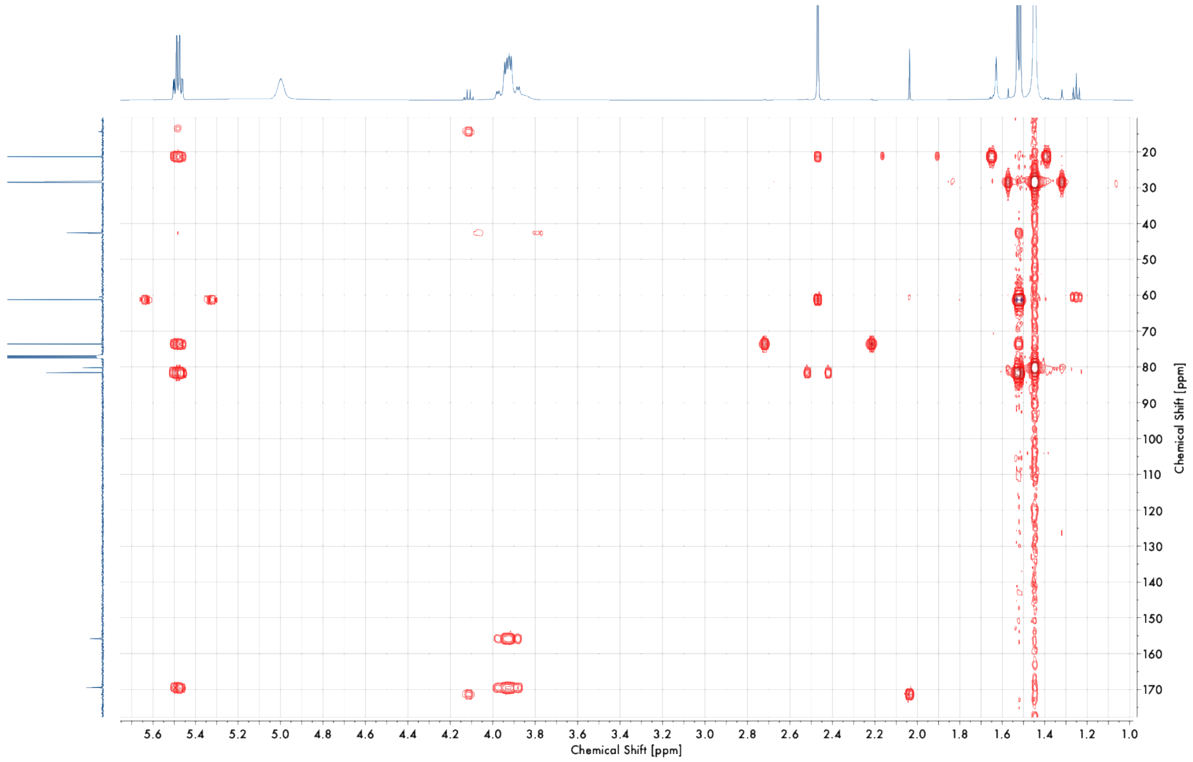

(*S*)-But-3-en-2-yl-(*tert*-butoxycarbonyl)glycinate [8]

^{1}H NMR (500 MHz, $CDCl_3$, **8**):

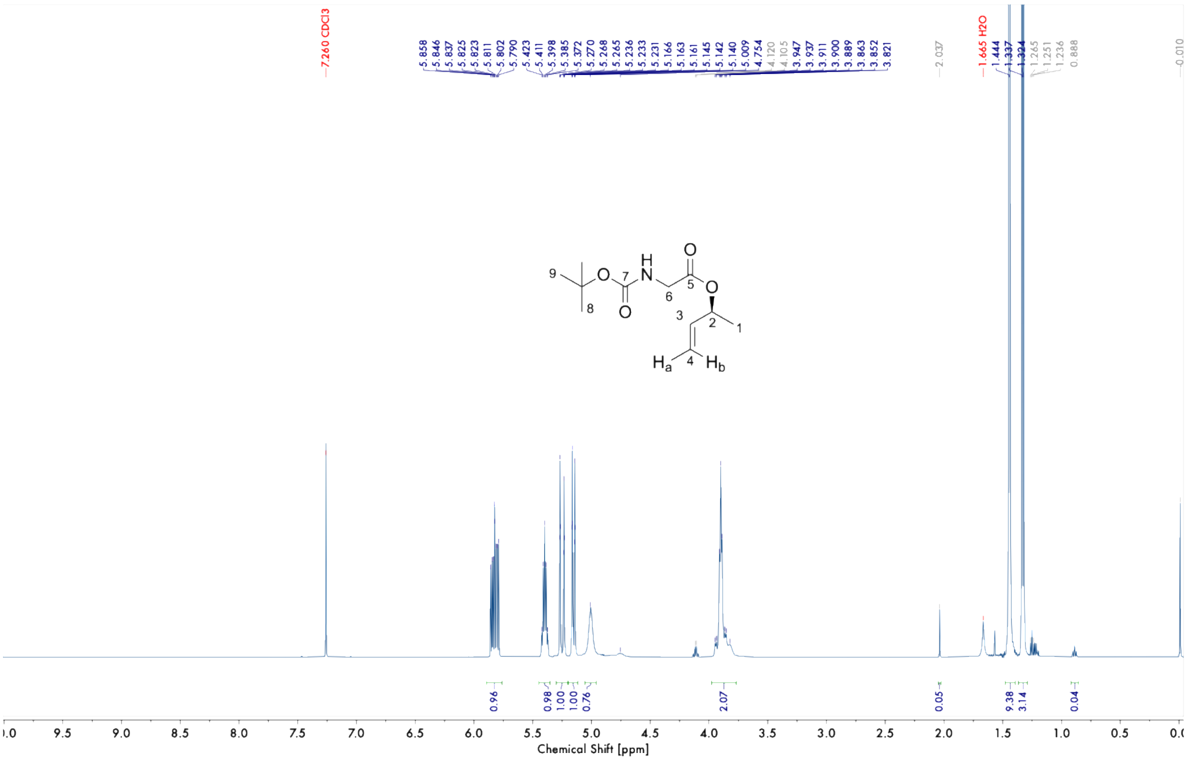

^{13}C NMR (126 MHz, $CDCl_3$, **8**):

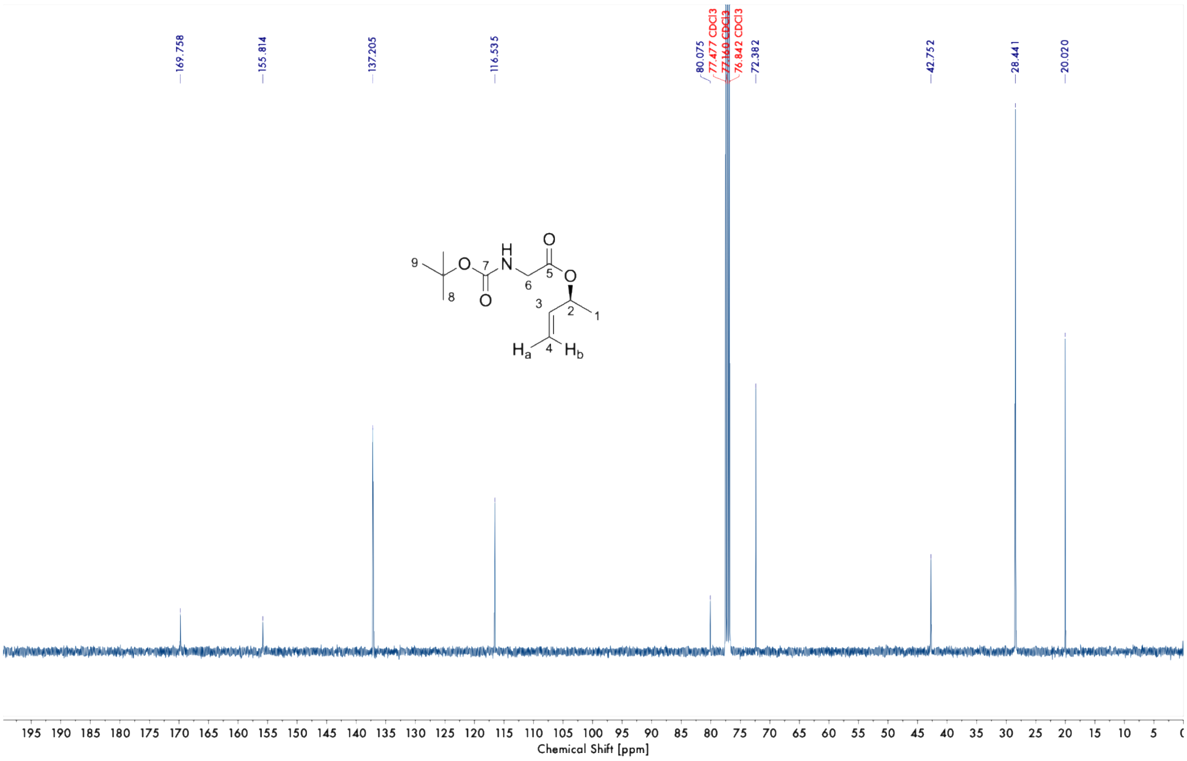

(^{1}H,^{1}H)-COSY (CDCl$_3$, **8**):

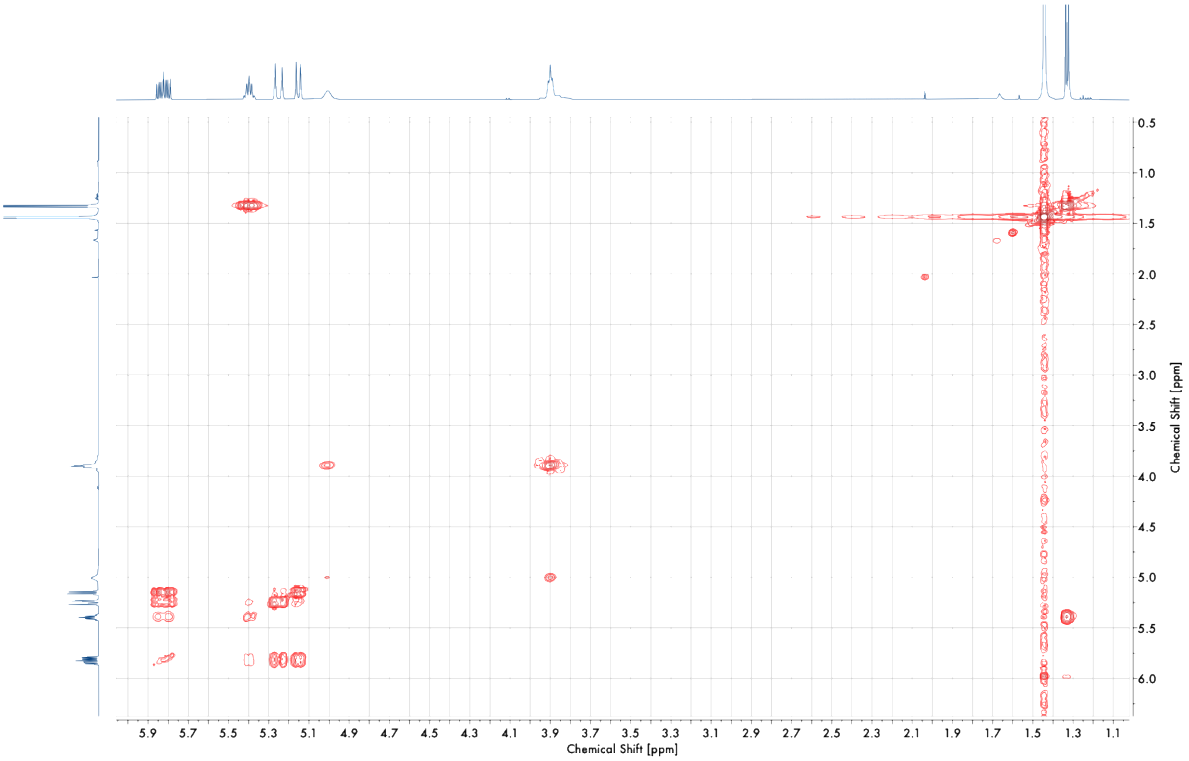

(^{1}H,^{13}C)-HSQC (CDCl$_3$, **8**):

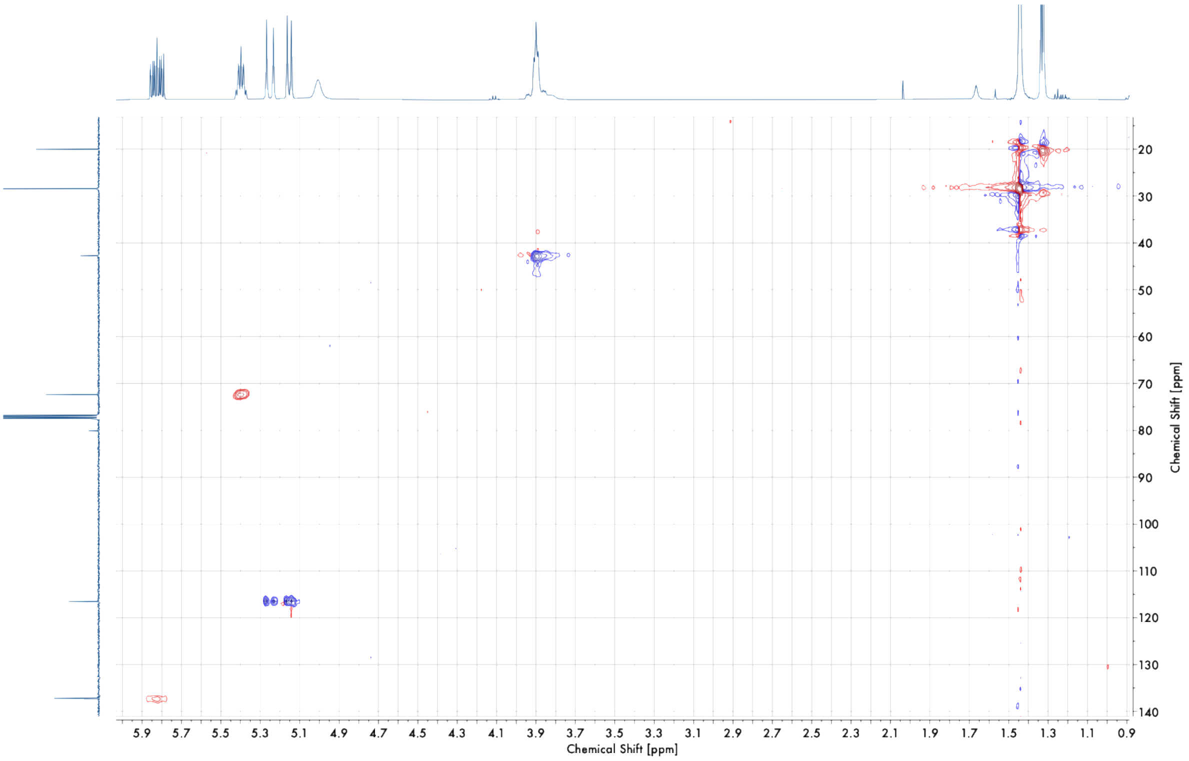

(^{1}H,^{13}C)-HMBC (CDCl$_3$, **8**):

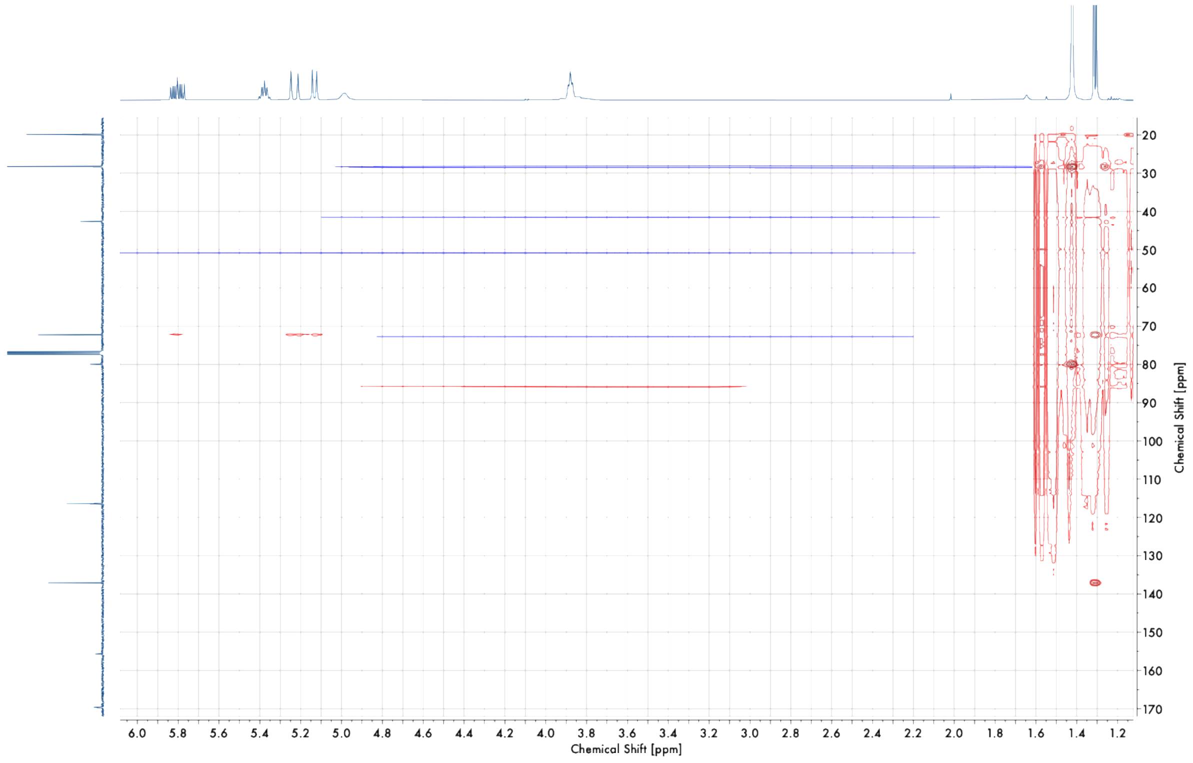

Benzyl (*S*,*E*)-2-((*tert*-butoxycarbonyl)amino)hex-4-enoate [9]

^{1}H NMR (500 MHz, CDCl$_3$, **9**):

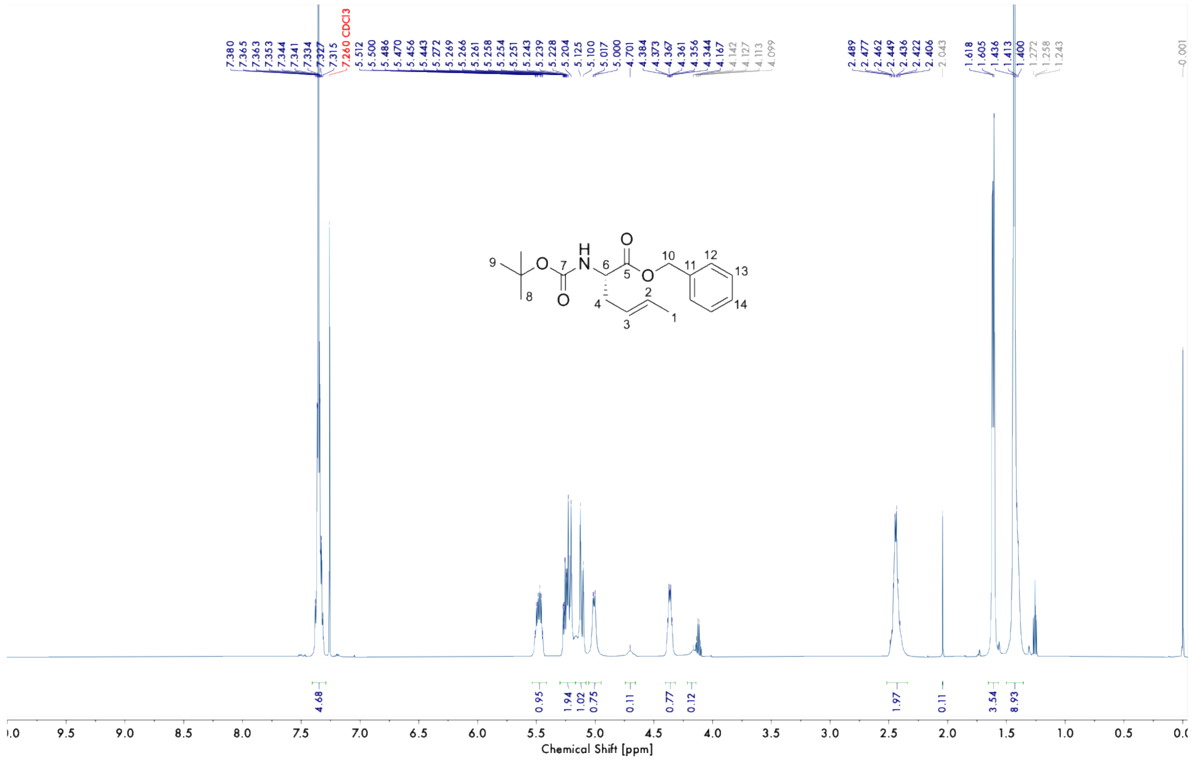

^{13}C NMR (126 MHz, CDCl$_3$, **9**):

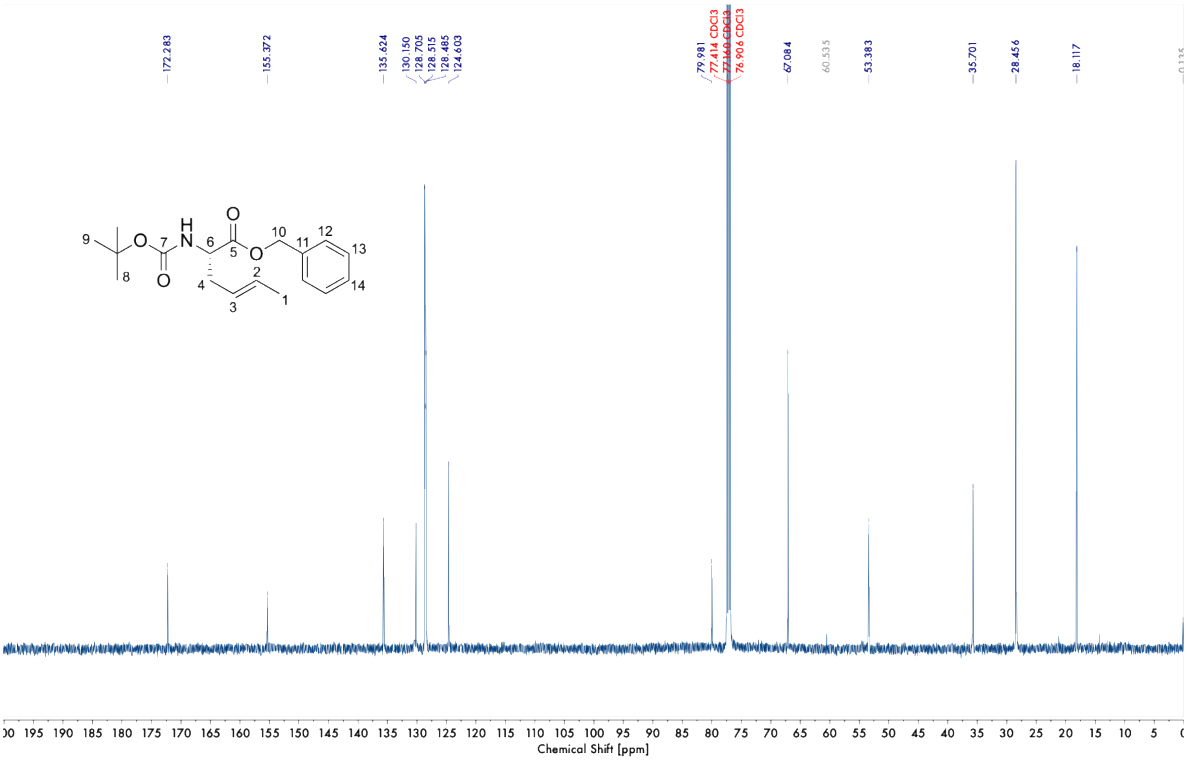

(**^{1}H,^{1}H)-COSY** (CDCl$_3$, **9**):

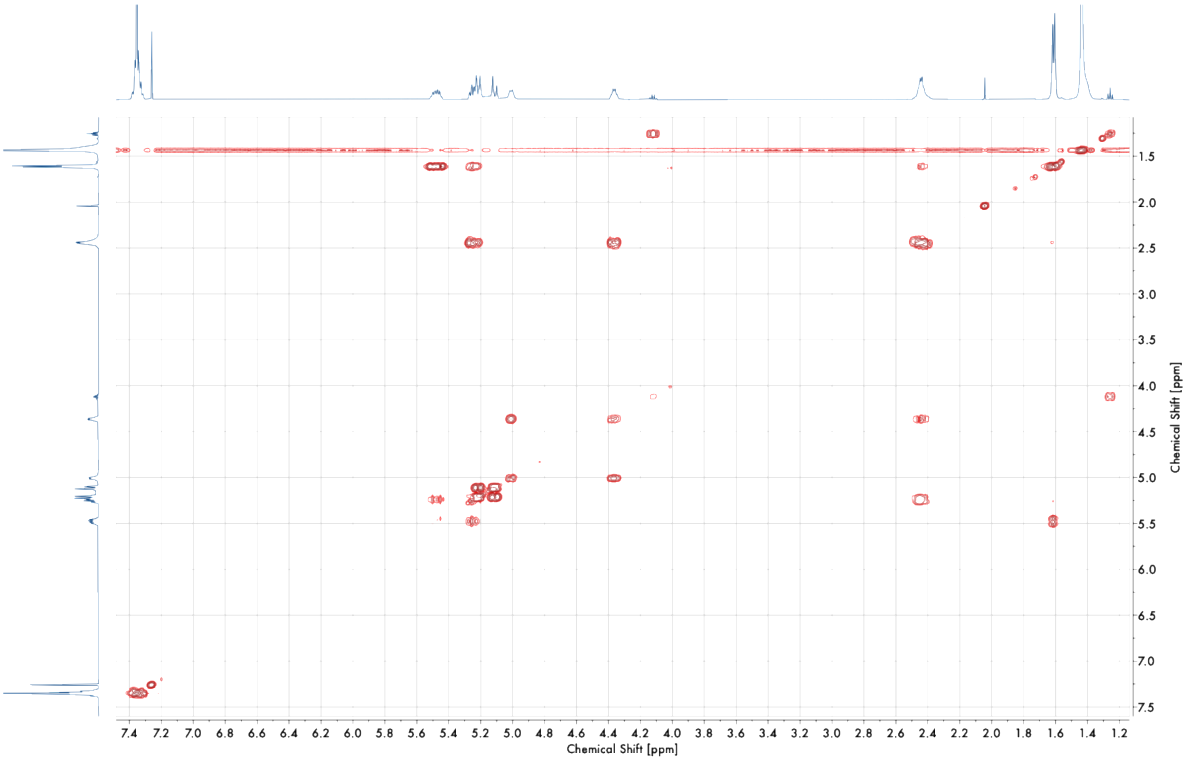

(^{1}H,^{13}C)-**HSQC** (CDCl$_3$, **9**):

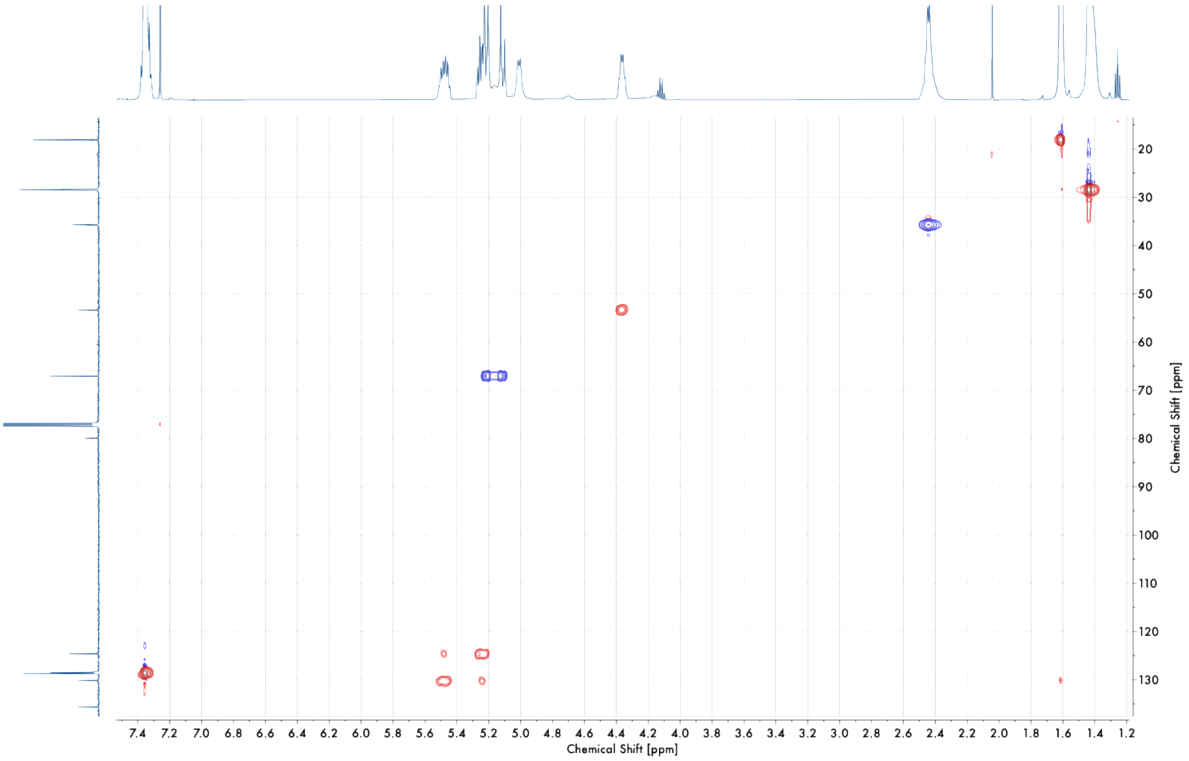

(^{1}H,^{13}C)-**HMBC** (CDCl$_3$, **9**):

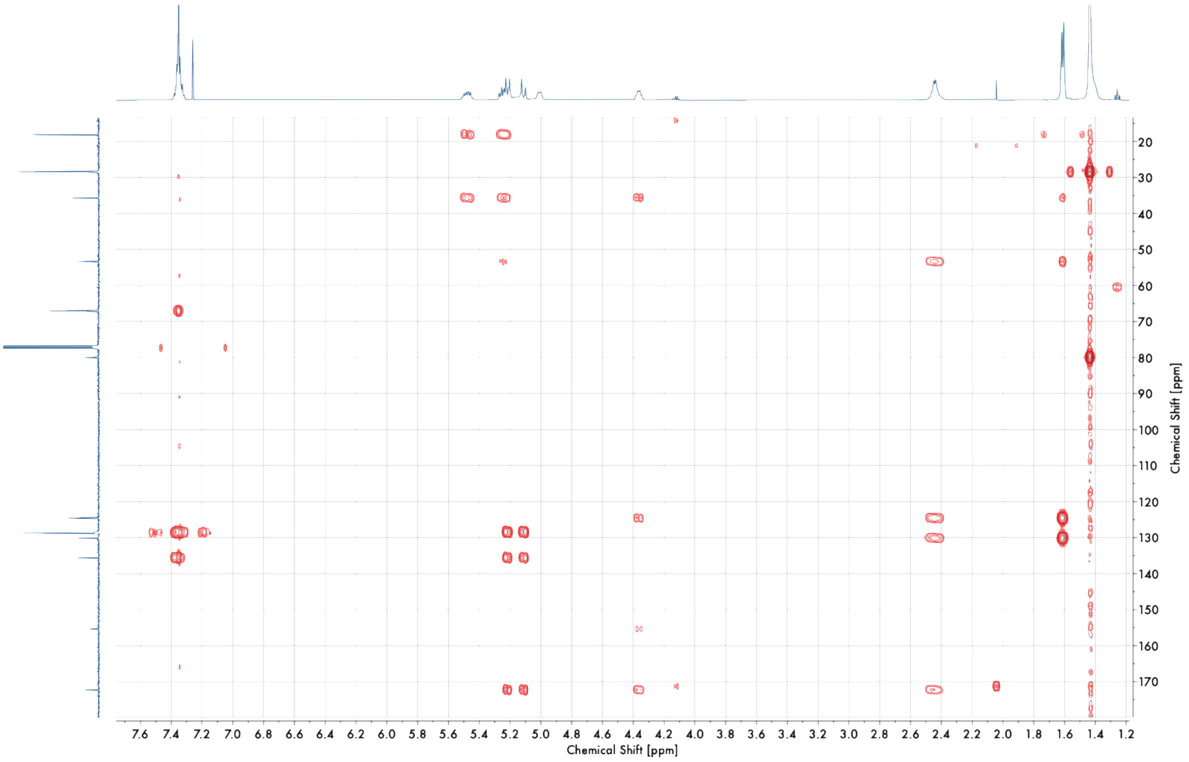

Benzyl (*S*,*E*)-2-(bis(*tert*-butoxycarbonyl)amino)hex-4-enoate [10]

^{1}H NMR (500 MHz, $CDCl_3$, **10**):

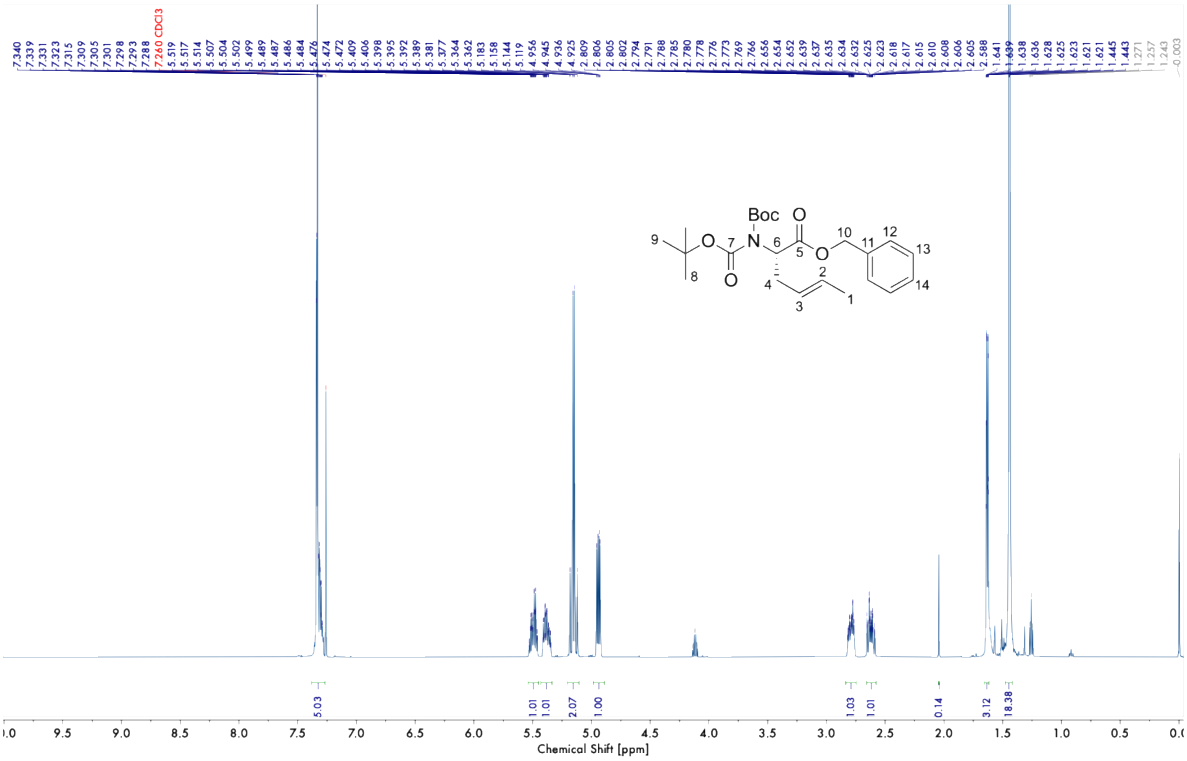

^{13}C NMR (126 MHz, $CDCl_3$, **10**):

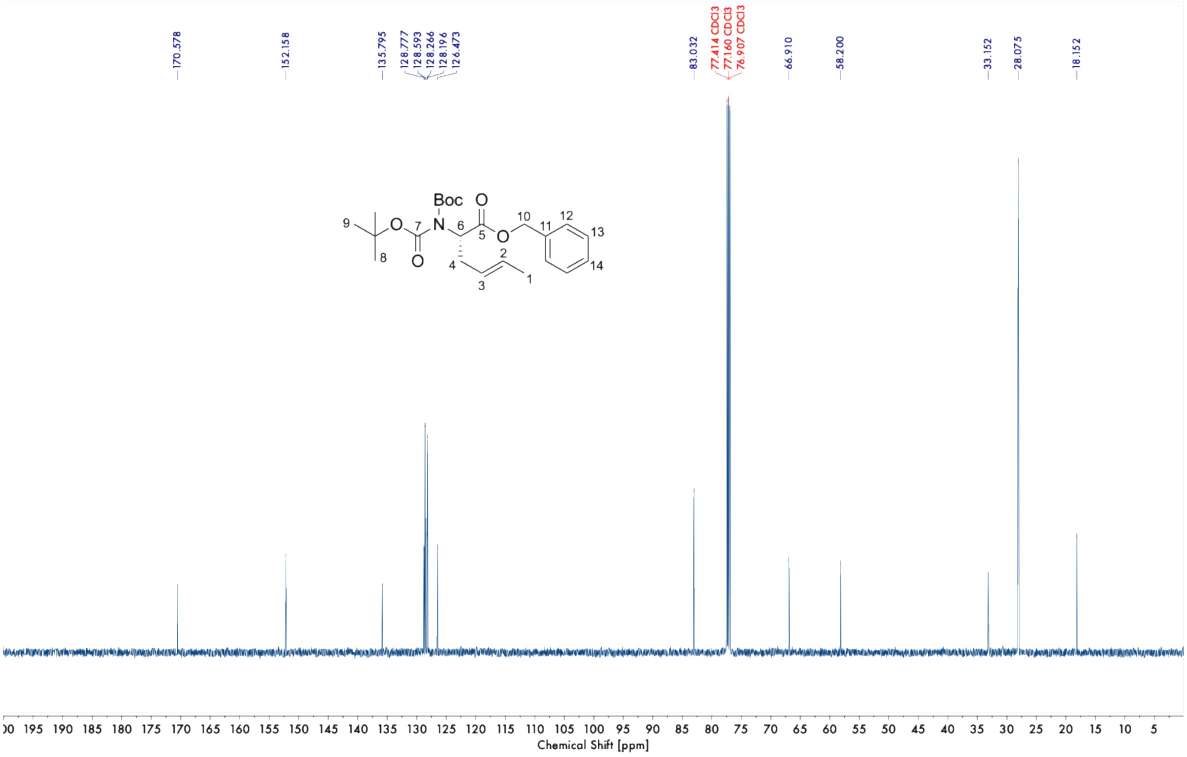

(^{1}H,^{1}H)-COSY (CDCl$_3$, **10**):

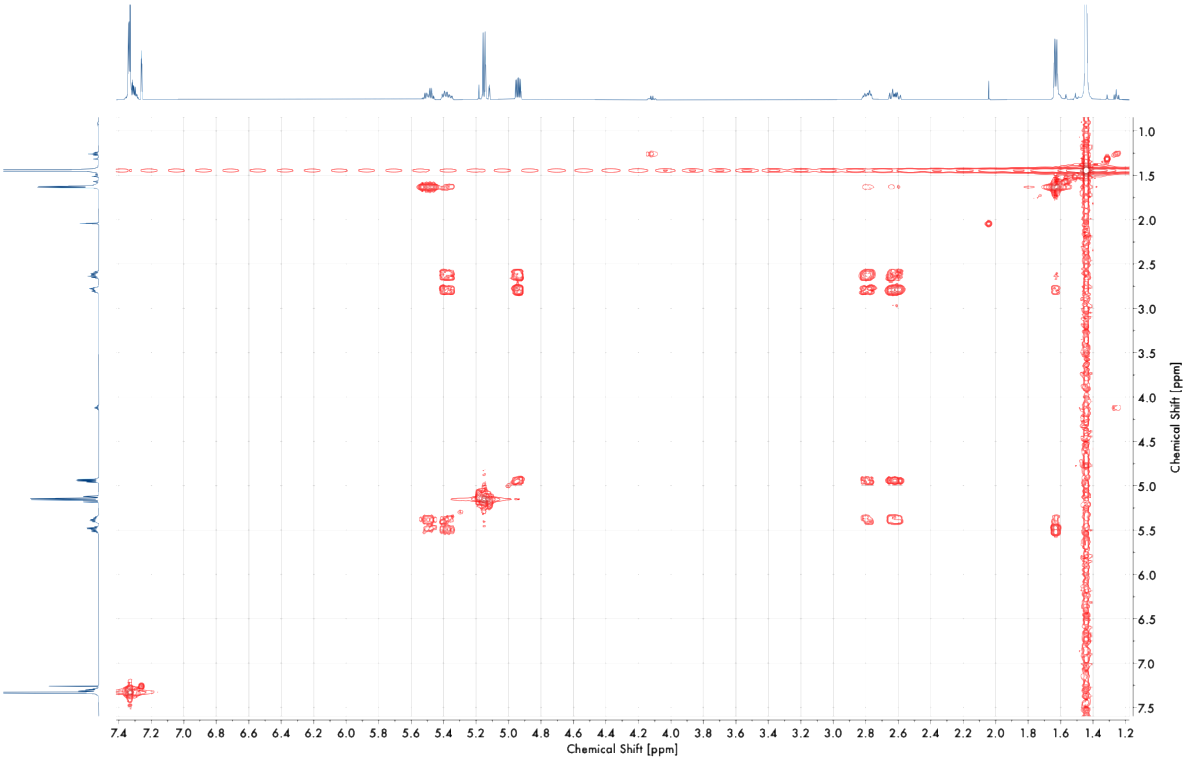

(^{1}H,^{13}C)-HSQC (CDCl$_3$, **10**):

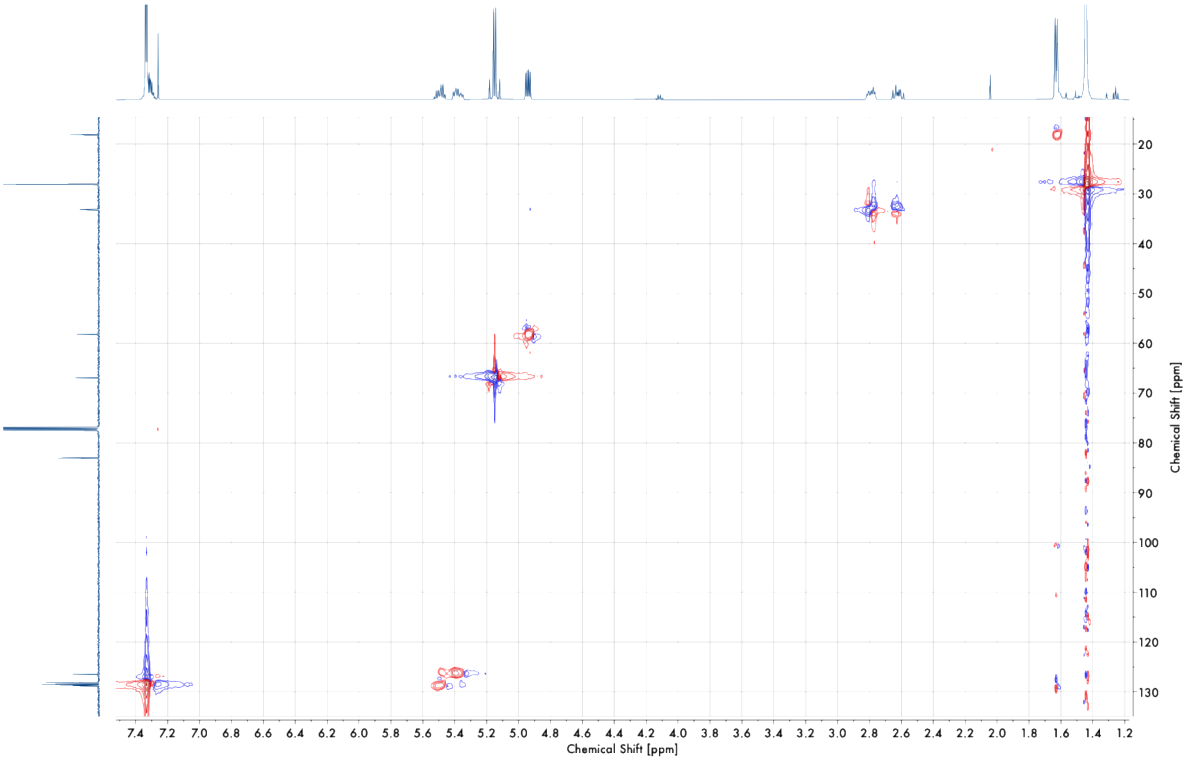

(^{1}H,^{13}C)-HMBC (CDCl$_3$, **10**):

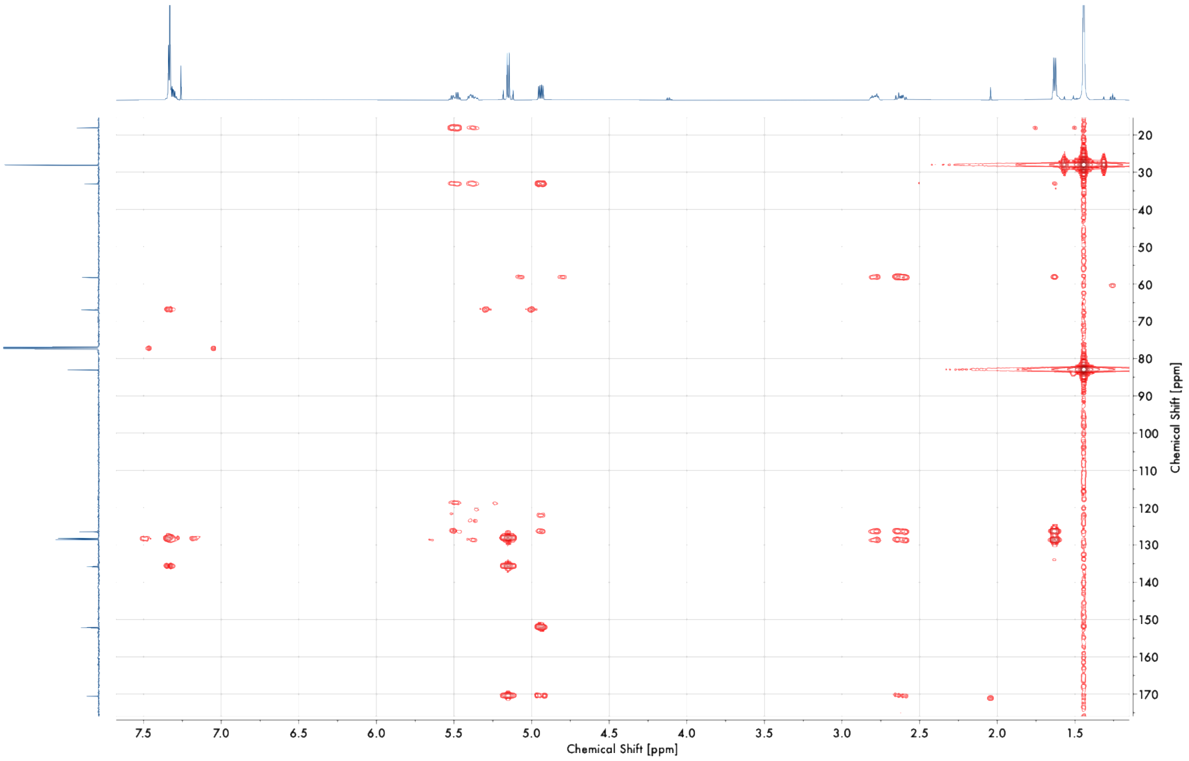

Benzyl (2*S*,4*R*,5*R*)-2-(bis(*tert*-butoxycarbonyl)amino)-4,5-dihydroxyhexanoate [11]

^{1}H NMR (500 MHz, CDCl$_3$, **11**):

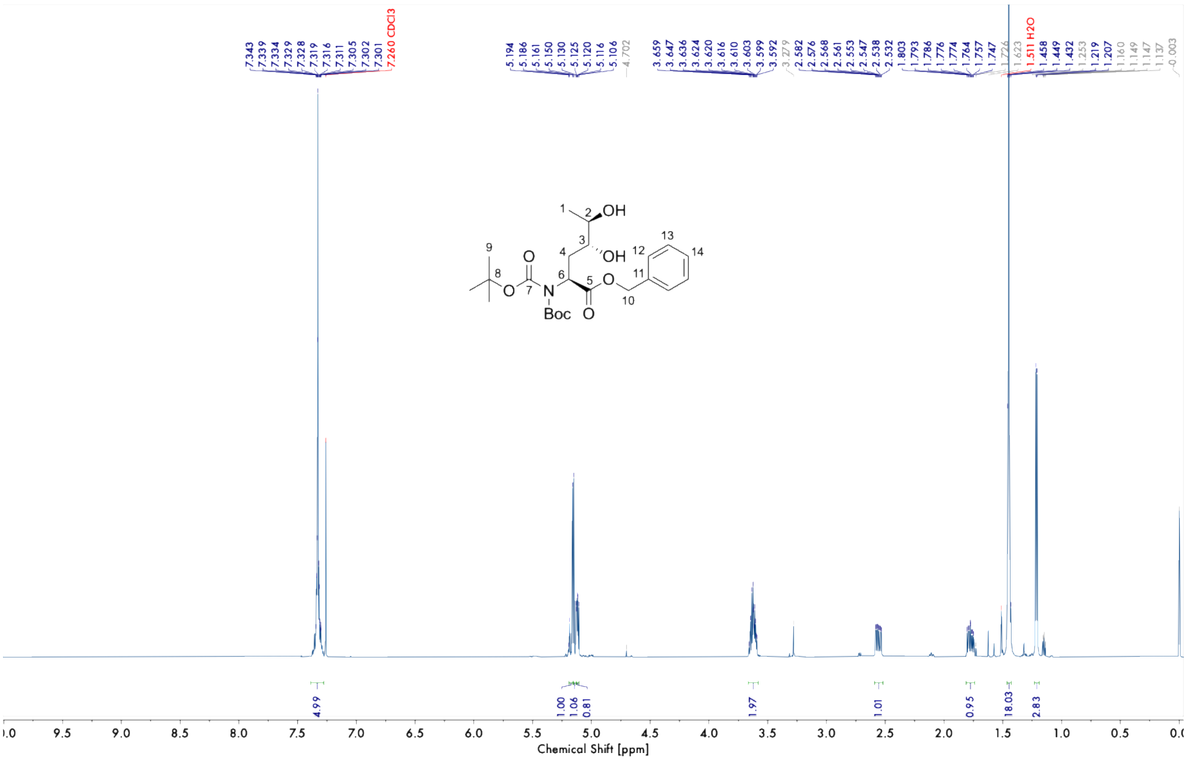

^{13}C NMR (126 MHz, $CDCl_3$, **11**):

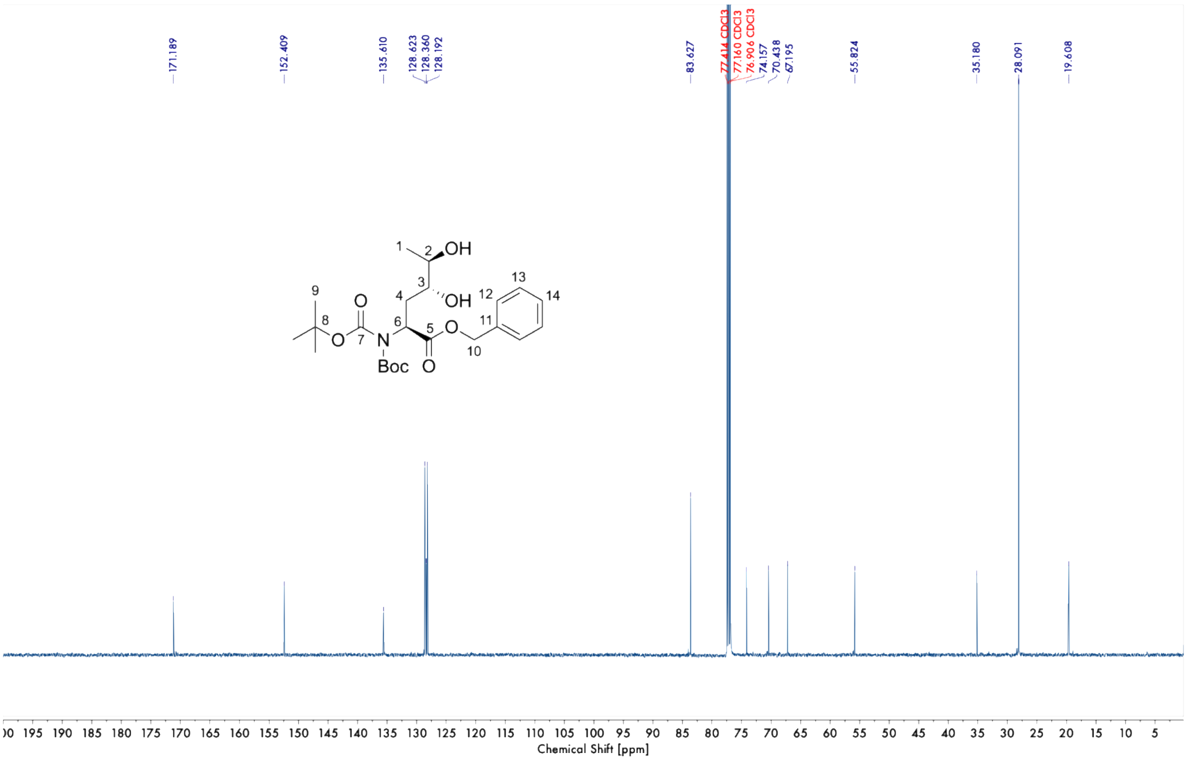

(^{1}H,^{1}H)-COSY ($CDCl_3$, **11**):

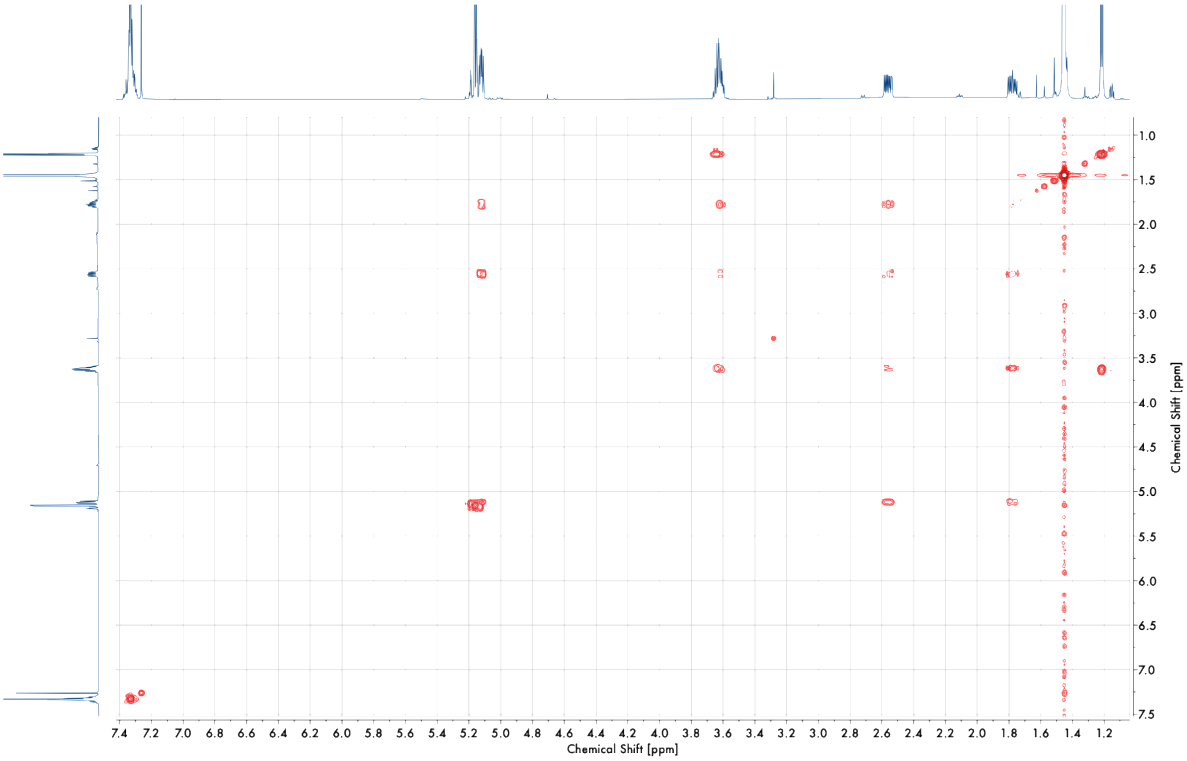

(^{1}H,^{13}C)-HSQC (CDCl$_3$, **11**):

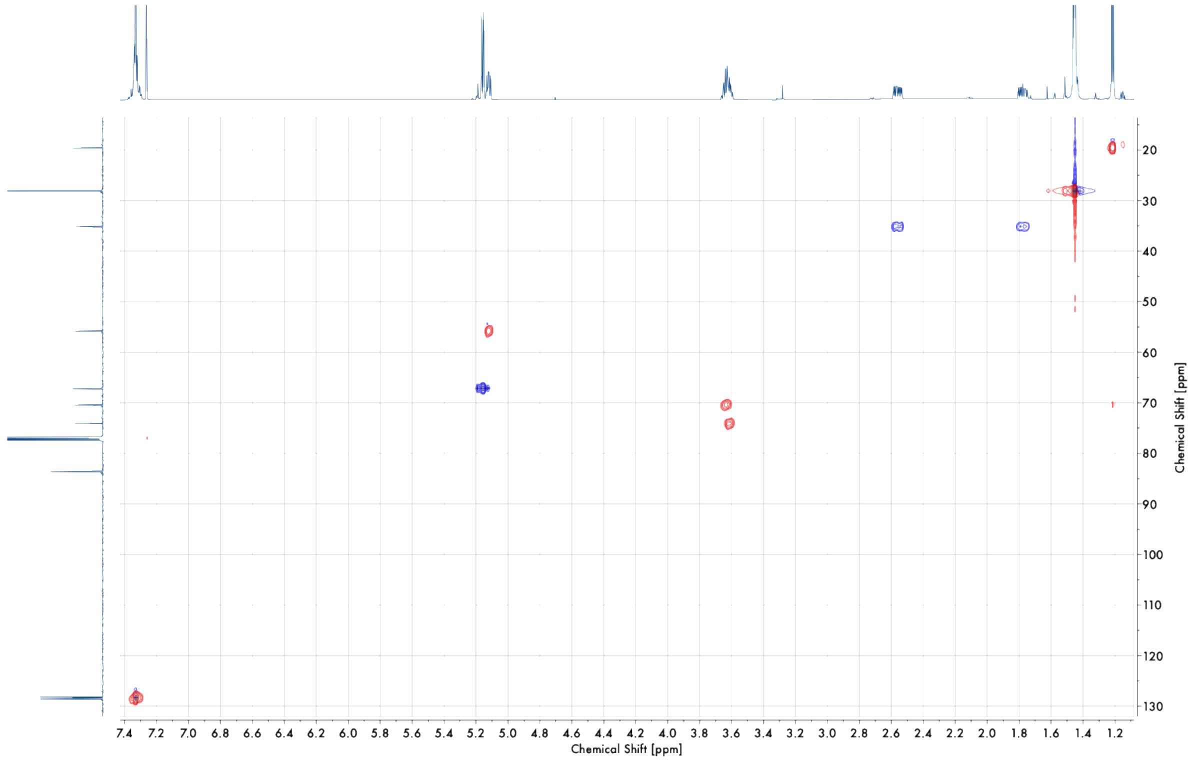

(^{1}H,^{13}C)-HMBC (CDCl$_3$, **11**):

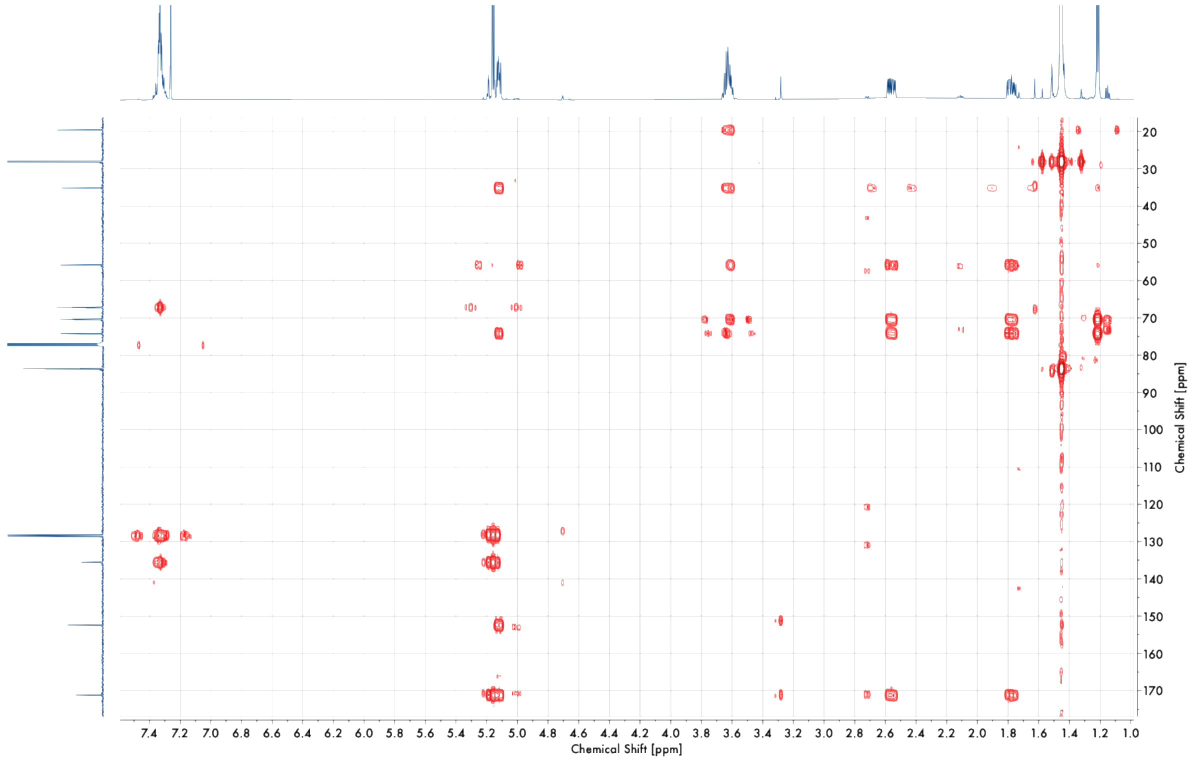

Benzyl (*S*)-2-(bis(*tert*-butoxycarbonyl)amino)-3-((4*R*,5*R*)-5-methyl-2,2-dioxido-1,3,2-dioxathiolan-4-yl)propanoate [12]

^{1}H NMR (400 MHz, DMSO-d_6, **12**):

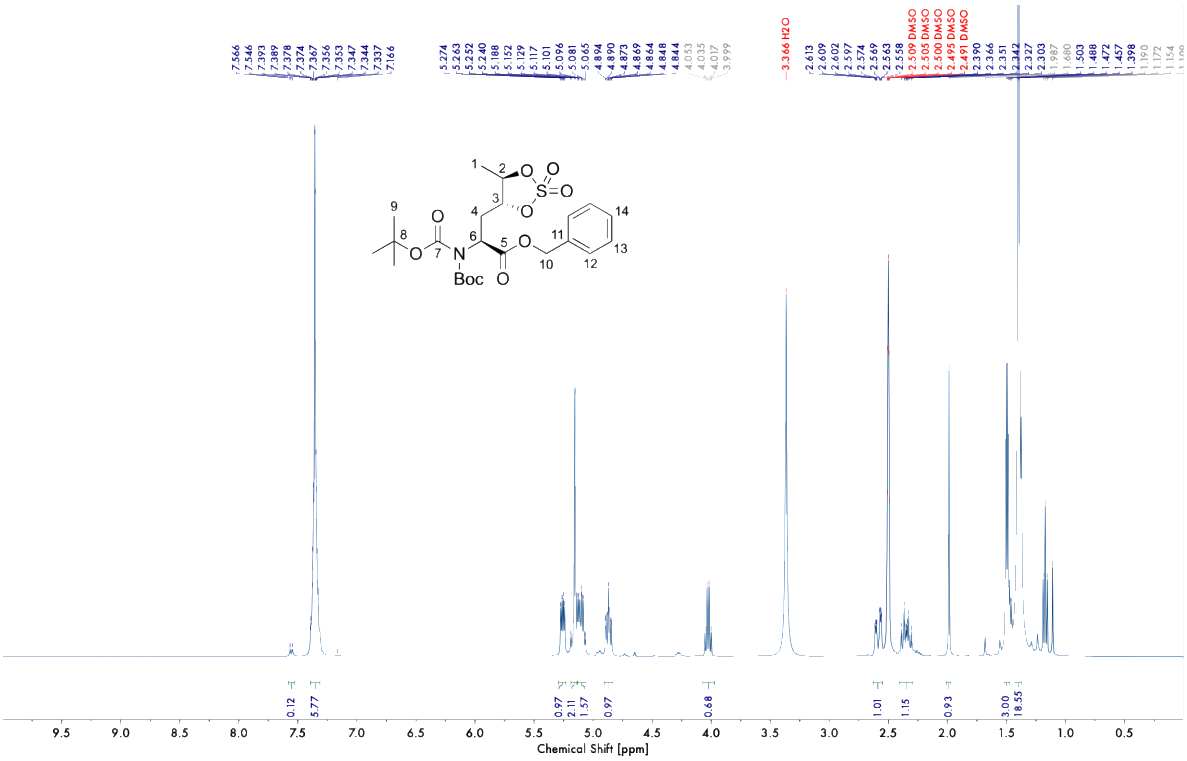

^{13}C NMR (101 MHz, DMSO-d_6, **12**):

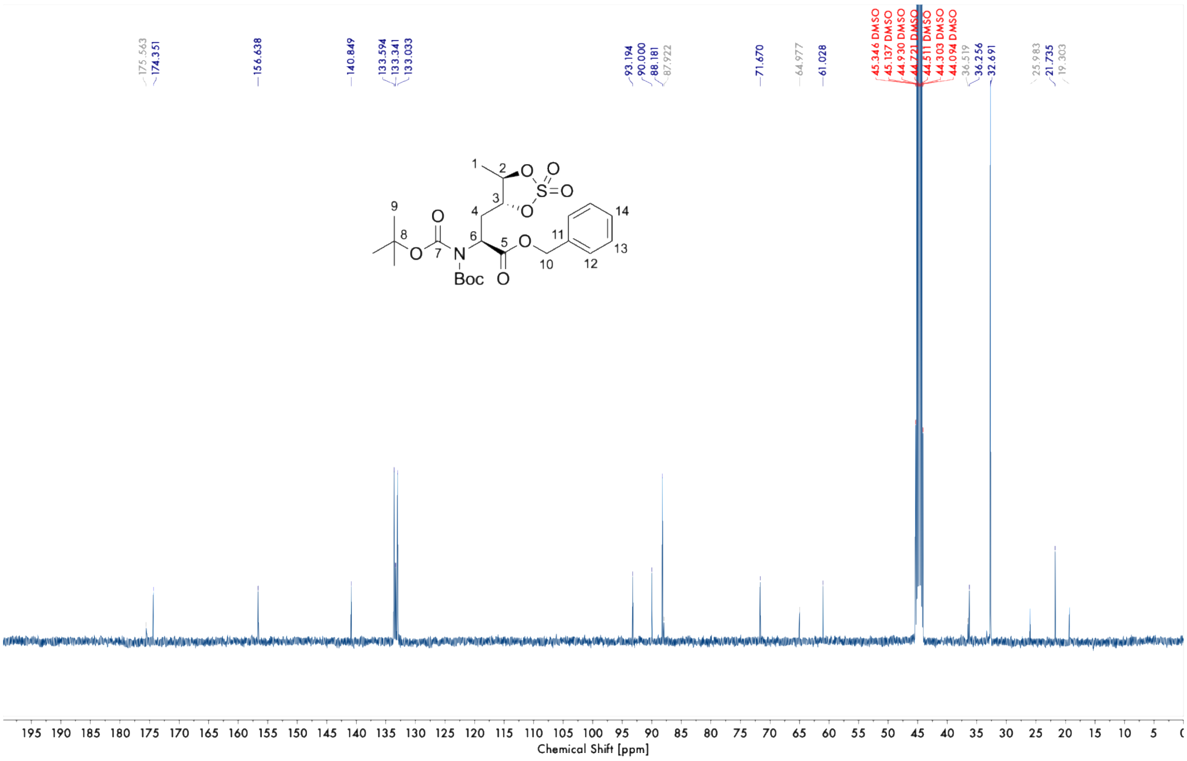

(^{1}H,^{1}H)-COSY (DMSO-d_6, **12**):

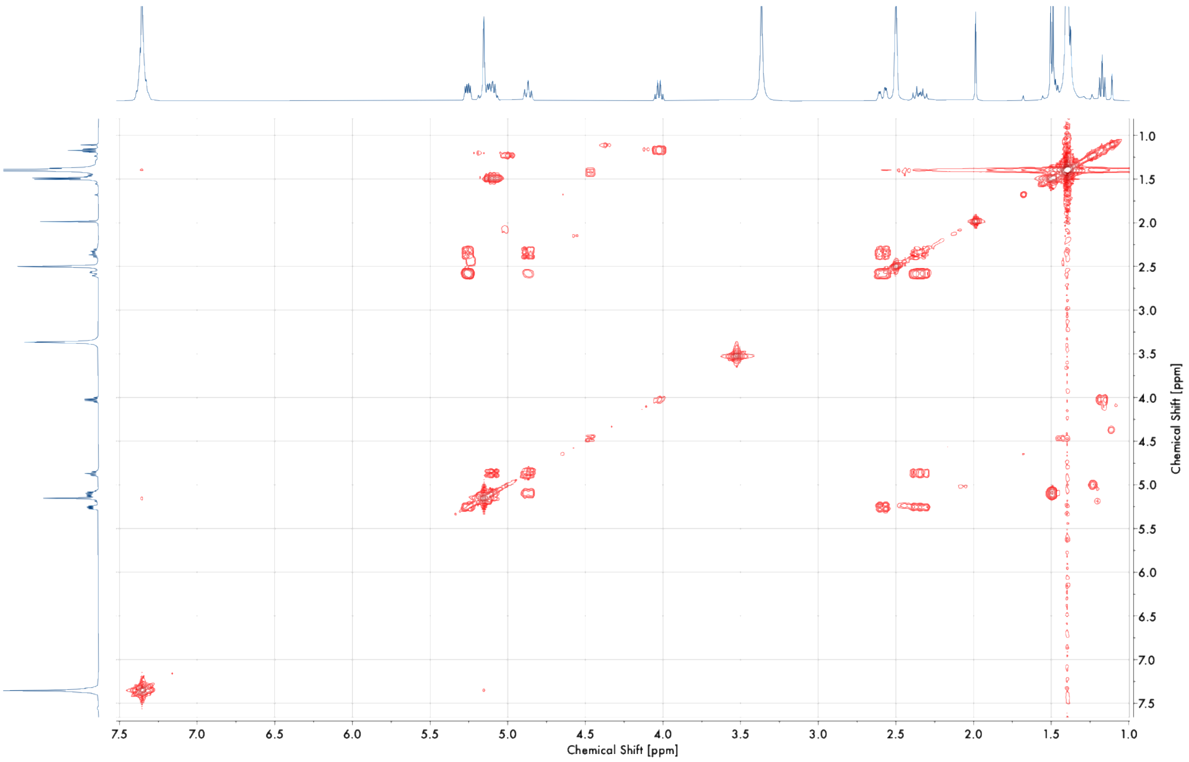

(^{1}H,^{13}C)-HSQC (DMSO-d_6, **12**):

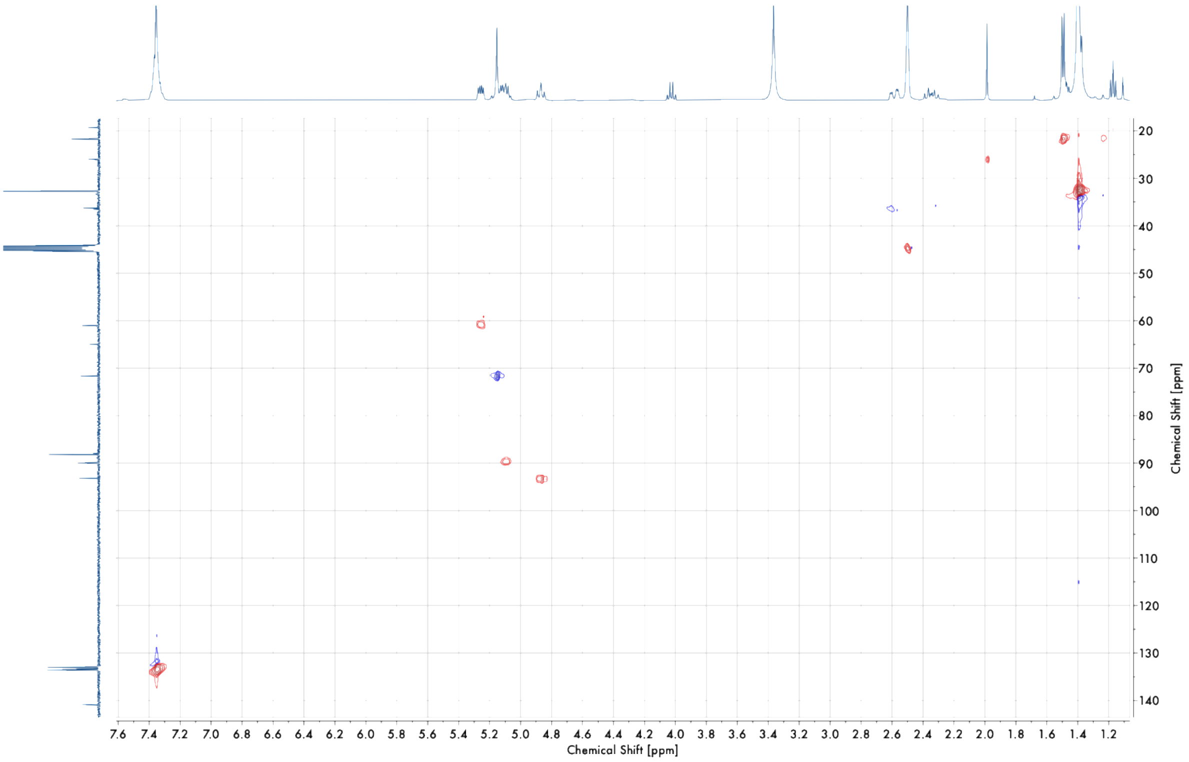

(^{1}H,^{13}C)-HMBC (DMSO-d_6, **12**):

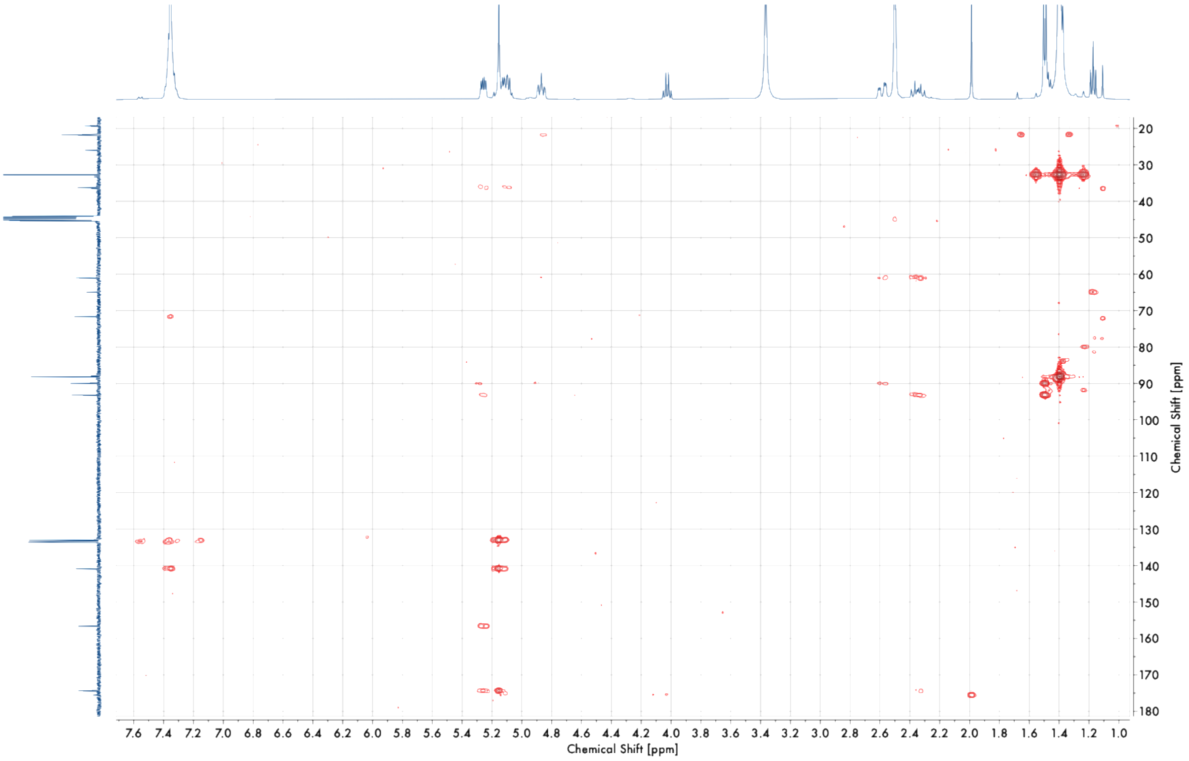

Fmoc-MeHyPro-OBn [13]

^{1}H NMR (500 MHz, DMSO-d_6, 373 K, **13**):

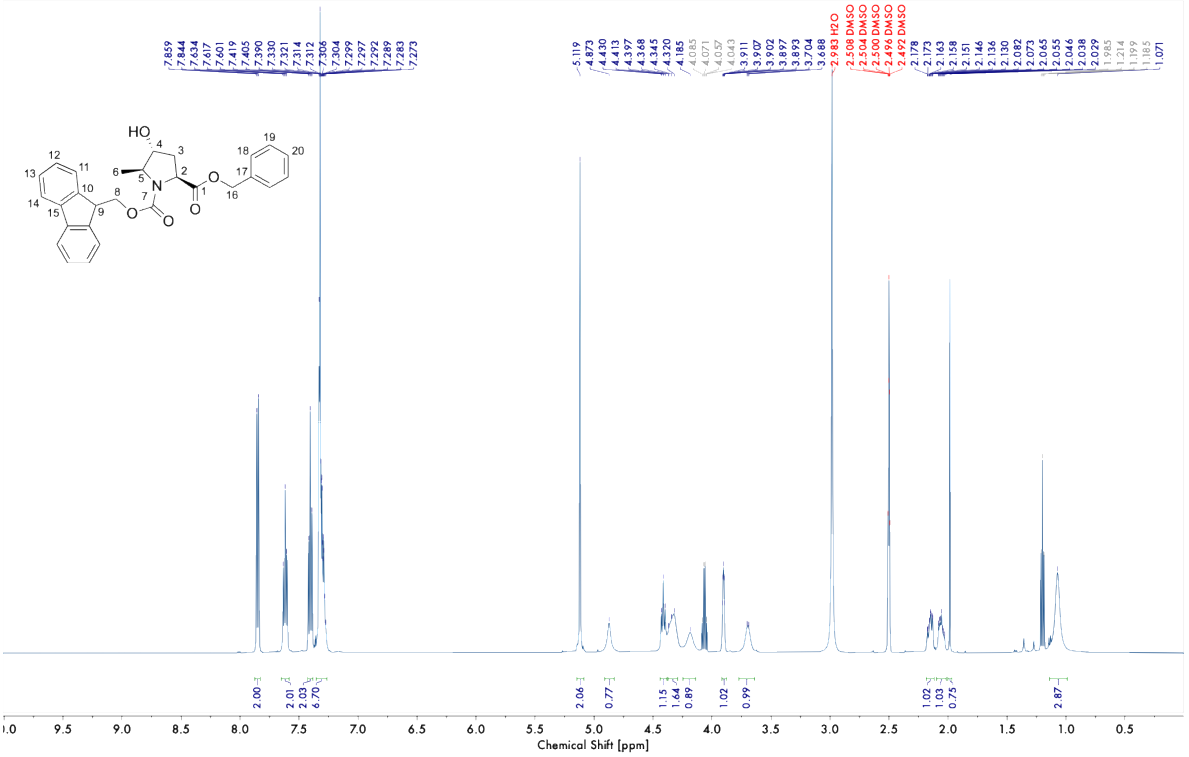

^{13}C NMR (126 MHz, DMSO-d_6, 373 K, **13**):

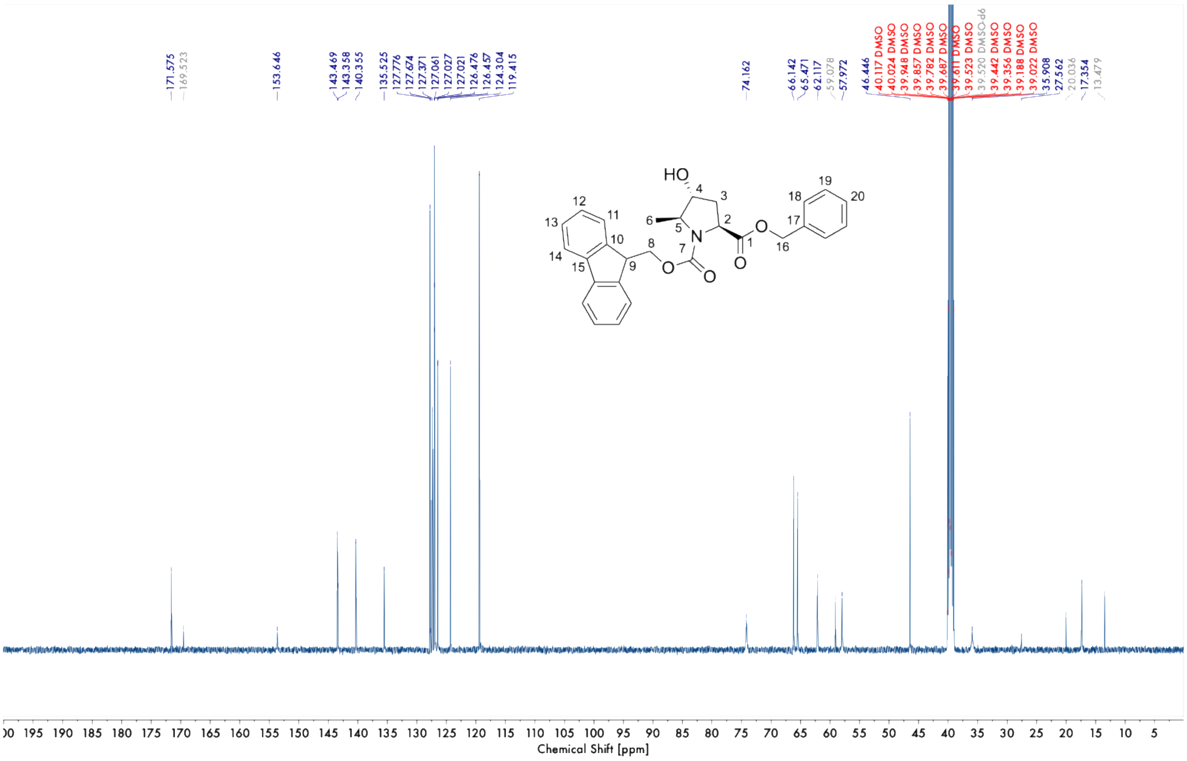

(^{1}H,^{1}H)-COSY (DMSO-d_6, 373 K, **13**):

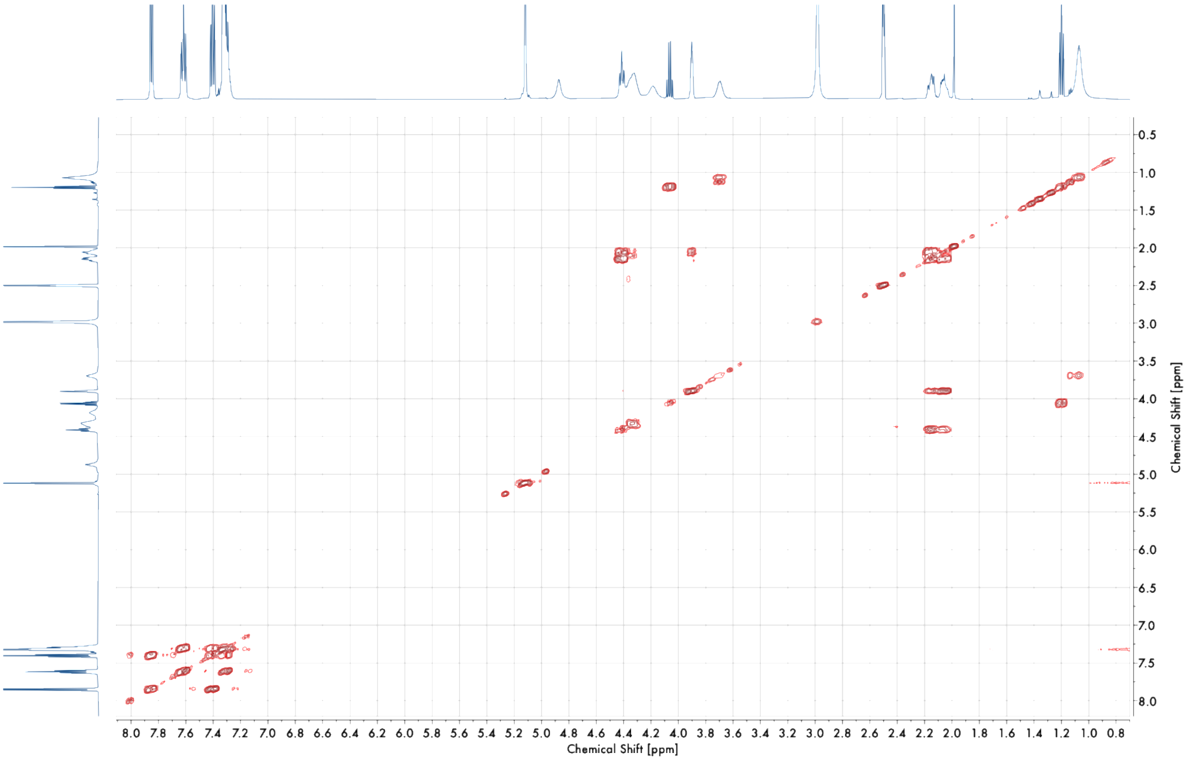

(^{1}H,^{13}C)-HSQC (DMSO-d_6, 373 K, **13**):

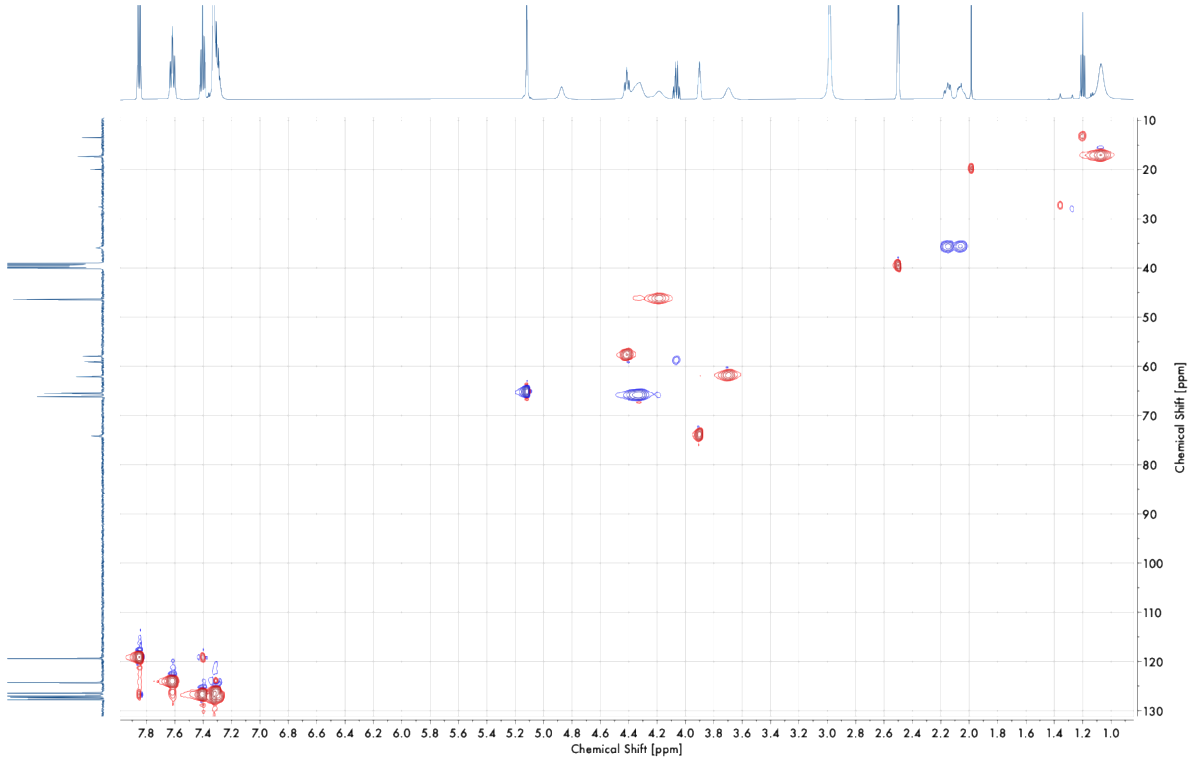

(^{1}H,^{13}C)-HMBC (DMSO-d_6, 373 K, **13**):

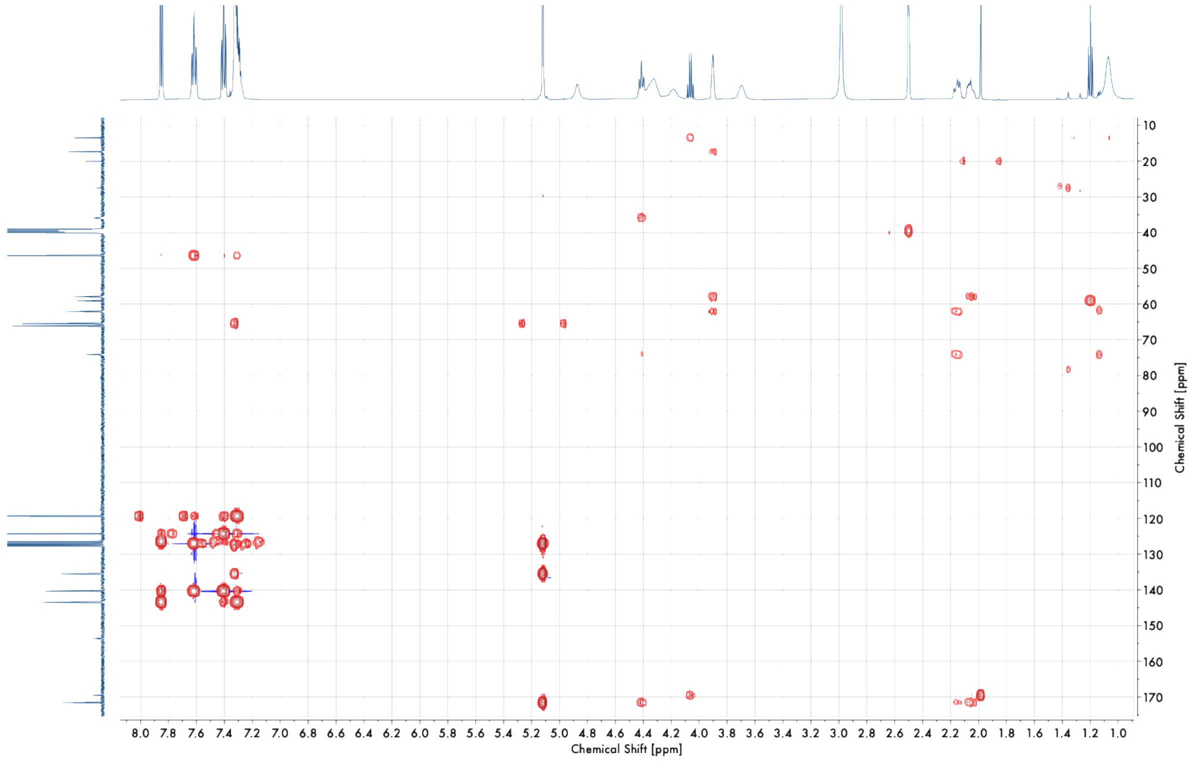

Fmoc-MeHyPro(*O*Ac)-OBn [SI-2]

^{1}H NMR (500 MHz, $CDCl_3$, **SI-2**):

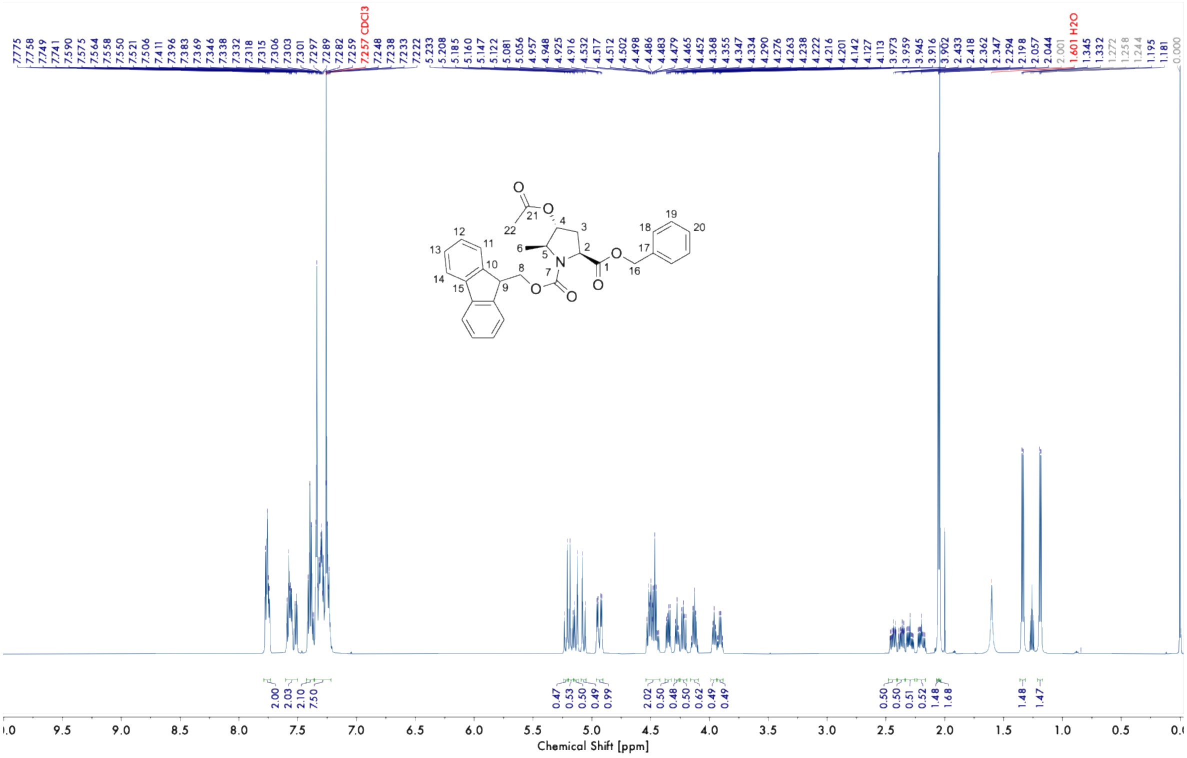

^{13}C NMR (126 MHz, $CDCl_3$, **SI-2**):

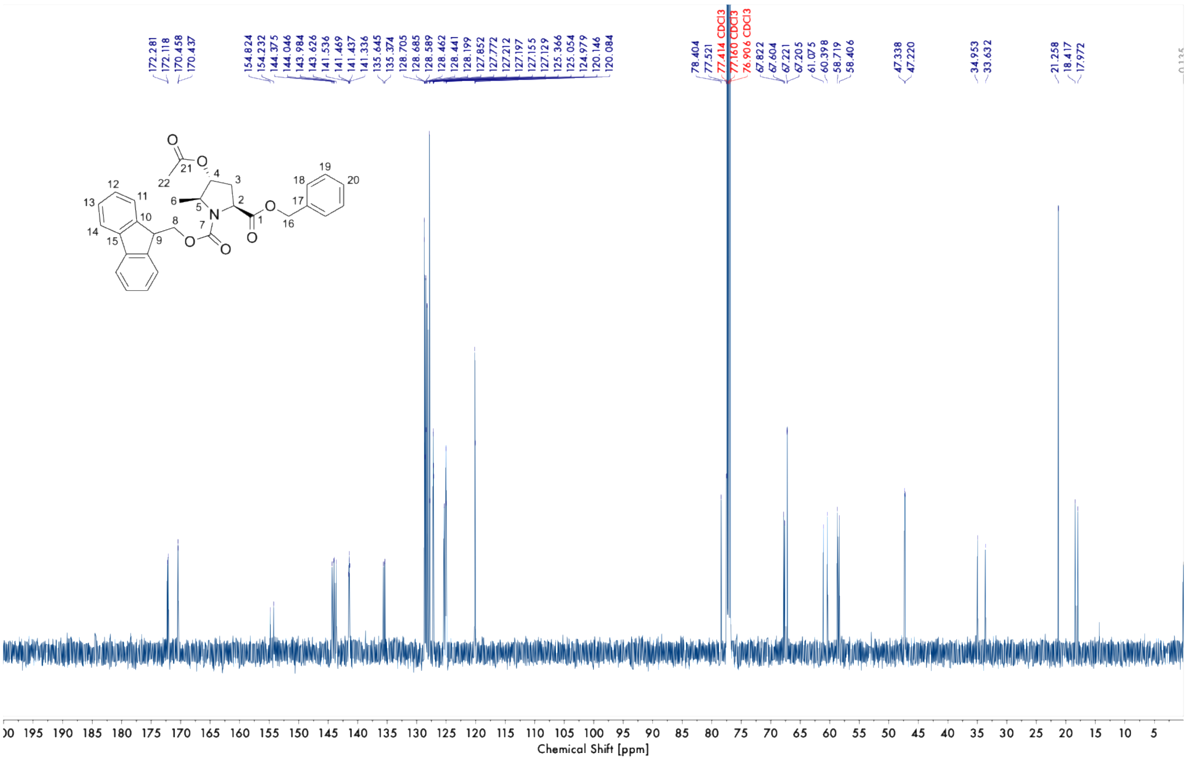

(^{1}H,^{1}H)-COSY (CDCl$_3$, **SI-2**):

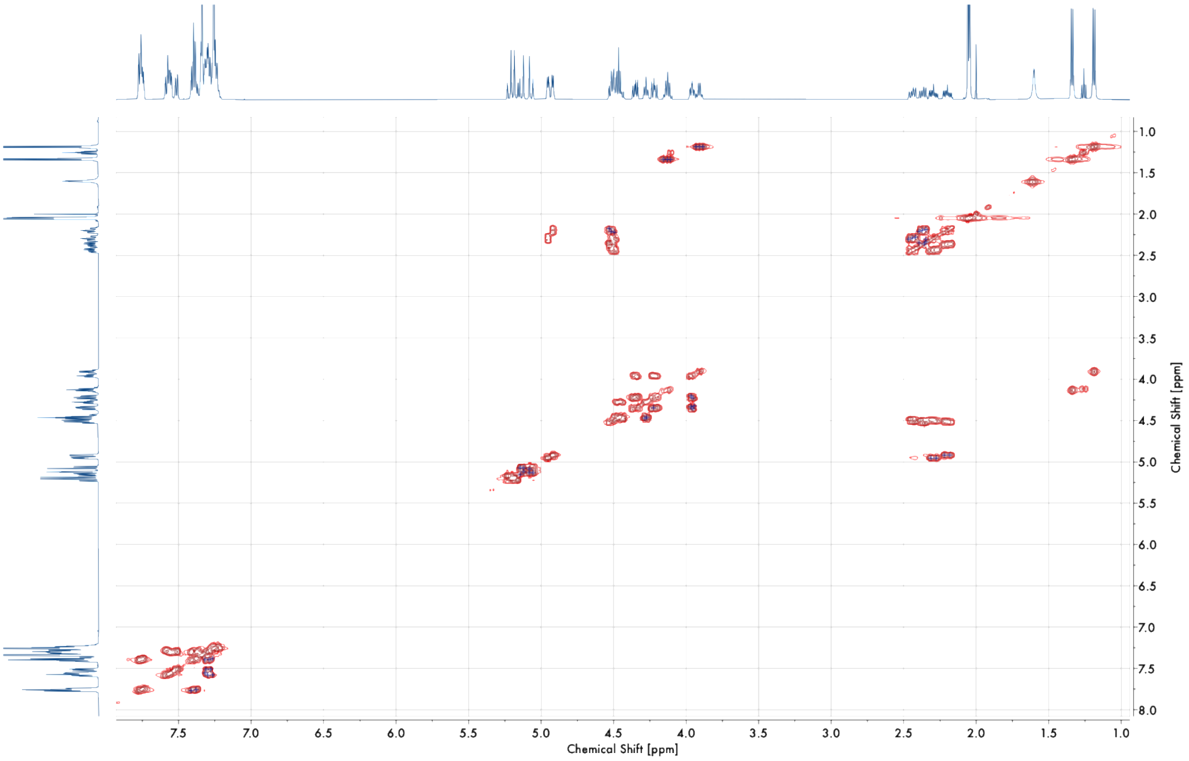

(^{1}H,^{13}C)-HSQC (CDCl$_3$, **SI-2**):

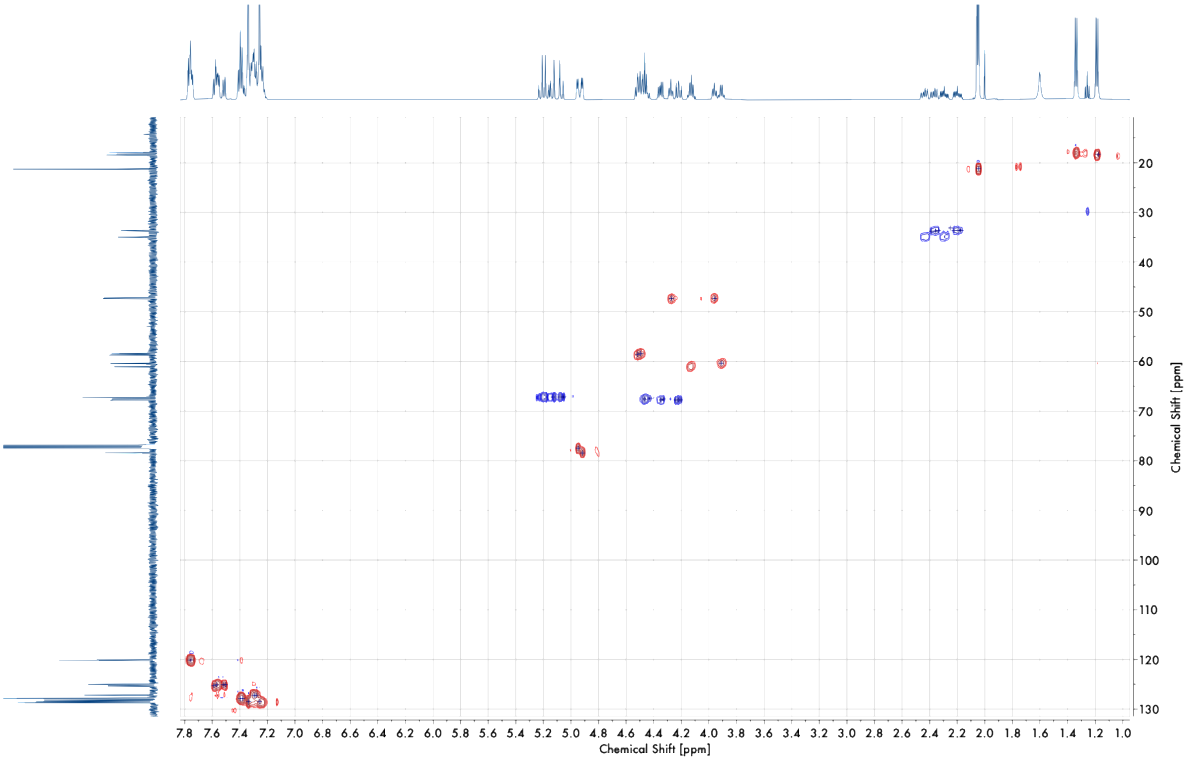

(¹H,¹³C)-HMBC (CDCl₃, **SI-2**):

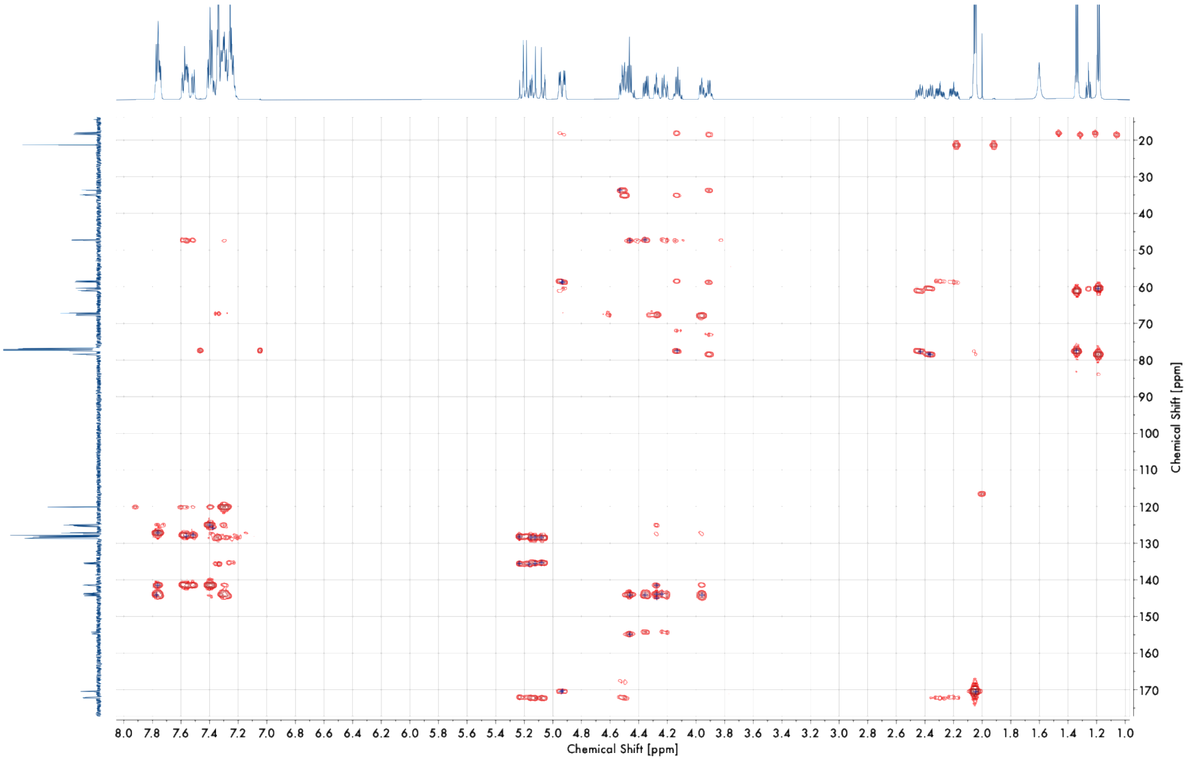

Fmoc-MeHyPro(*O*Ac)-OH [14]

^{1}H NMR (500 MHz, DMSO-d_6, 373 K, **14**):

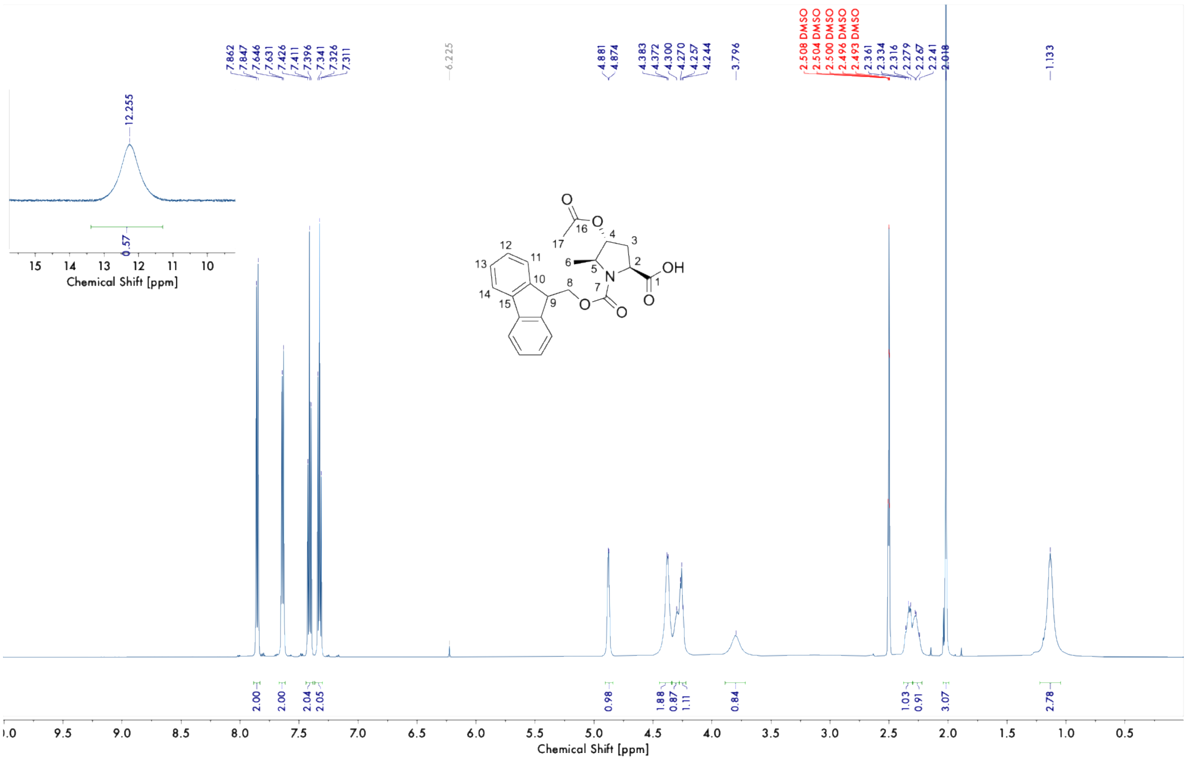

^{13}C NMR (126 MHz, DMSO-d_6, 373 K, **14**):

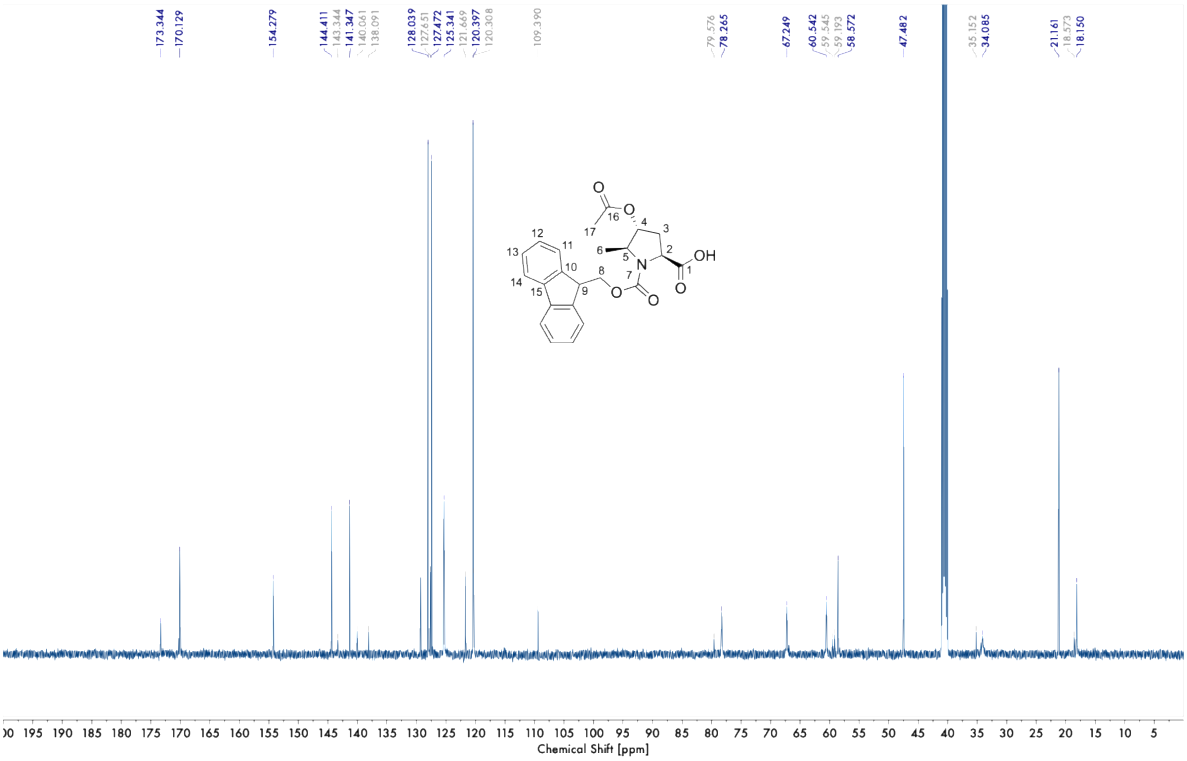

(^{1}H,^{1}H)-COSY (DMSO-d_6, 373 K, **14**):

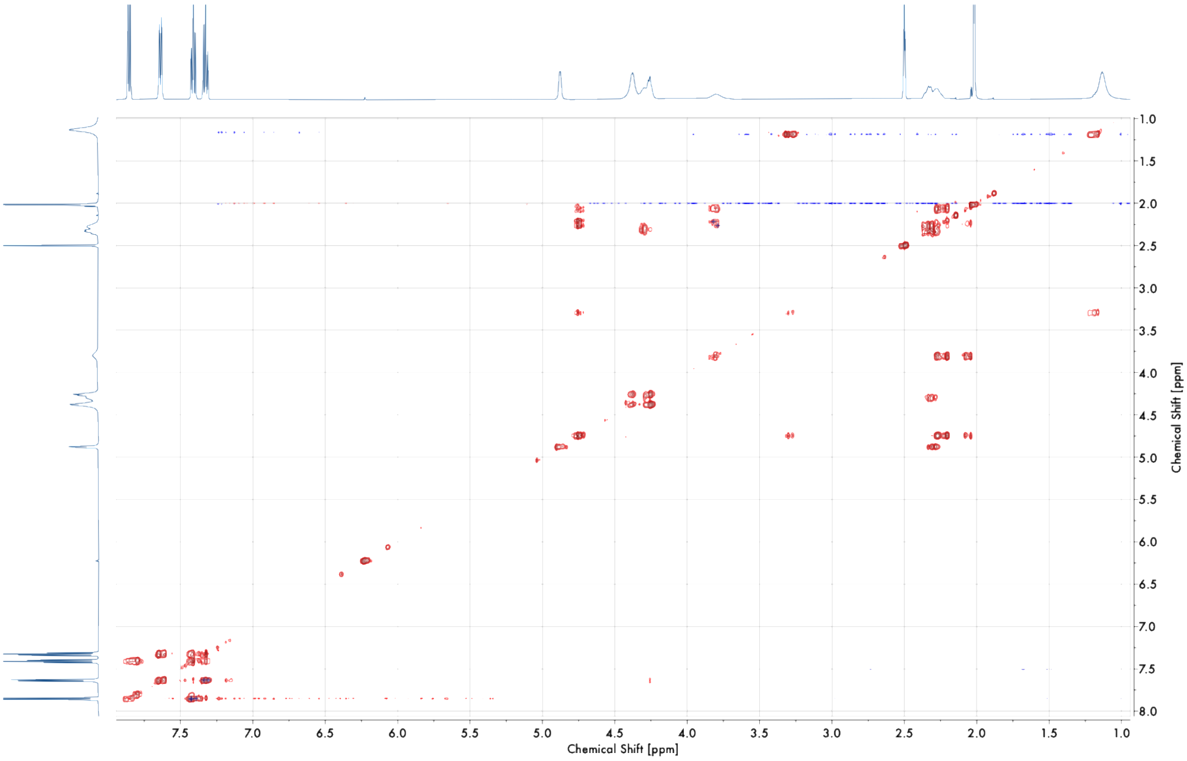

(^{1}H,^{13}C)-HSQC (DMSO-d_6, 373 K, **14**):

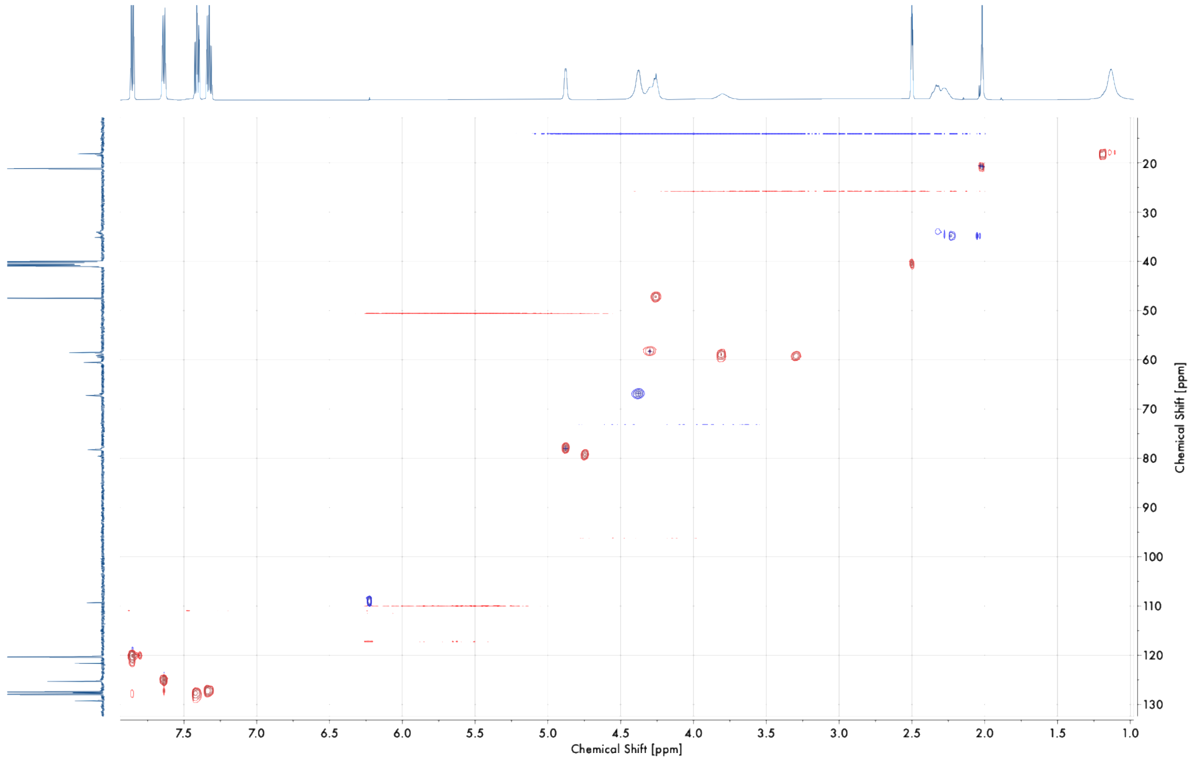

(^{1}H,^{13}C)-HMBC (DMSO-d_6, 373 K, **14**):

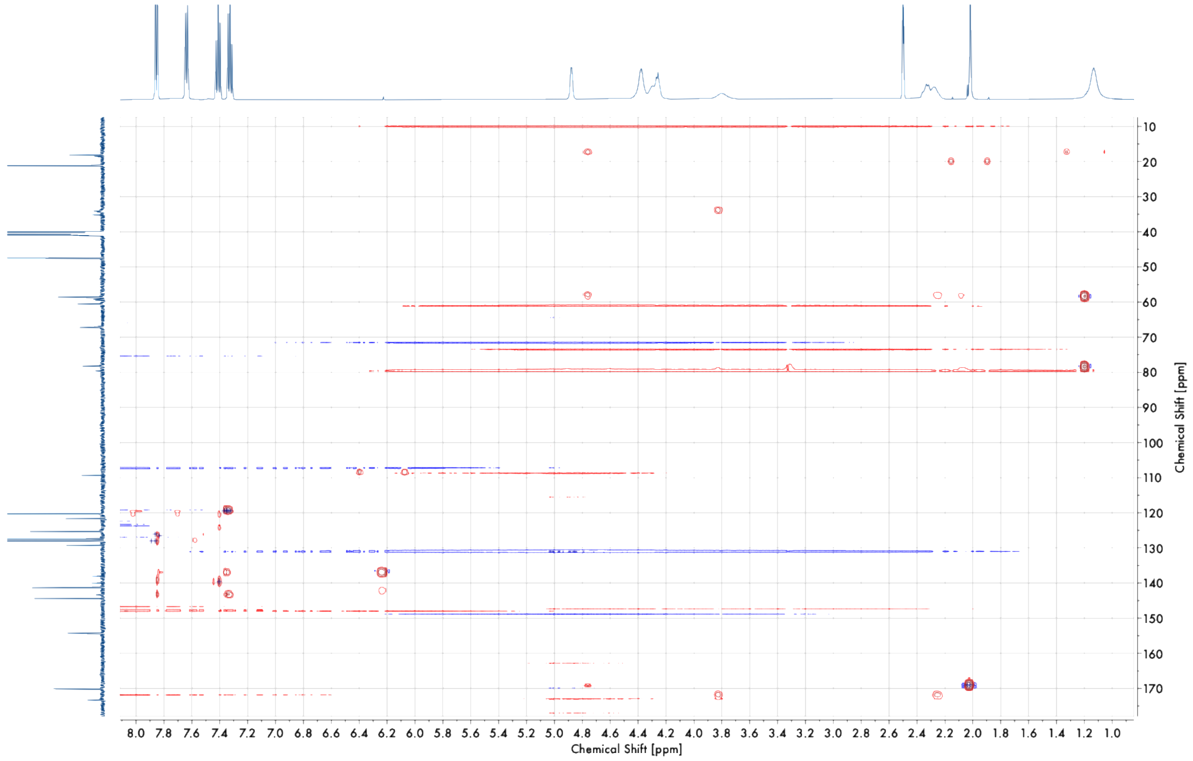

4-(Allyloxy)-3,5-dichlorobenzaldehyde [15]

^{1}H NMR (500 MHz, $CDCl_3$, **15**):

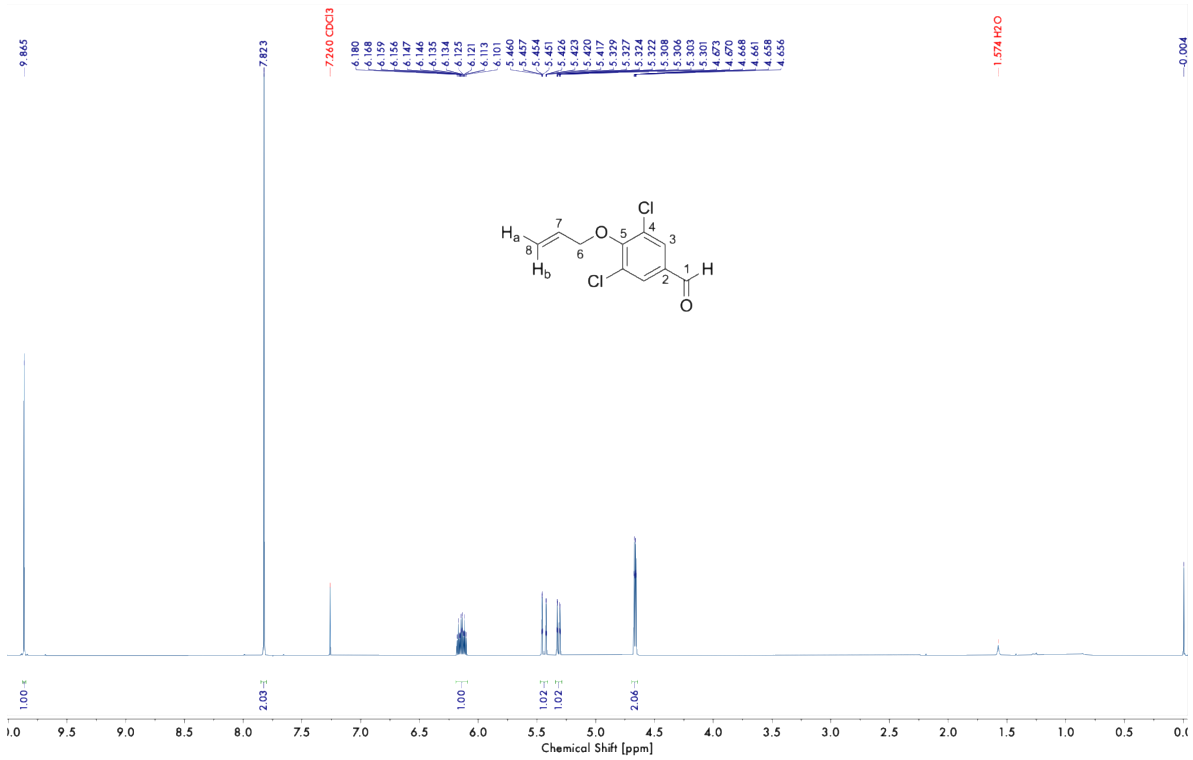

^{13}C NMR (126 MHz, $CDCl_3$, **15**):

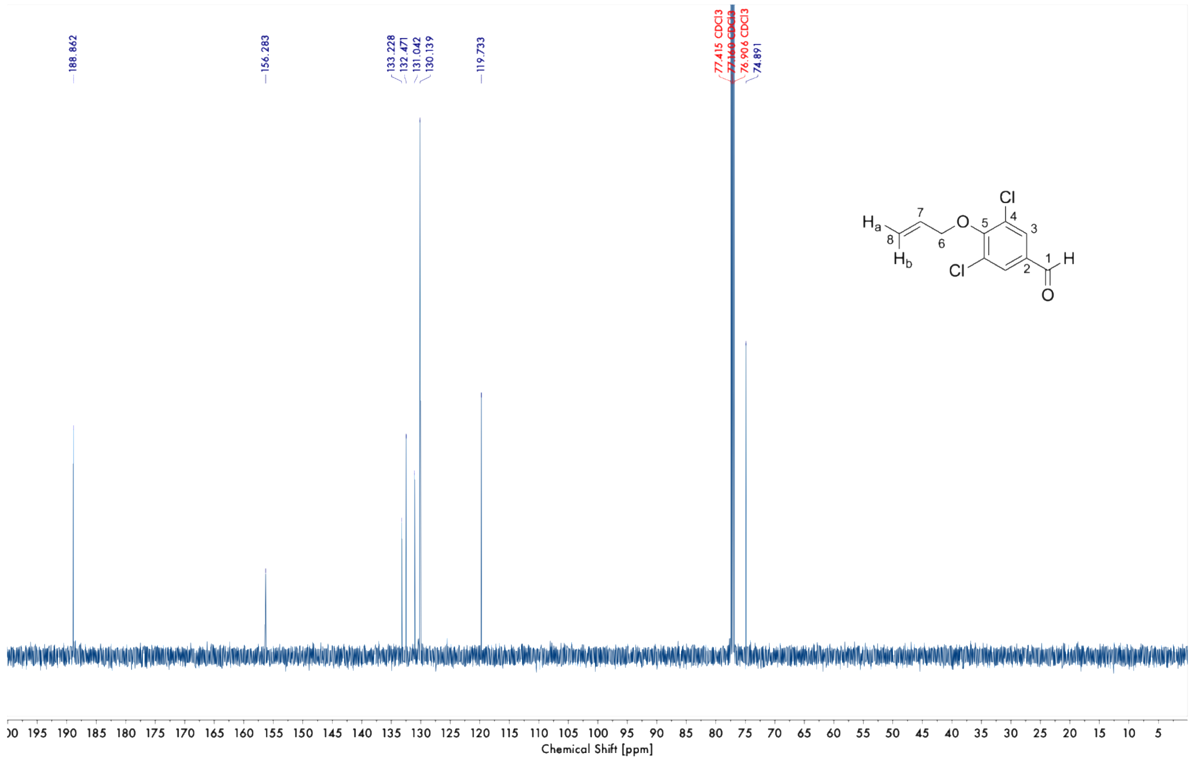

(^{1}H,^{1}H)-COSY (CDCl$_3$, **15**):

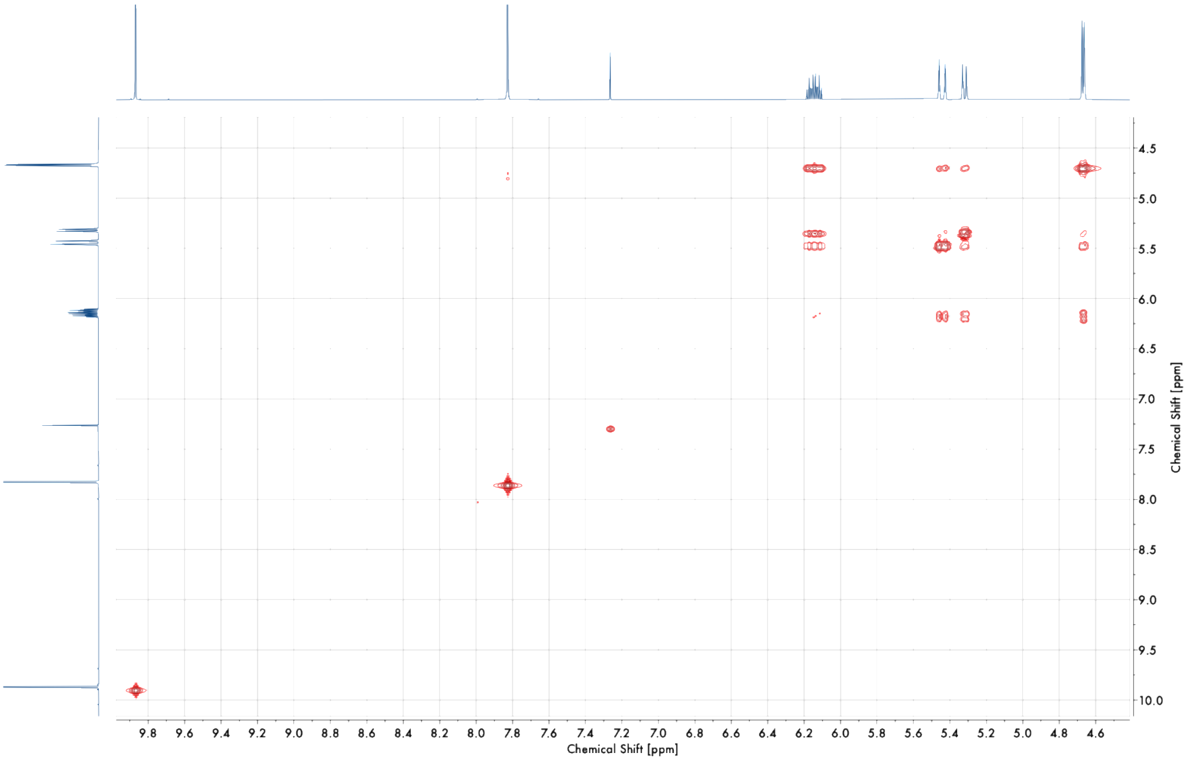

(^{1}H,^{13}C)-HSQC (CDCl$_3$, **15**):

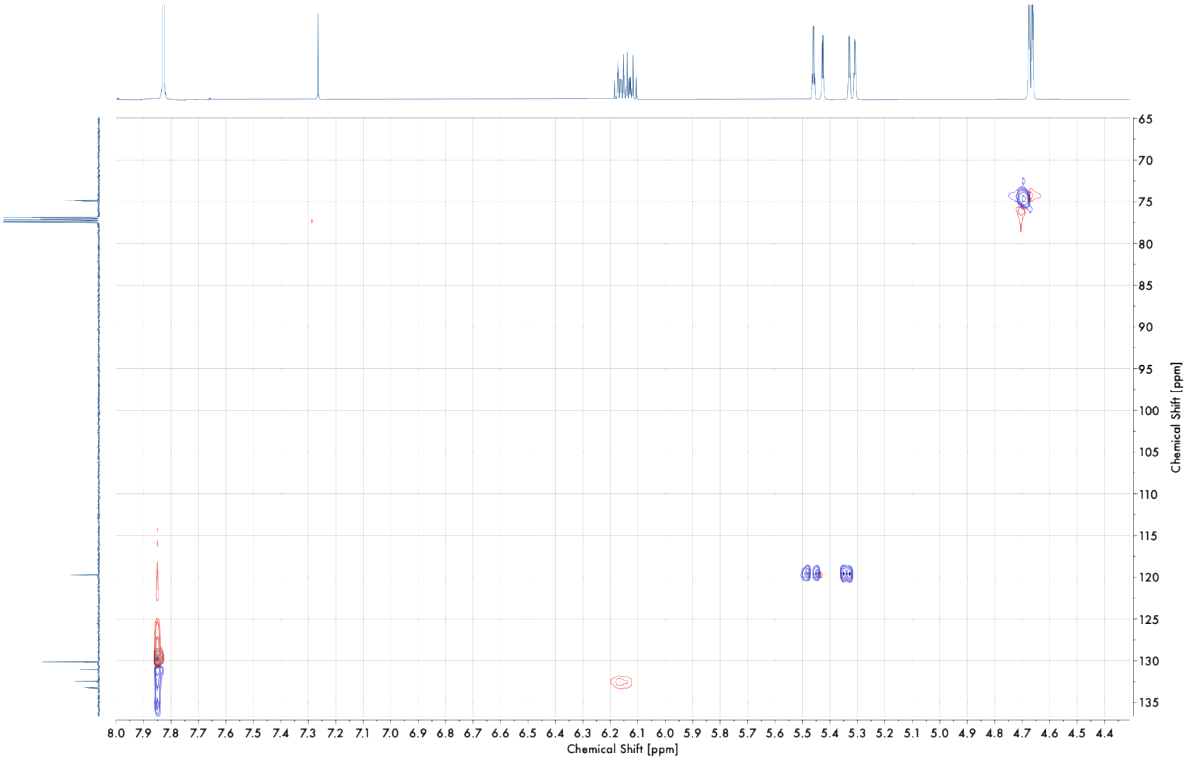

(^{1}H,^{13}C)-HMBC (CDCl$_3$, **15**):

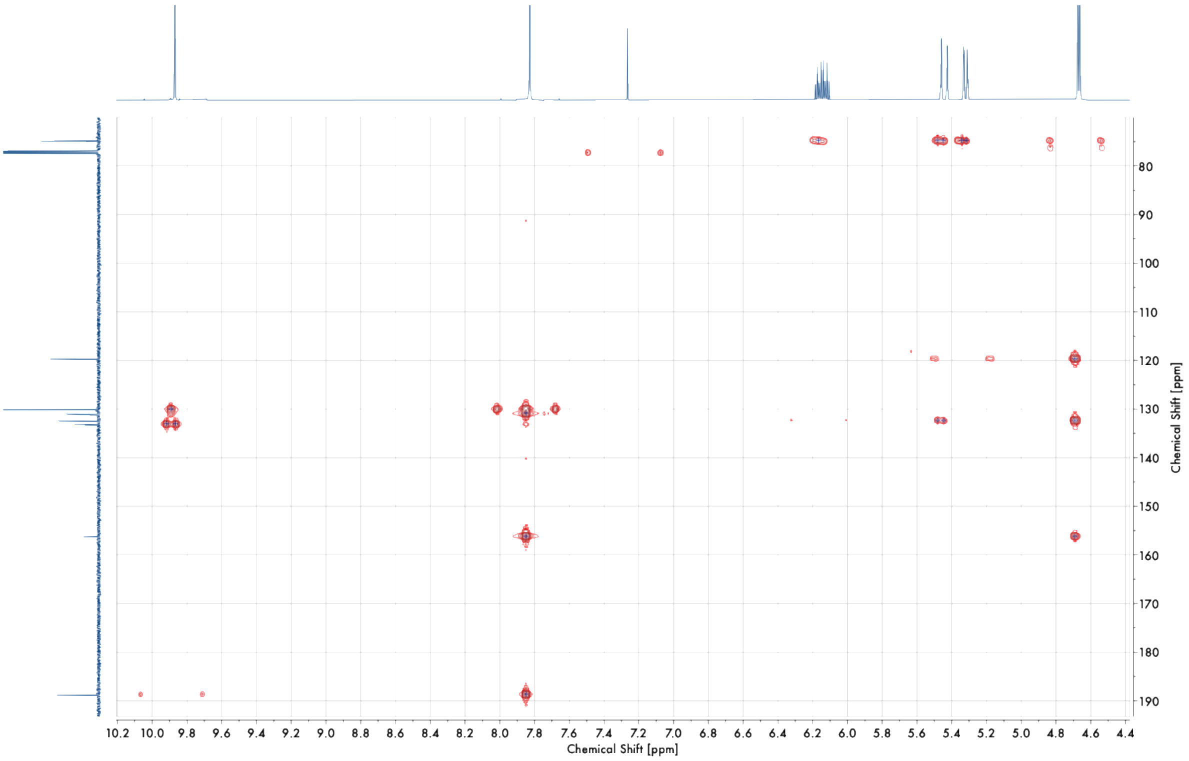

Methyl 2-bromo-2-methoxyacetate [SI-3]

^{1}H NMR (500 MHz, CDCl$_3$, **SI-3**):

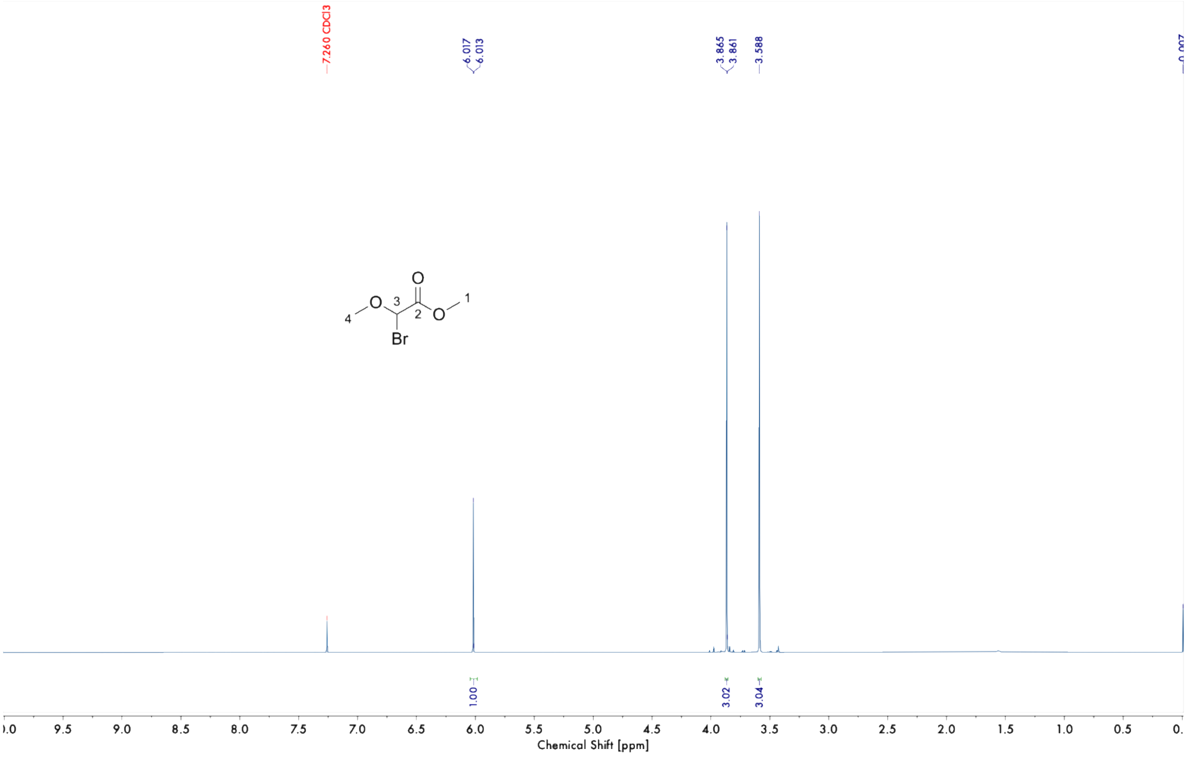

^{13}C NMR (126 MHz, $CDCl_3$, **SI-3**):

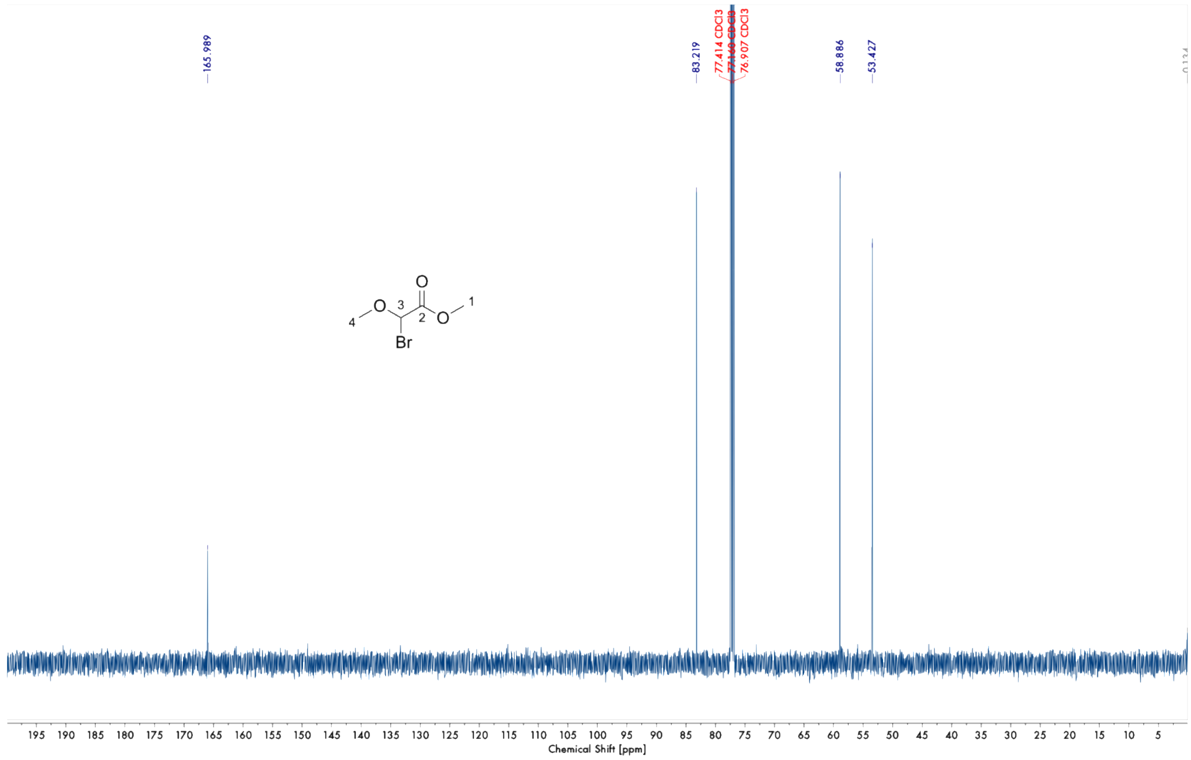

(^{1}H,^{1}H)-COSY ($CDCl_3$, **SI-3**):

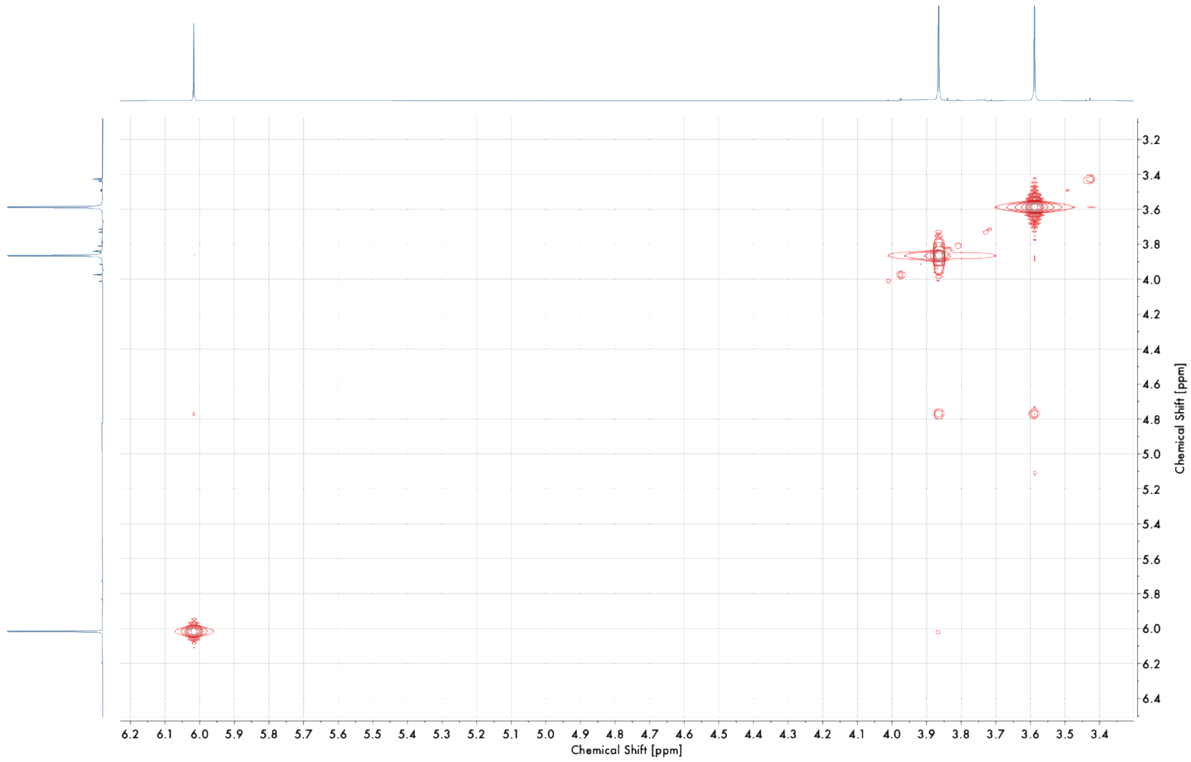

(^{1}H,^{13}C)-HSQC (CDCl$_3$, **SI-3**):

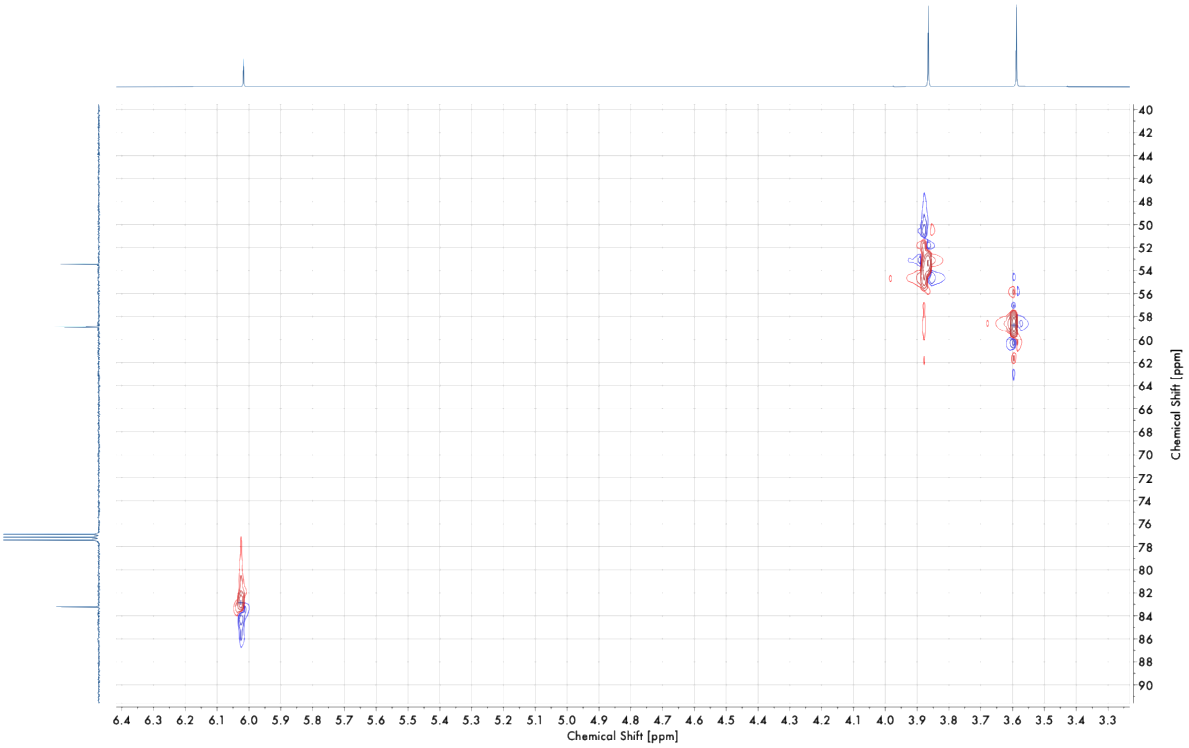

(^{1}H,^{13}C)-HMBC (CDCl$_3$, **SI-3**):

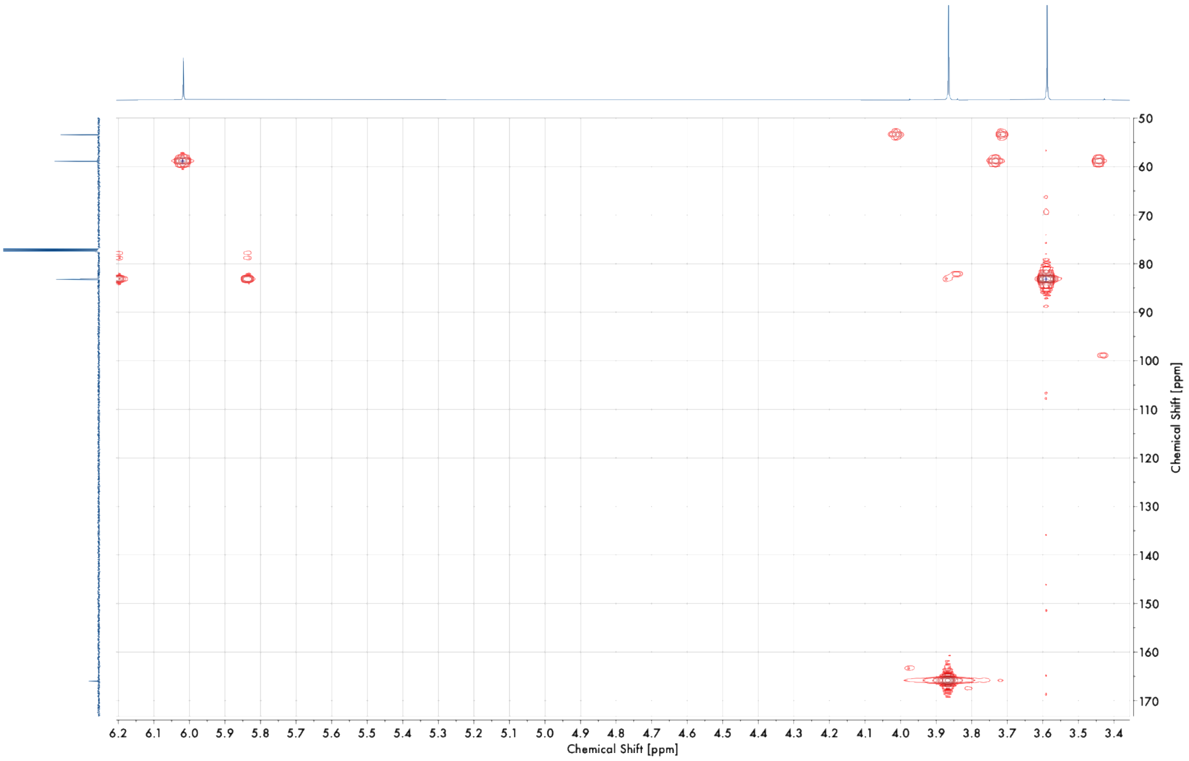

(1,2-Dimethoxy-2-oxoethyl)triphenylphosphonium bromide [16]

^{1}H NMR (500 MHz, $CDCl_3$, **16**):

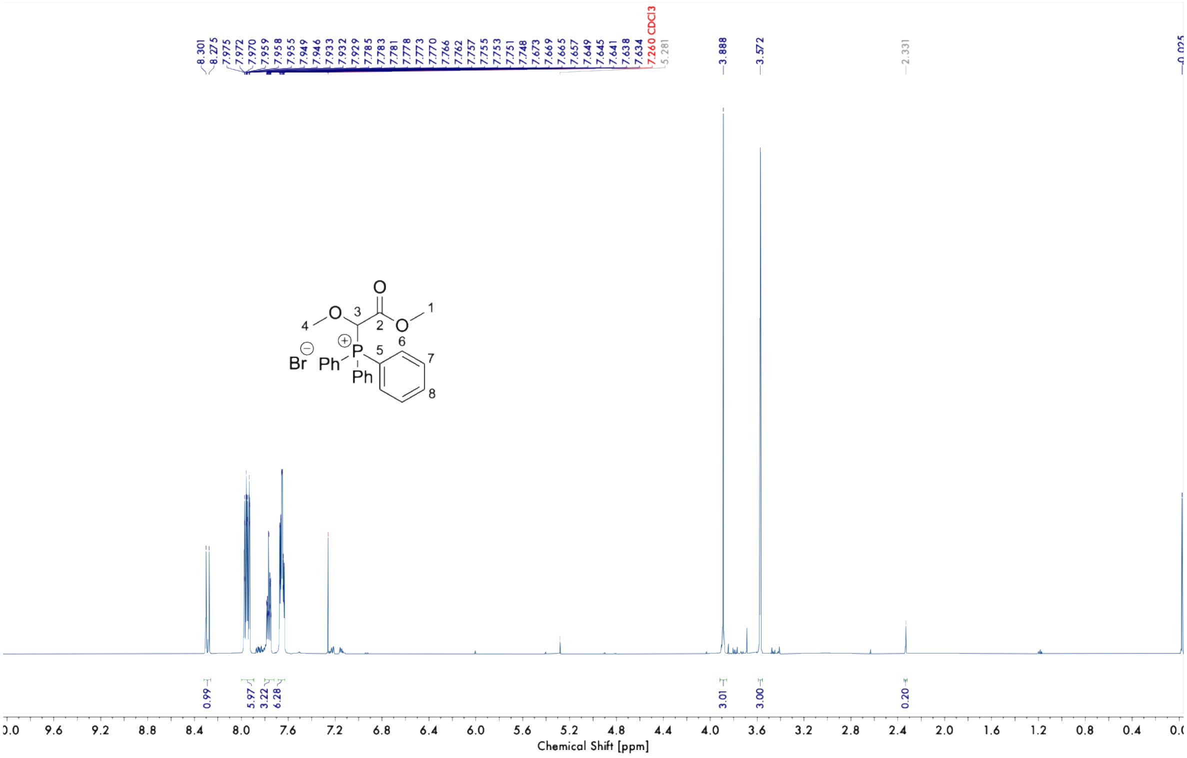

^{13}C NMR (126 MHz, $CDCl_3$, **16**):

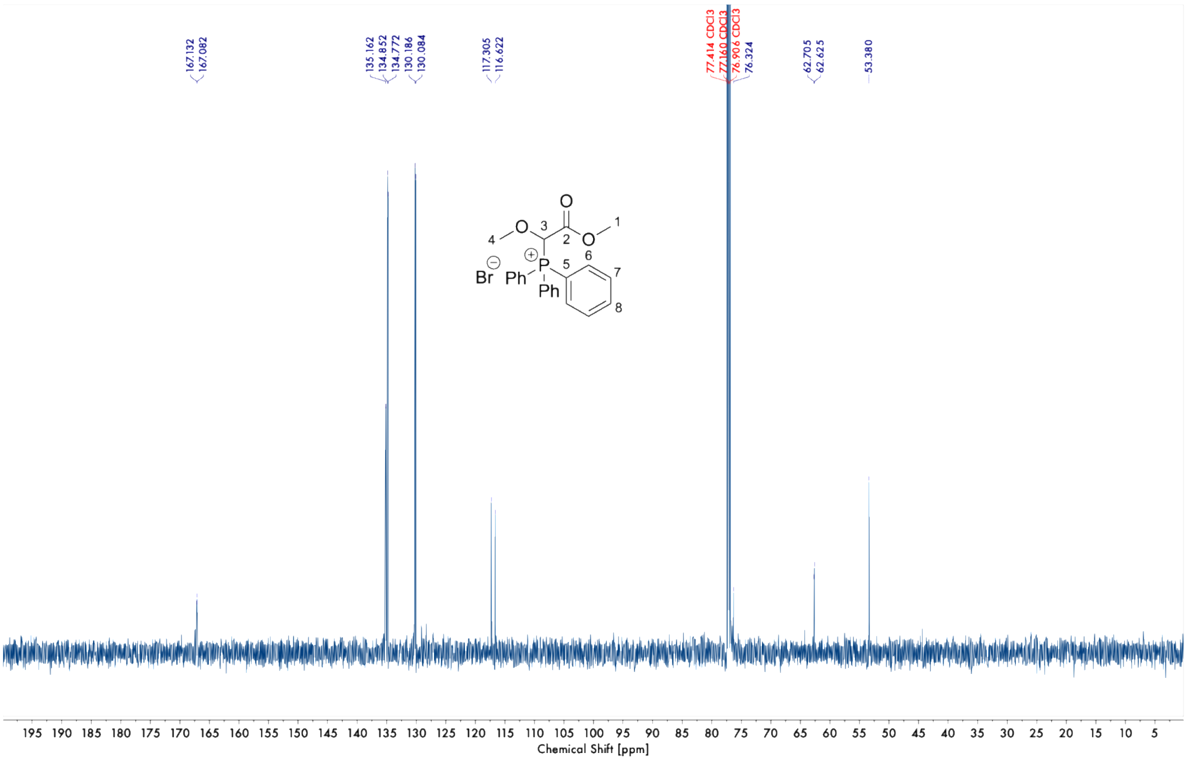

(^{1}H,^{1}H)-COSY (CDCl$_3$, **16**):

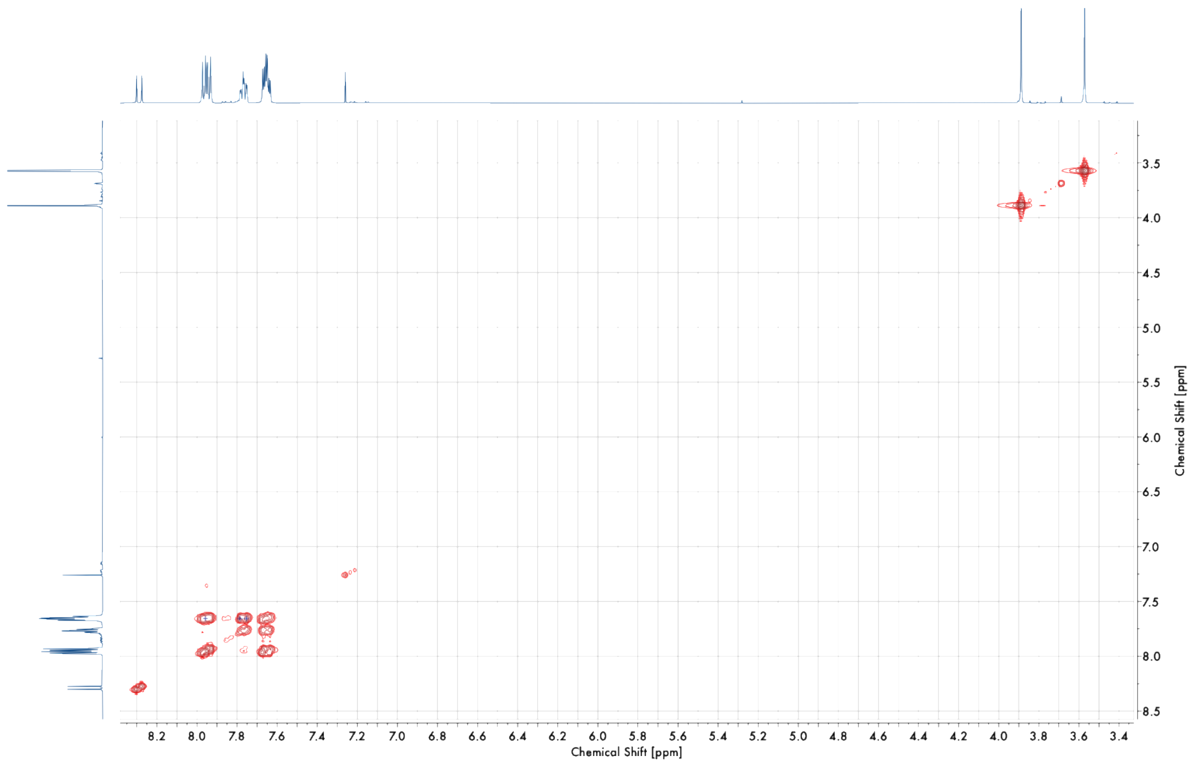

(^{1}H,^{13}C)-HSQC (CDCl$_3$, **16**):

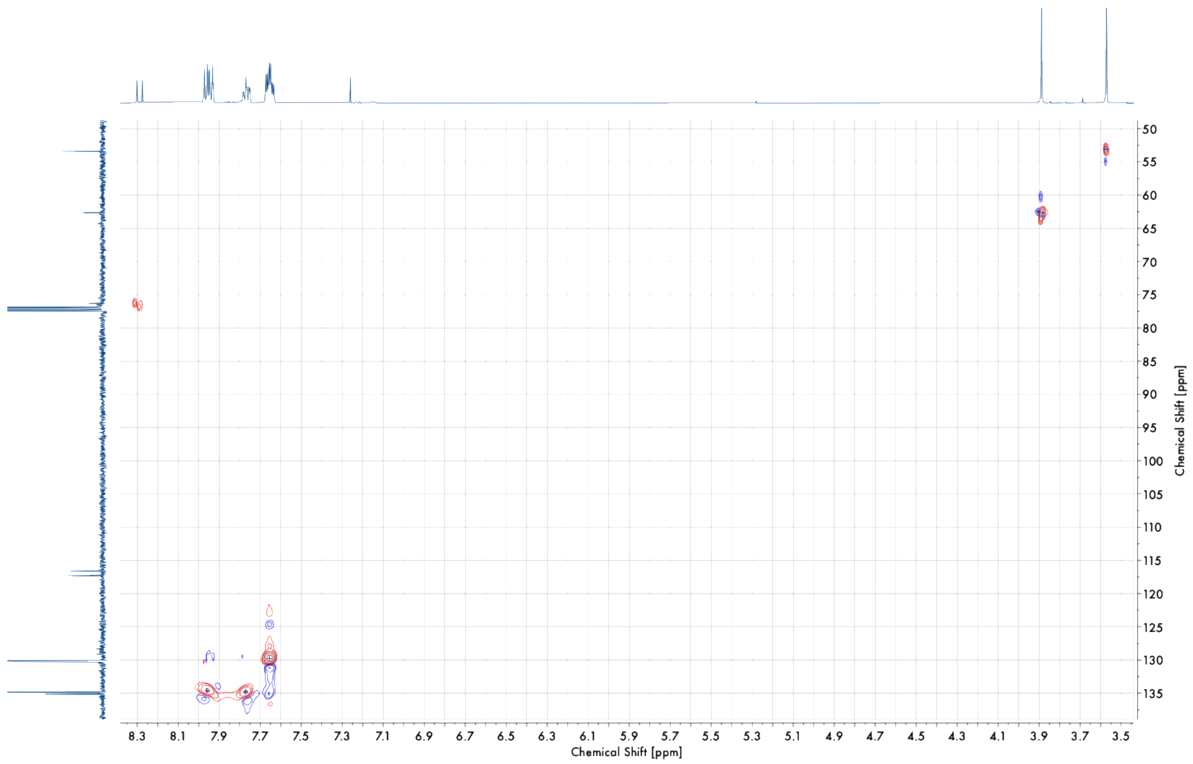

(^{1}H,^{13}C)-HMBC (CDCl$_3$, **16**):

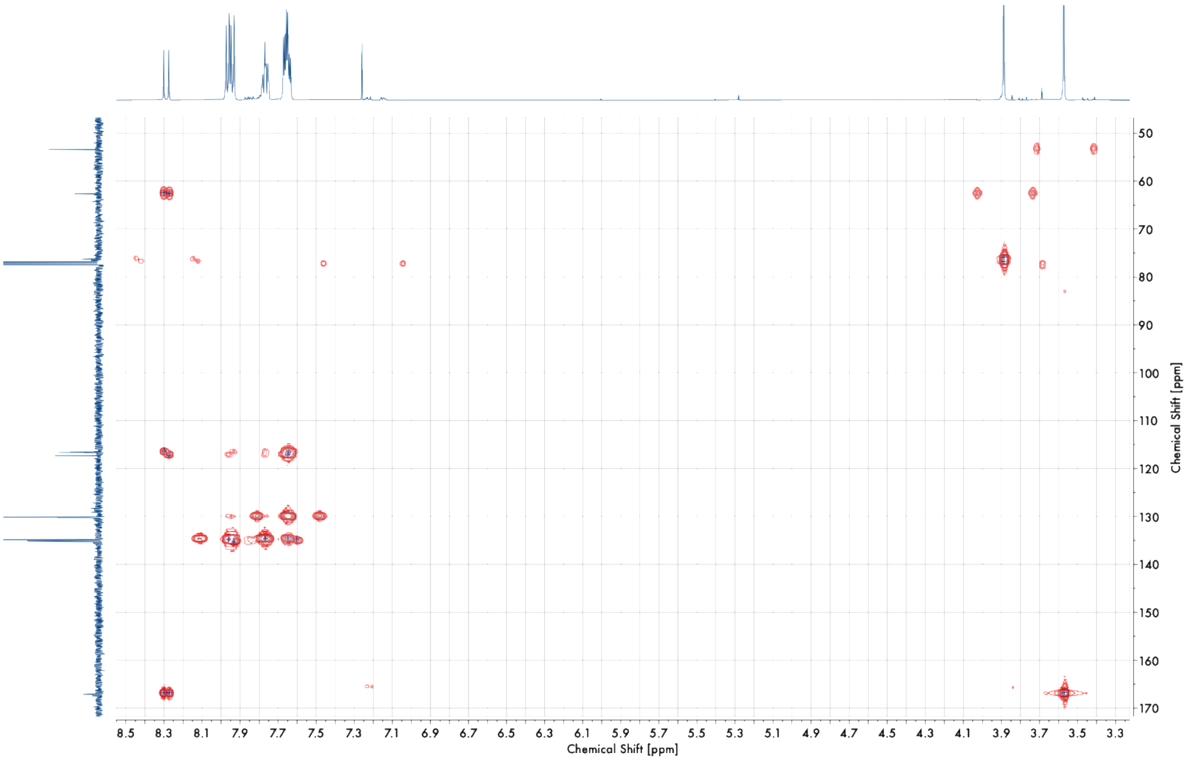

Methyl (*Z*)-3-(4-(allyloxy)-3,5-dichlorophenyl)-2-methoxyacrylate [17]

^{1}H NMR (500 MHz, CDCl$_3$, **17**):

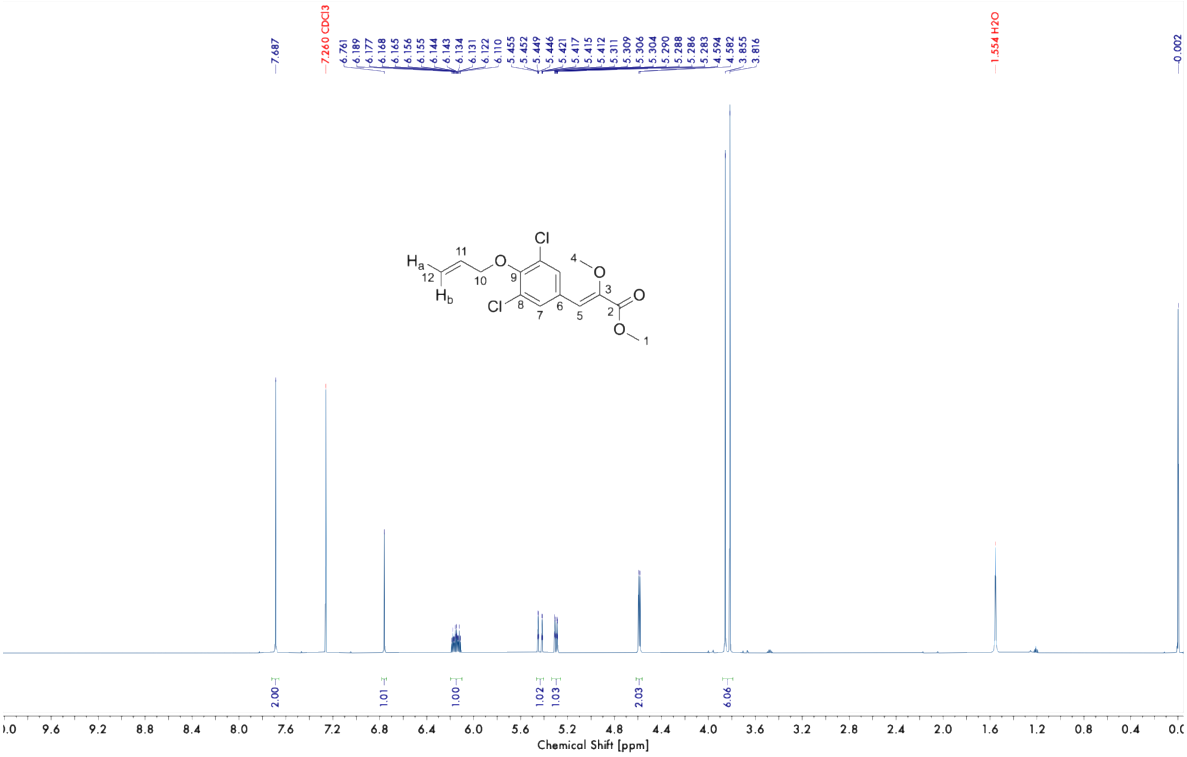

^{13}C NMR (126 MHz, $CDCl_3$, **17**):

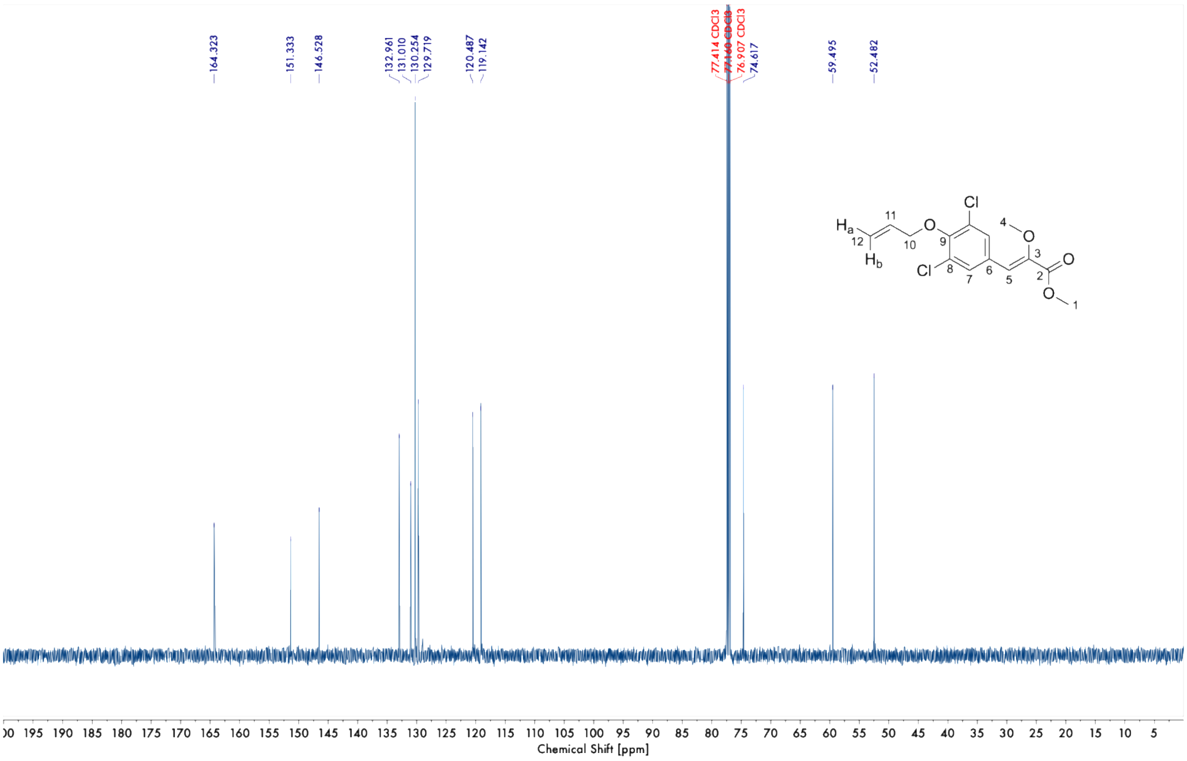

(^{1}H,^{1}H)-COSY ($CDCl_3$, **17**):

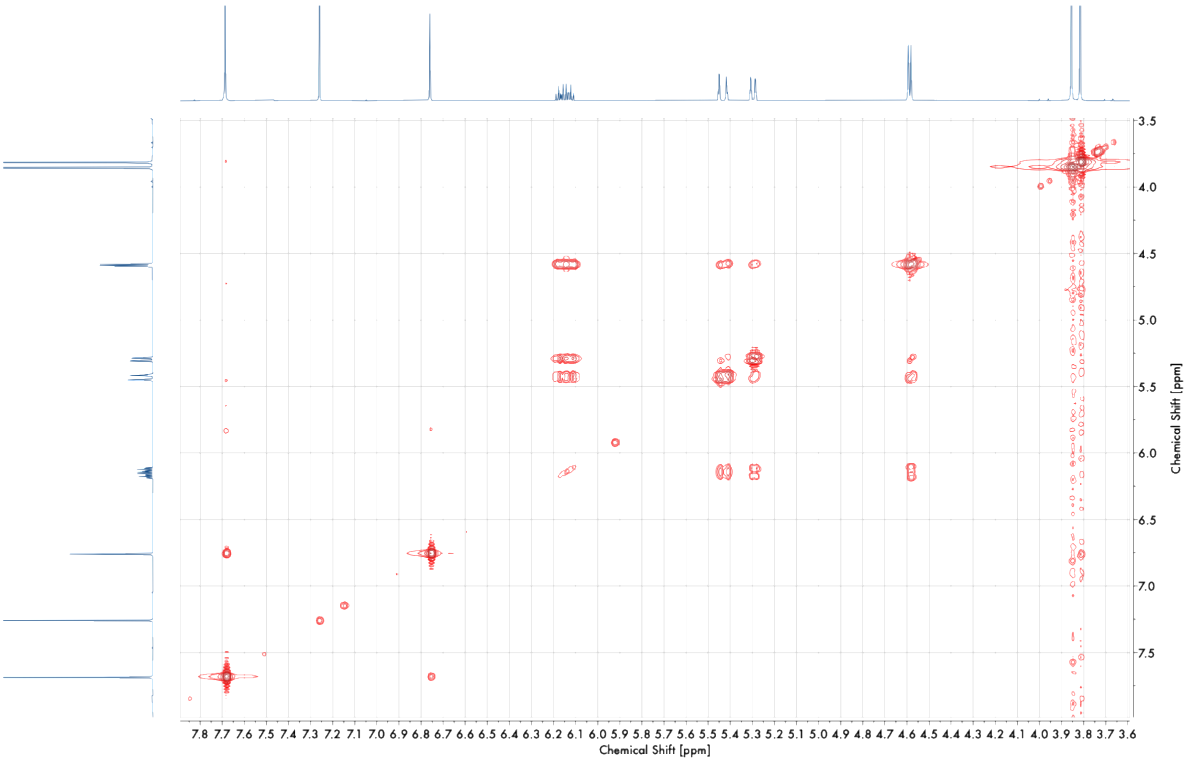

(^{1}H,^{13}C)-HSQC (CDCl$_3$, **17**):

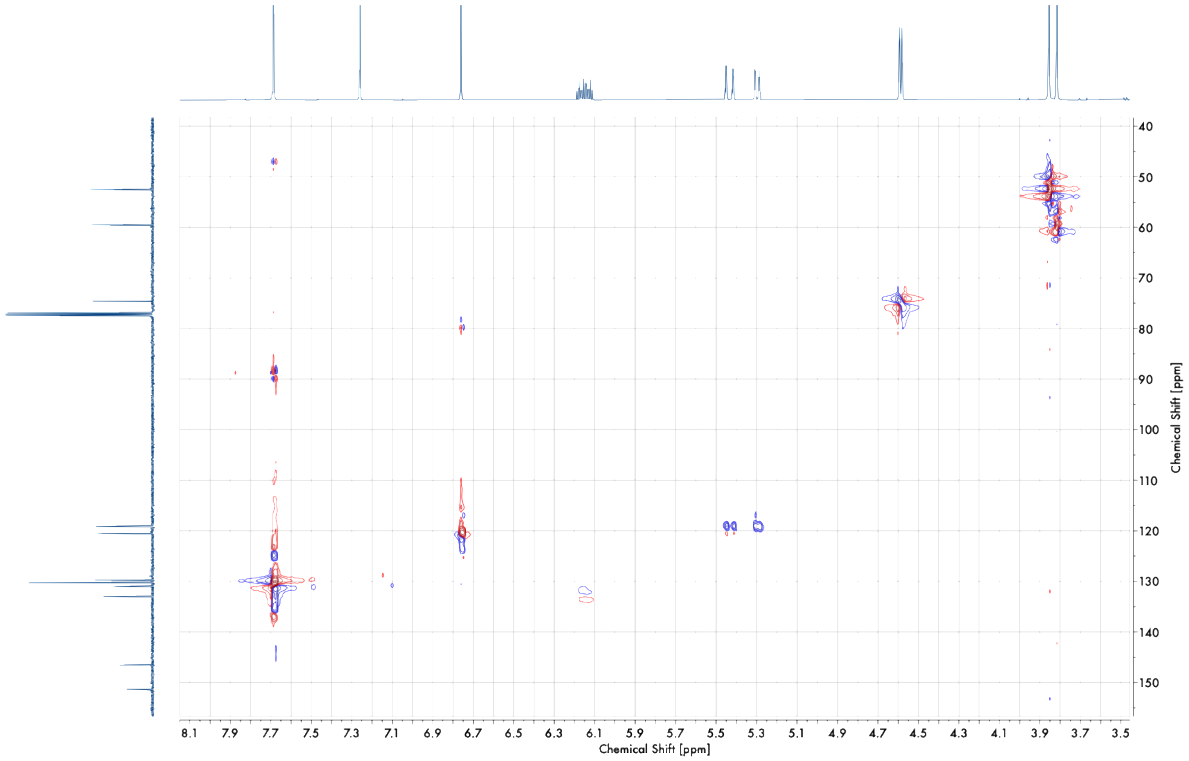

(^{1}H,^{13}C)-HMBC (CDCl$_3$, **17**):

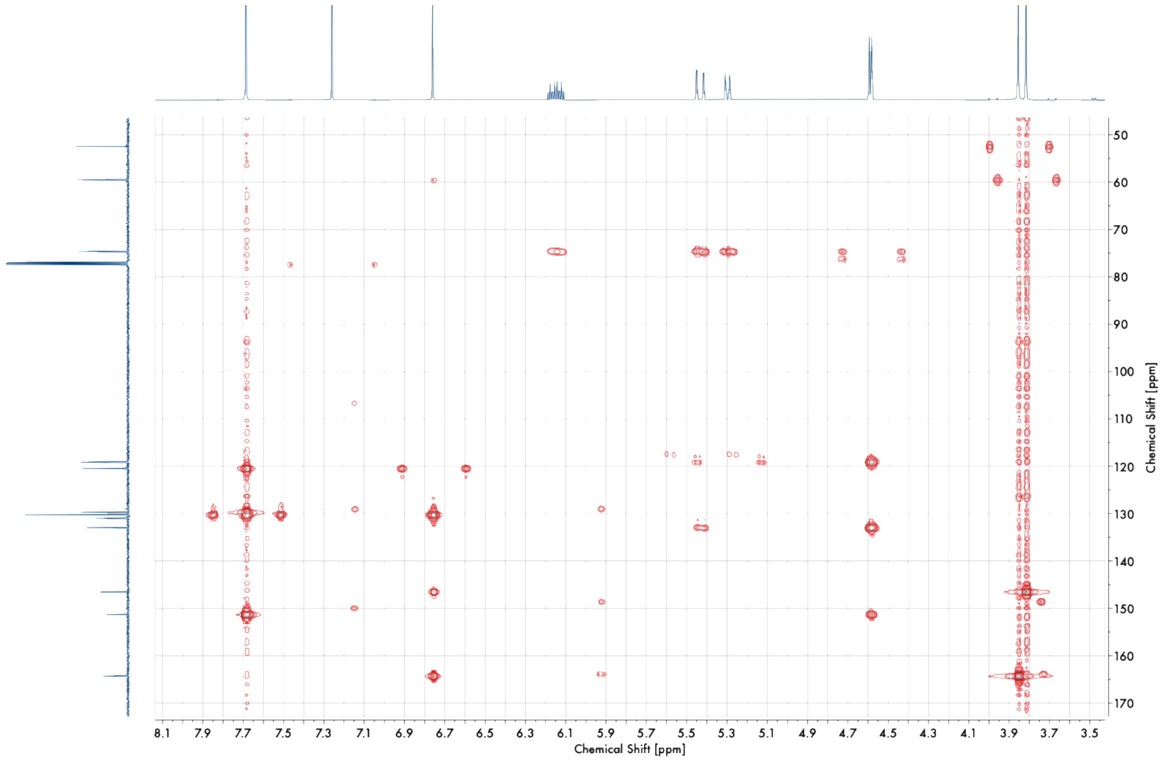

(^{1}H,^{1}H)-NOESY (CDCl$_3$, **17**):

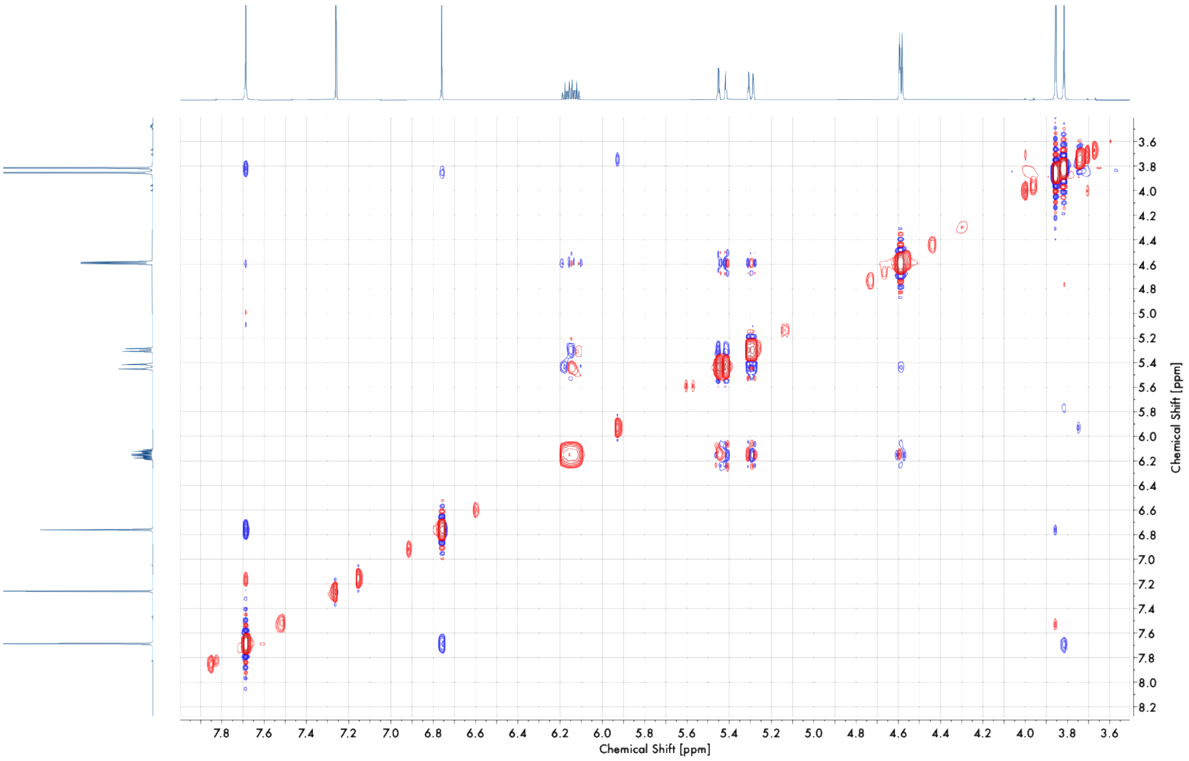

(*Z*)-3-(4-(Allyloxy)-3,5-dichlorophenyl)-2-methoxyacrylic acid [18]

^{1}H NMR (500 MHz, DMSO-d_6, **18**):

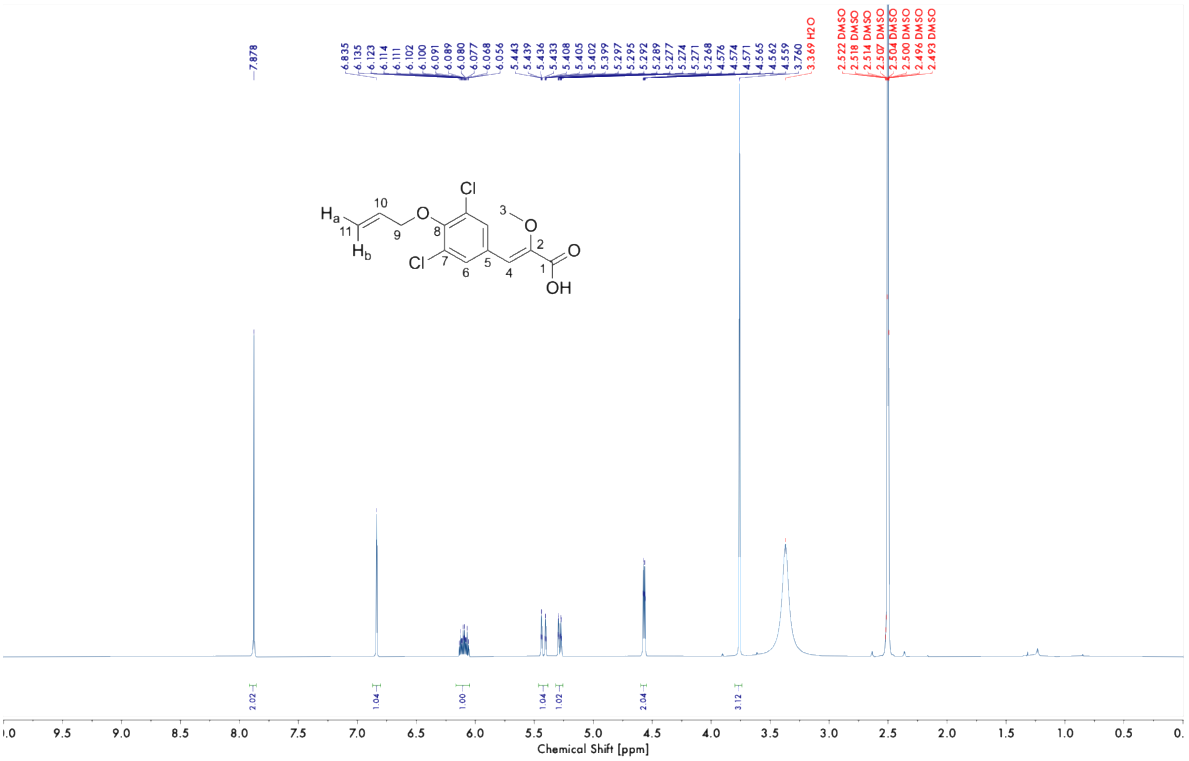

^{13}C NMR (126 MHz, DMSO-d_6, **18**):

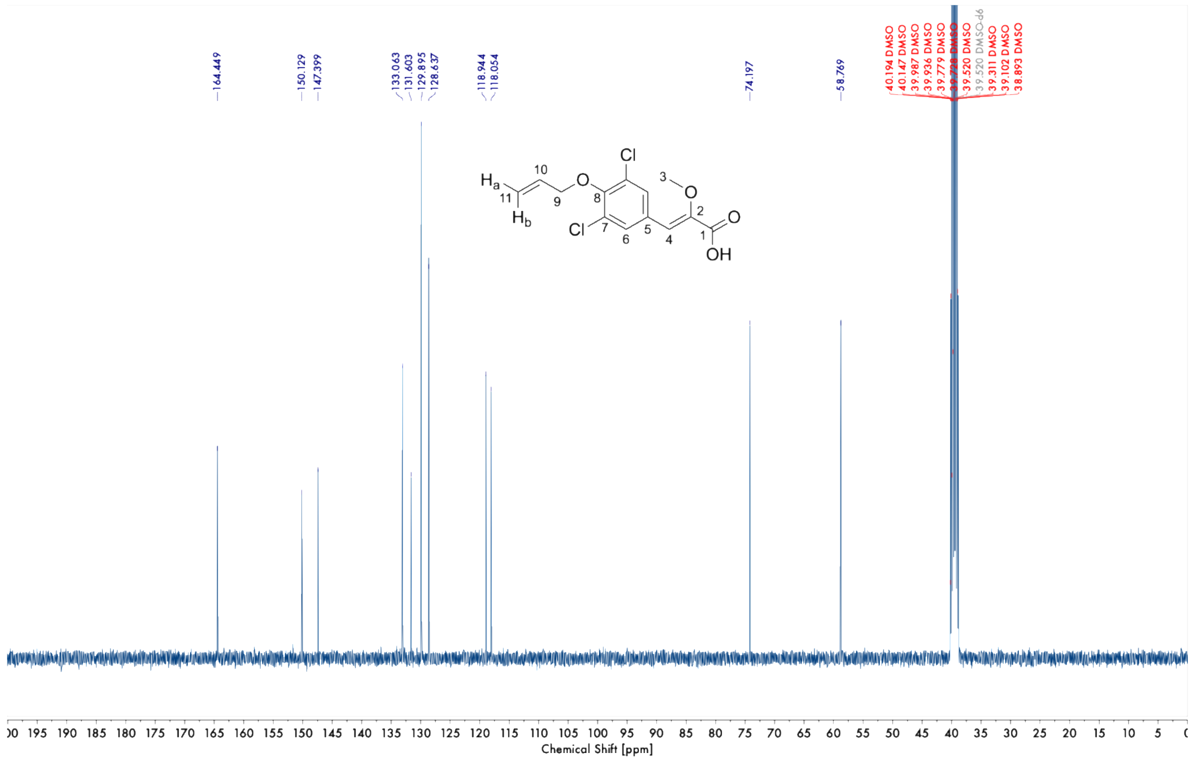

(^{1}H,^{1}H)-COSY (DMSO-d_6, **18**):

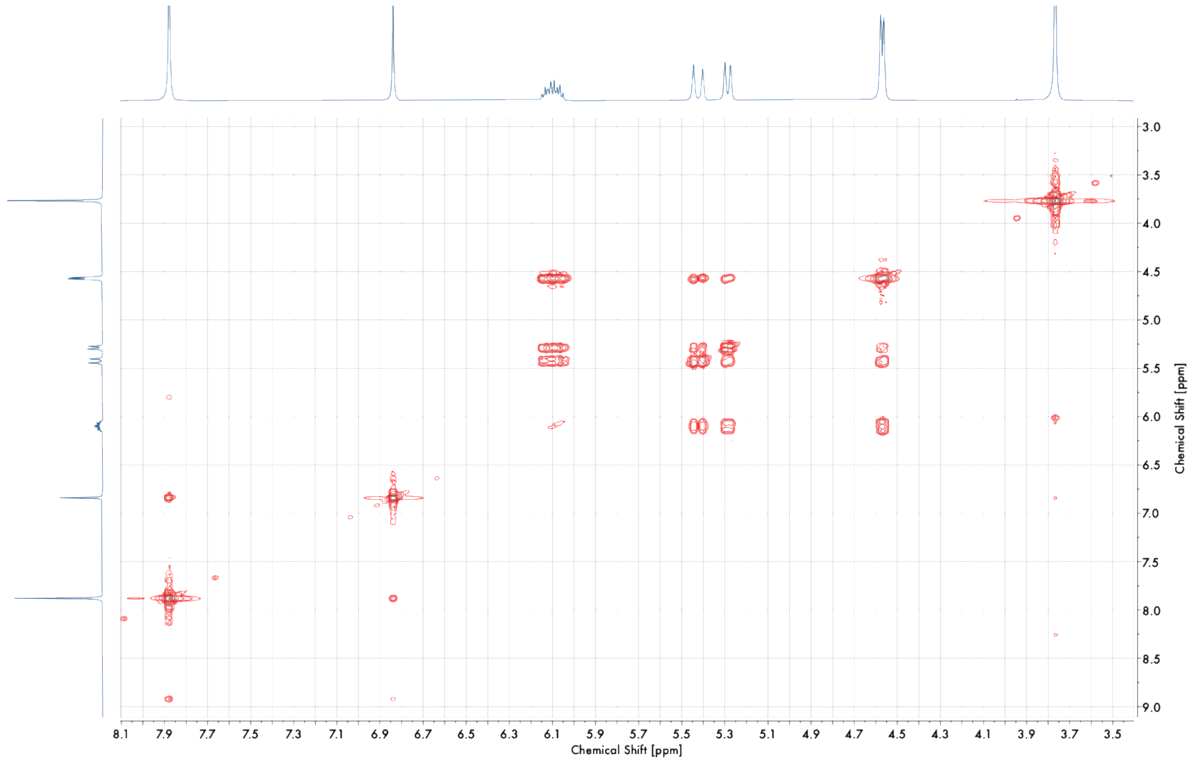

(^{1}H,^{13}C)-HSQC (DMSO-d_6, **18**):

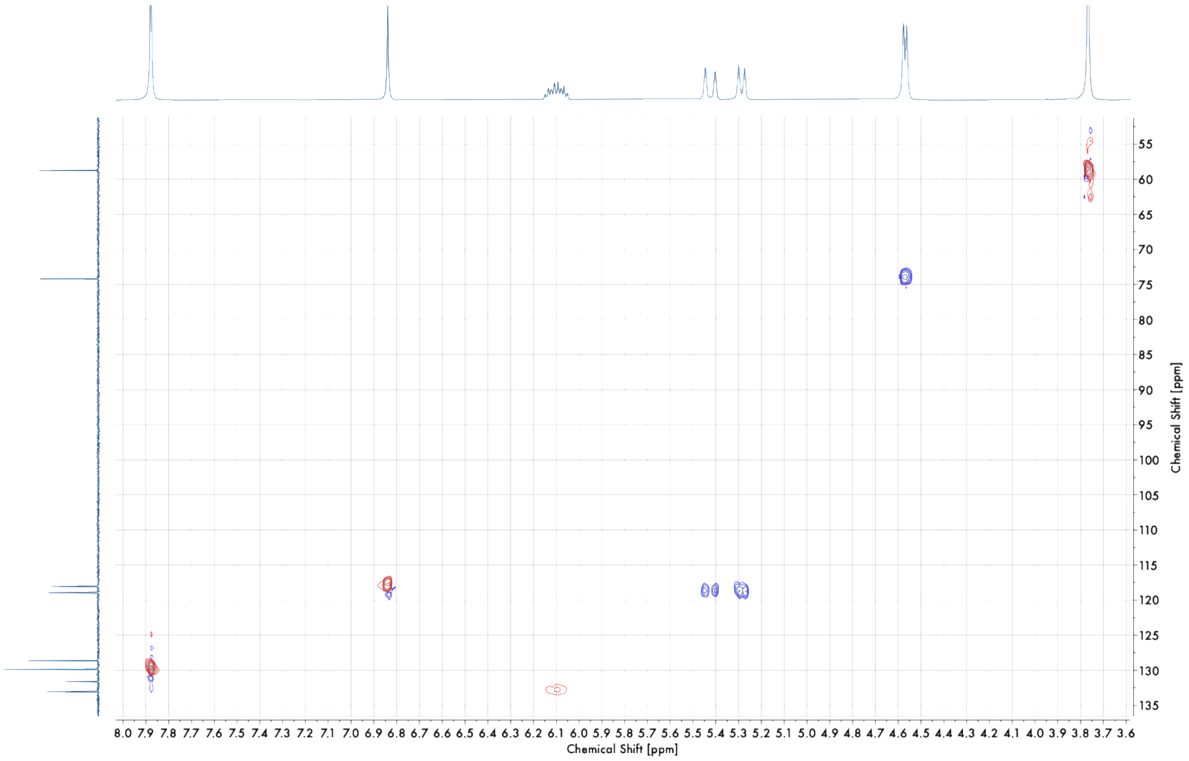

(^{1}H,^{13}C)-HMBC (DMSO-d_6, **18**):

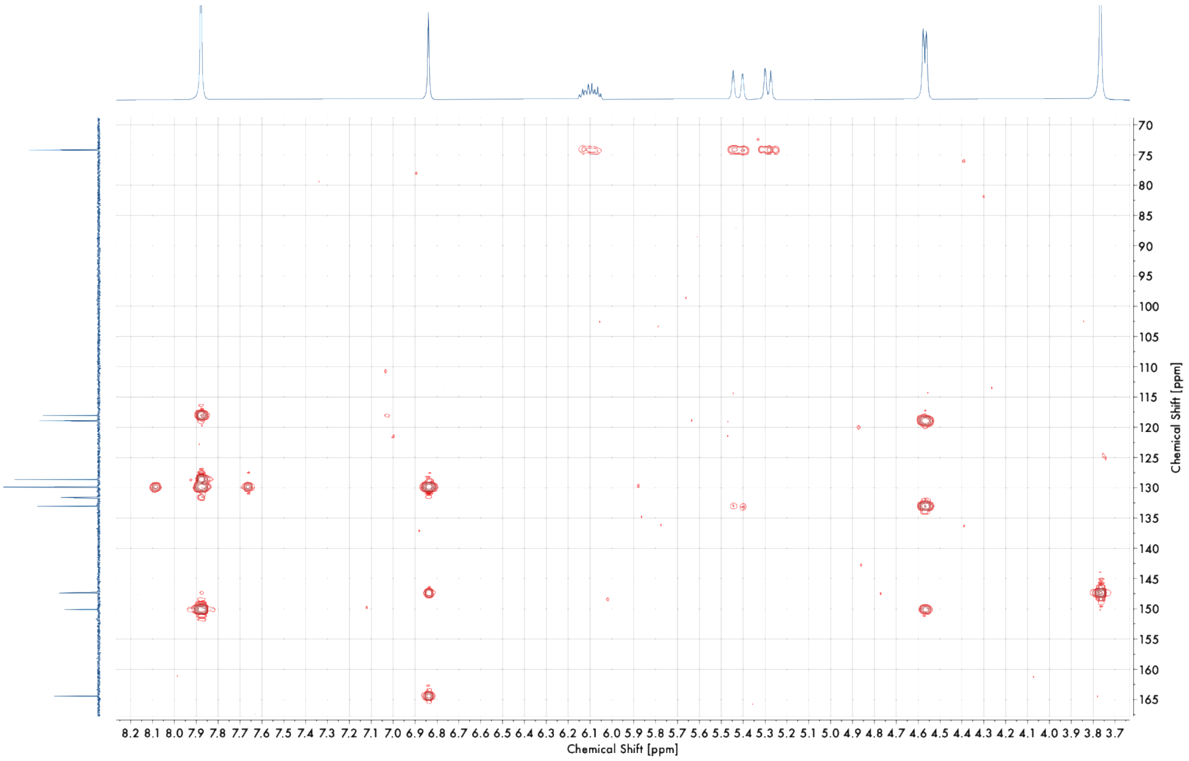

***tert*-Butyl *N*-cyanomethyl-L-valinate [SI-4]**

^{1}H NMR (500 MHz, $CDCl_3$, **SI-4**):

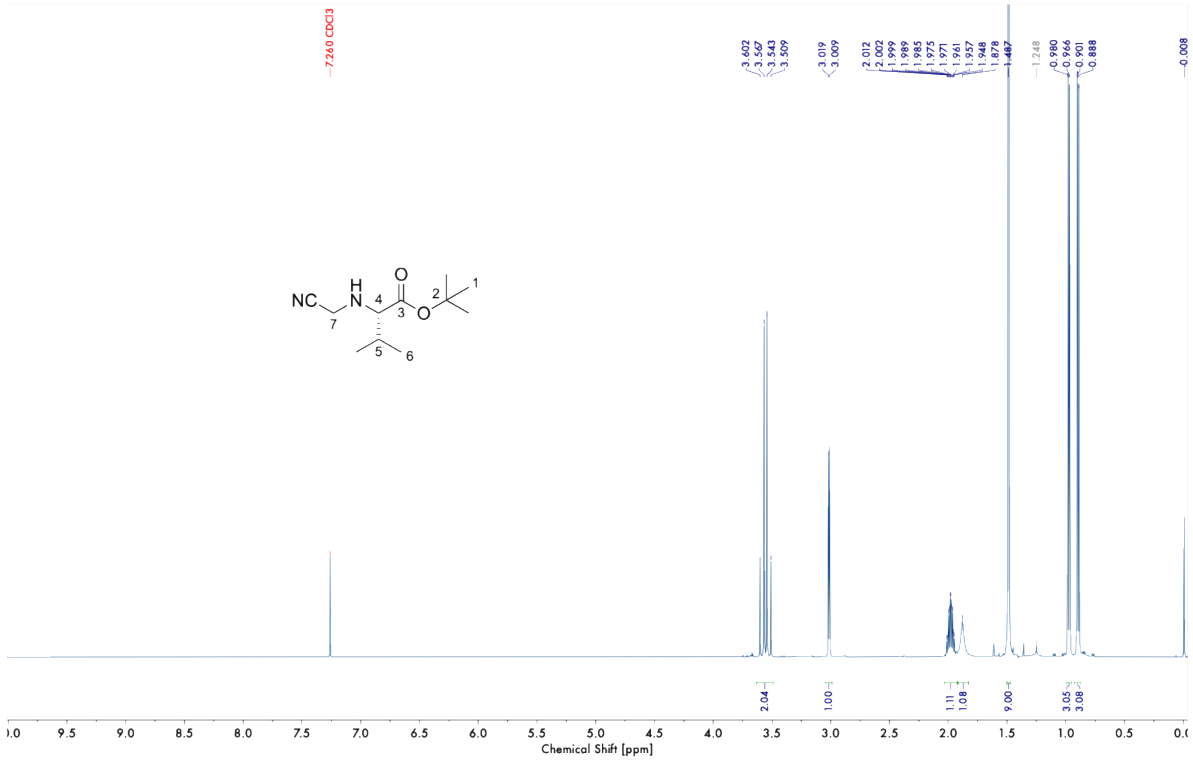

^{13}C NMR (126 MHz, $CDCl_3$, **SI-4**):

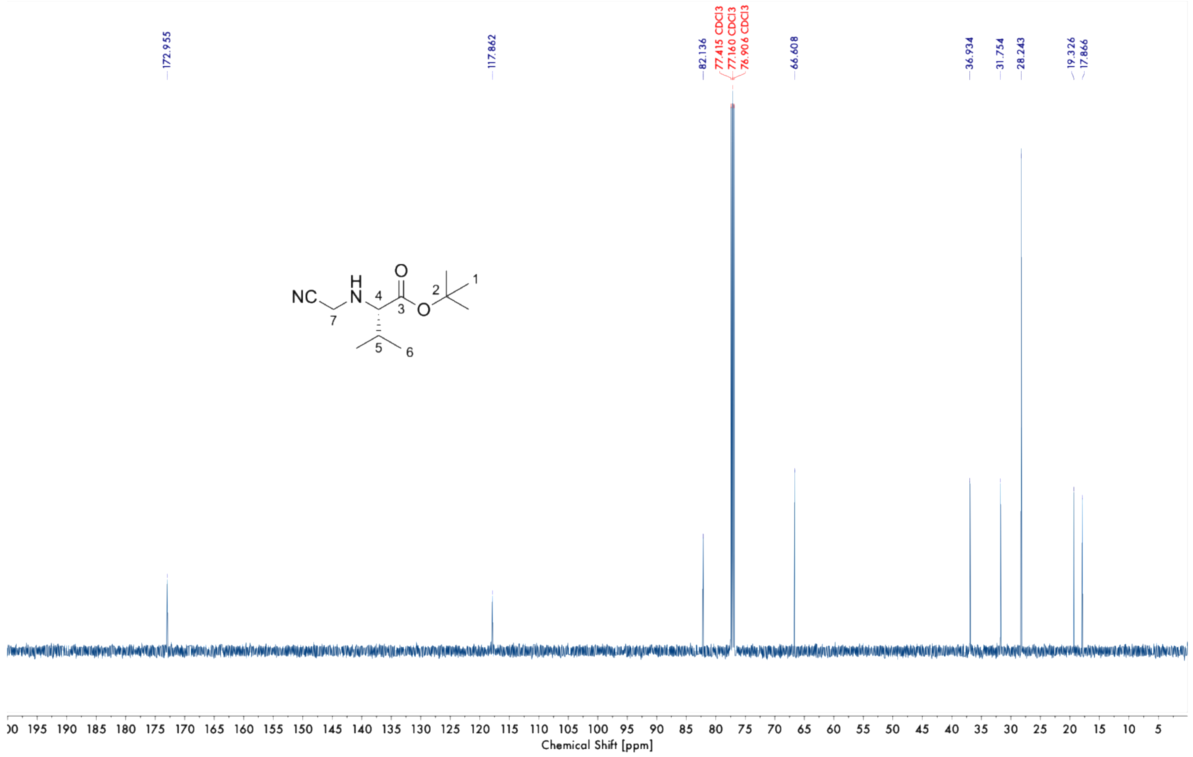

(^{1}H,^{1}H)-COSY (CDCl$_3$, **SI-4**):

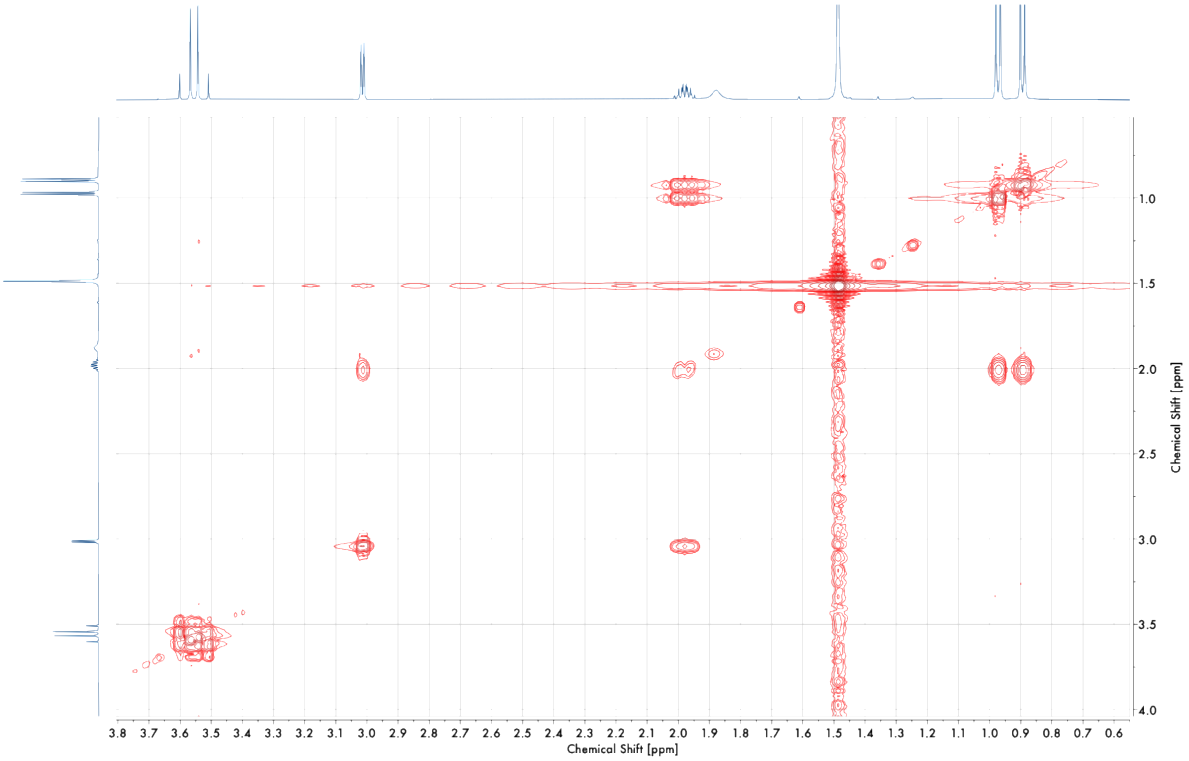

(^{1}H,^{13}C)-HSQC (CDCl$_3$, **SI-4**):

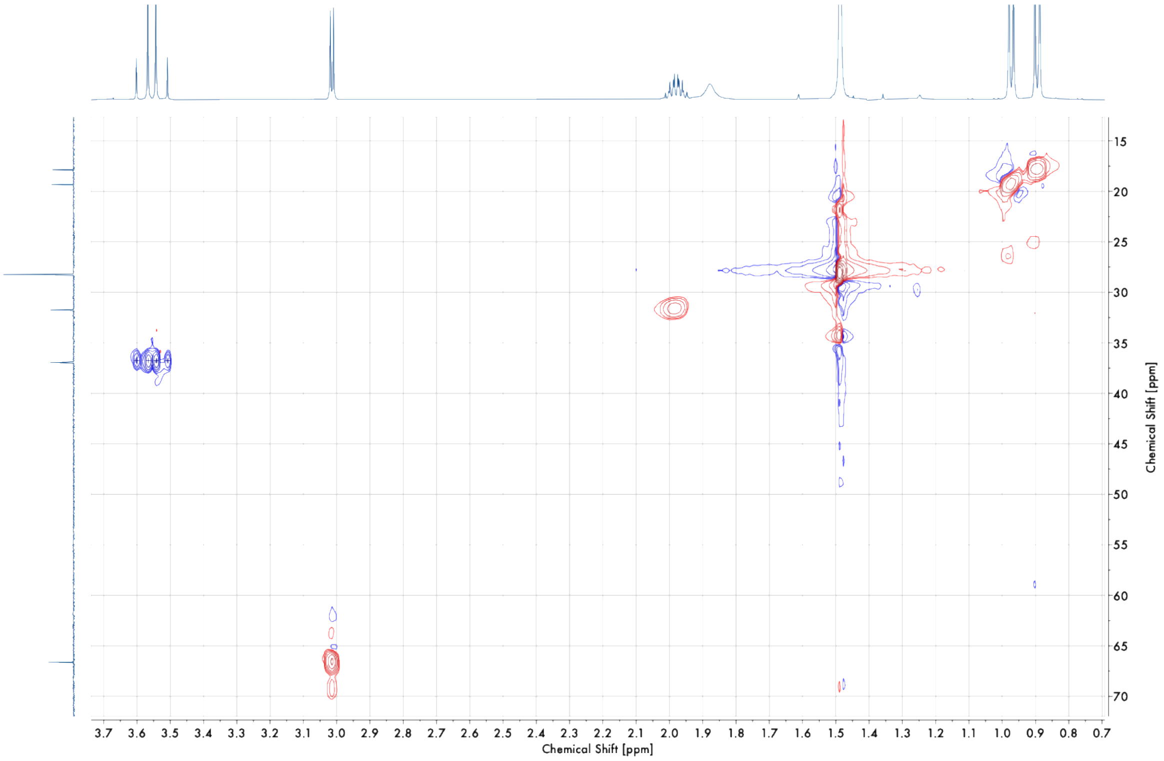

(^{1}H,^{13}C)-HMBC ($CDCl_3$, **SI-4**):

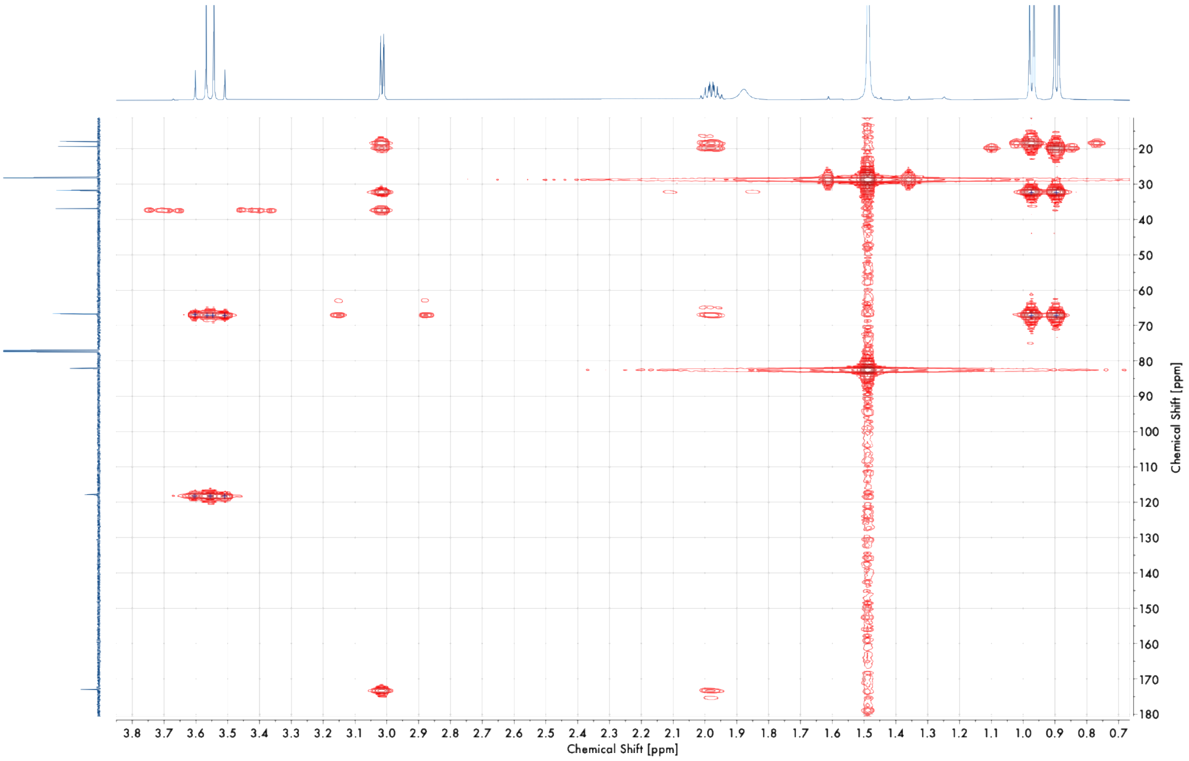

tert-Butyl *N*-hydroxy-L-valinate [19]

^{1}H NMR (400 MHz, $CDCl_3$):

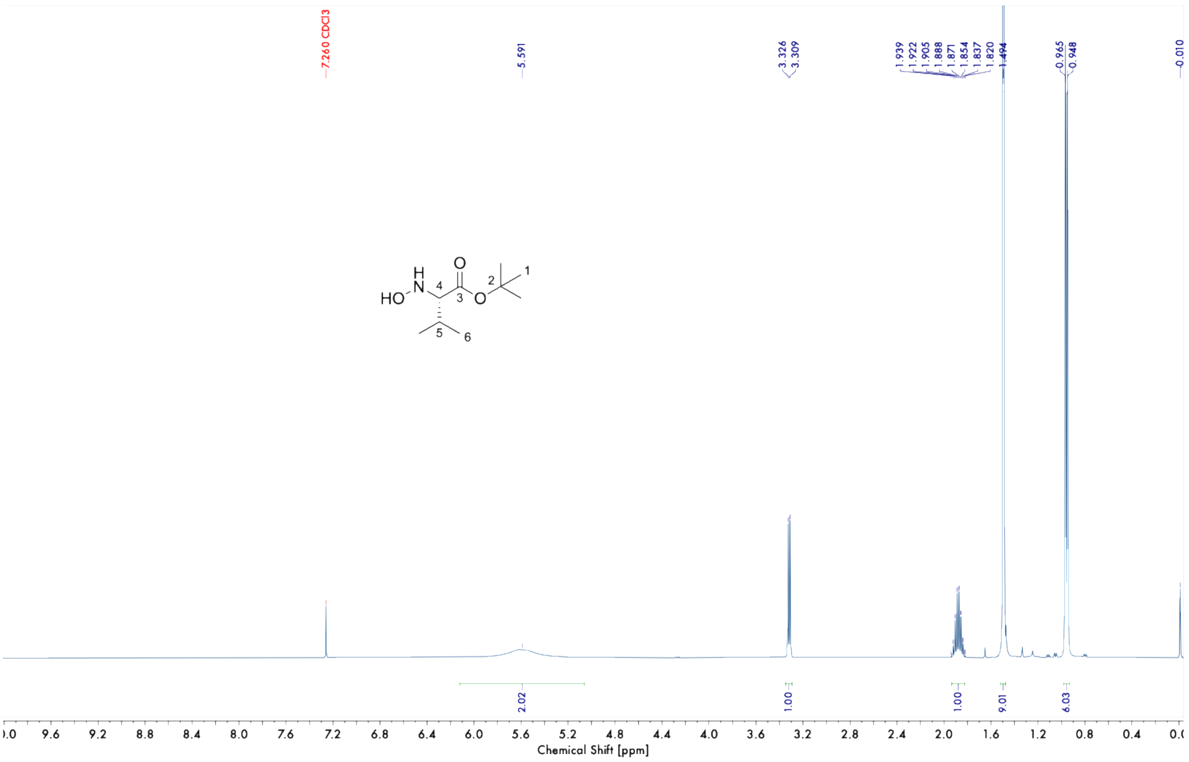

^{13}C NMR (126 MHz, $CDCl_3$):

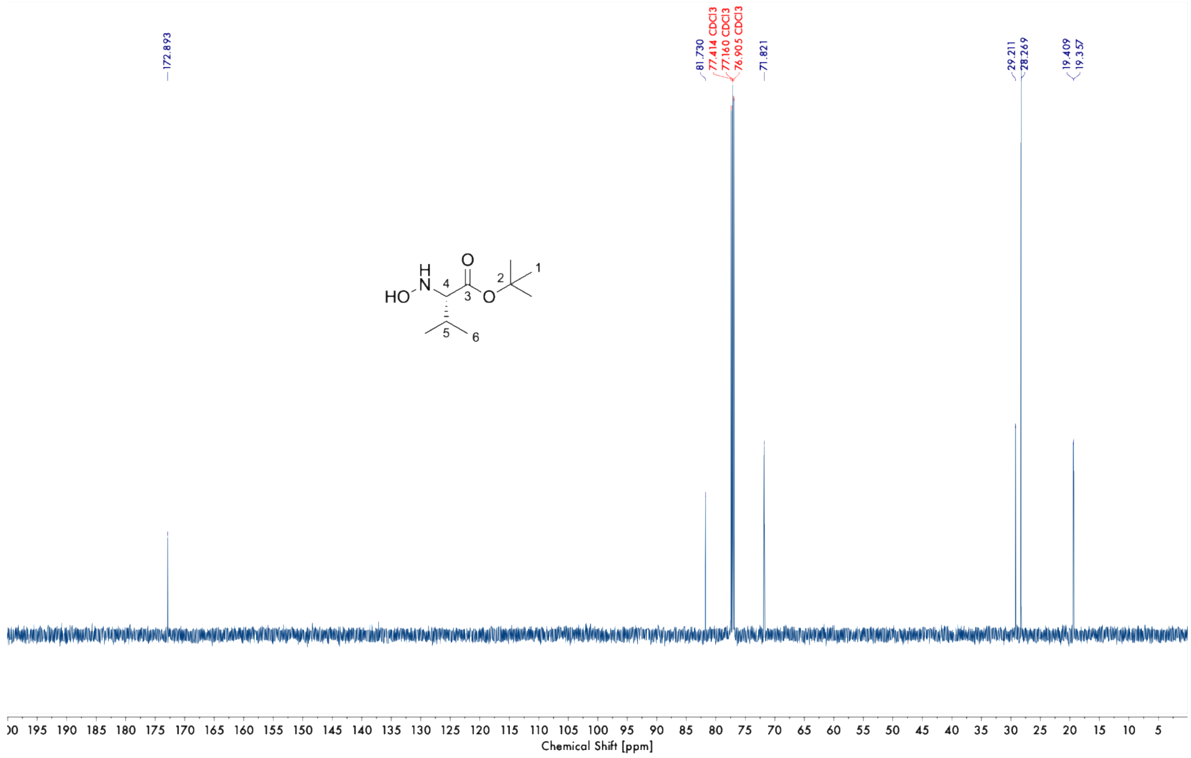

(^1H,^1H)-COSY ($CDCl_3$):

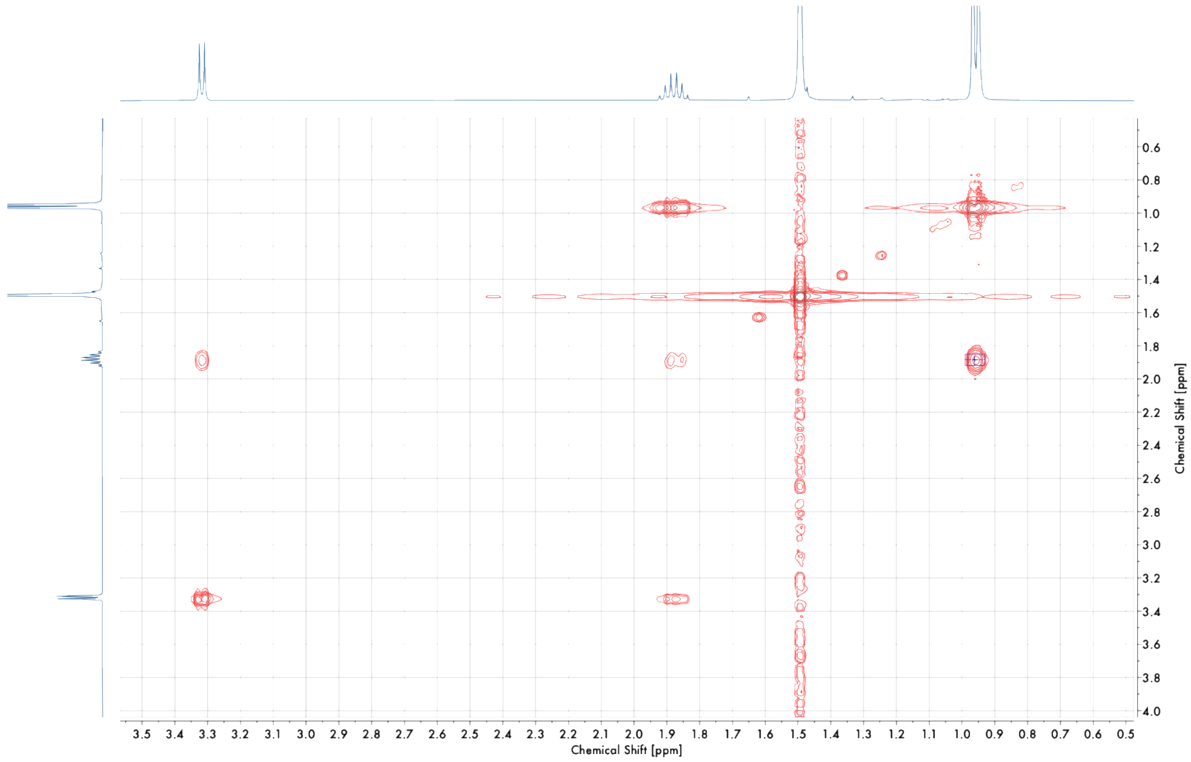

(^{1}H,^{13}C)-HSQC ($CDCl_3$):

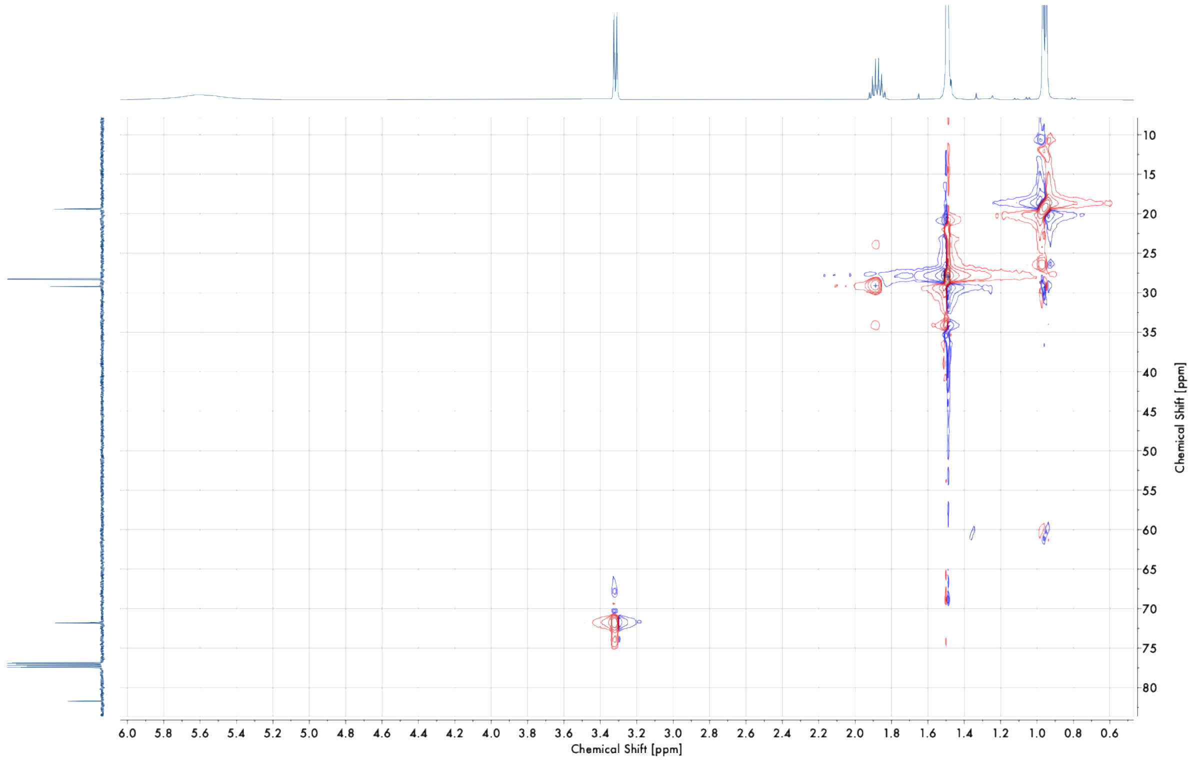

(^{1}H,^{13}C)-HMBC ($CDCl_3$):

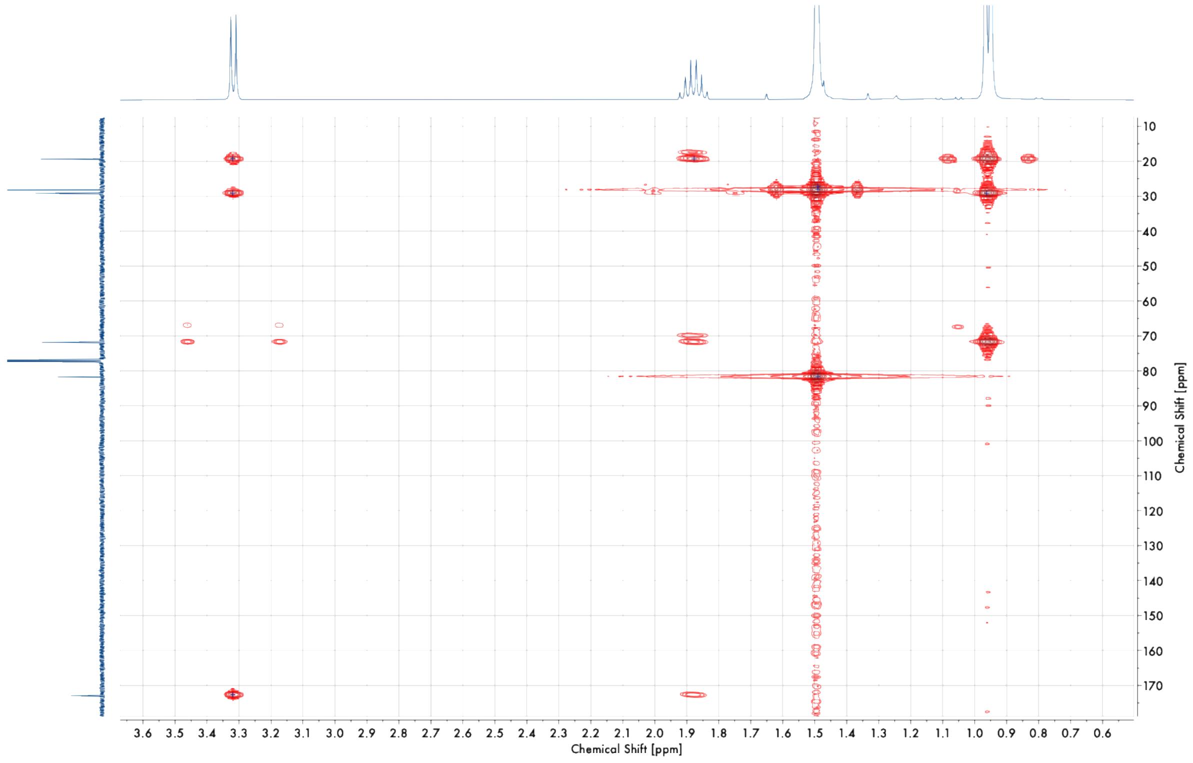

Fmoc-Leu-HyVal-O*t*-Bu [20]

^{1}H NMR (500 MHz, $CDCl_3$, **20**):

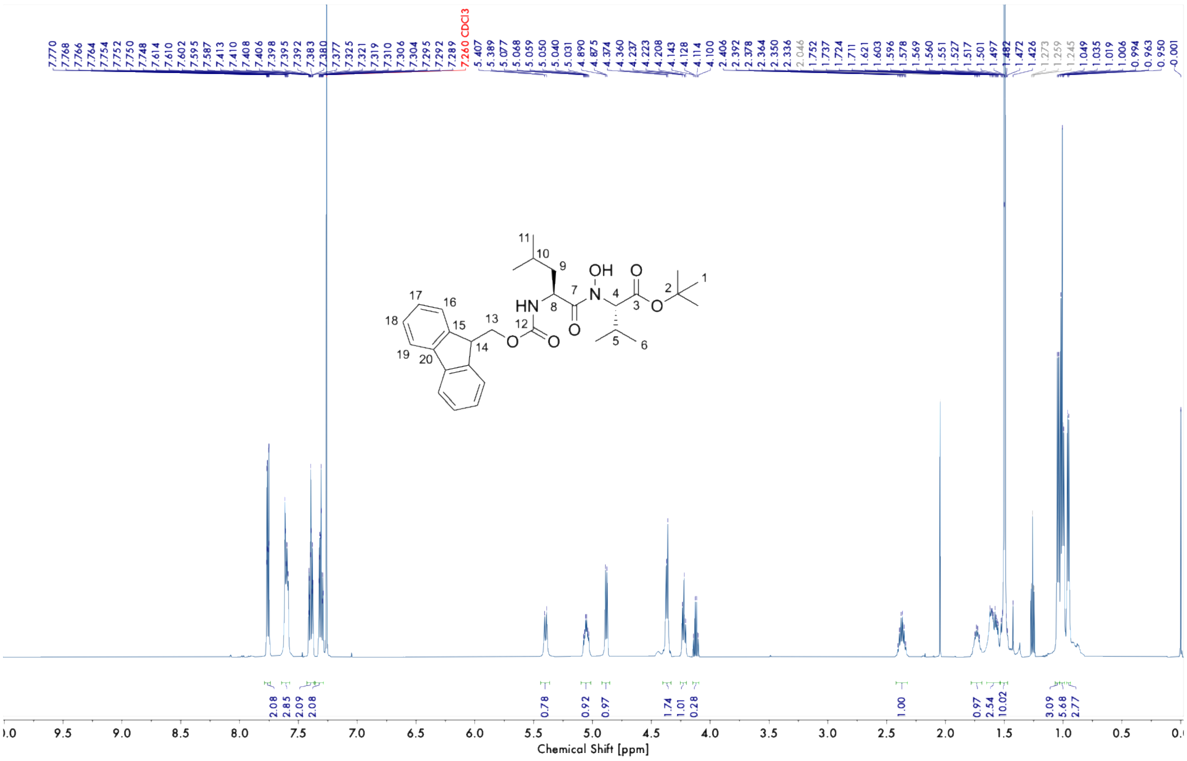

^{13}C NMR (101 MHz, $CDCl_3$, **20**):

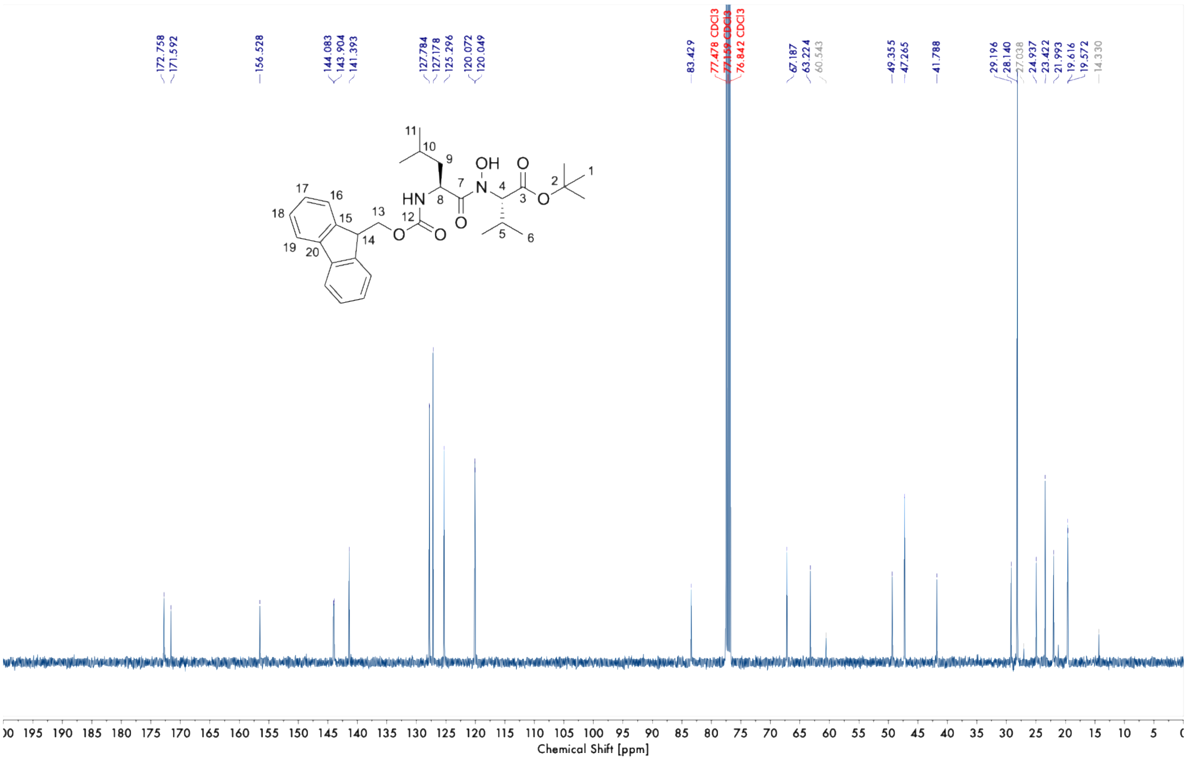

(^{1}H,^{1}H)-COSY ($CDCl_3$, **20**):

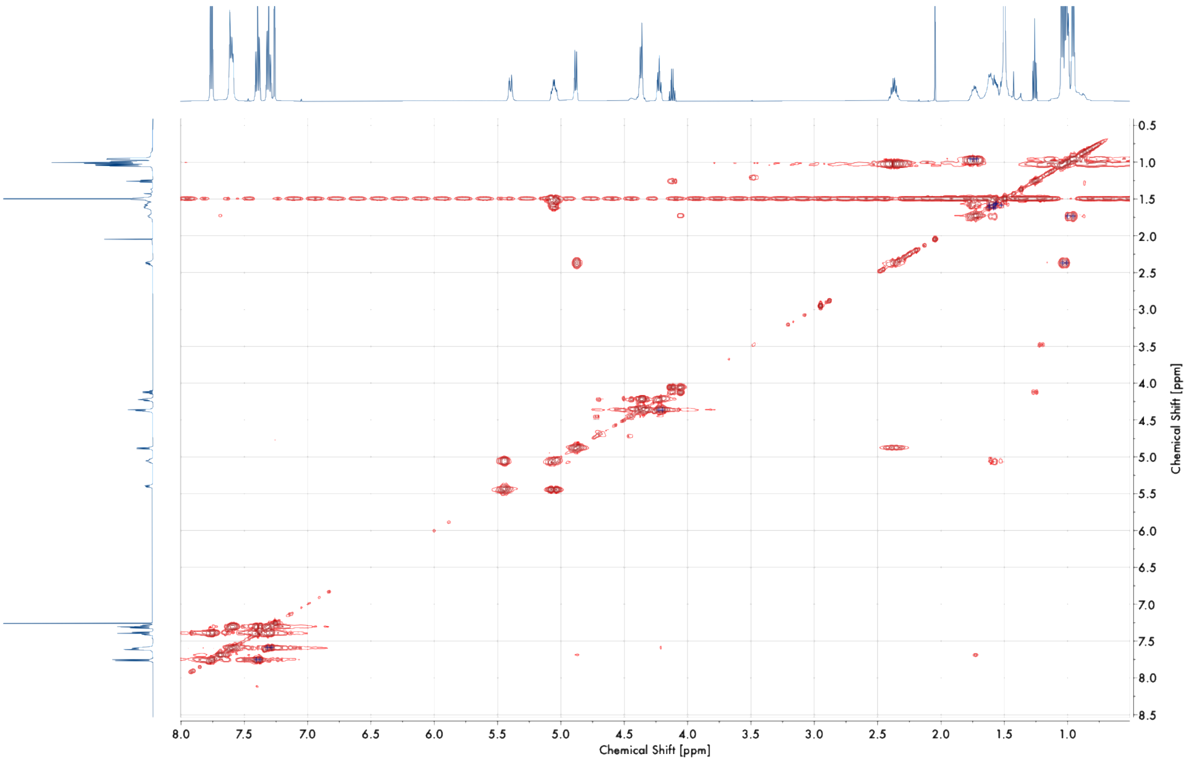

(^{1}H,^{13}C)-HSQC ($CDCl_3$, **20**):

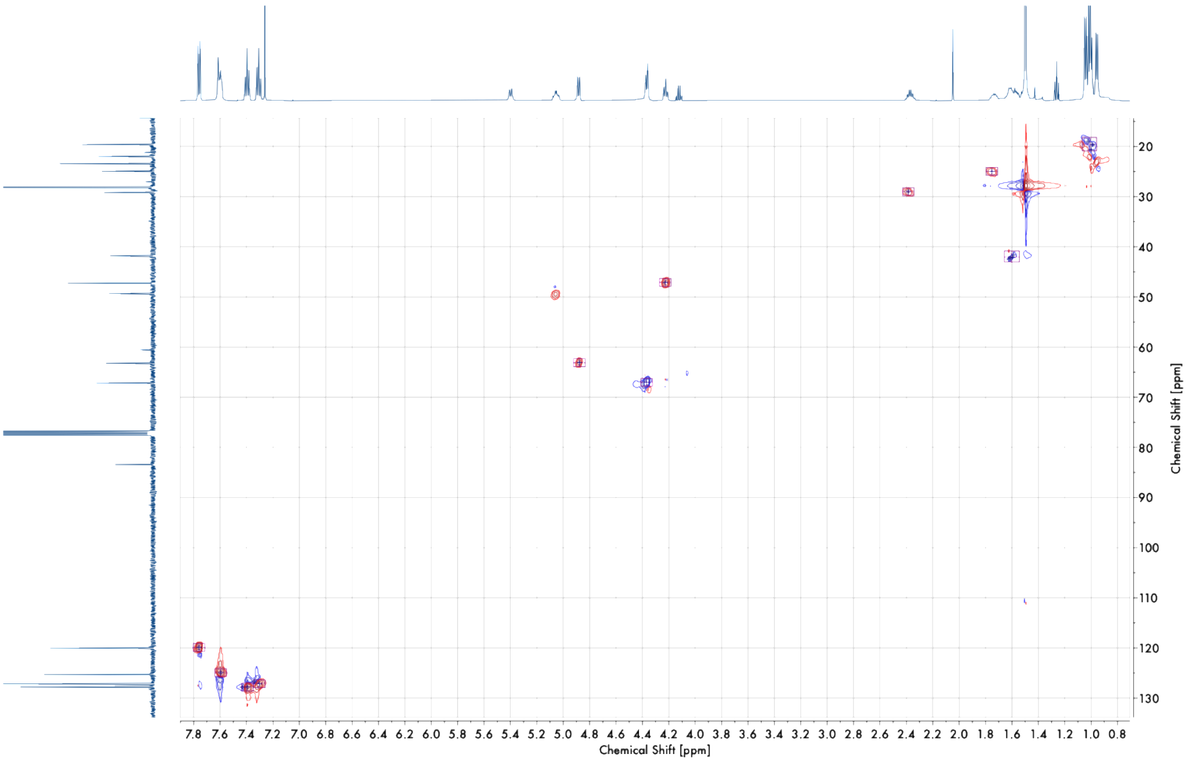

(^{1}H,^{13}C)-HMBC (CDCl$_3$, **20**):

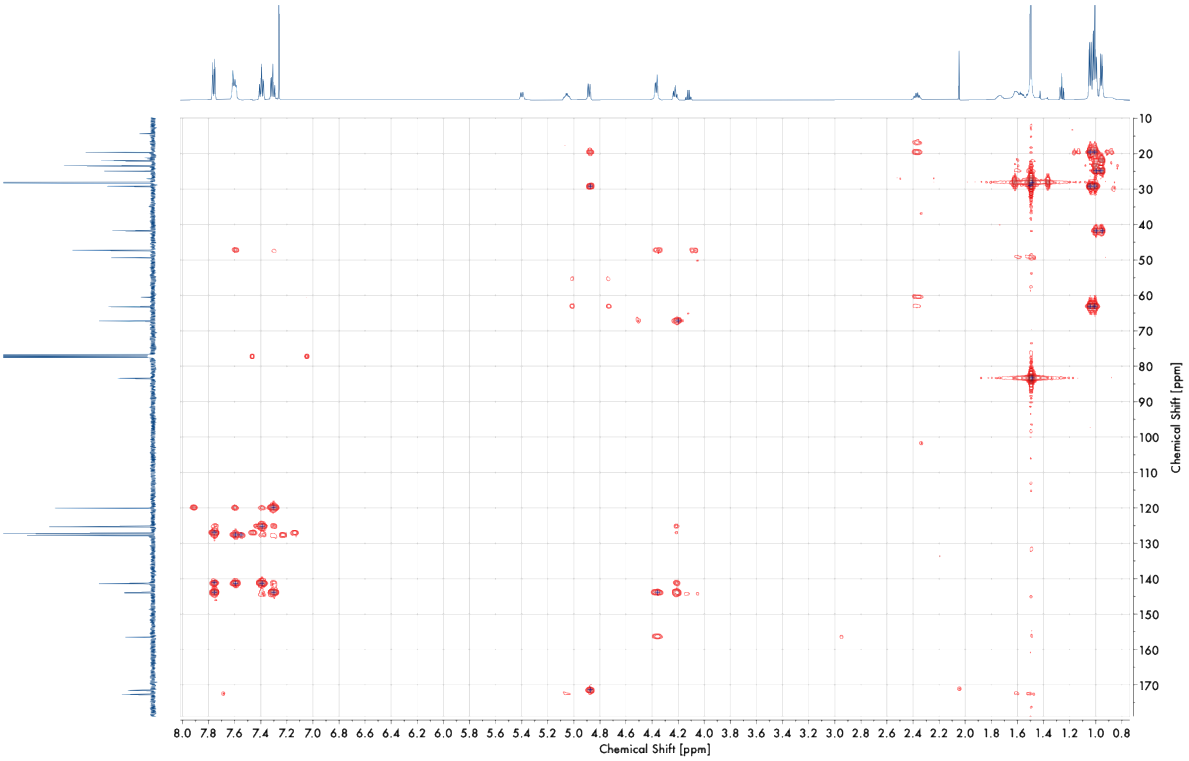

Fmoc-Leu-HyVal(*O*Allyl)-O*t*-Bu [SI-5]

^{1}H NMR (500 MHz, CDCl$_3$, **SI-5**):

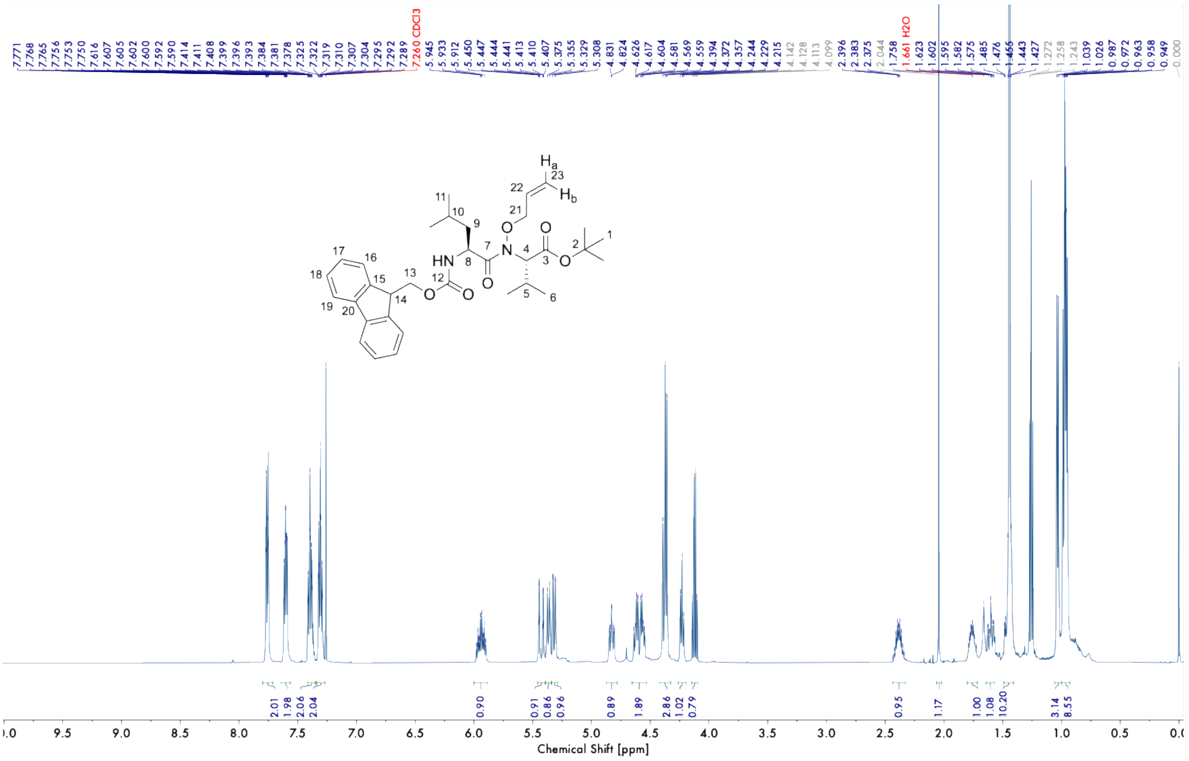

^{13}C NMR (126 MHz, $CDCl_3$, **SI-5**):

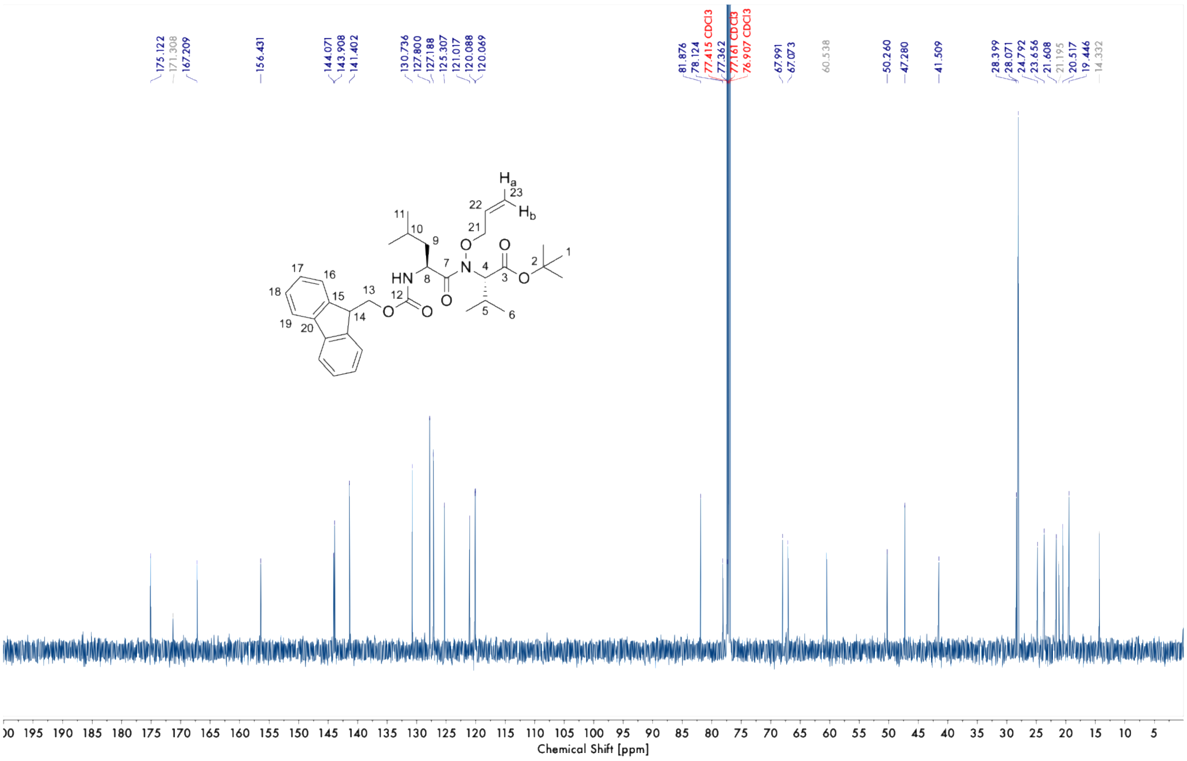

(^{1}H,^{1}H)-COSY ($CDCl_3$, **SI-5**):

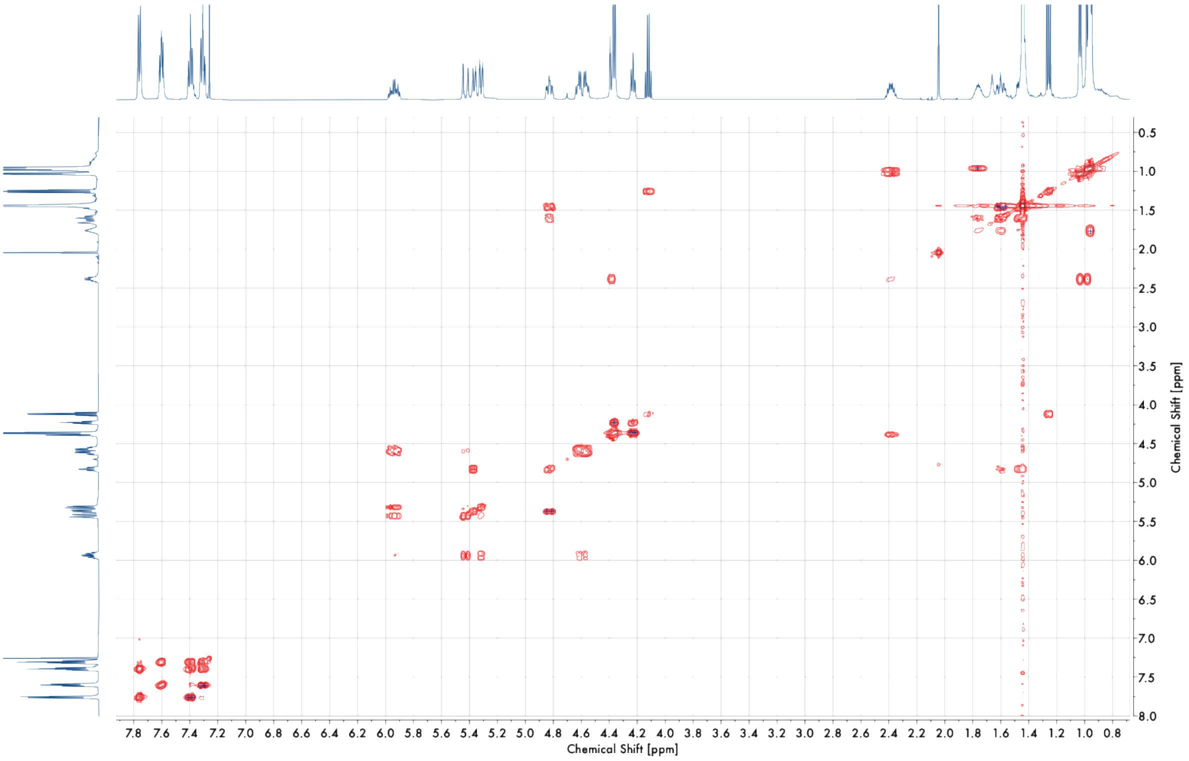

(^{1}H,^{13}C)-HSQC (CDCl$_3$, **SI-5**):

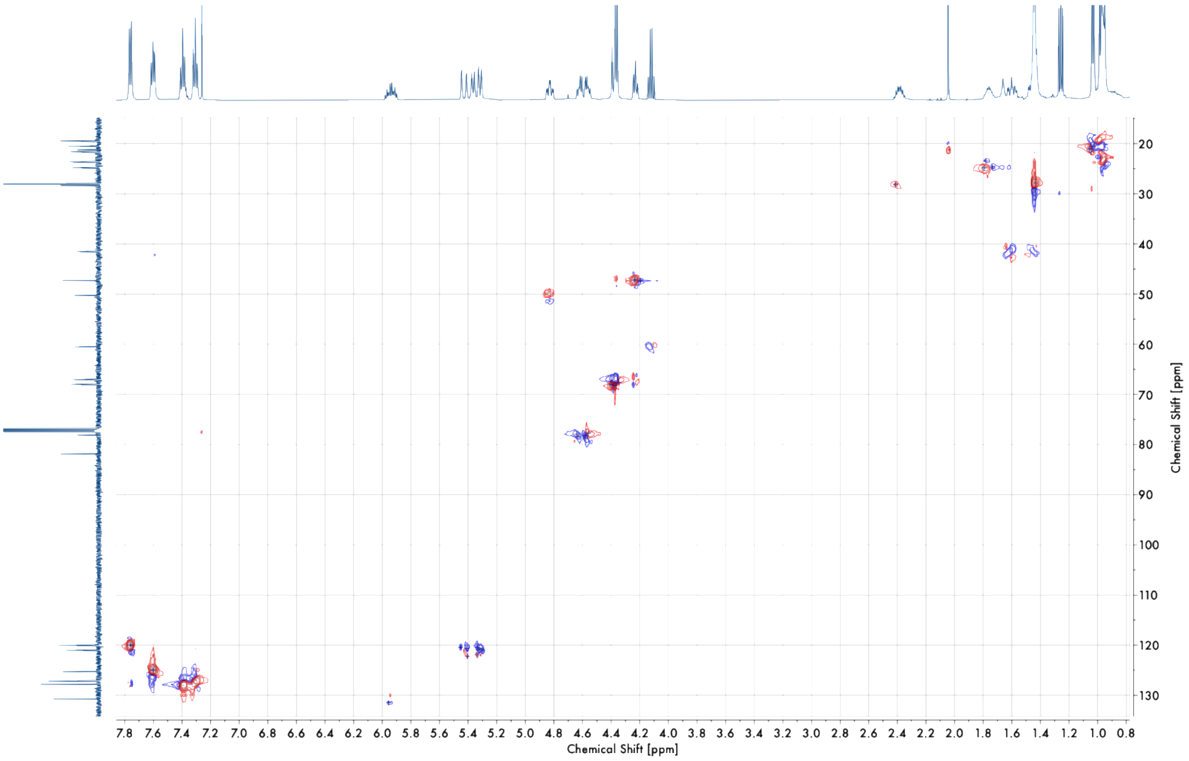

(^{1}H,^{13}C)-HMBC (CDCl$_3$, **SI-5**):

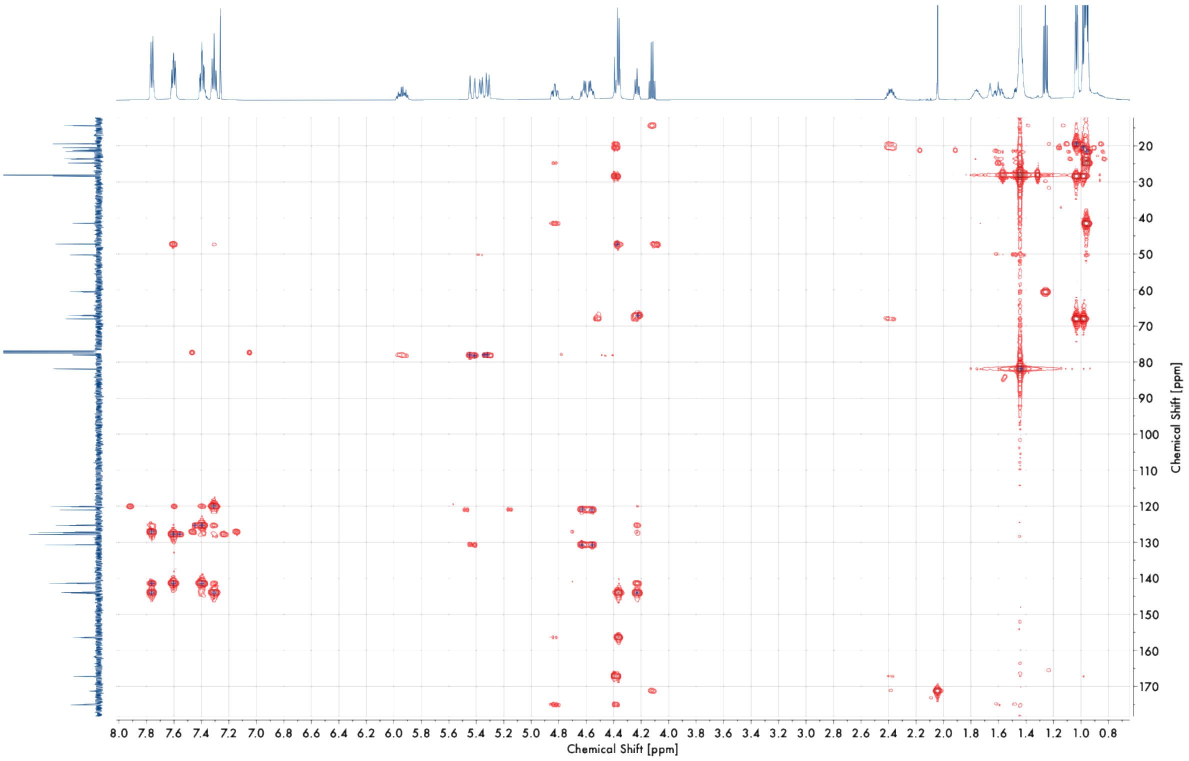

Fmoc-Leu-HyVal(*O*Allyl)-OH [21]

^{1}H NMR (400 MHz, $CDCl_3$, **21**):

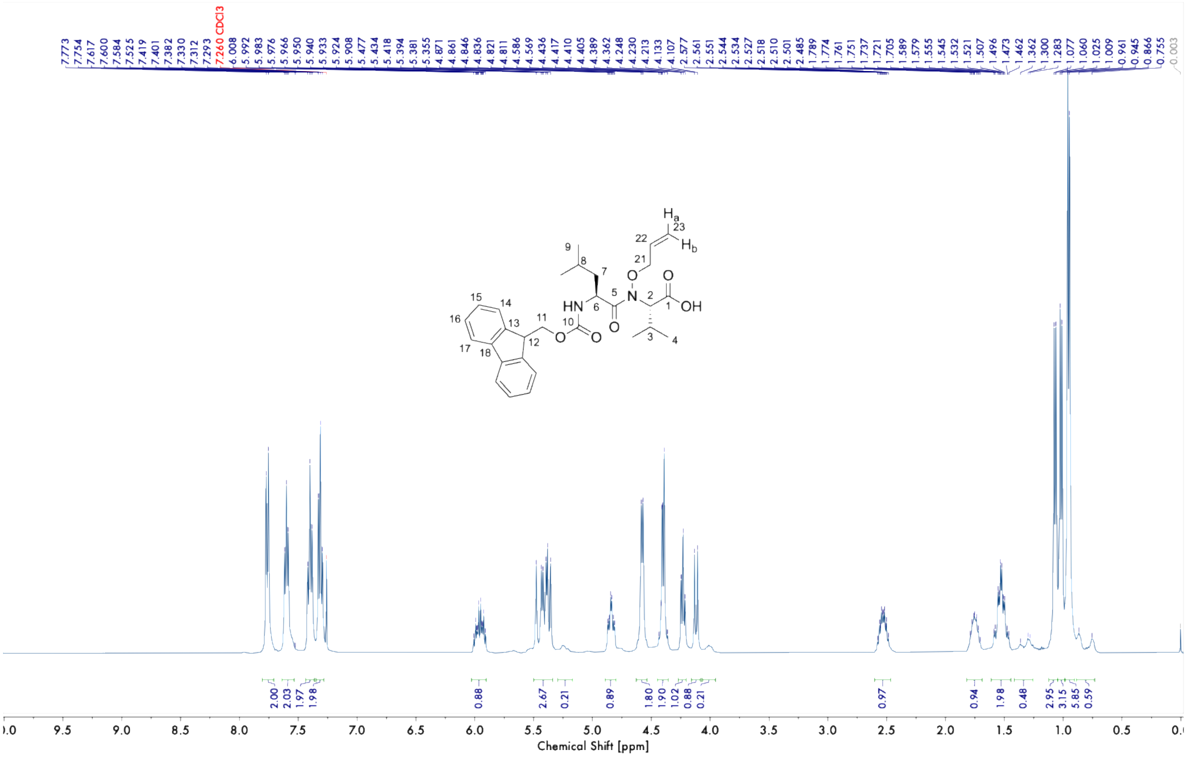

^{13}C NMR (101 MHz, $CDCl_3$, **21**):

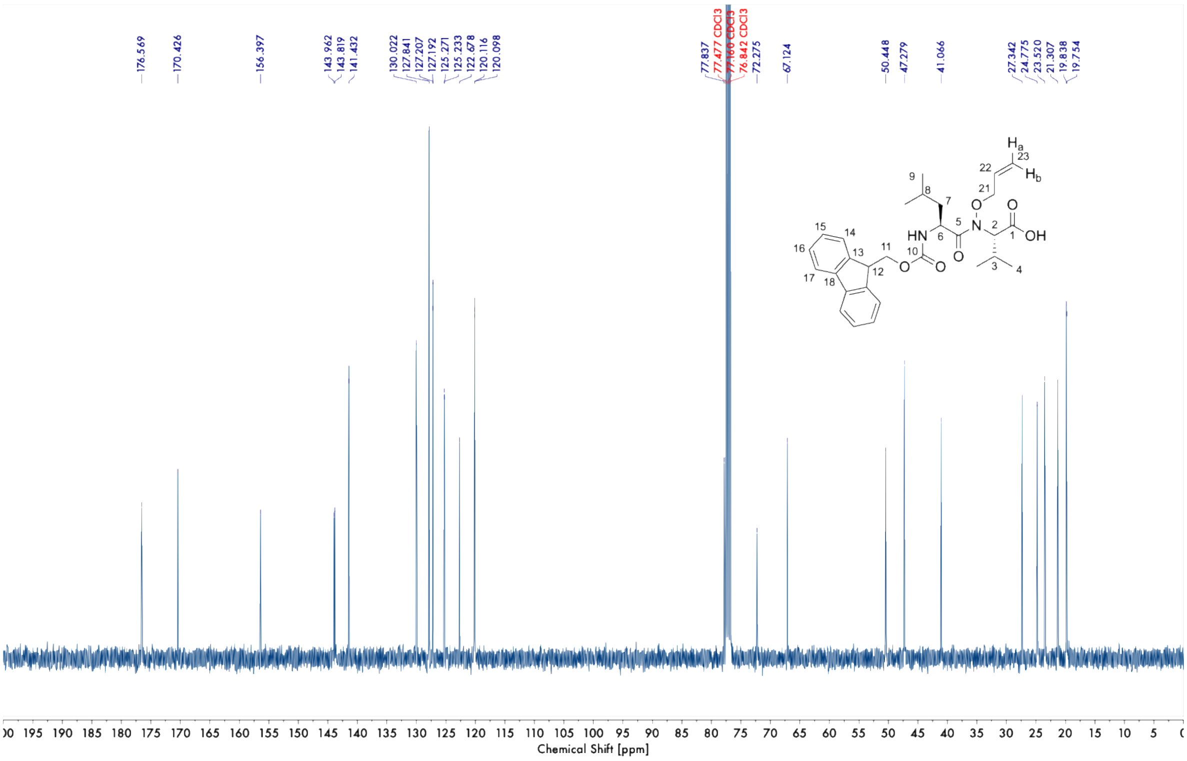

(^{1}H,^{1}H)-COSY (CDCl$_3$, **21**):

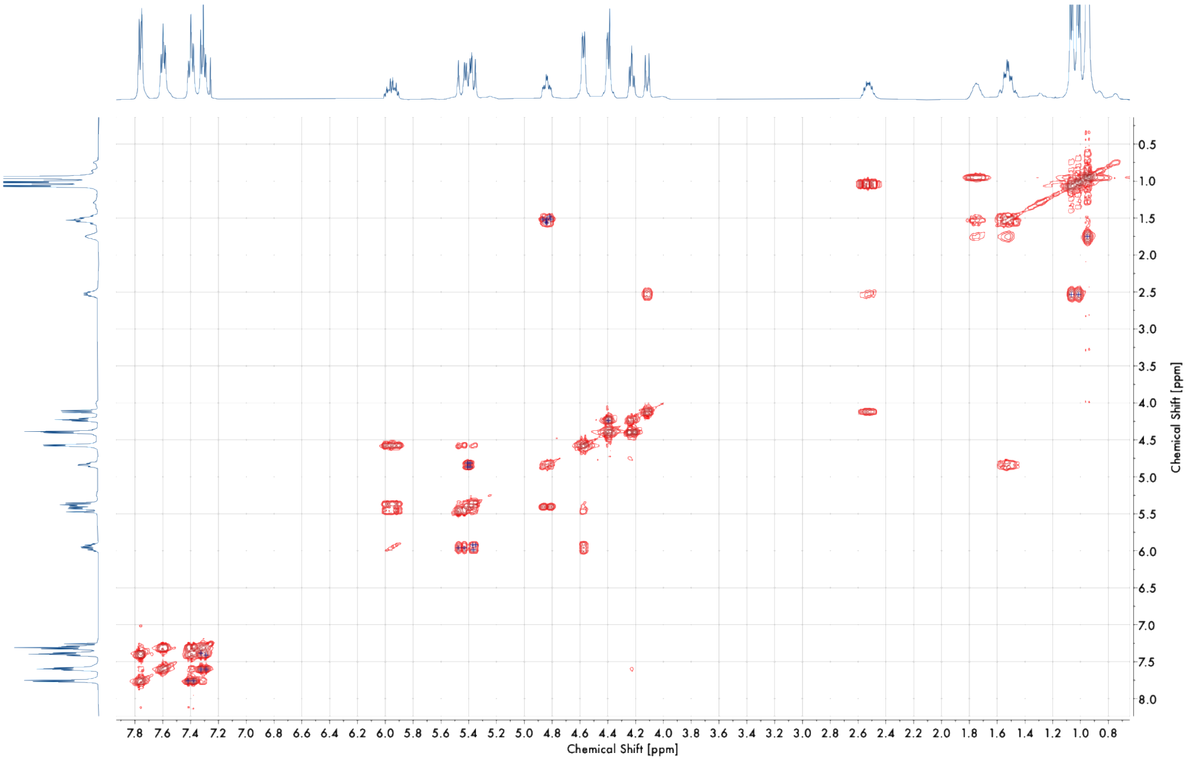

(^{1}H,^{13}C)-HSQC (CDCl$_3$, **21**):

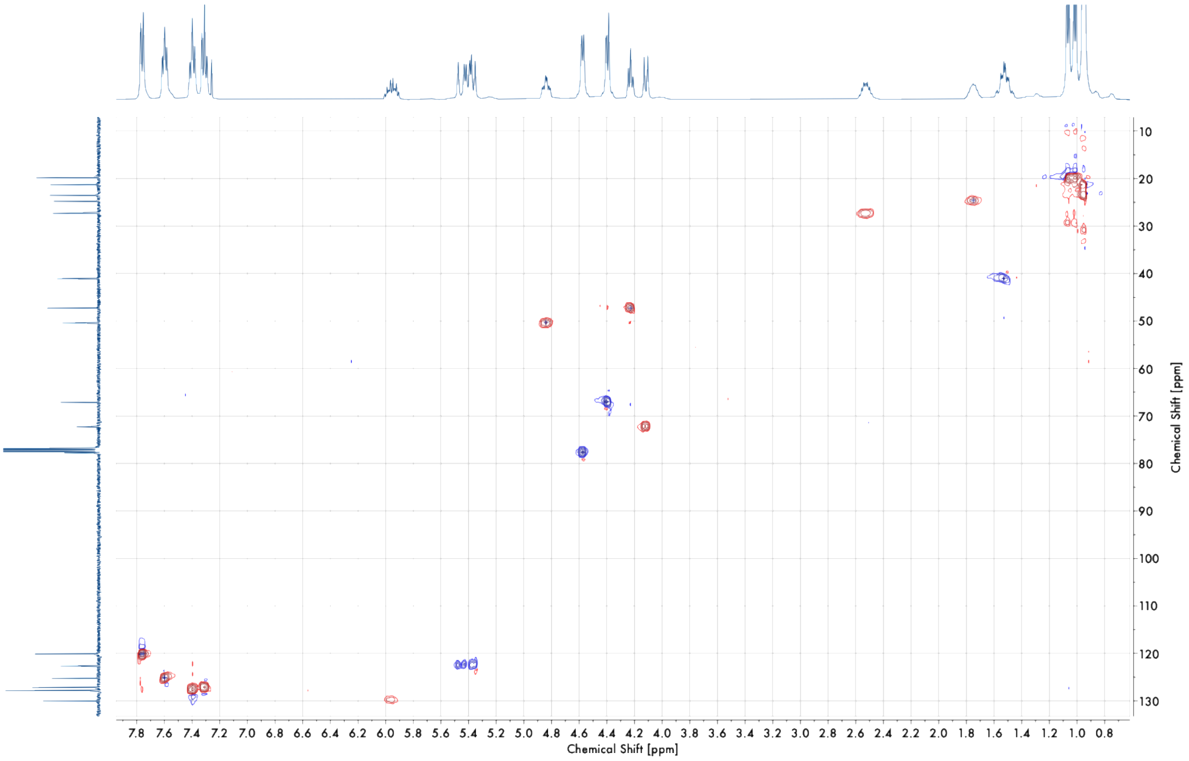

(^{1}H,^{13}C)-HMBC (CDCl$_3$, **21**):

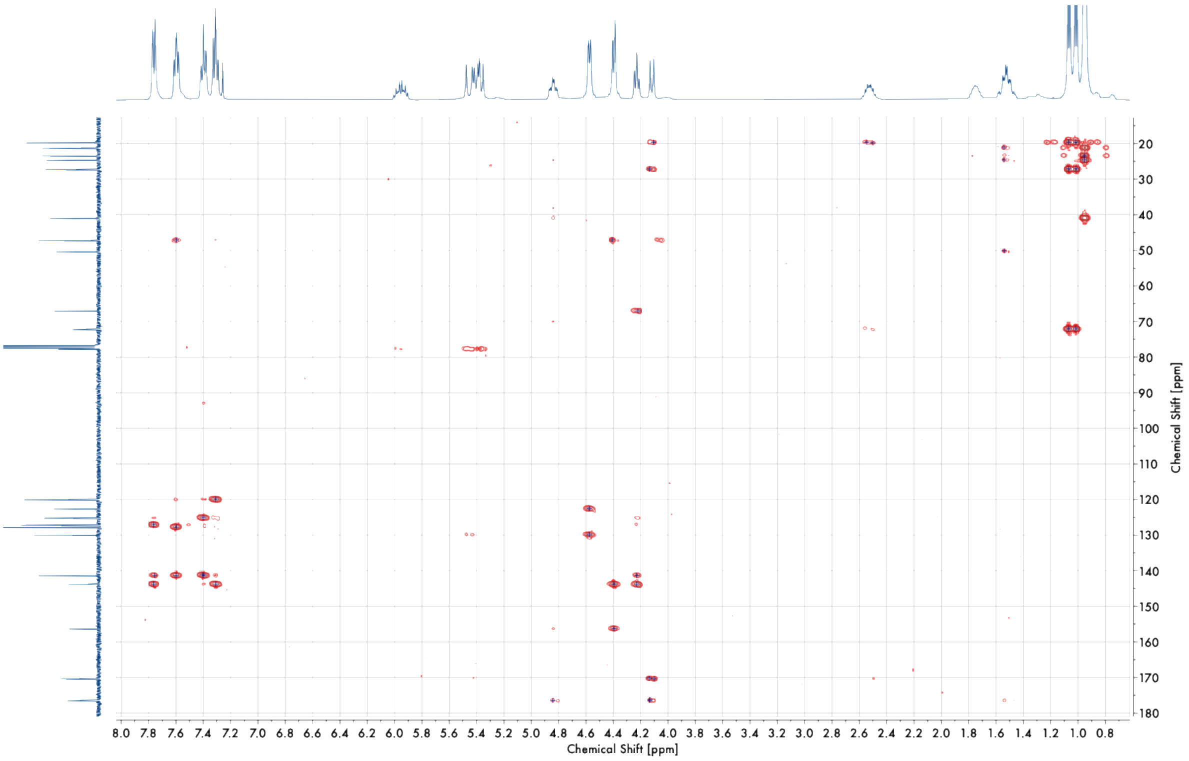

Fmoc-Leu-HyVal(*O*All)-Pro-O*t*-Bu [22]

^{1}H NMR (500 MHz, DMSO-d_6, 373 K):

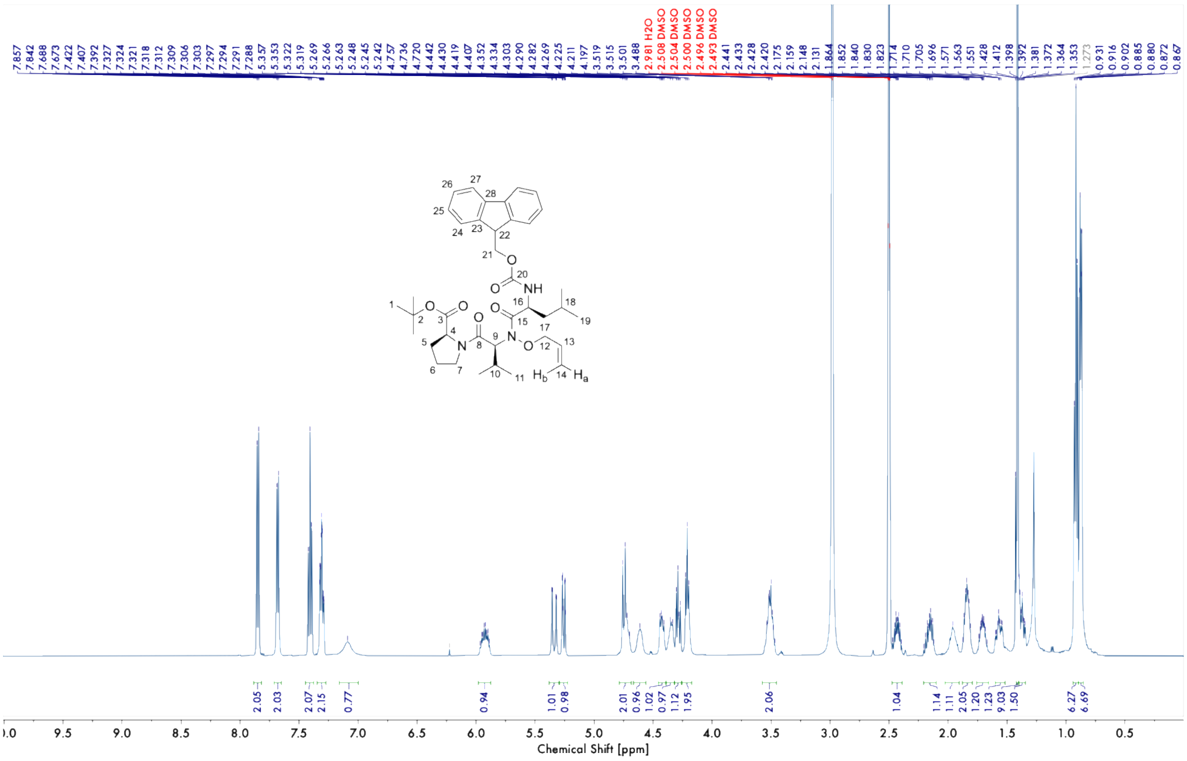

^{13}C NMR (126 MHz, DMSO-d_6, 373 K):

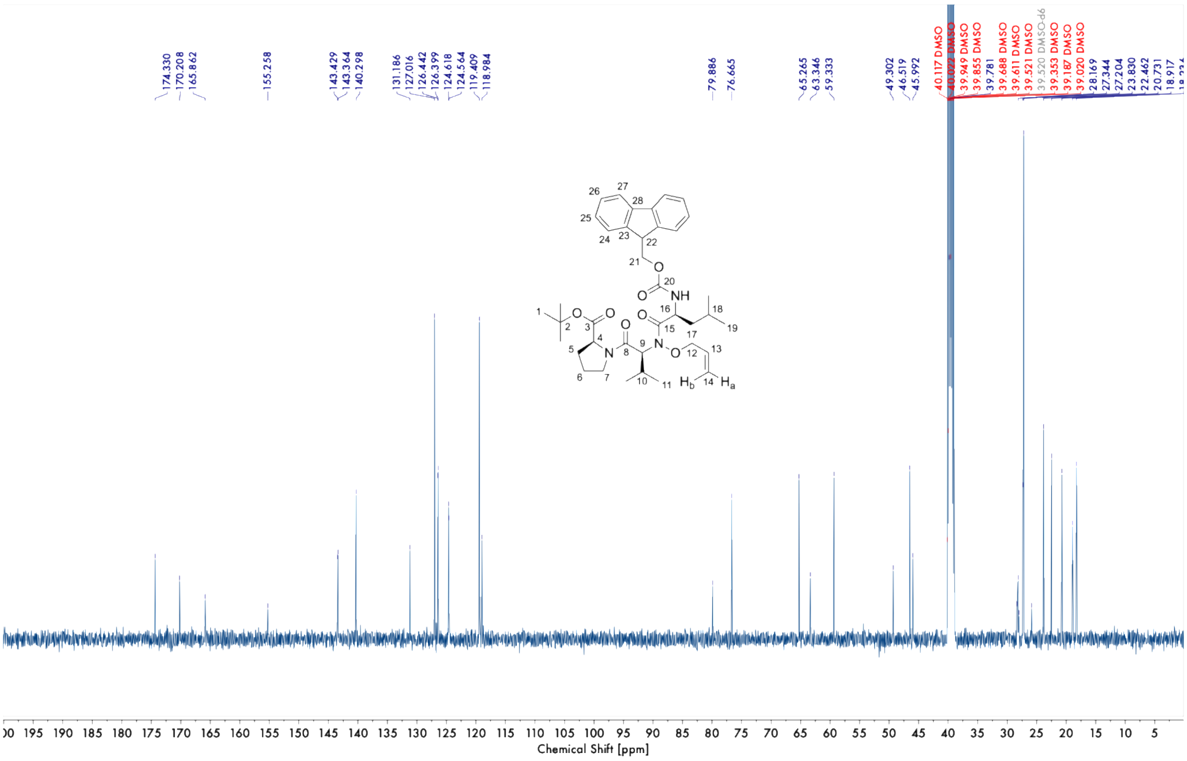

(^{1}H,^{1}H)-COSY (DMSO-d_6, 373 K):

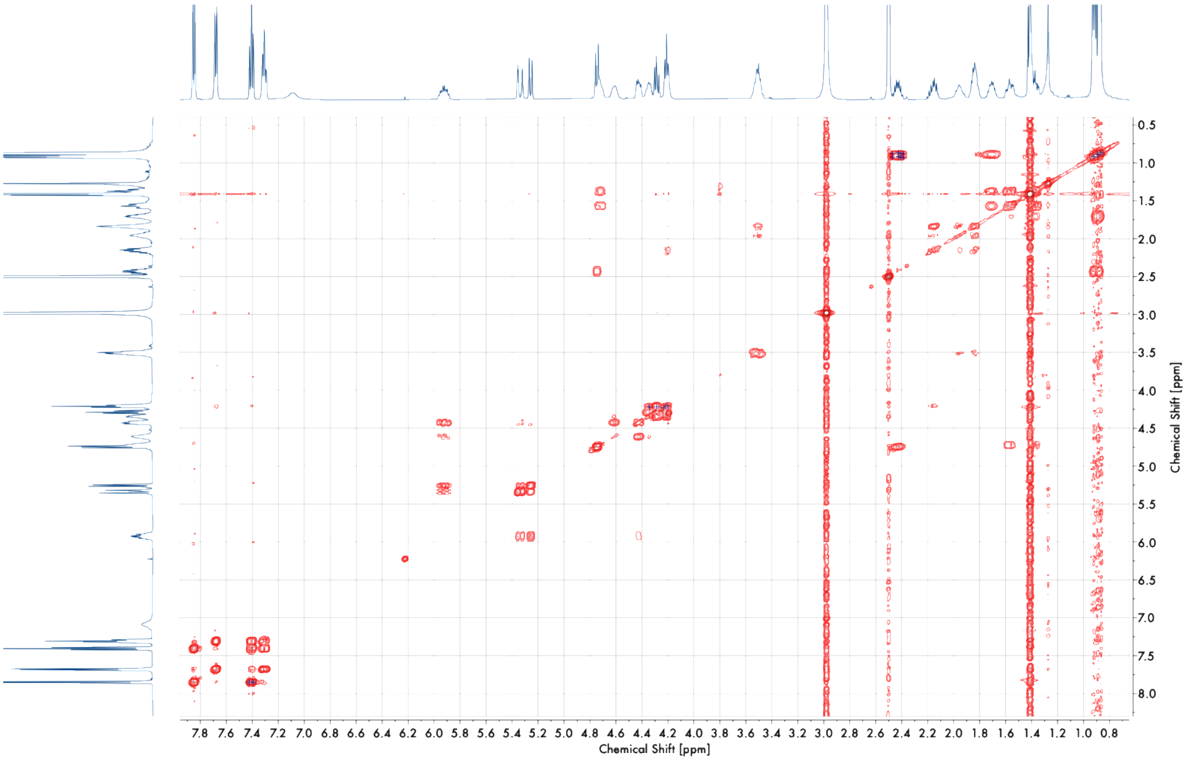

(^{1}H,^{13}C)-HSQC (DMSO-d_6, 373 K):

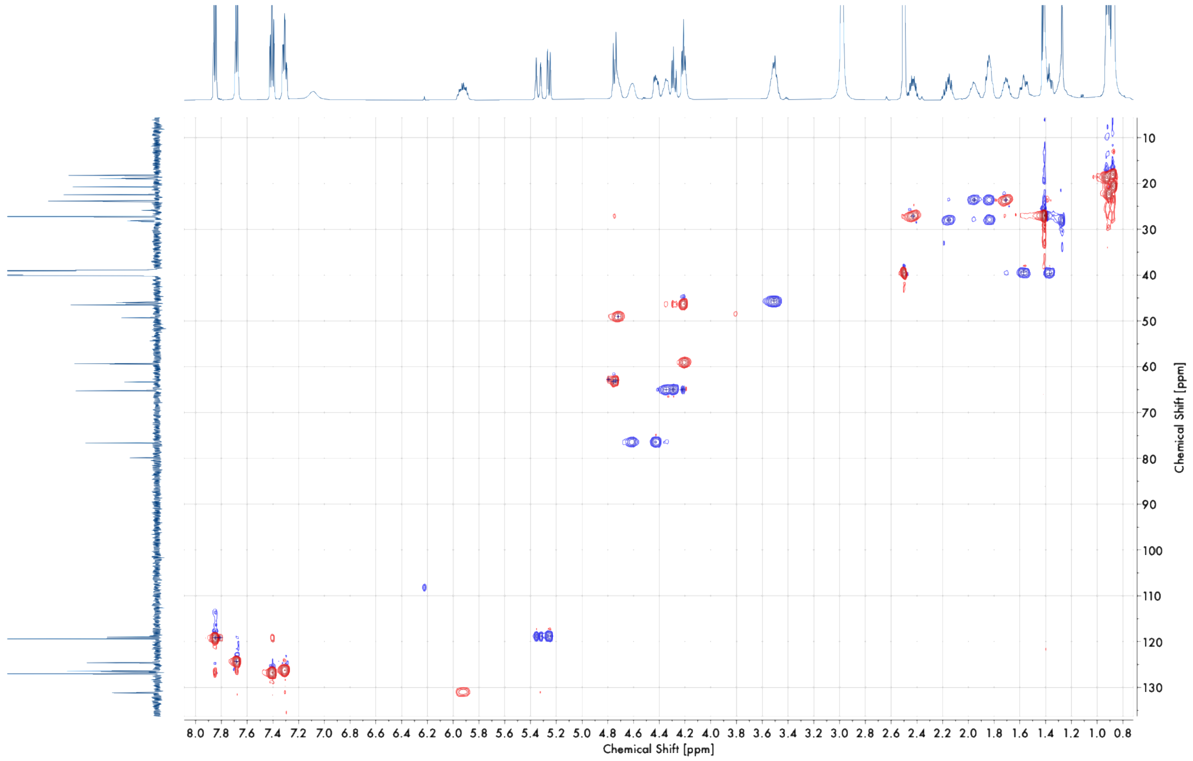

(^{1}H,^{13}C)-HMBC (DMSO-d_6, 373 K):

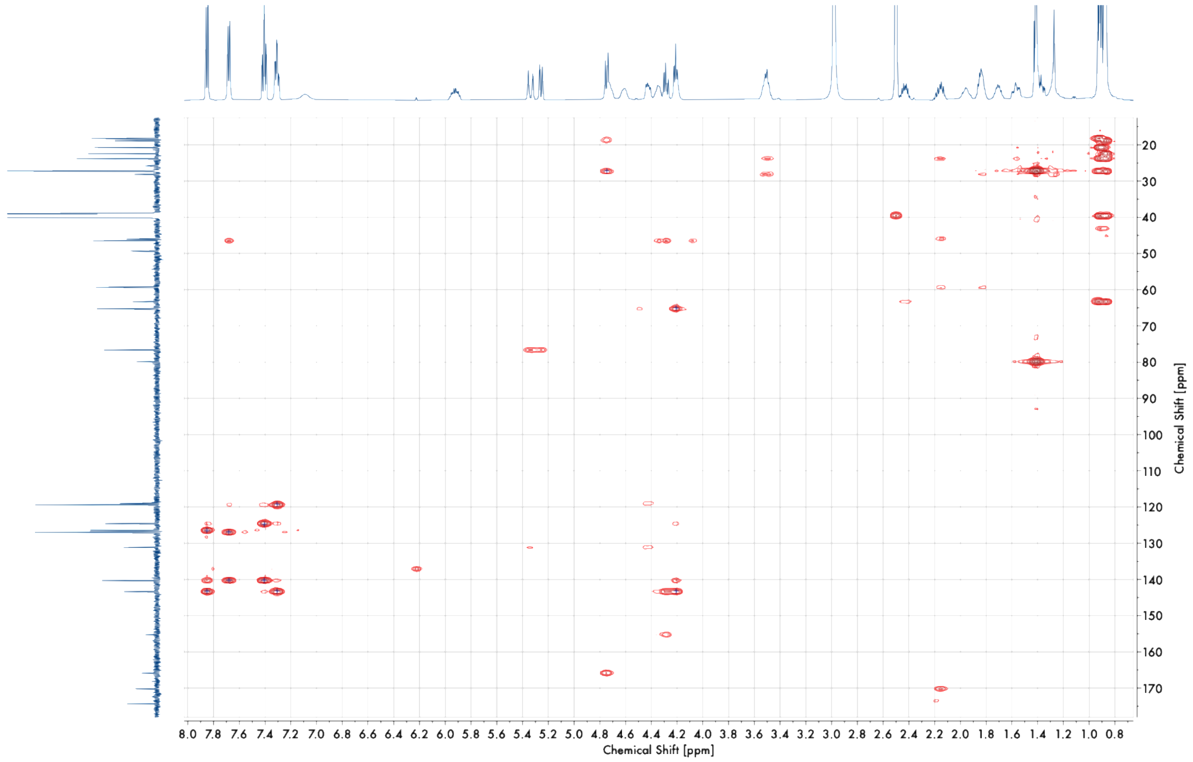

Fmoc-MeHyPro(*O*Ac)-Leu-HyVal(*O*All)-Pro-O*t*-Bu [23]

^{1}H NMR (500 MHz, DMSO-d_6, 373 K, **23**):

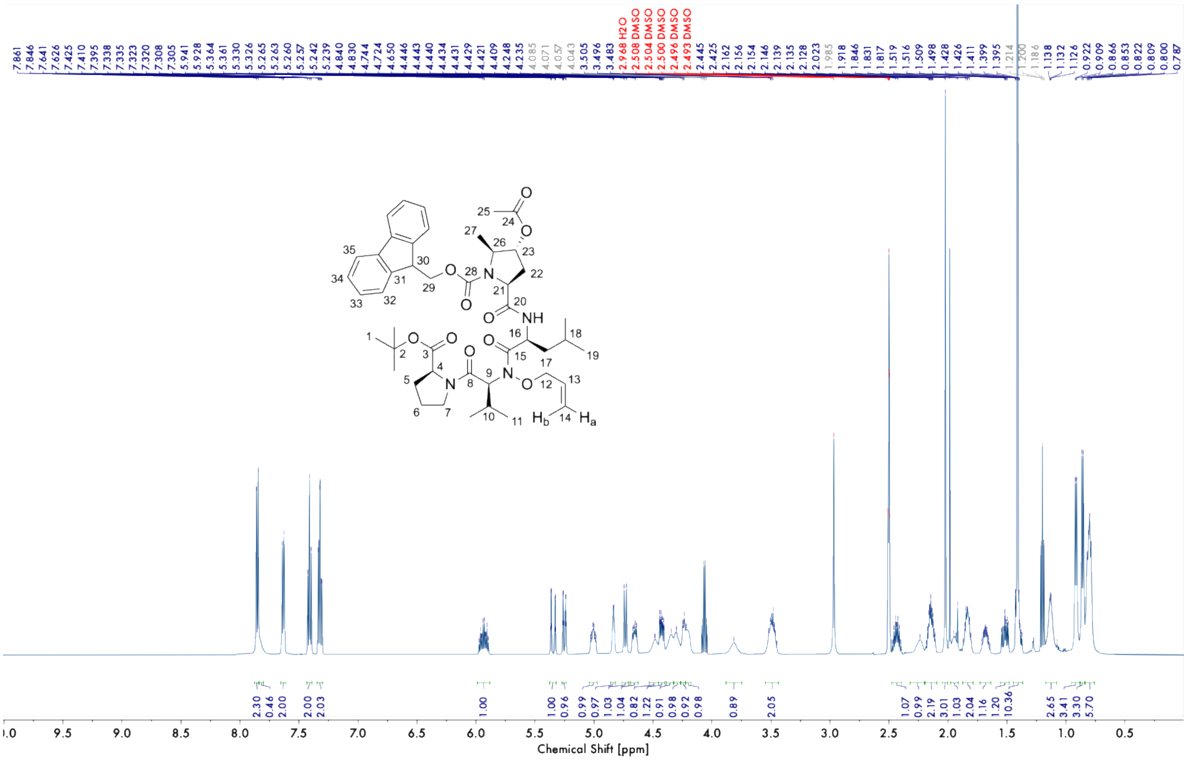

^{13}C NMR (126 MHz, DMSO-d_6, 373 K, **23**):

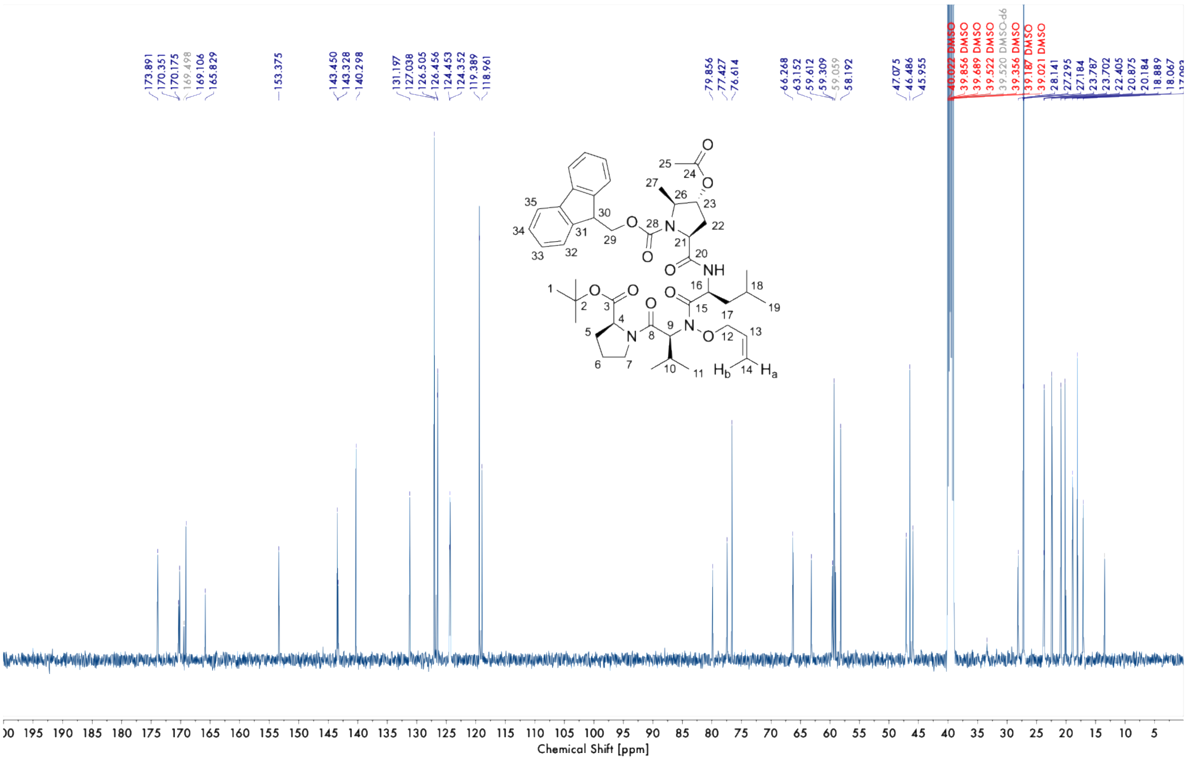

(^{1}H,^{1}H)-COSY (DMSO-d_6, 373 K, **23**):

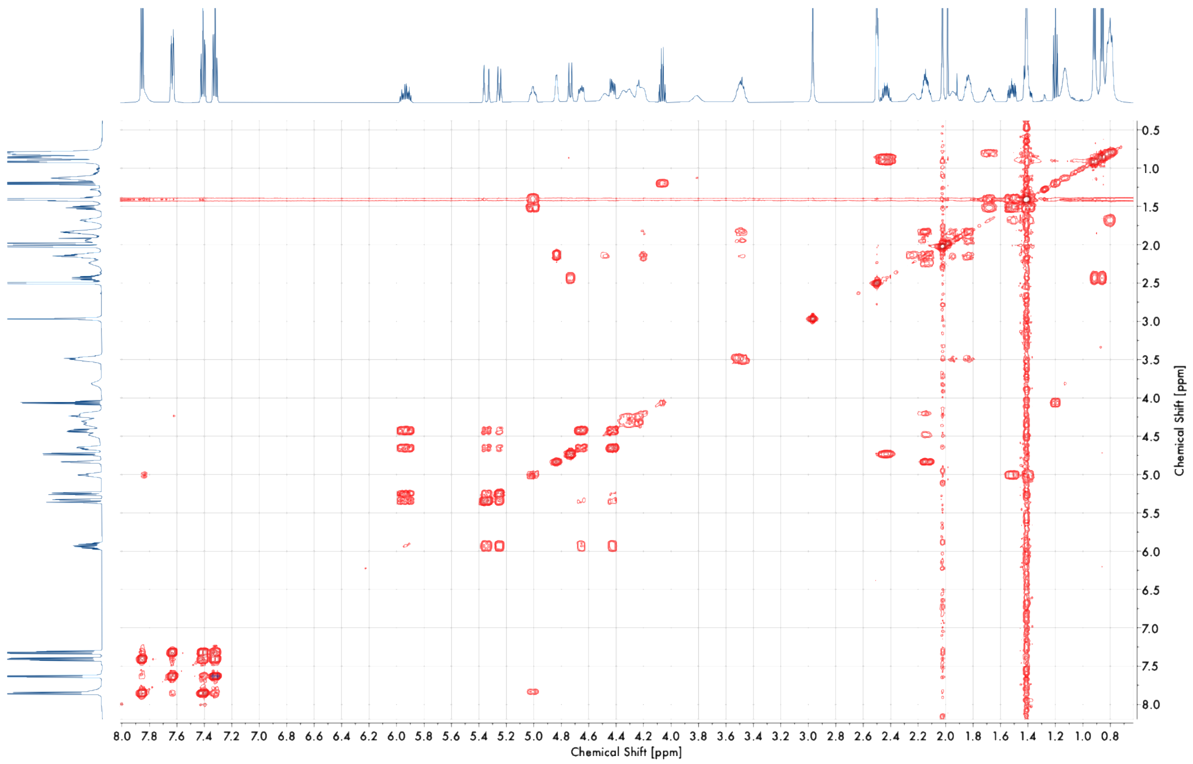

(^{1}H,^{13}C)-HSQC (DMSO-d_6, 373 K, **23**):

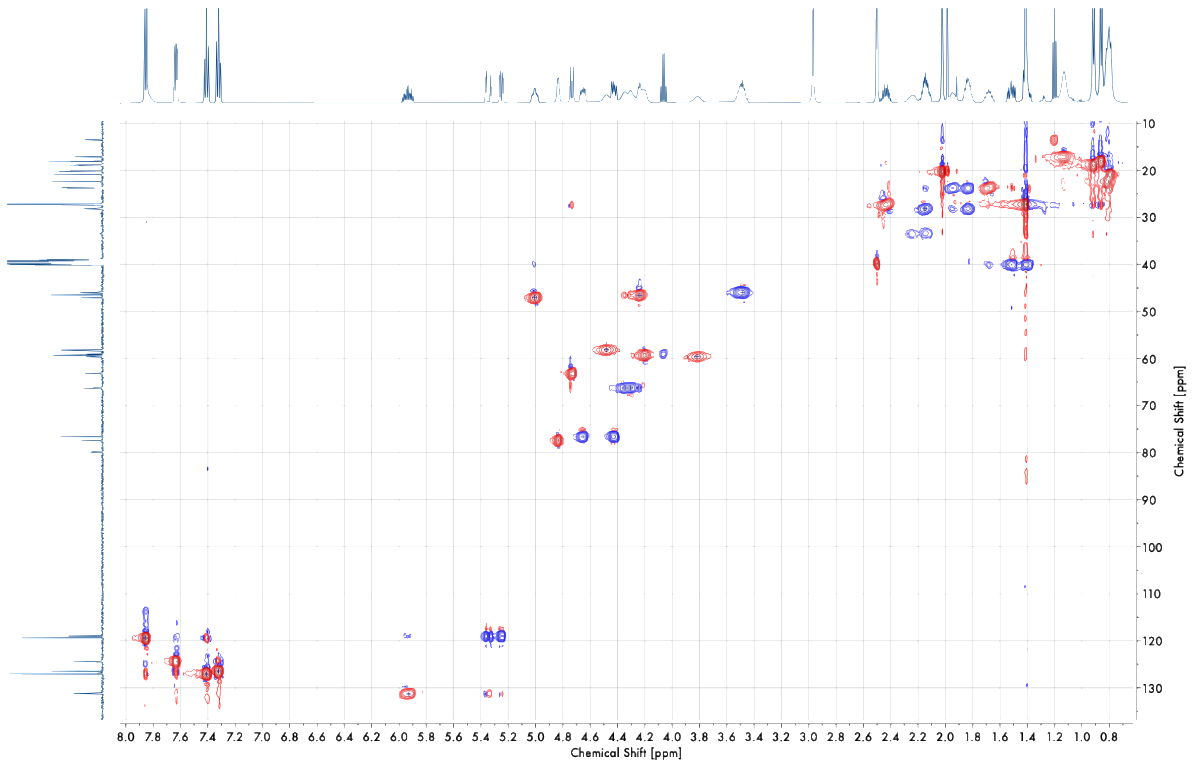

(¹H,¹³C)-HMBC (DMSO-d_6, 373 K, **23**):

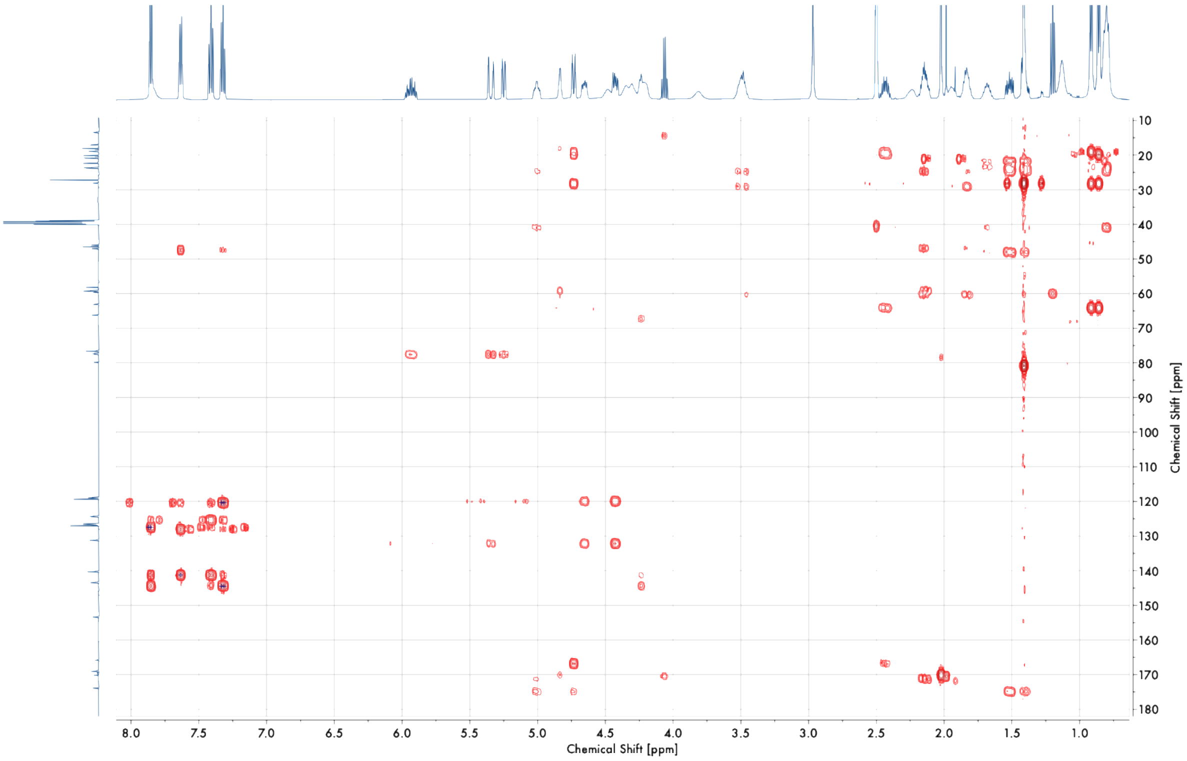

Fmoc-Leu-MeHyPro(*O*Ac)-Leu-HyVal(*O*All)-Pro-O*t*-Bu [24]

¹H NMR (500 MHz, DMSO-d_6, 373 K, **24**):

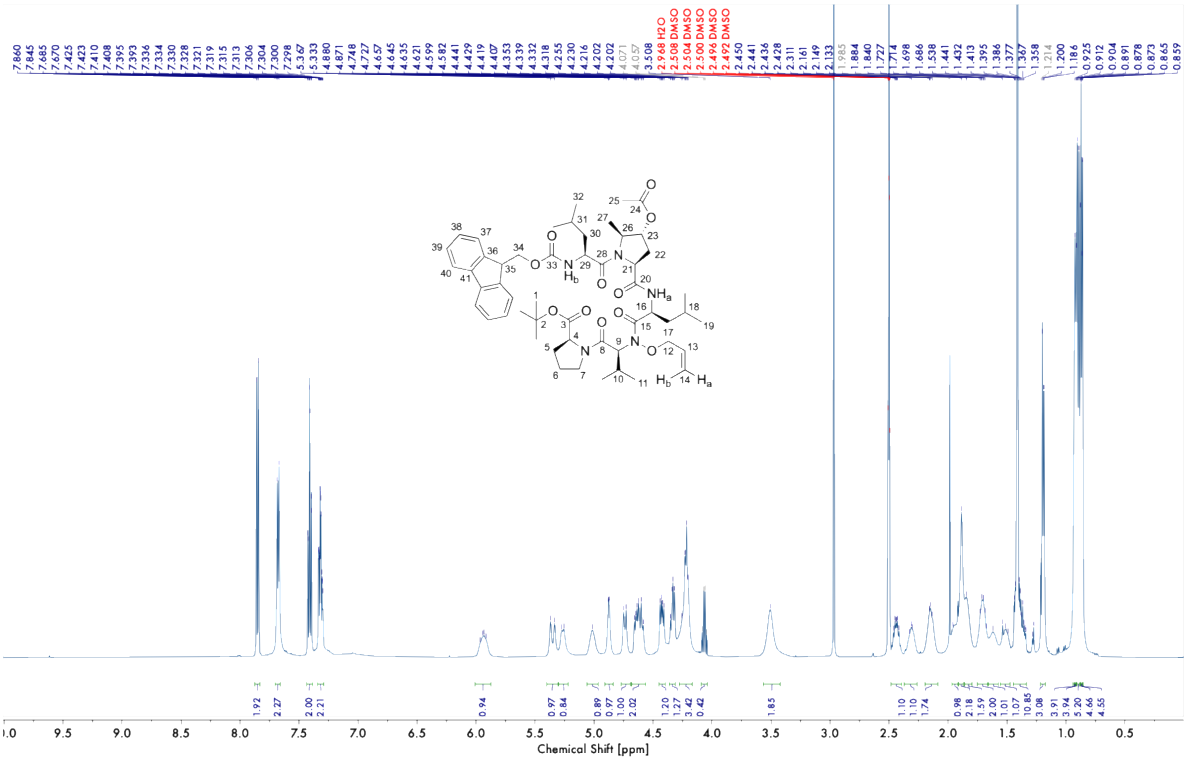

^{13}C NMR (126 MHz, DMSO-d_6, 373 K, **24**):

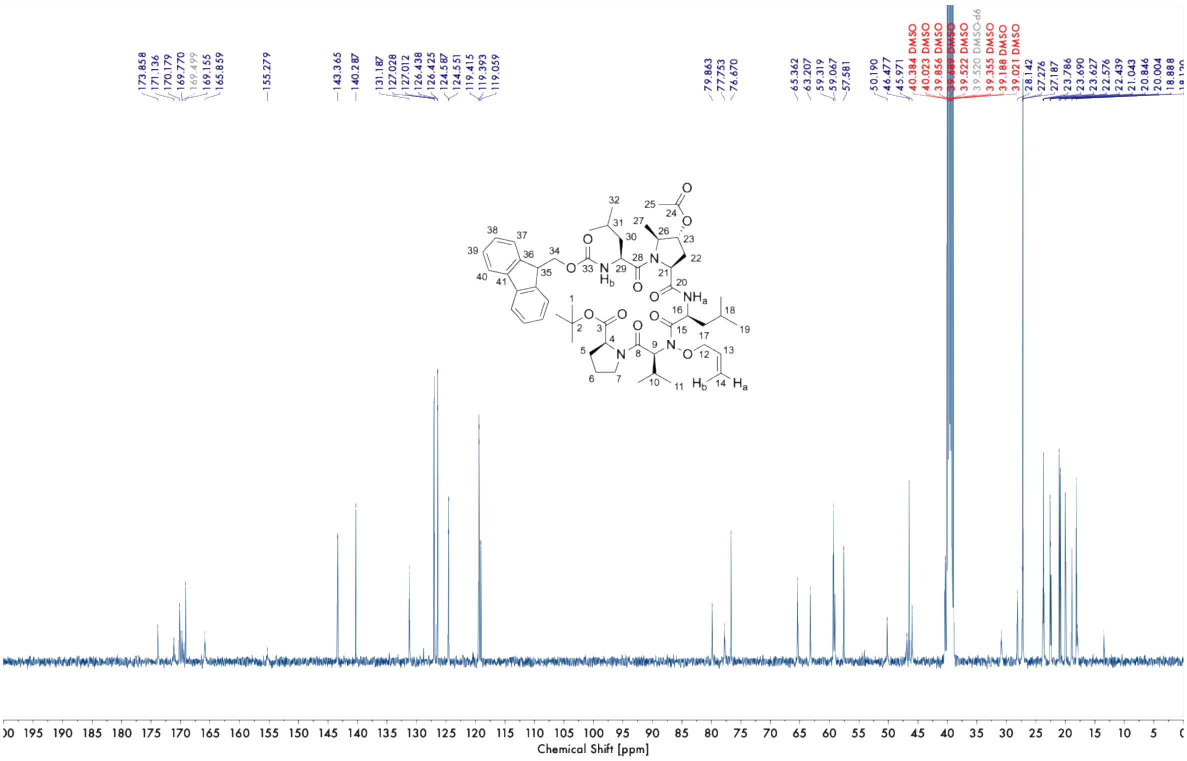

(^{1}H,^{1}H)-COSY (DMSO-d_6, 373 K, **24**):

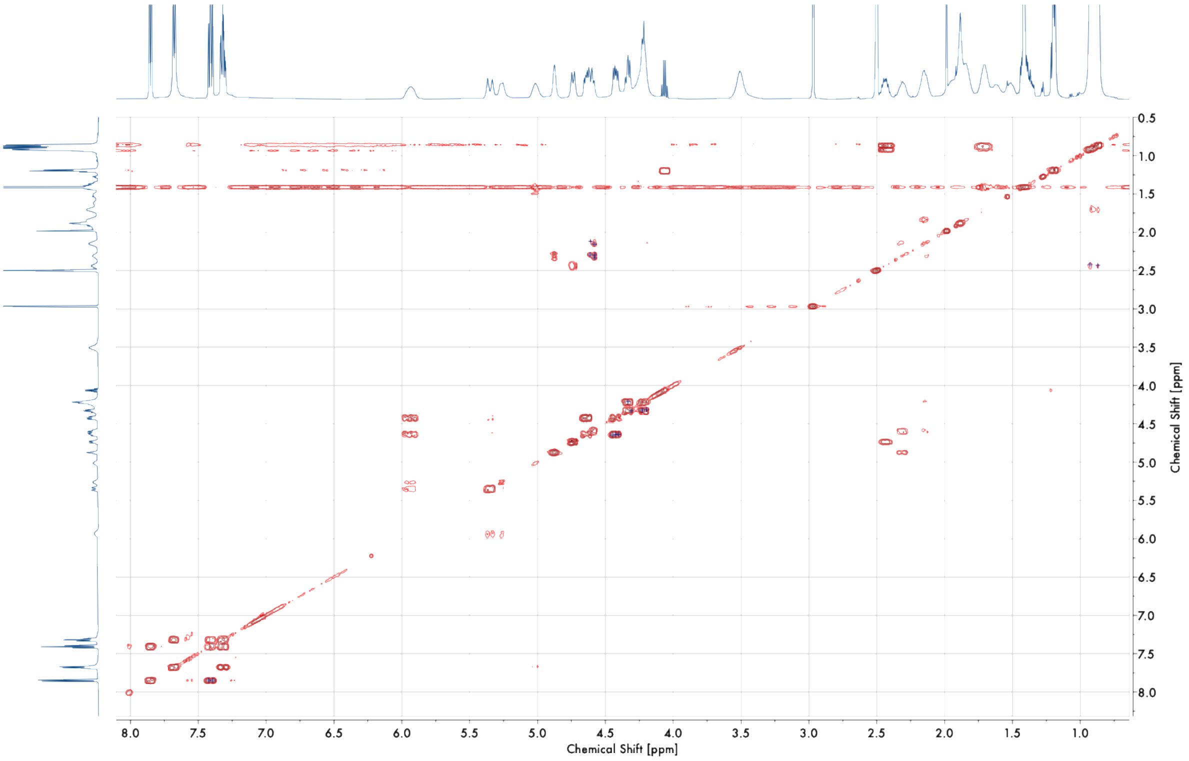

(^{1}H,^{13}C)-HSQC (DMSO-d_6, 373 K, **24**):

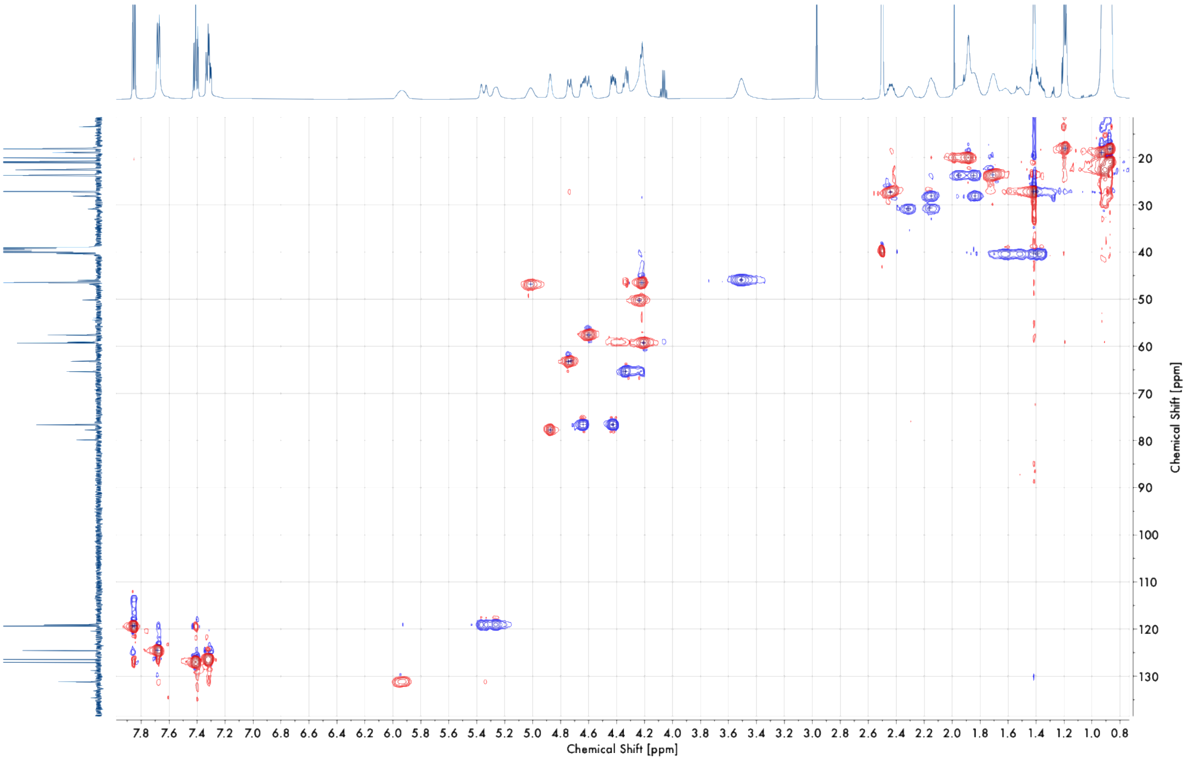

(^{1}H,^{13}C)-HMBC (DMSO-d_6, 373 K, **24**):

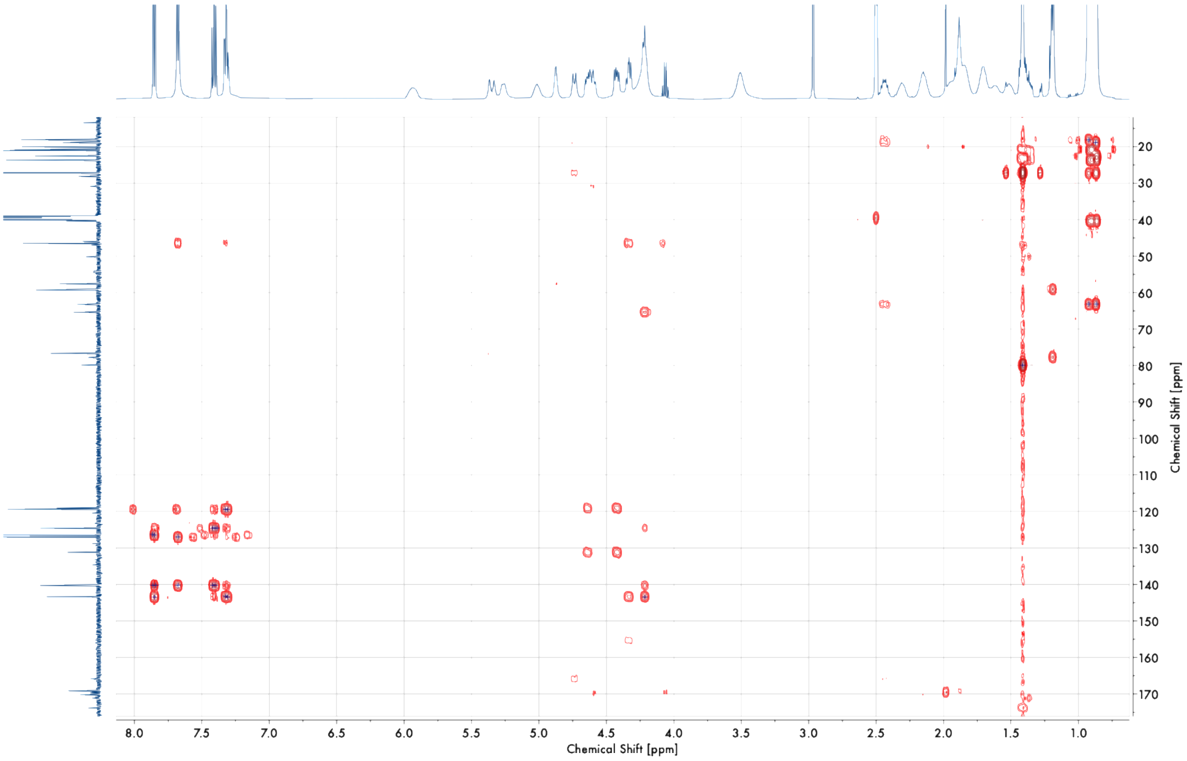

Fmoc-Thr-Leu-MeHyPro(*O*Ac)-Leu-HyVal(*O*All)-Pro-O*t*-Bu [25]

^{1}H NMR (500 MHz, DMSO-d_6, **25**):

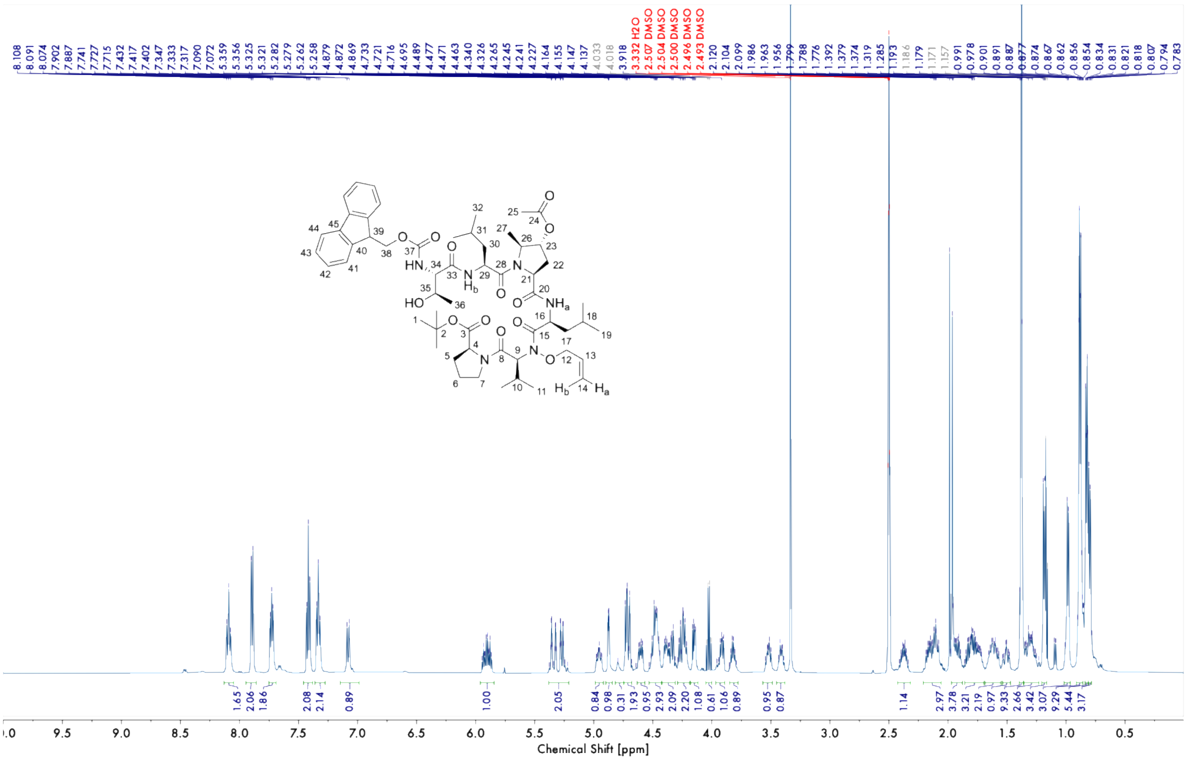

^{13}C NMR (126 MHz, DMSO-d_6, **25**):

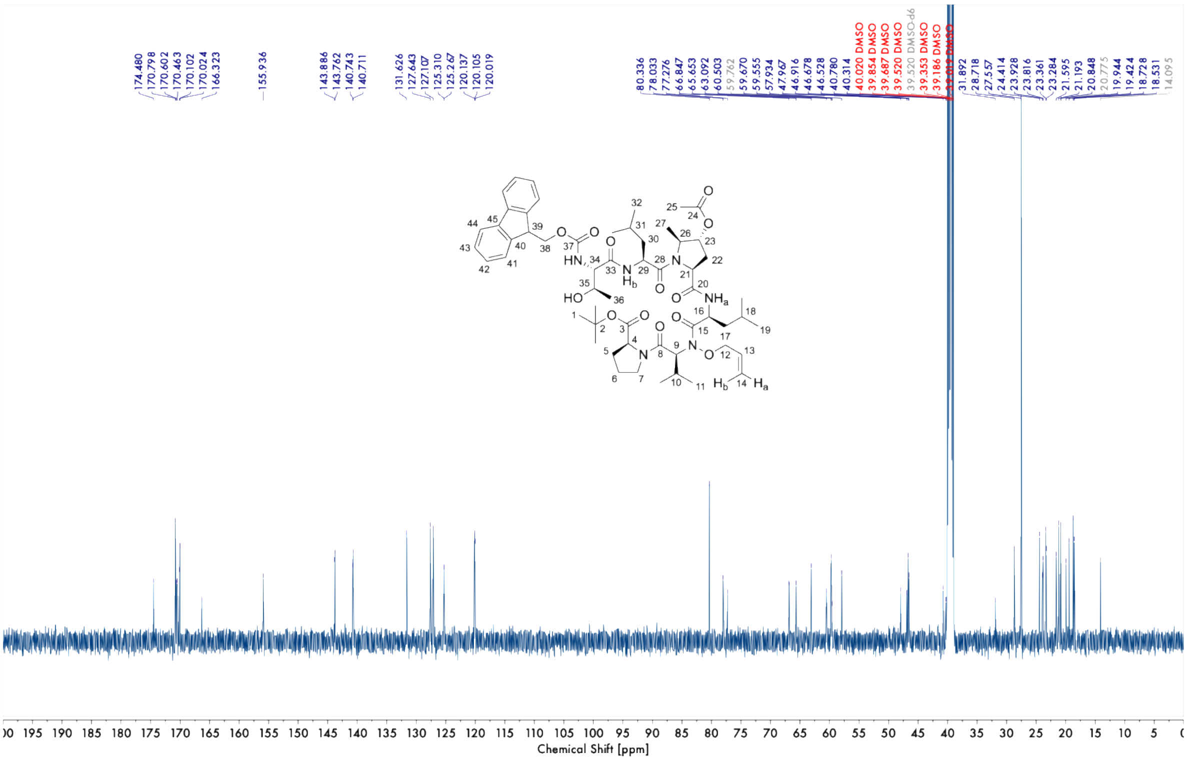

(^{1}H,^{1}H)-COSY (DMSO-d_6, **25**):

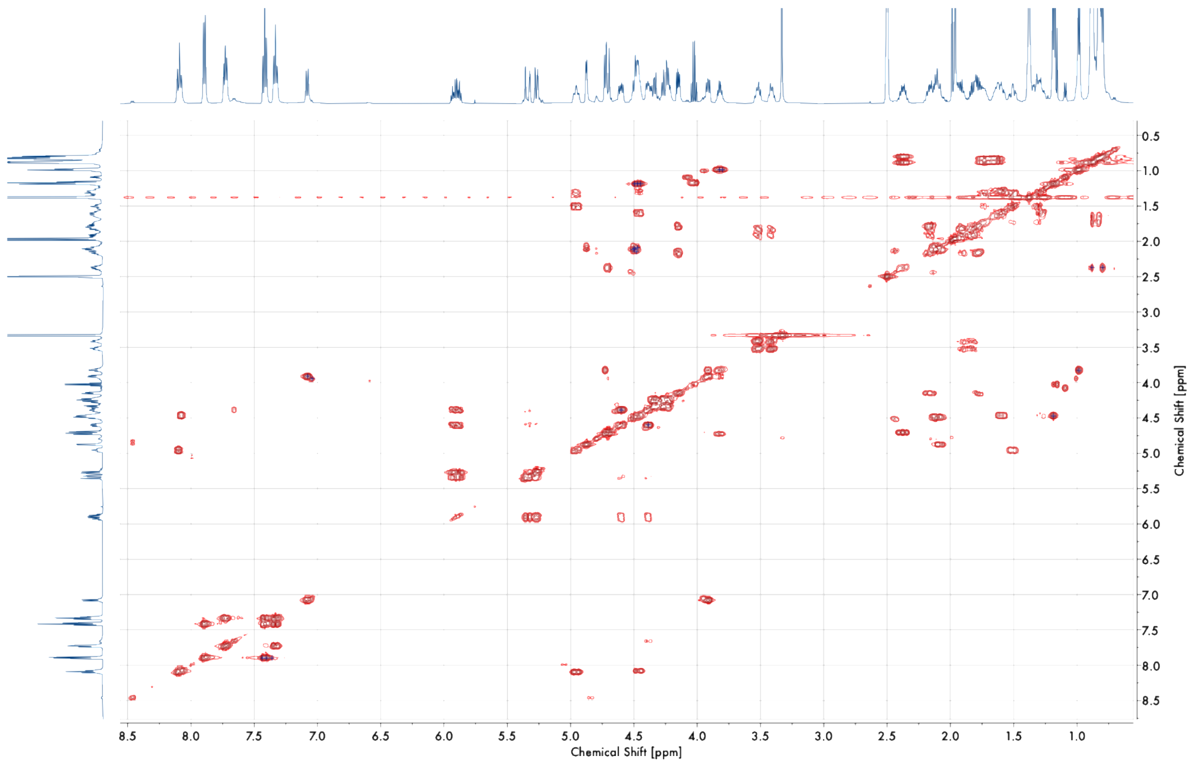

(^{1}H,^{13}C)-HSQC (DMSO-d_6, **25**):

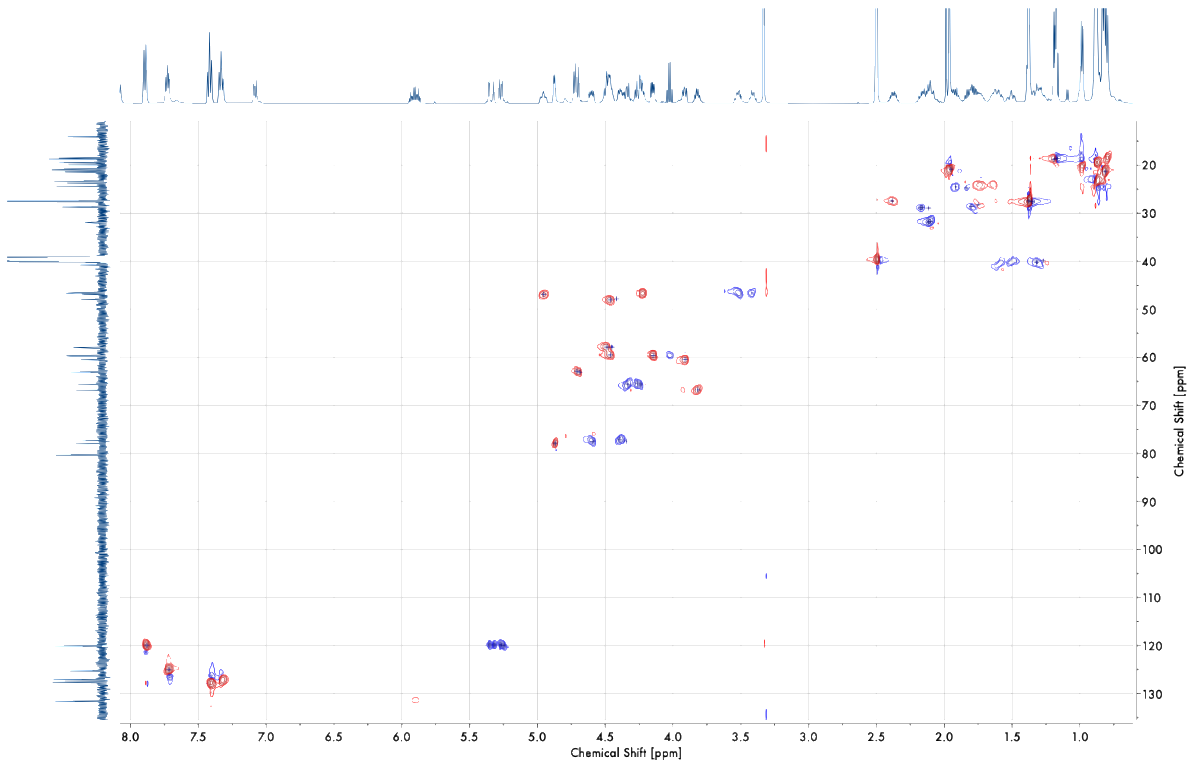

(^{1}H,^{13}C)-HMBC (DMSO-d_6, **25**):

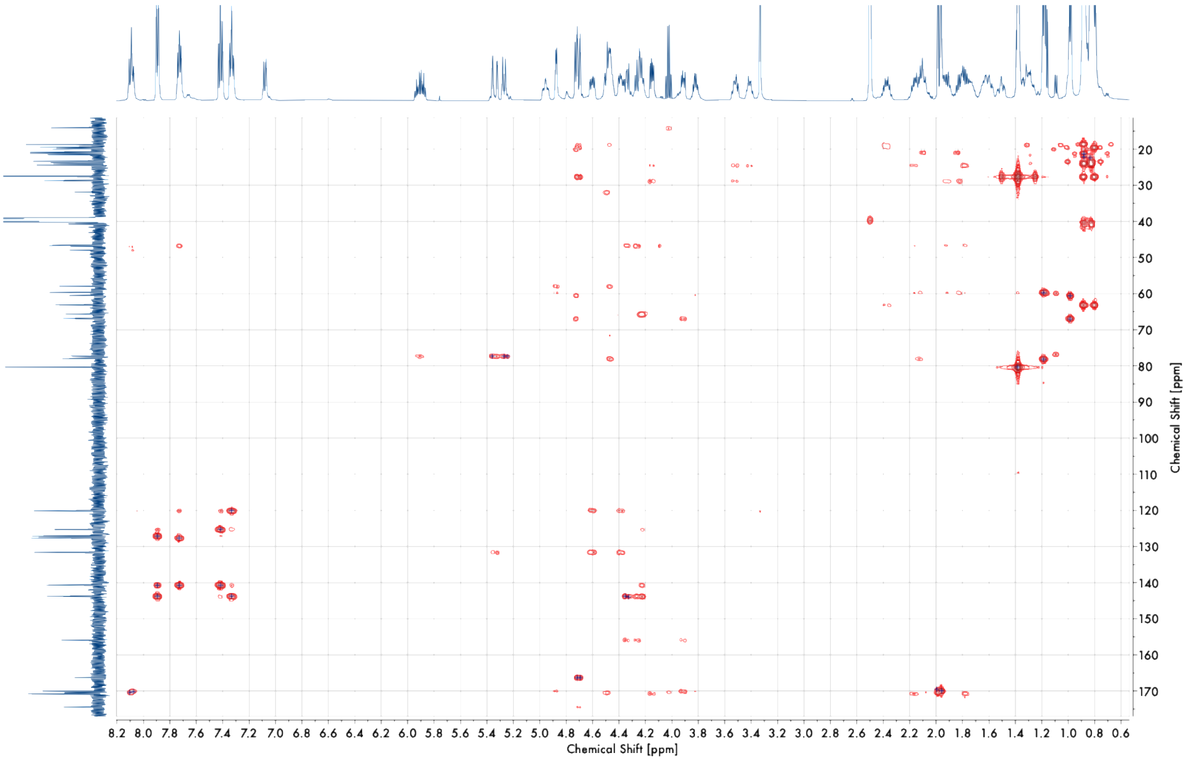

SacCl(*O*All)-Thr-Leu-MeHyPro(*O*Ac)-Leu-HyVal(*O*All)-Pro-O*t*-Bu [26]

^{1}H NMR (500 MHz, DMSO-d_6, 373 K, **26**):

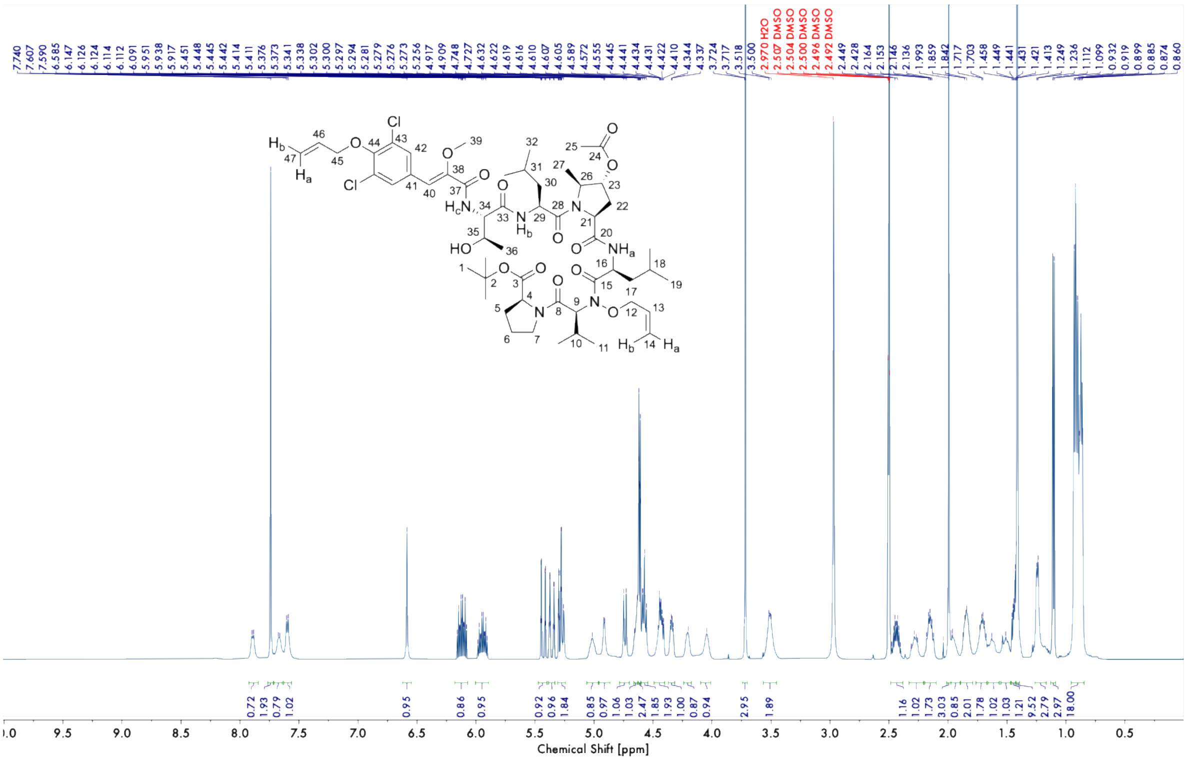

^{13}C NMR (126 MHz, DMSO-d_6, 373 K, **26**):

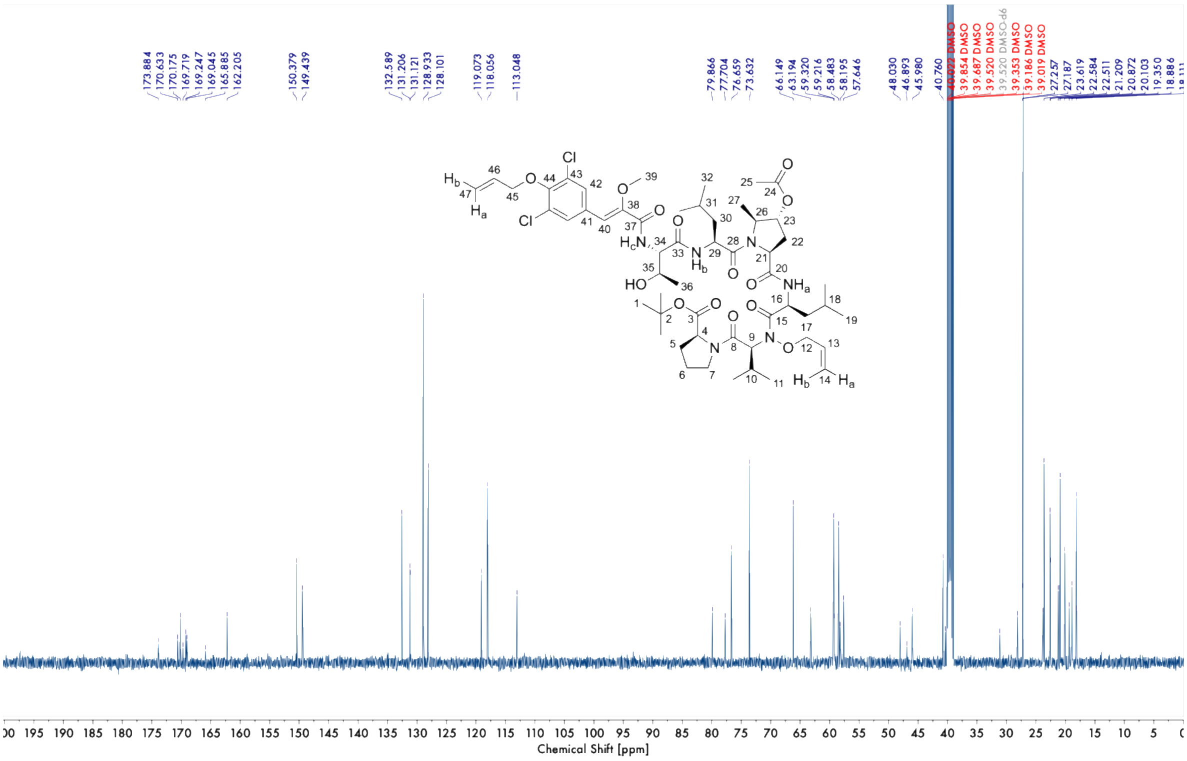

(^{1}H,^{1}H)-COSY (DMSO-d_6, 373 K, **26**):

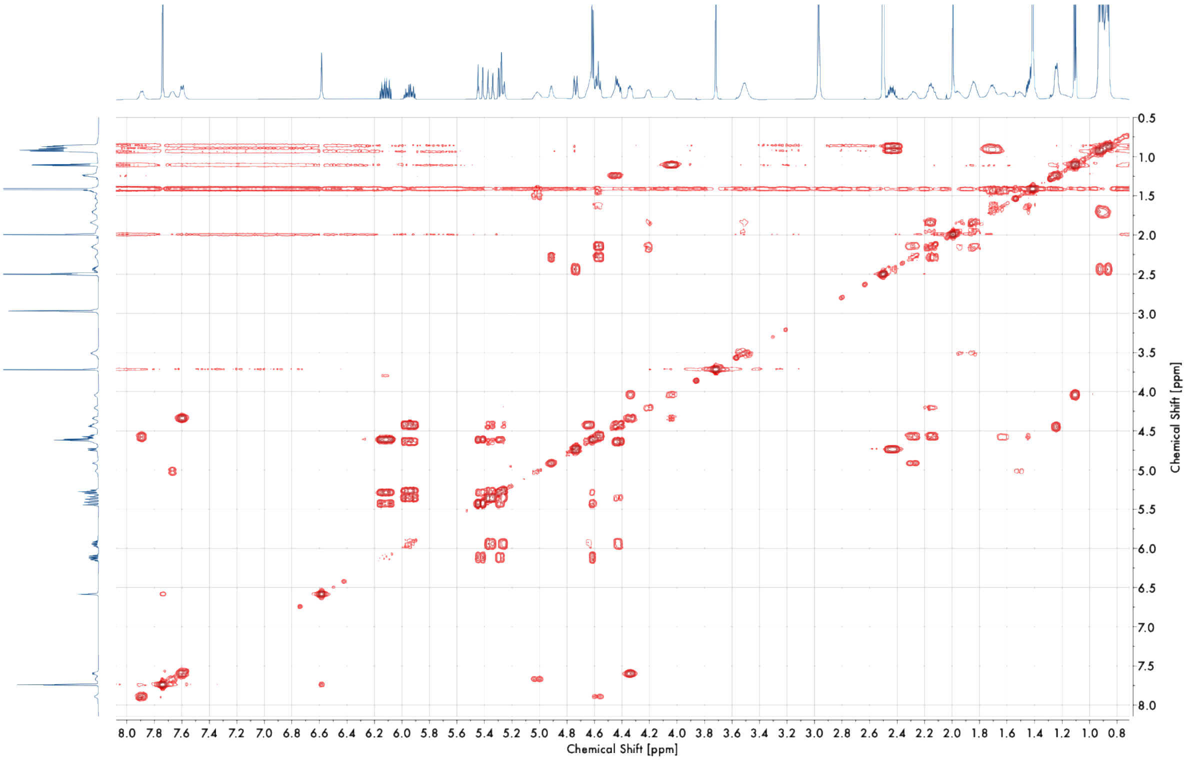

(^{1}H,^{13}C)-HSQC (DMSO-d_6, 373 K, **26**):

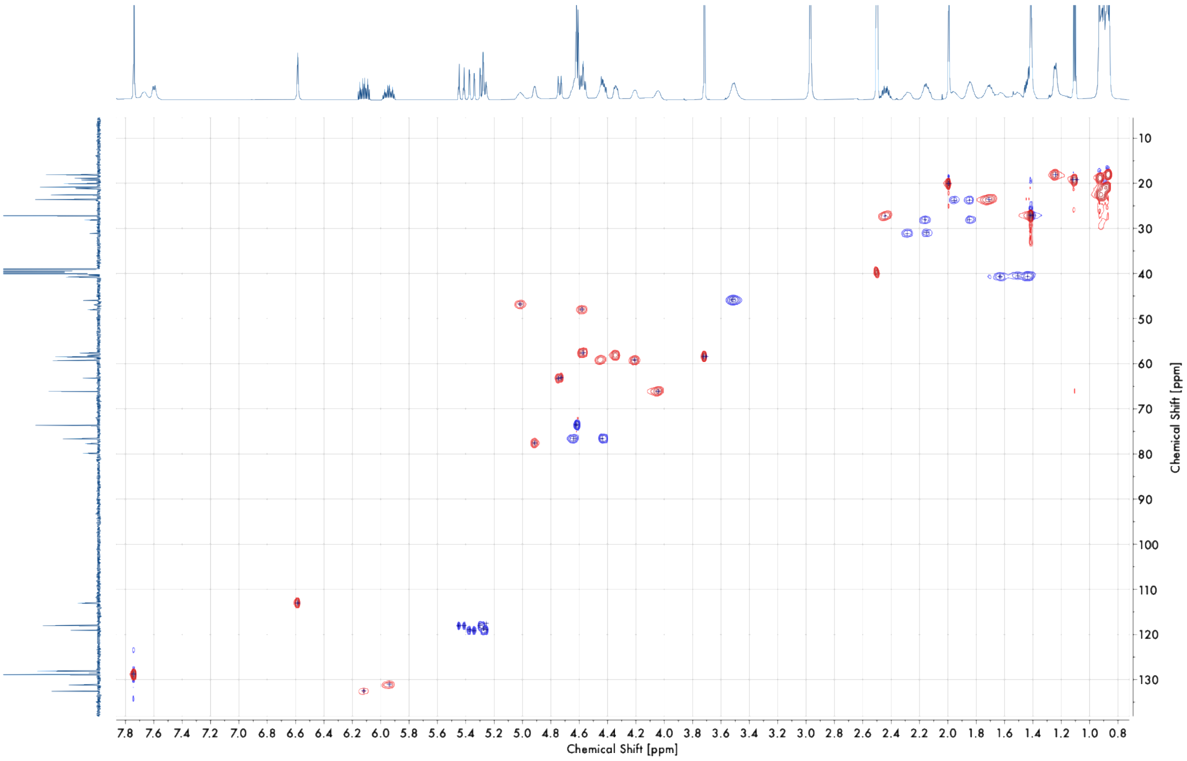

(^{1}H,^{13}C)-HMBC (DMSO-d_6, 373 K, **26**):

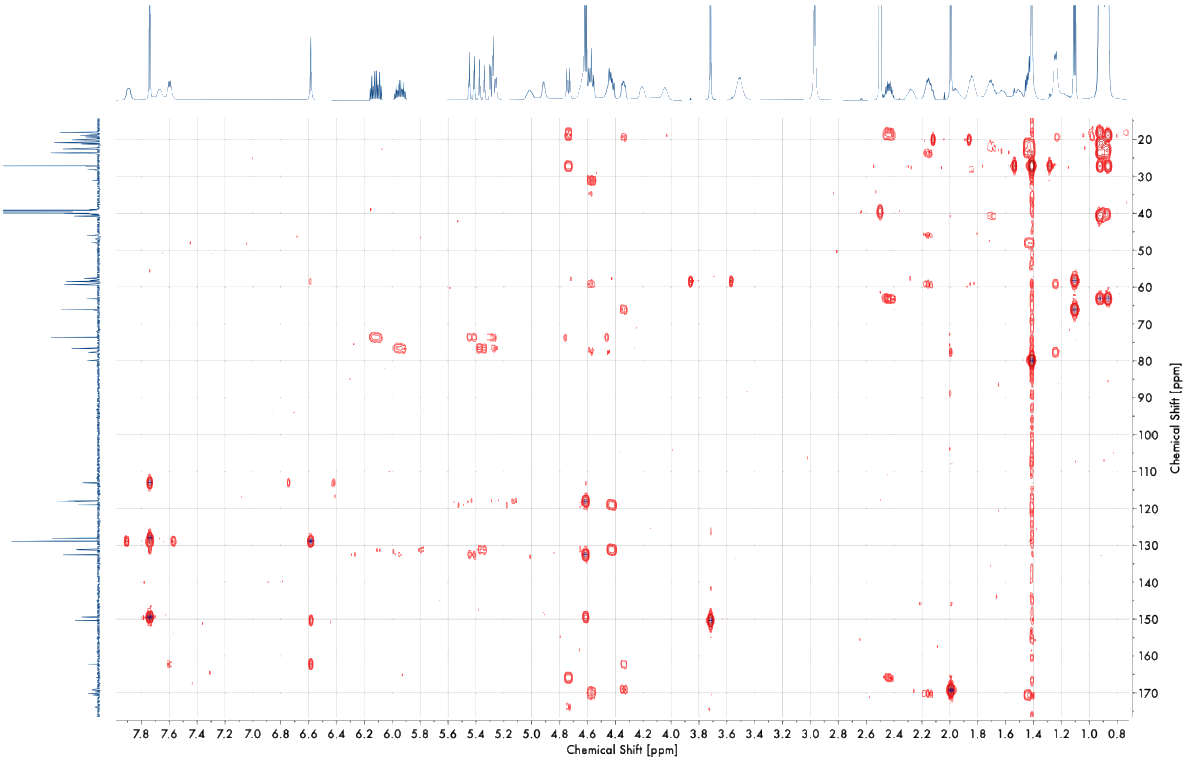

Bonnevillamide C [27]

^{1}H NMR (500 MHz, DMSO-d_6, **27**):

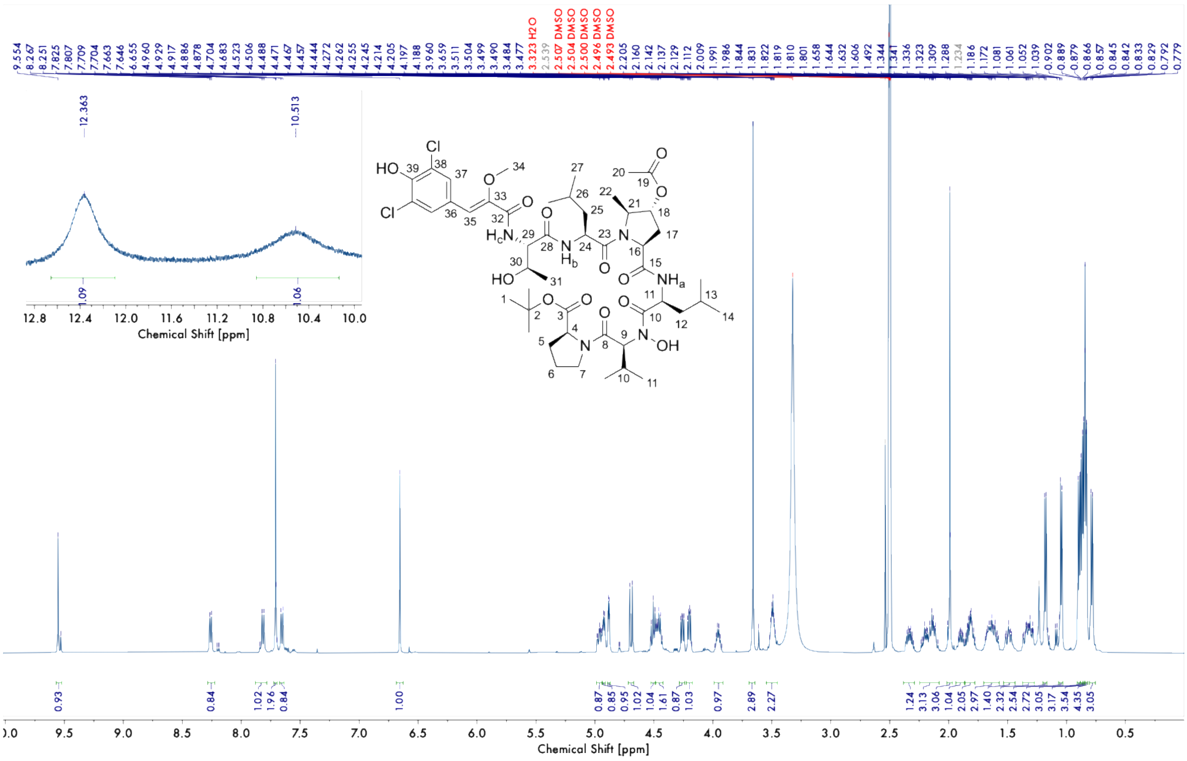

^{1}H NMR (500 MHz, DMSO-d_6 + 0.4 vol% TFA, **27**):

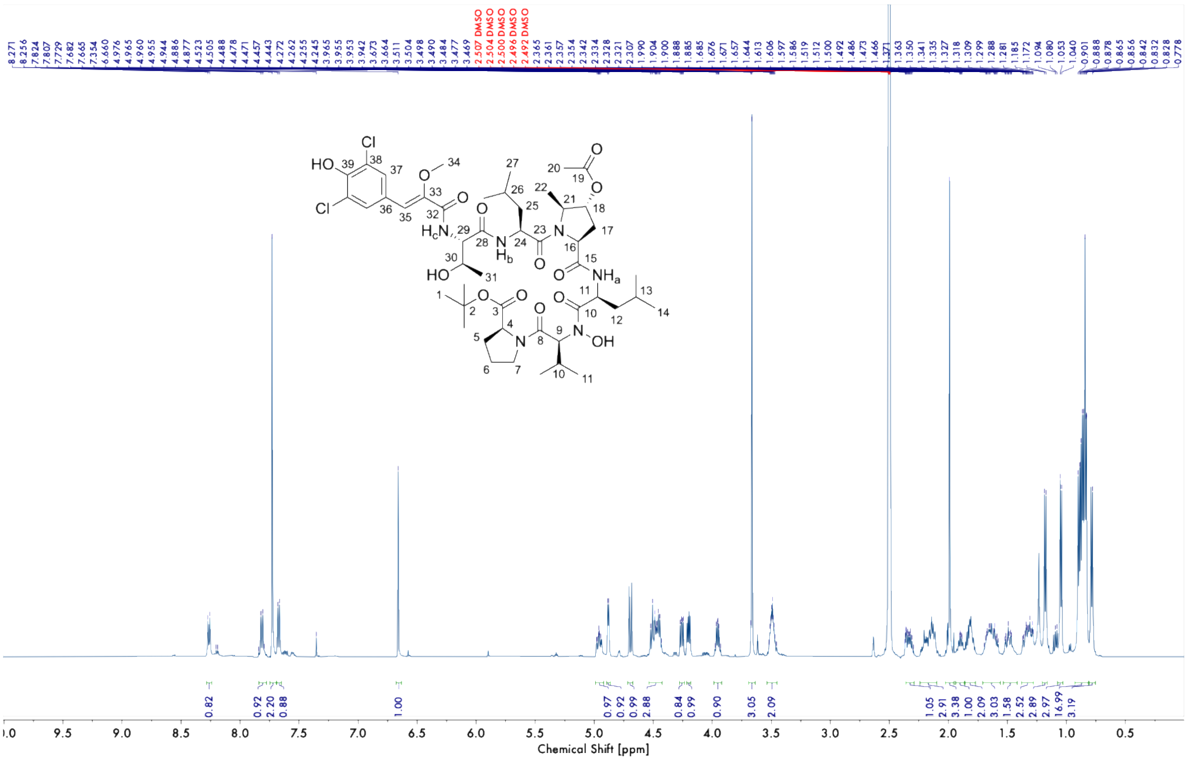

^{13}C NMR (126 MHz, DMSO-d_6, **27**):

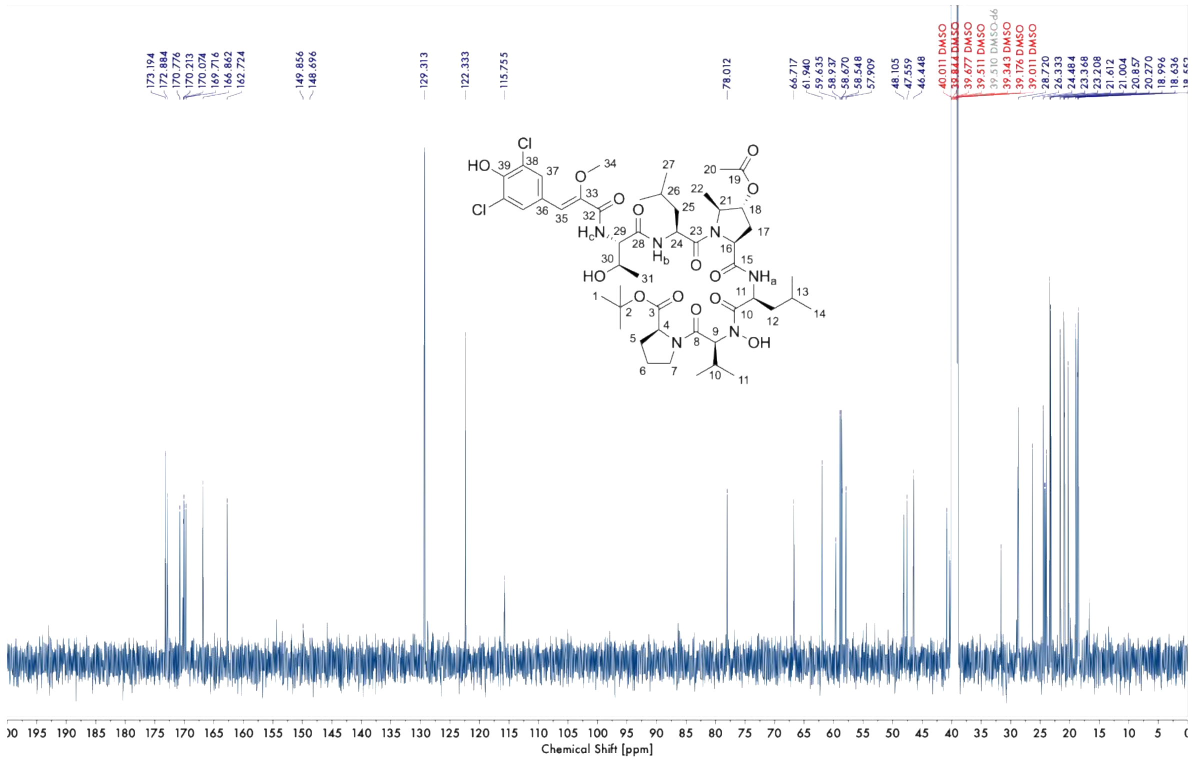

^{13}C NMR (126 MHz, DMSO-d_6 + 0.4 vol% TFA, **27**):

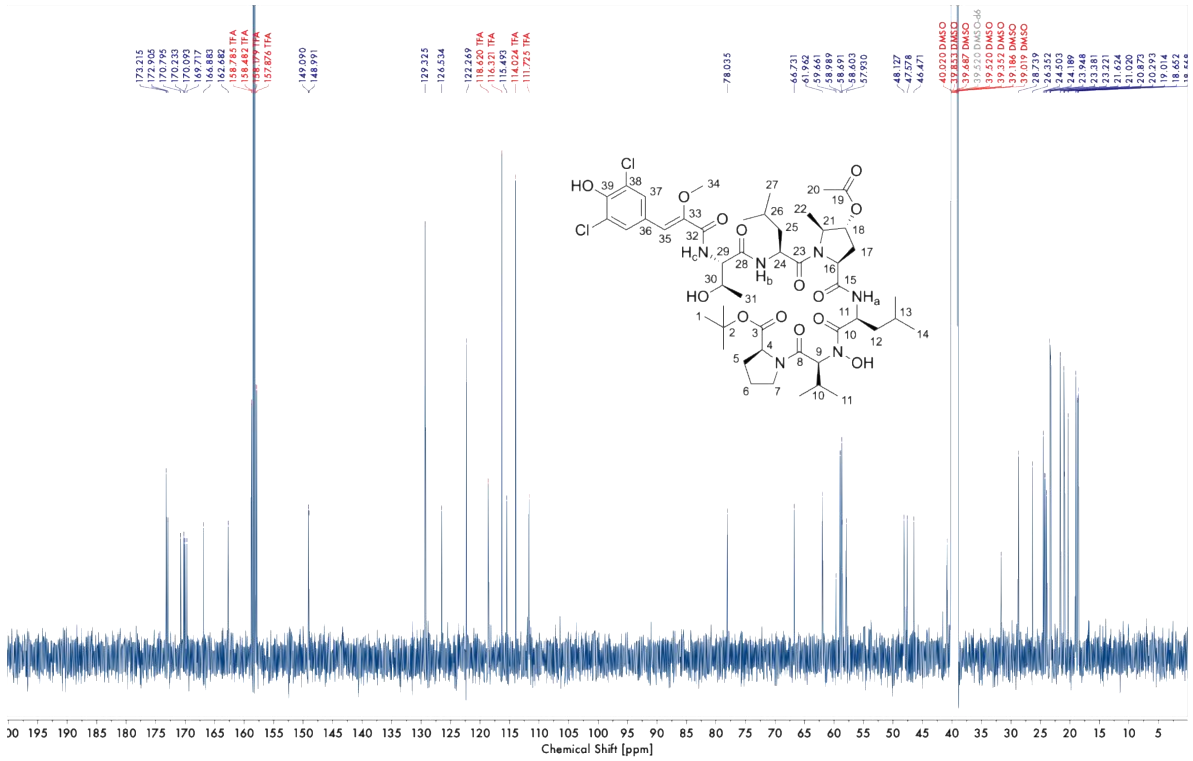

(^{1}H,^{1}H)-COSY (DMSO-*d*$_6$, **27**):

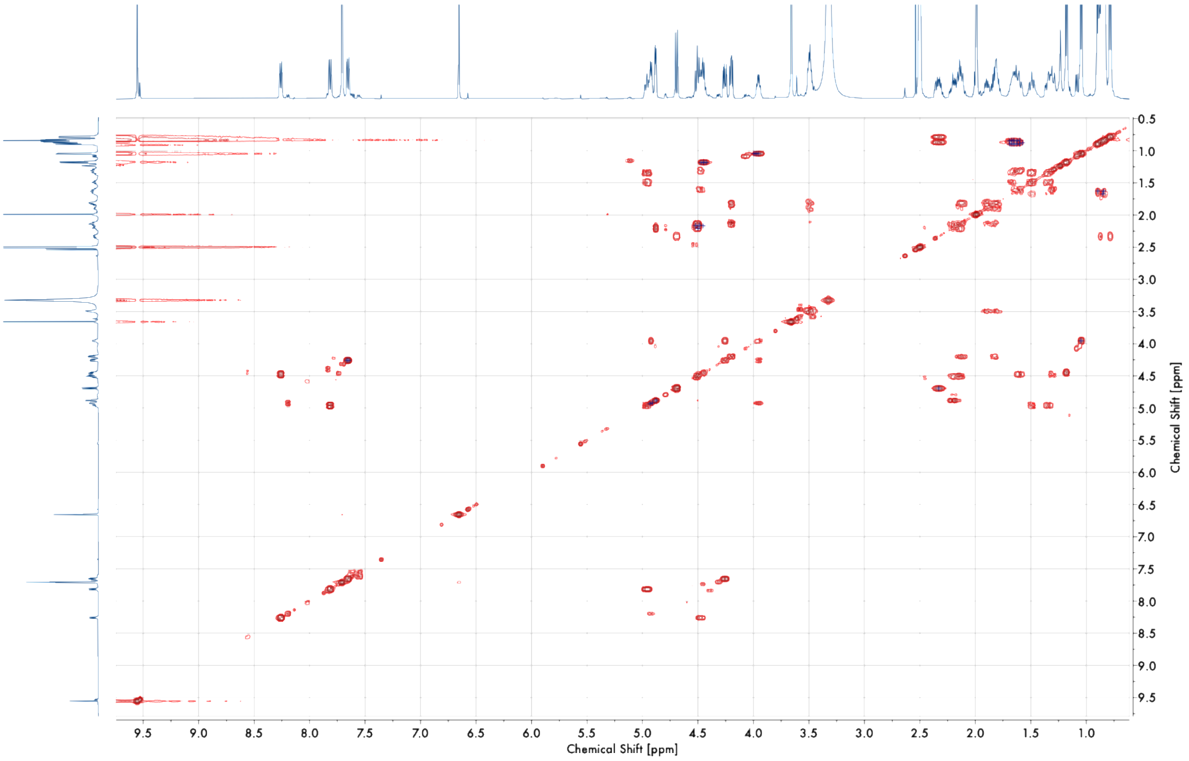

(^{1}H,^{13}C)-HSQC (DMSO-*d*$_6$, **27**):

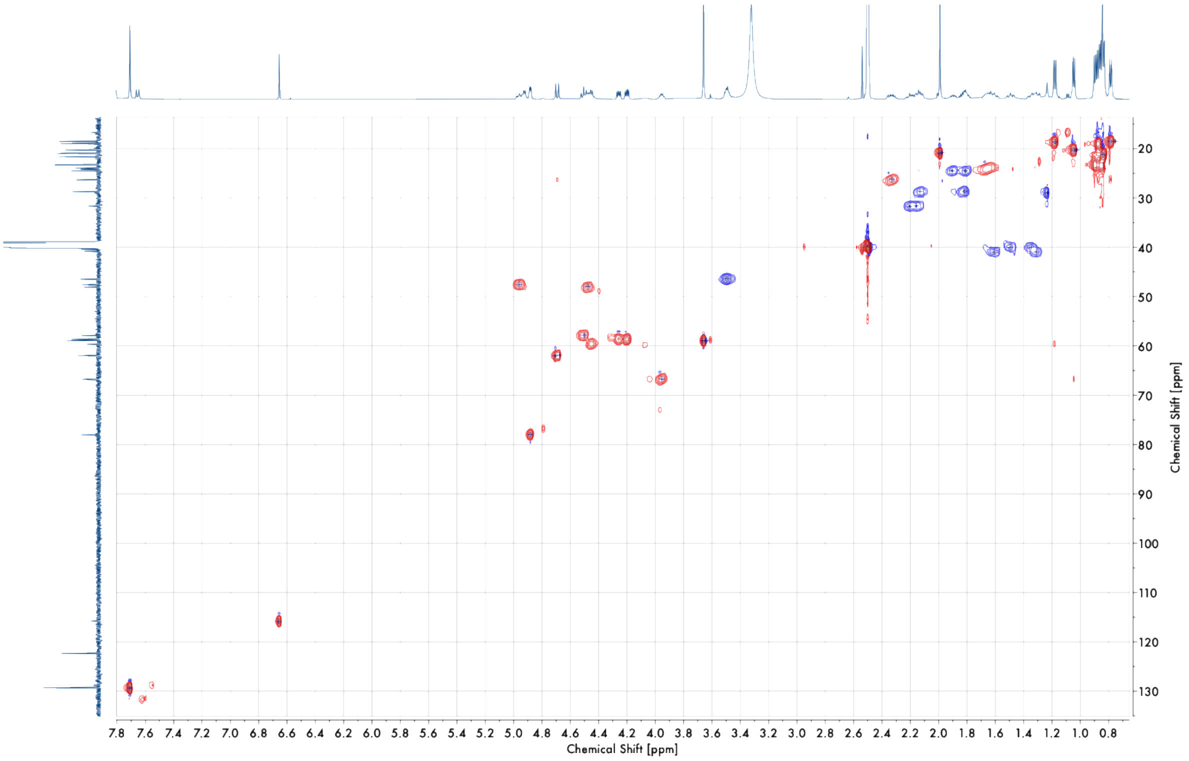

(^{1}H,^{13}C)-HMBC (DMSO-d_6, **27**):

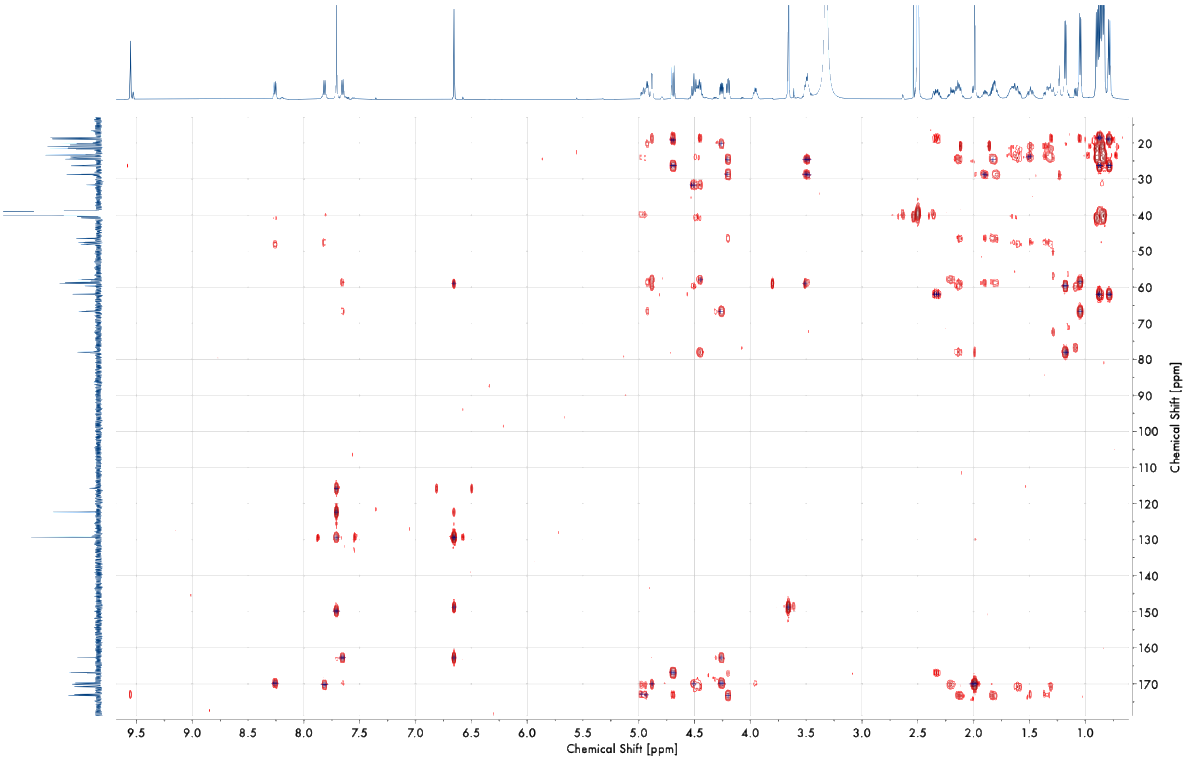

(^{1}H,^{1}H)-TOCSY (DMSO-d_6, **27**):

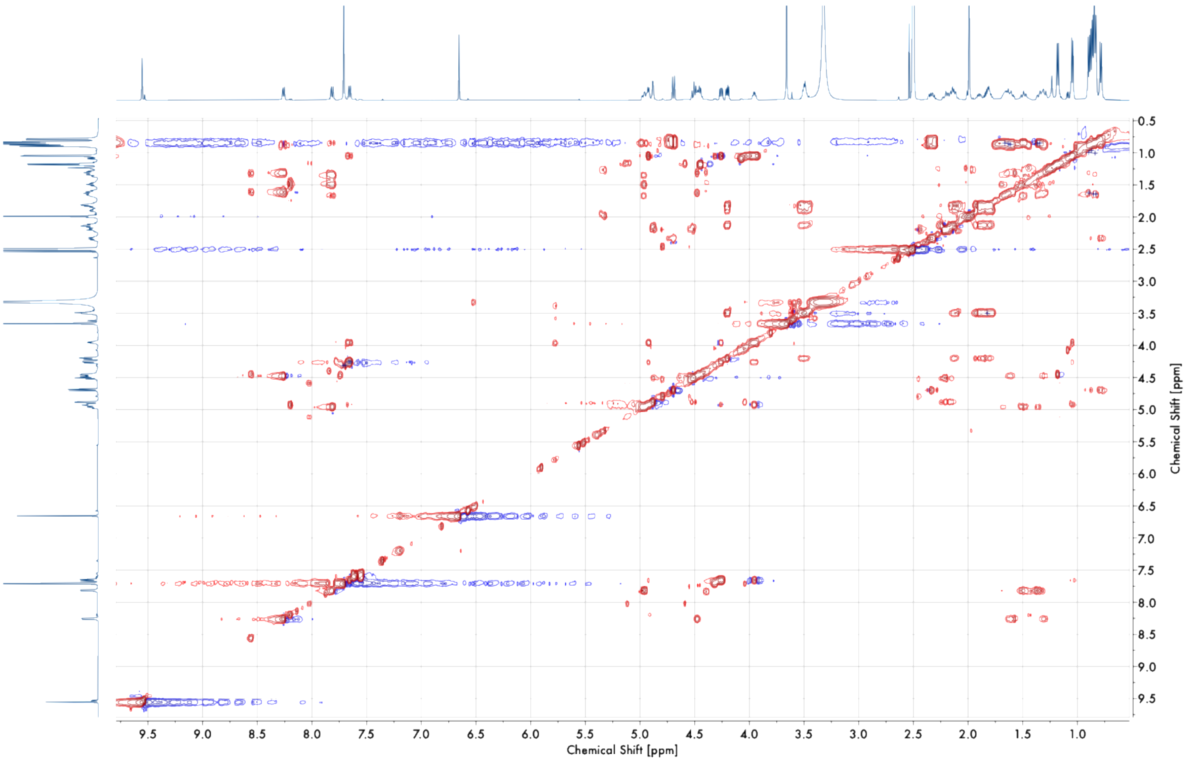

Bonnevillamide B [28]

^{1}H NMR (500 MHz, DMSO-d_6, **28**):

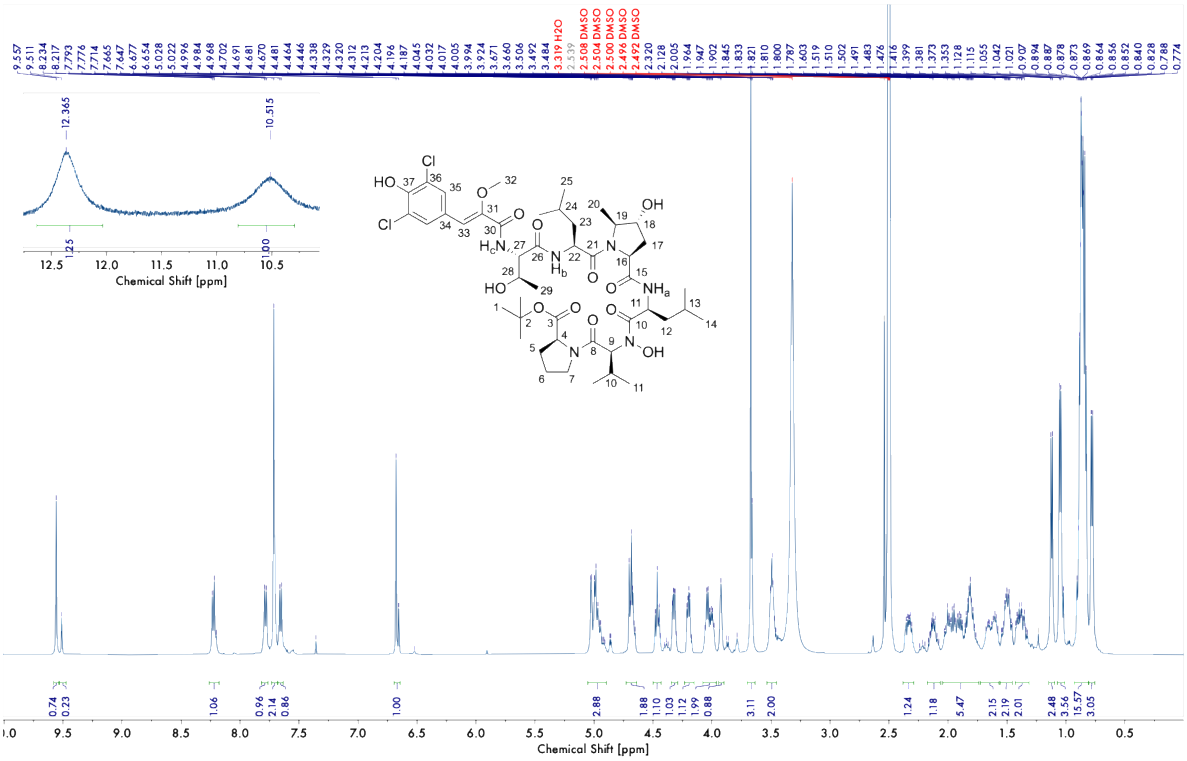

^{1}H NMR (500 MHz, DMSO-d_6 + 0.4 vol% TFA, **28**):

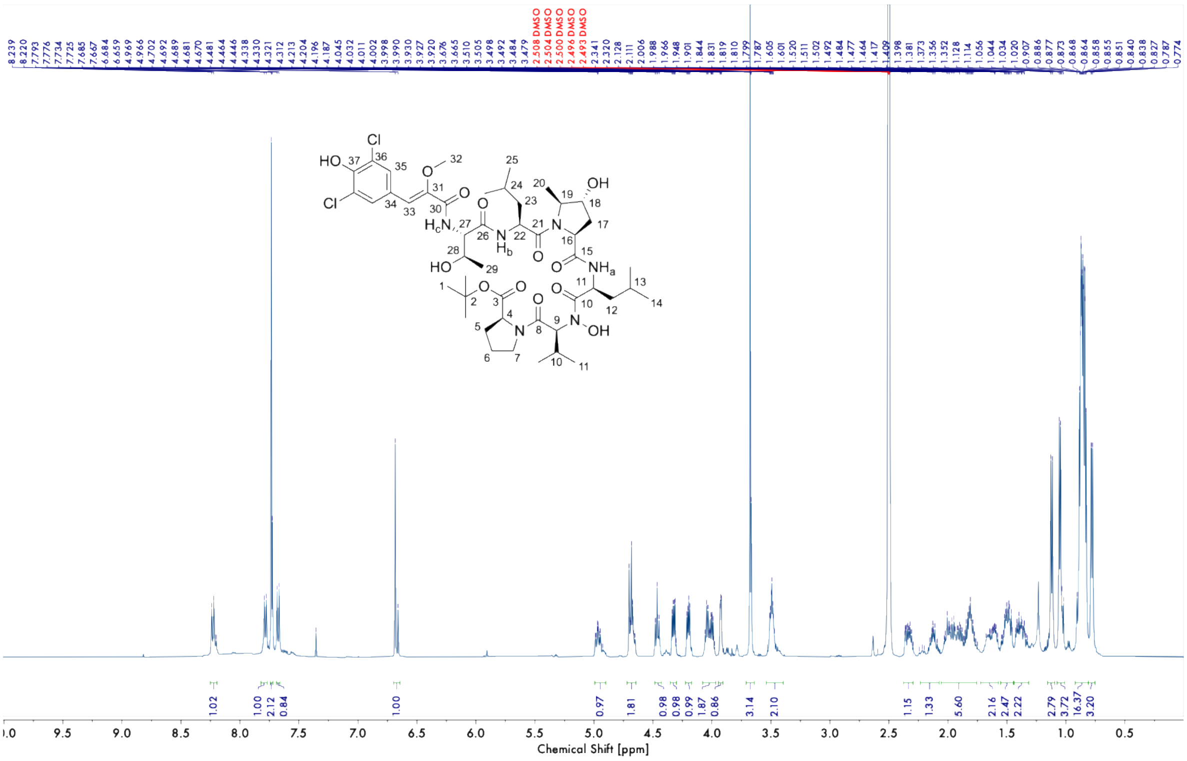

^{13}C NMR (126 MHz, DMSO-d_6, **28**):

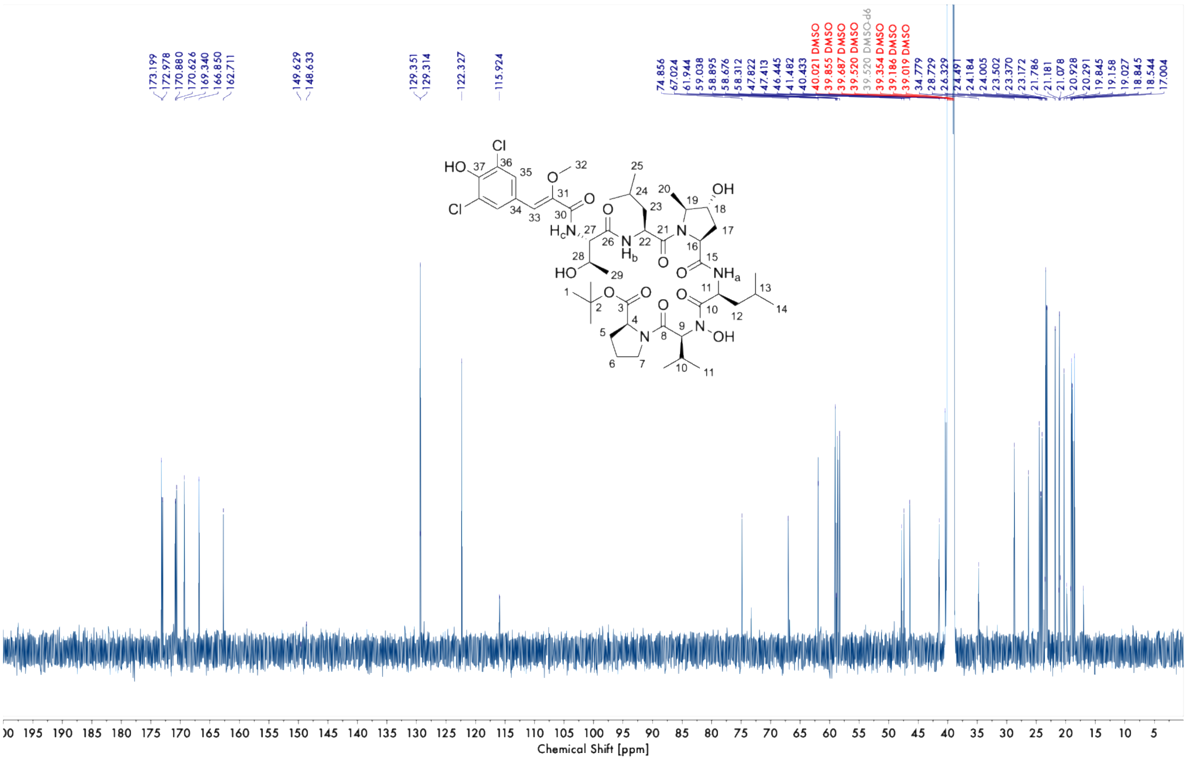

^{13}C NMR (126 MHz, DMSO-d_6 + 0.4 vol% TFA, **28**):

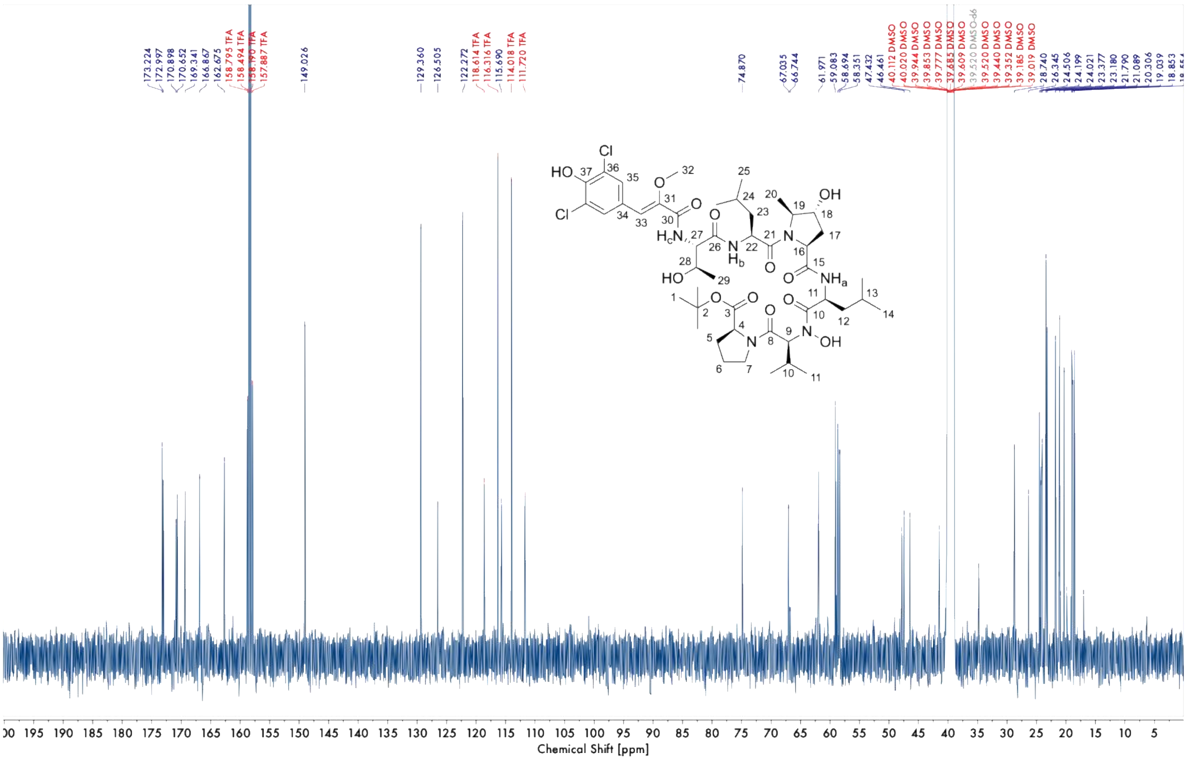

(^{1}H,^{1}H)-COSY (DMSO-d_6, **28**):

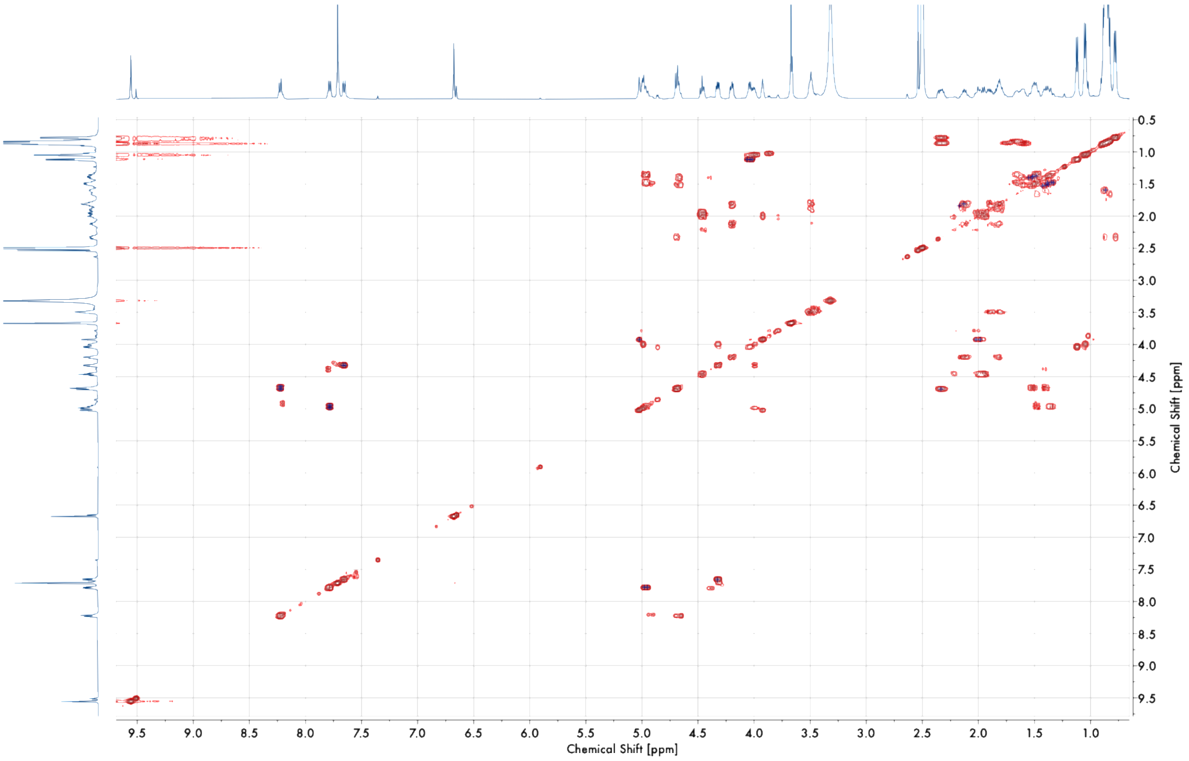

(^{1}H,^{13}C)-HSQC (DMSO-d_6, **28**):

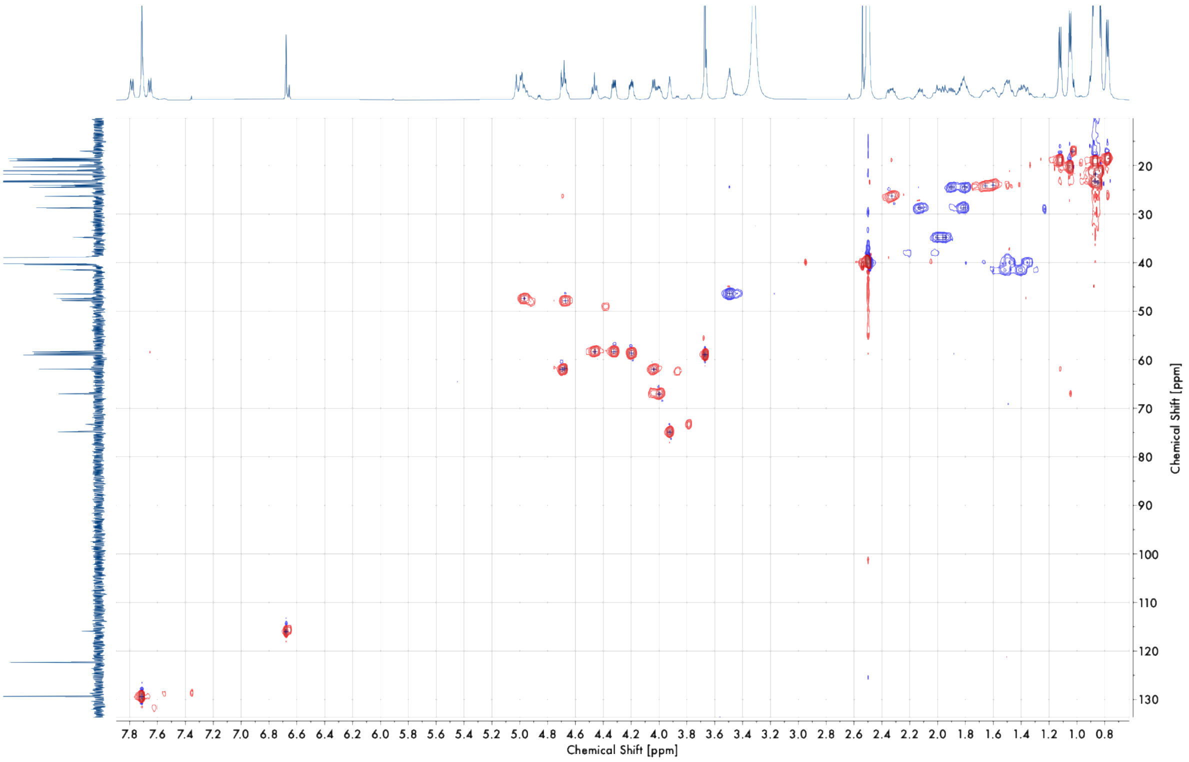

(^{1}H,^{13}C)-HMBC (DMSO-d_6, **28**):

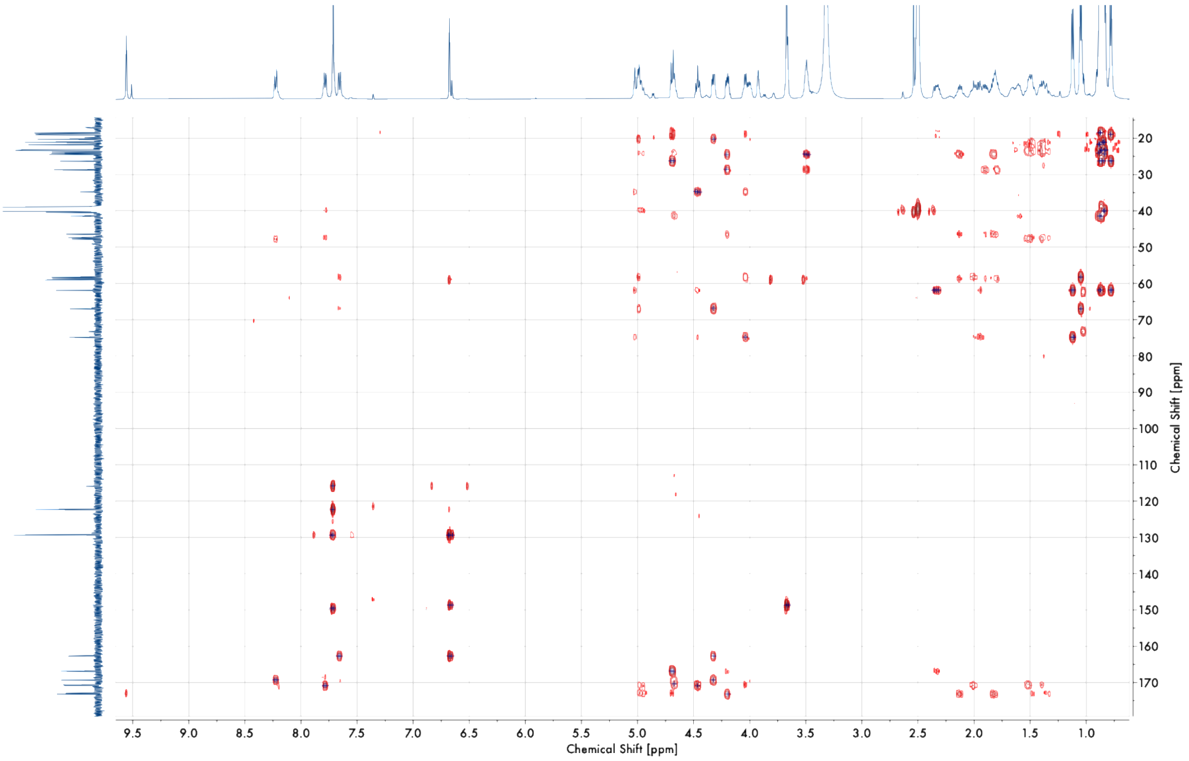

(^{1}H,^{1}H)-TOCSY (DMSO-d_6, **28**):

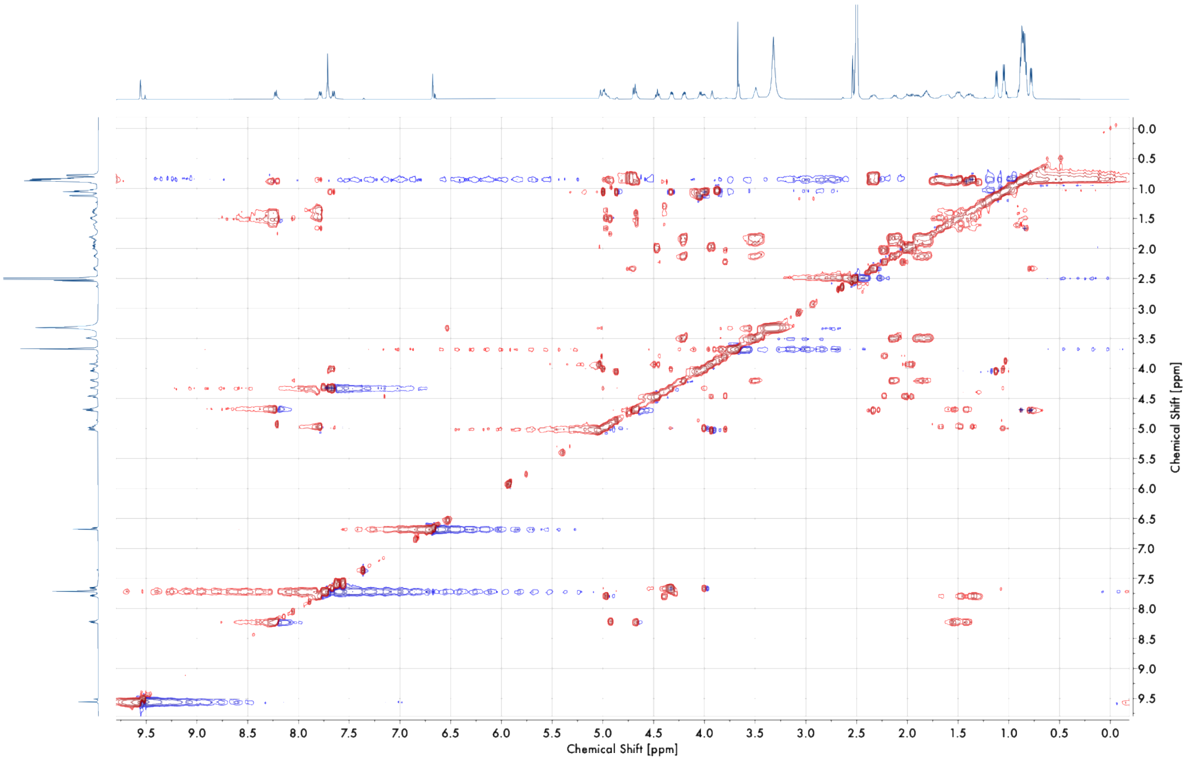

NMR Data Comparison of Authentic and Synthetic Bonnevillamides B and C

Table 2: Comparison of ^{1}H NMR chemical shifts for reported BvC[17] and synthetic **27** in the absence and presence of 0.4 vol% TFA (DMSO-d_6, 500 MHz; $\Delta\delta(^1H) = \delta(BvC)-\delta(\mathbf{27})$; δ and Δδ in ppm).

H-Atom	δ(BvC)	δ(**27**)	$\Delta\delta(^1H)$	δ(**27**+TFA)	$\Delta\delta(^1H)$
2-H (Pro)	4.21	4.20	0.01	4.20	0.01
3-H_a (Pro)	1.83	1.82	0.01	1.82	0.01
3-H_b (Pro)	2.14	2.13	0.01	2.13	0.01
4-H_a (Pro)	1.83	1.81	0.02	1.81	0.02
4-H_b (Pro)	1.91	1.90	0.01	1.90	0.01
5-H (Pro)	3.50	3.50	0.00	3.50	0.00
7-H (HyVal)	4.70	4.69	0.01	4.69	0.01
8-H (HyVal)	2.34	2.32	0.02	2.32	0.02
9-H (HyVal)	0.79	0.79	0.00	0.79	0.00
9-H' (HyVal)	0.88	0.87	0.01	0.87	0.01
11-H (Leu1)	4.97	4.96	0.01	4.96	0.01
12-H_a (Leu1)	1.37	1.32	0.05	1.32	0.05
12-H_b (Leu1)	1.50	1.49	0.01	1.49	0.01
13-H (Leu1)	1.67	1.67	0.00	1.67	0.00
14-H (Leu1)	0.83	0.83	0.00	0.83	0.00
14-H' (Leu1)	0.83	0.85	-0.02	0.85	-0.02
NH_a (Leu1)	7.82	7.82	0.00	7.82	0.00
16-H (Hyp)	4.51	4.51	0.00	4.51	0.00
17-H (MeHyPro)	2.19	2.17	0.02	2.17	0.02
18-H (MeHyPro)	4.89	4.88	0.01	4.88	0.01
20-H (MeHyPro)	2.00	1.99	0.01	2.00	0.00
21-H (MeHyPro)	4.49	4.45	0.04	4.45	0.04
22-H (MeHyPro)	1.18	1.18	0.00	1.18	0.00
24-H (Leu2)	4.49	4.48	0.01	4.48	0.01
25-H_a (Leu2)	1.31	1.32	-0.01	1.32	-0.01
25-Hb (Leu2)	1.62	1.63	-0.01	1.63	-0.01
26-H (Leu2)	1.62	1.63	-0.01	1.63	-0.01
27-H (Leu2)	0.82	0.83	-0.01	0.83	-0.01
27-H' (Leu2)	0.89	0.90	-0.01	0.90	-0.01
NH_b (Leu2)	8.26	8.26	0.00	8.26	0.00
29-H (Thr)	4.27	4.26	0.01	4.26	0.01
30-H (Thr)	3.97	3.95	0.02	3.95	0.02
31-H (Thr)	1.05	1.05	0.00	1.05	0.00
NH_c (Thr)	7.68	7.65	0.03	7.67	0.01
34-H (SacCl)	3.67	3.66	0.01	3.67	0.00
35-H (SacCl)	6.67	6.66	0.01	6.67	0.01
37-H (SacCl)	7.73	7.71	0.02	7.73	0.00

Table 3: Comparison of ^{13}C NMR chemical shifts for reported BvC[17] and synthetic **27** in the absence and presence of 0.4 vol% TFA (DMSO-d_6, 126 MHz; $\Delta\delta(^{13}C)$ = δ(BvC)-δ(**27**); δ and Δδ in ppm).

C-Atom	δ(BvC)	δ(**27**)	$\Delta\delta(^{13}C)$	δ(**27**+TFA)	$\Delta\delta(^{13}C)$
C-2 (Pro)	59.2	58.7	0.5	58.7	0.5
C-3 (Pro)	29.2	28.7	0.5	28.7	0.5
C-4 (Pro)	24.9	24.5	0.4	24.5	0.4
C-5 (Pro)	46.9	46.5	0.4	46.5	0.4
C-6 (HyVal)	167.3	166.9	0.4	166.9	0.4
C-7 (HyVal)	62.4	62.0	0.4	62.0	0.4
C-8 (HyVal)	26.9	26.3	0.6	26.4	0.5
C-9 (HyVal)	19.0	18.6	0.4	18.6	0.4
C-9' (HyVal)	19.5	19.0	0.5	19.0	0.5
C-11 (Leu1)	48.1	47.6	0.5	47.6	0.5
C-12 (Leu1)	40.3	40.4	-0.1	40.4	-0.1
C-13 (Leu1)	24.5	24.2	0.3	24.2	0.3
C-14 (Leu1)	22.1	21.0	1.1	21.0	1.1
C-14' (Leu1)	22.3	23.4	-1.1	23.4	-1.1
C-15 (MeHyPro)	170.7	170.2	0.5	170.2	0.5
C-16 (MeHyPro)	58.4	57.9	0.5	57.9	0.5
C-17 (MeHyPro)	32.2	31.7	0.5	31.7	0.5
C-18 (MeHyPro)	78.5	78.0	0.5	78.0	0.5
C-19 (MeHyPro)	170.5	170.1	0.4	170.1	0.4
C-20 (MeHyPro)	21.4	20.9	0.5	20.9	0.5
C-21 (MeHyPro)	60.1	59.7	0.4	59.7	0.4
C-22 (MeHyPro)	19.1	18.7	0.4	18.7	0.4
C-24 (Leu2)	48.6	48.1	0.5	48.1	0.5
C-25 (Leu2)	41.4	40.8	0.6	40.9	0.5
C-26 (Leu2)	24.7	23.9	0.8	23.9	0.8
C-27 (Leu2)	21.5	21.6	-0.1	21.6	-0.1
C-27' (Leu2)	23.7	23.2	0.5	23.2	0.5
C-28 (Thr)	170.2	169.7	0.5	169.7	0.5
C-29 (Thr)	59.1	58.6	0.5	58.6	0.5
C-30 (Thr)	67.1	66.7	0.4	66.7	0.4
C-31 (Thr)	20.7	20.3	0.4	20.3	0.4
C-32 (SacCl)	163.1	162.7	0.4	162.7	0.4
C-33 (SacCl)	149.5	148.7	0.8	149.0	0.5
C-34 (SacCl)	59.4	59.0	0.4	59.0	0.4
C-35 (SacCl)	115.9	115.8	0.1	115.5	0.3
C-36 (SacCl)	129.7	129.3	0.4	129.3	0.4
C-37 (SacCl)	129.7	129.3	0.4	129.3	0.4
C-38 (SacCl)	122.6	122.3	0.3	122.3	0.3
C-39 (SacCl)	149.5	149.9	-0.4	149.1	0.4

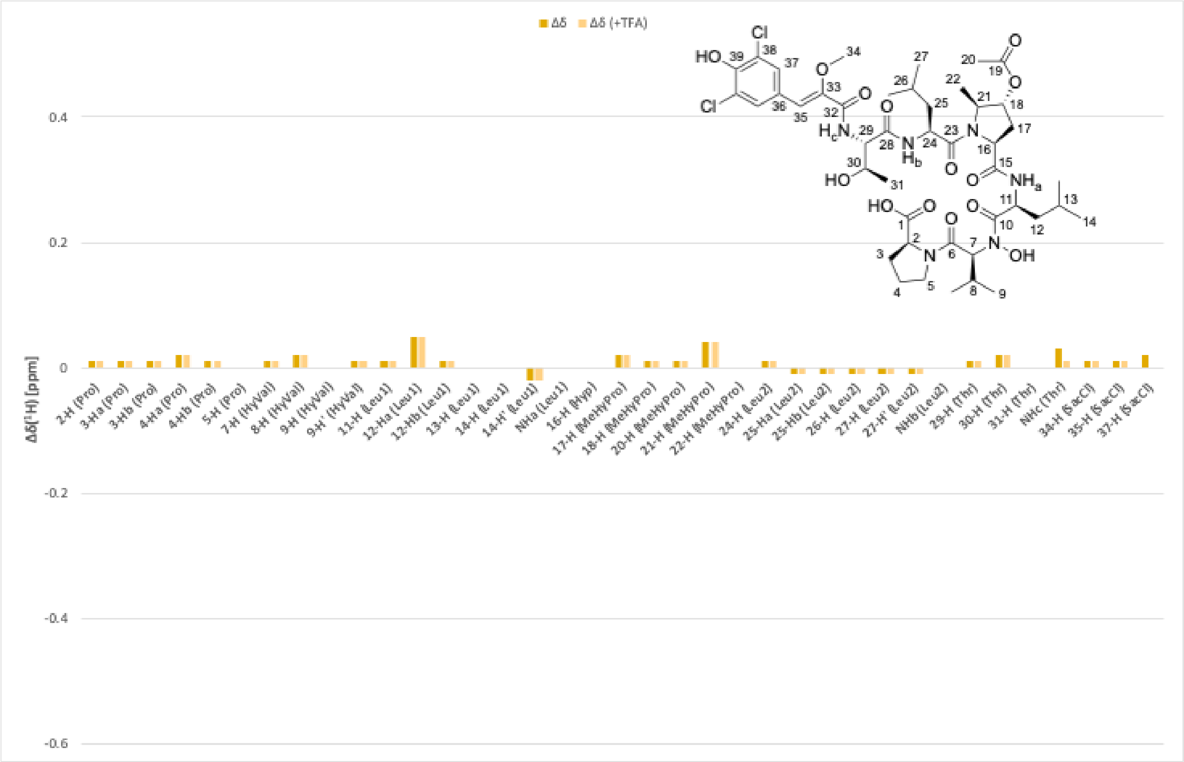

Figure 1: Comparison of ^{1}H NMR chemical shifts for reported BvC[17] and synthetic **27** in the absence and presence of 0.4 vol% TFA (DMSO-d_6, 500 MHz; $\Delta\delta(^{1}H) = \delta(BvC)-\delta(\mathbf{27})$; δ and Δδ in ppm).

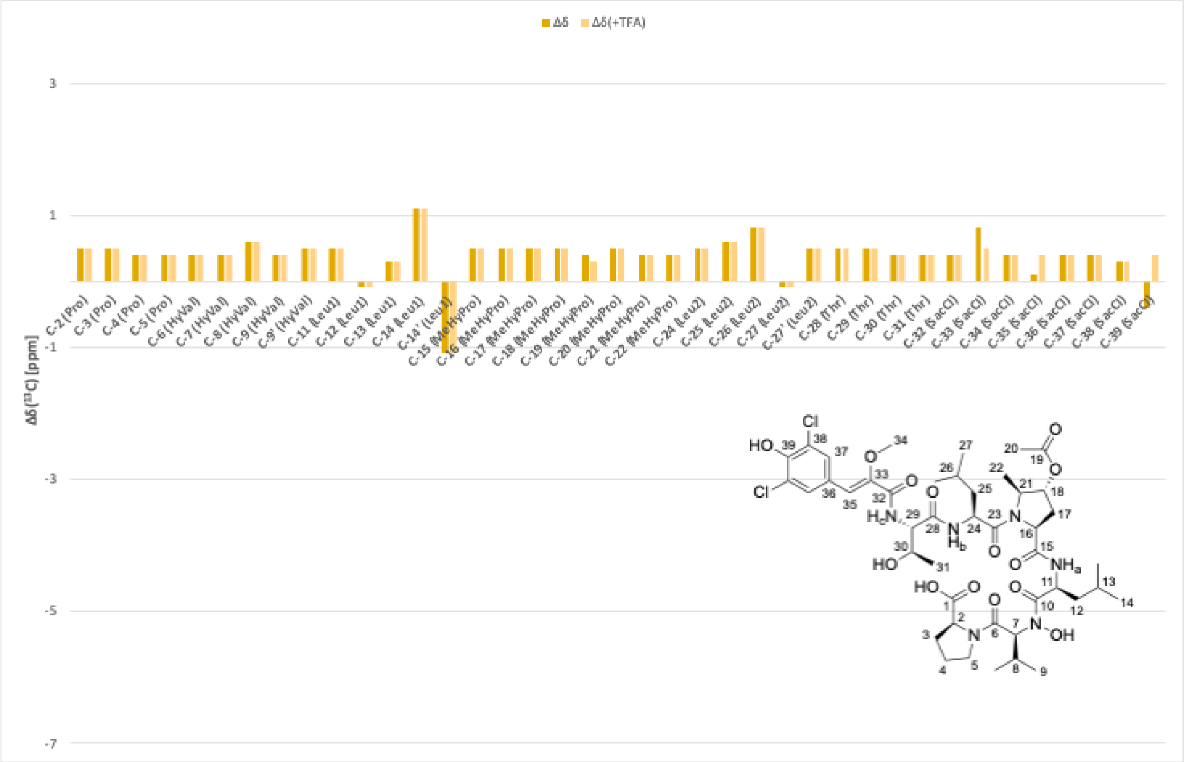

Figure 2: Comparison of ^{13}C NMR chemical shifts for reported BvC[17] and synthetic **27** in the absence and presence of 0.4 vol% TFA (DMSO-d_6, 126 MHz; $\Delta\delta(^{13}C) = \delta(BvC)-\delta(\mathbf{27})$; δ and Δδ in ppm).

Table 4: Comparison of ^{1}H NMR chemical shifts for reported BvB[17] and synthetic **28** (DMSO-d_6, 500 MHz; $\Delta\delta(^{1}H) = \delta(BvB) - \delta(\mathbf{28})$).

H-Atom	δ(BvB) [ppm]	δ(**28**) [ppm]	Δδ(^{1}H) [ppm]
2-H (Pro)	4.18	4.20	-0.02
3-H_a (Pro)	1.78	1.81	-0.03
3-H_b (Pro)	2.10	2.13	-0.03
4-H_a (Pro)	1.80	1.81	-0.01
4-H_b (Pro)	1.90	1.90	0.00
5-H (Pro)	3.47	3.49	-0.02
7-H (HyVal)	4.66	4.69	-0.03
8-H (HyVal)	2.30	2.33	-0.03
9-H (HyVal)	0.76	0.78	-0.02
9-H' (HyVal)	0.83	0.87	-0.04
11-H (Leu1)	4.66	4.94	-0.28
12-H_a (Leu1)	1.38	1.38	0.00
12-H_b (Leu1)	1.47	1.50	-0.03
13-H (Leu1)	1.57	1.65	-0.08
14-H (Leu1)	0.80	0.83	-0.03
14-H' (Leu1)	0.84	0.87	-0.03
NH_a (Leu1)	8.20	7.78	0.42
16-H (Hyp)	4.44	4.46	-0.02
17-H (MeHyPro)	1.95	1.98	-0.03
18-H (MeHyPro)	3.90	3.92	-0.02
19-H (MeHyPro)	4.01	4.04	-0.03
20-H (MeHyPro)	1.10	1.12	-0.02
22-H (Leu2)	4.95	4.67	0.28
23-H_a (Leu2)	1.34	1.38	-0.04
23-H_b (Leu2)	1.46	1.50	-0.04
24-H (Leu2)	1.64	1.61	0.04
25-H (Leu2)	0.83	0.87	-0.04
25-H' (Leu2)	0.85	0.87	-0.02
NH_b (Leu2)	7.76	8.22	-0.46
27-H (Thr)	4.30	4.32	-0.02
28-H (Thr)	3.97	4.00	-0.03
29-H (Thr)	1.02	1.05	-0.03
NH_c (Thr)	7.65	7.66	-0.01
32-H (SacCl)	3.65	3.67	-0.02
33-H (SacCl)	6.66	6.68	-0.02
35-H (SacCl)	7.70	7.71	-0.01

Table 5: Comparison of ^{13}C NMR chemical shifts for reported BvB[17] and synthetic **28** (DMSO-d_6, 126 MHz; $\Delta\delta(^1H) = \delta(BvB)-\delta(\mathbf{28})$).

C-Atom	δ(BvB) [ppm]	δ(**28**) [ppm]	$\Delta\delta(^{13}C)$ [ppm]
C-1 (Pro)	171.1	173.2	-2.1
C-2 (Pro)	59.1	58.7	0.4
C-3 (Pro)	29.2	28.7	0.5
C-4 (Pro)	25.0	24.5	0.5
C-5 (Pro)	46.9	46.5	0.4
C-6 (HyVal)	167.3	166.9	0.4
C-7 (HyVal)	62.4	61.9	0.5
C-8 (HyVal)	26.8	26.3	0.5
C-9 (HyVal)	19.0	18.5	0.5
C-9' (HyVal)	19.5	19.0	0.5
C-10 (Leu1)	167.3	173	-5.7
C-11 (Leu1)	48.3	47.4	0.9
C-12 (Leu1)	41.9	40.4	1.5
C-13 (Leu1)	24.5	24.2	0.3
C-14 (Leu1)	21.5	21.1	0.4
C-14' (Leu1)	23.8	23.4	0.4
C-15 (MeHyPro)	171.3	170.9	0.4
C-16 (MeHyPro)	58.8	58.3	0.5
C-17 (MeHyPro)	35.2	34.8	0.4
C-18 (MeHyPro)	75.3	74.9	0.4
C-19 (MeHyPro)	62.4	61.9	0.5
C-20 (MeHyPro)	19.3	18.8	0.5
C-21 (Leu2)	173.7	170.6	3.1
C-22 (Leu2)	47.9	47.8	0.1
C-23 (Leu2)	40.5	41.5	-1.0
C-24 (Leu2)	24.7	24.0	0.7
C-25 (Leu2)	22.2	21.8	0.4
C-25' (Leu2)	23.6	23.2	0.4
C-26 (Thr)	169.8	169.3	0.5
C-27 (Thr)	58.8	58.3	0.5
C-28 (Thr)	67.5	67.0	0.5
C-29 (Thr)	20.1	20.3	-0.2
C-30 (SacCl)	163.1	162.7	0.4
C-31 (SacCl)	149.5	148.6	0.9
C-32 (SacCl)	59.5	59.0	0.5
C-33 (SacCl)	116.1	115.9	0.2
C-34 (SacCl)	126.9	129.3	-2.4
C-35 (SacCl)	129.8	129.4	0.4
C-36 (SacCl)	122.7	122.3	0.4
C-37 (SacCl)	149.5	149.6	-0.1

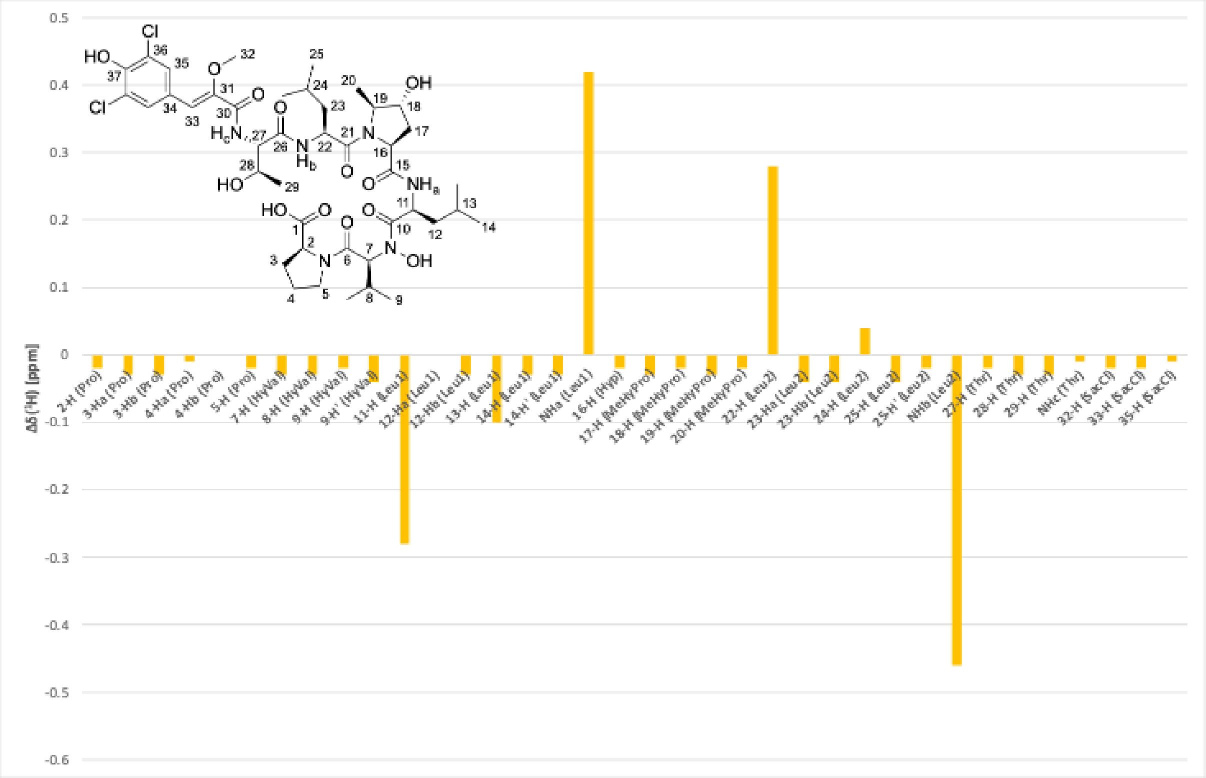

Figure 3: Comparison of ^{1}H NMR chemical shifts for reported BvB[17] and synthetic **28** (DMSO-d_6, 500 MHz; $\Delta\delta(^1H) = \delta(BvB)-\delta(\mathbf{28})$).

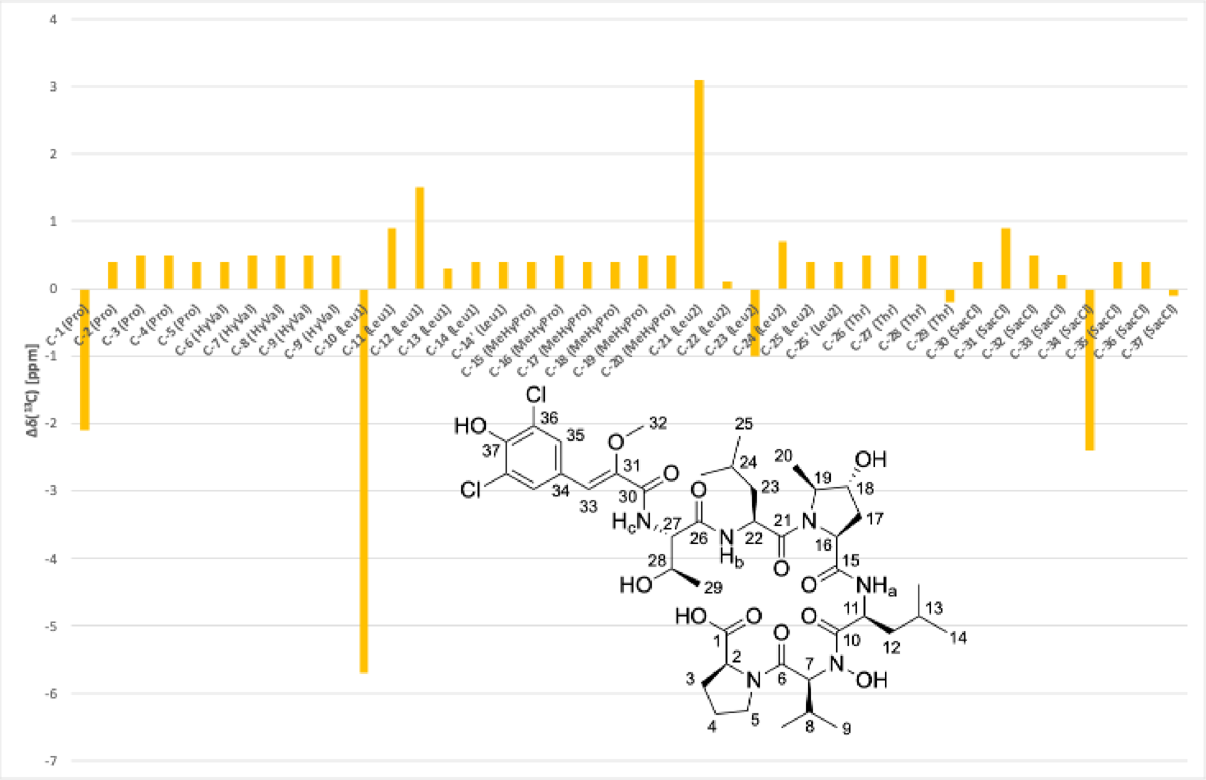

Figure 4: Comparison of ^{13}C NMR chemical shifts for reported BvB[17] and synthetic **28** (DMSO-d_6, 126 MHz; $\Delta\delta(^1H) = \delta(BvB)-\delta(\mathbf{28})$).

Inconsistencies in the assignment of NMR signals in the original publication[17] for the isolation and structure elucidation of the bonnevillamides

Mar. Drugs **2017**, *15*, 195 6 of 11

Table 1. *Cont.*

Residue	Position	Bonnevillamide A (1)		Bonnevillamide B (2)		Bonnevillamide C (3)	
		δ_C [a]	δ_H (J in Hz) [b]	δ_C [a]	δ_H (J in Hz) [b]	δ_C [c]	δ_H (J in Hz) [b]
Leu 1	1	173.8 C	-	173.7 C	-	[d]	-
	2	48.0 CH	4.98, m	47.9 CH	4.95, m	48.6 CH	4.49, m
	3	40.1 CH_2	1.37, m; 1.52, m	40.5 CH_2	1.34, m; 1.46, m	41.4 CH_2	1.31, m; 1.62, m
	4	24.4 CH	1.66, m	24.7 CH	1.64, m	24.7 CH	1.62, m
	5	22.1 CH_3	0.83, overlap	23.6 CH_3	0.85, overlap	21.5 CH_3	0.82, overlap
	6	22.4 CH_3	0.83, overlap	22.2 CH_3	0.83, overlap	23.7 CH_3	0.89, d (6.9)
	NH	-	7.83, d (8.4)		7.76, d (6.8)	-	8.26, d (8.0)
HMPro	1	170.7 C	-	171.3 C	-	170.7 C	-
	2	58.4 CH	4.43, overlap	58.8 CH	4.44, m	58.4 CH	4.51, overlap
	3	32.1 CH_2	2.15, m	35.2 CH_2	1.92, m; 1.98, m	32.2 CH_2	2.16, m; 2.22 m
	4	78.5 CH	4.88, m	75.3 CH	3.90, brs	78.5 CH	4.89, d
	5	60.1 CH	4.49, m	62.4 CH	4.01, m	60.1 CH	4.49, overlap
	6	19.1 CH_3	1.18, d (6.8)	19.3 CH_3	1.10, d (6.7)	19.1 CH_3	1.18, d (6.9)
	7	170.5 C	-	-	-	170.5 C	-
	8	21.3 CH_3	1.99, s	-	-	21.4 CH_3	2.00, s
Leu 2	1	171.2 C	-	167.3 C	-	[d]	-
	2	48.6 CH	4.49, m	48.3 CH	4.66, m	48.1 CH	4.97, m
	3	41.3 CH_2	1.31, m; 1.62, m	41.9 CH_2	1.38, m; 1.47, m	40.3 CH_2	1.37, m; 1.50, m
	4	24.7 CH	1.62, m	24.5 CH	1.57, m	24.5 CH	1.67, m
	5	21.5 CH_3	0.82, overlap	23.8 CH_3	0.84, overlap	22.1 CH_3	0.83, overlap
	6	23.7 CH_3	0.88, d (6.9)	21.5 CH_3	0.80, overlap	22.3 CH_3	0.83, overlap
	NH	-	8.25, d (8.0)	-	8.20, d (7.6)	-	7.82, d (8.6)
N-OH-Val	1	168.3 C	-	167.3 C	-	167.3 C	-
	2	61.2 CH	4.43, m	62.4 CH	4.66, m	62.4 CH	4.70, d (10.5)
	3	26.1 CH_2	2.31, m	26.8 CH	2.30, m	26.9 CH_2	2.34, m
	4	18.9 CH_3	0.77, d (6.7)	19.0 CH_3	0.76, d (7.0)	19.0 CH_3	0.79, d (6.7)
	5	19.7 CH_3	0.84, overlap	19.5 CH_3	0.83, overlap	19.5 CH_3	0.88, d (6.6)
MACME/Pro	1	171.6 C	-	171.1 C	-	[d]	-
	2	56.2 CH	4.49, m	59.1 CH	4.18, m	59.2 CH	4.21, dd (4.6, 8.7)
	3	27.9 CH_2	2.69, m; 1.72, m	29.2 CH_2	1.78, m; 2.10, m	29.2 CH_2	1.83, m; 2.14, m
	4	57.3 CH	4.36, m	25.0 CH_2	1.80, m; 1.90, m	24.9 CH_2	1.91, m; 1.83, m
	5	22.4 CH_3	1.47, d (6.2)	46.9 CH_2	3.47, m	46.9 CH_2	3.50, m
	6	52.4 CH_3	3.67, s	-	-	-	-

[a], ^{13}C spectrum was recorded in DMSO-d_6 in 500 MHz NMR; [b], ^{1}H spectrum was recorded in DMSO-d_6 in 600 MHz NMR; [c], The carbon chemical shifts were deduced by HSQC and HMBC experiments; [d], Carbon signals were not detected.

Obviously the NMR signals of Leu1 and Leu2 are differently assigned in the Bonnevillamides B and C

Table 6: Comparison of ^{1}H NMR chemical shifts for reported BvB[17] and synthetic **28** in the absence and presence of 0.4 vol% TFA with **revised assignments** (DMSO-d_6, 500 MHz; $\Delta\delta(^1H) = \delta(BvB)-\delta(\mathbf{28})$).

H-Atom	δ(BvB)	δ(**28**)	$\Delta\delta(^1H)$	δ(**28**+TFA)	$\Delta\delta(^1H)$
2-H (Pro)	4.18	4.20	-0.02	4.20	-0.02
3-H_a (Pro)	1.78	1.81	-0.03	1.81	-0.03
3-H_b (Pro)	2.10	2.13	-0.03	2.13	-0.03
4-H_a (Pro)	1.80	1.81	-0.01	1.81	-0.01
4-H_b (Pro)	1.90	1.90	0.00	1.90	0.00
5-H (Pro)	3.47	3.49	-0.02	3.49	-0.02
7-H (HyVal)	4.66	4.69	-0.03	4.69	-0.03
8-H (HyVal)	2.30	2.33	-0.03	2.33	-0.03
9-H (HyVal)	0.76	0.78	-0.02	0.78	-0.02
9-H' (HyVal)	0.83	0.87	-0.04	0.87	-0.04
11-H (Leu1)	4.66	4.67	-0.01	4.67	-0.01
12-H_a (Leu1)	1.38	1.38	0.00	1.38	0.00
12-H_b (Leu1)	1.47	1.50	-0.03	1.50	-0.03
13-H (Leu1)	1.57	1.65	-0.08	1.65	-0.08
14-H (Leu1)	0.80	0.83	-0.03	0.83	-0.03
14-H' (Leu1)	0.84	0.87	-0.03	0.87	-0.03
NH_a (Leu1)	8.20	8.22	-0.02	8.22	-0.02
16-H (Hyp)	4.44	4.46	-0.02	4.46	-0.02
17-H (MeHyPro)	1.95	1.98	-0.03	1.98	-0.03
18-H (MeHyPro)	3.90	3.92	-0.02	3.92	-0.02
19-H (MeHyPro)	4.01	4.04	-0.03	4.04	-0.03
20-H (MeHyPro)	1.10	1.12	-0.02	1.12	-0.02
22-H (Leu2)	4.95	4.94	0.01	4.97	-0.02
23-H_a (Leu2)	1.34	1.38	-0.04	1.38	-0.04
23-H_b (Leu2)	1.46	1.50	-0.04	1.50	-0.04
24-H (Leu2)	1.64	1.61	0.03	1.67	-0.03
25-H (Leu2)	0.83	0.87	-0.04	0.87	-0.04
25-H' (Leu2)	0.85	0.87	-0.02	0.87	-0.02
NH_b (Leu2)	7.76	7.78	-0.02	7.78	-0.02
27-H (Thr)	4.30	4.32	-0.02	4.32	-0.02
28-H (Thr)	3.97	4.00	-0.03	4.00	-0.03
29-H (Thr)	1.02	1.05	-0.03	1.05	-0.03
NH_c (Thr)	7.65	7.66	-0.01	7.66	-0.01
32-H (SacCl)	3.65	3.67	-0.02	3.68	-0.03
33-H (SacCl)	6.66	6.68	-0.02	6.68	-0.02
35-H (SacCl)	7.70	7.71	-0.01	7.73	-0.03

Table 7: Comparison of ^{13}C NMR chemical shifts for reported BvB[17] and synthetic **28** in the absence and presence of 0.4 vol% TFA with revised assignments (DMSO-d_6, 126 MHz; Δδ(^1H) = δ(BvB)-δ(**28**)).

C-Atom	δ(BvB)	δ(**28**)	Δδ(^{13}C)	δ(**28**+TFA)	Δδ(^{13}C)
C-1 (Pro)	171.1	173.2	-2.1	173.2	-2.1
C-2 (Pro)	59.1	58.7	0.4	58.7	0.4
C-3 (Pro)	29.2	28.7	0.5	28.7	0.5
C-4 (Pro)	25.0	24.5	0.5	24.5	0.5
C-5 (Pro)	46.9	46.5	0.4	46.5	0.4
C-6 (HyVal)	167.3	166.9	0.4	166.9	0.4
C-7 (HyVal)	62.4	61.9	0.5	62.0	0.4
C-8 (HyVal)	26.8	26.3	0.5	26.3	0.5
C-9 (HyVal)	19.0	18.5	0.5	18.6	0.4
C-9' (HyVal)	19.5	19.0	0.5	19.0	0.5
C-10 (Leu1)	167.3	170.6	-3.3	170.7	-3.4
C-11 (Leu1)	48.3	47.8	0.5	47.8	0.5
C-12 (Leu1)	41.9	41.5	0.4	41.5	0.4
C-13 (Leu1)	24.5	24.0	0.5	24.0	0.5
C-14 (Leu1)	21.5	21.1	0.4	21.1	0.4
C-14' (Leu1)	23.8	23.4	0.4	23.4	0.4
C-15 (MeHyPro)	171.3	170.9	0.4	170.9	0.4
C-16 (MeHyPro)	58.8	58.3	0.5	58.4	0.4
C-17 (MeHyPro)	35.2	34.8	0.4	34.8	0.4
C-18 (MeHyPro)	75.3	74.9	0.4	74.9	0.4
C-19 (MeHyPro)	62.4	61.9	0.5	62.0	0.4
C-20 (MeHyPro)	19.3	18.8	0.5	18.8	0.5
C-21 (Leu2)	173.6	173	0.6	173	0.6
C-22 (Leu2)	47.9	47.4	0.5	47.4	0.5
C-23 (Leu2)	40.5	40.4	0.1	40.3	0.2
C-24 (Leu2)	24.7	24.2	0.5	24.2	0.5
C-25 (Leu2)	22.2	21.8	0.4	21.8	0.4
C-25' (Leu2)	23.6	23.2	0.4	23.2	0.4
C-26 (Thr)	169.8	169.3	0.5	169.3	0.5
C-27 (Thr)	58.8	58.3	0.5	58.4	0.4
C-28 (Thr)	67.5	67.0	0.5	67.0	0.5
C-29 (Thr)	20.1	20.3	-0.2	20.3	-0.2
C-30 (SacCl)	163.1	162.7	0.4	162.7	0.4
C-31 (SacCl)	149.5	148.6	0.9	149.0	0.5
C-32 (SacCl)	59.5	59.0	0.5	59.1	0.4
C-33 (SacCl)	116.1	115.9	0.2	115.7	0.4
C-34 (SacCl)	126.9	129.3	-2.4	126.5	0.4
C-35 (SacCl)	129.8	129.4	0.4	129.4	0.4
C-36 (SacCl)	122.7	122.3	0.4	122.3	0.4
C-37 (SacCl)	149.5	149.6	-0.1	149.0	0.5

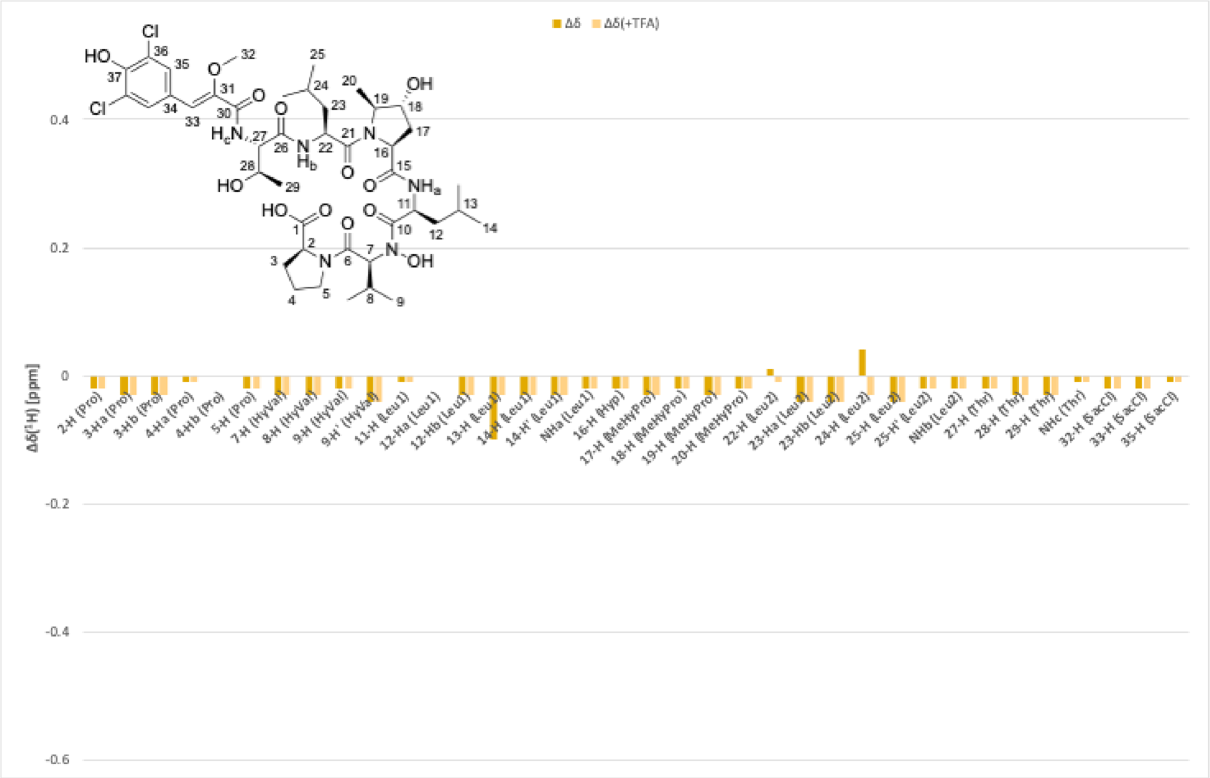

Figure 5: Comparison of ^1H NMR chemical shifts for reported BvB[17] and synthetic **28** in the absence (brown) and presence (yellow) of 0.4 vol% TFA with revised assignments (DMSO-d_6, 500 MHz; $\Delta\delta(^1H) = \delta(BvB) - \delta(\mathbf{28})$).

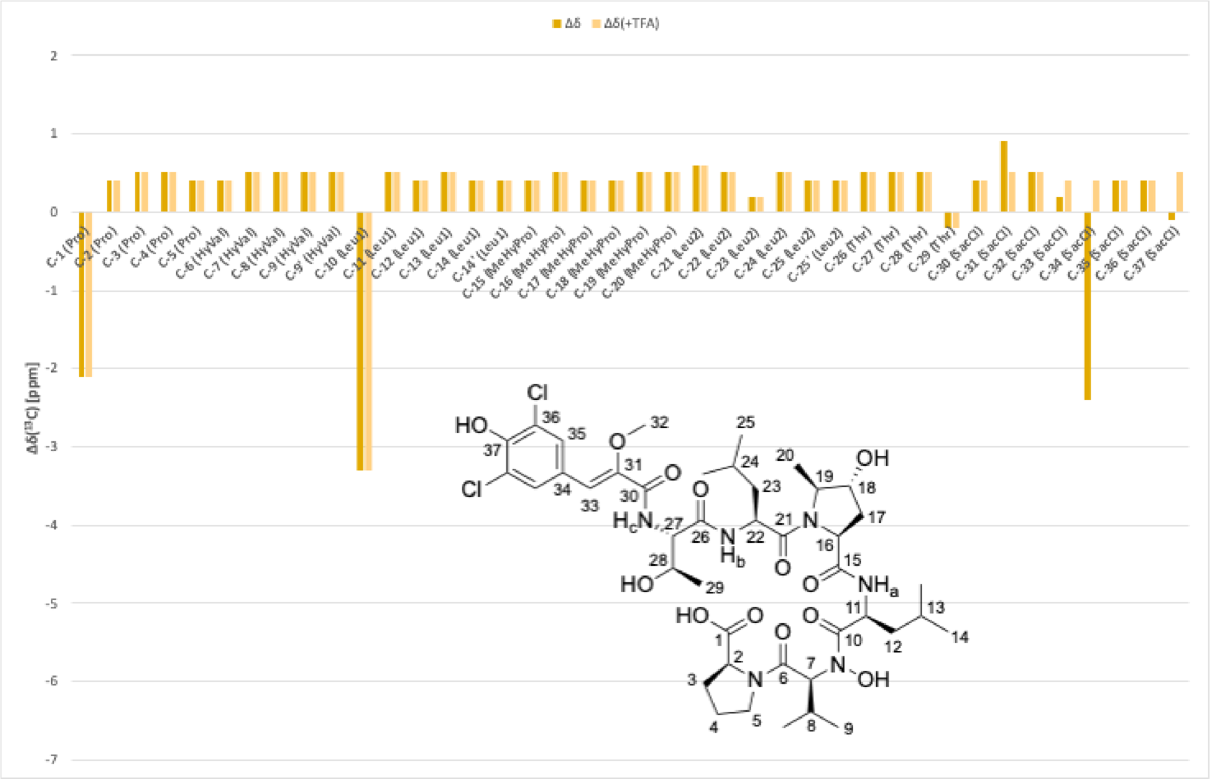

Figure 6: Comparison of ^{13}C NMR chemical shifts for reported BvB[17] and synthetic **28** in the absence (brown) and presence (yellow) of 0.4 vol% TFA with revised assignments (DMSO-d_6, 126 MHz; $\Delta\delta(^1H) = \delta(BvB) - \delta(\mathbf{28})$).

Bibliography

[1] Y. Nagai, N. Ito, I. Sultana, T. Sugai, *Tetrahedron* **2008**, *64*, 9599–9606.

[2] T. Ishizuka, T. Kunieda, *Tetrahedron Lett.* **1987**, *28*, 4185–4188.

[3] M. Hatano, K. Nishikawa, K. Ishihara, *J. Am. Chem. Soc.* **2017**, *139*, 8424–8427.

[4] A. Kiefer, C. D. Bader, J. Held, A. Esser, J. Rybniker, M. Empting, R. Müller, U. Kazmaier, *Chem. Eur. J.* **2019**, *25*, 8894–8902.

[5] K. Takeda, A. Akiyama, H. Nakamura, S. Takizawa, Y. Mizuno, H. Takayanagi, Y. Harigaya, *Synthesis* **1994**, *1994*, 1063–1066.

[6] J. N. Hernández, V. S. Martín, *J. Org. Chem.* **2004**, *69*, 3590–3592.

[7] C. Poock, M. Kalesse, *Org. Lett.* **2017**, *19*, 4536–4539.

[8] M. Biel, P. Deck, A. Giannis, H. Waldmann, *Chem. Eur. J.* **2006**, *12*, 4121–4143.

[9] C. Xiong, W. Wang, V. J. Hruby, *J. Org. Chem.* **2002**, *67*, 3514–3517.

[10] R. S. Navath, K. B. Pabbisetty, L. Hu, *Tetrahedron Lett.* **2006**, *47*, 389–393.

[11] S. Gagliardi, G. Nadler, E. Consolandi, C. Parini, M. Morvan, M.-N. Legave, P. Belfiore, A. Zocchetti, G. D. Clarke, I. James, P. Nambi, M. Gowen, C. Farina, *J. Med. Chem.* **1998**, *41*, 1568–1573.

[12] P. Seneci, I. Leger, M. Souchet, G. Nadler, *Tetrahedron* **1997**, *53*, 17097–17114.

[13] A. Fürstner, M. M. Domostoj, B. Scheiper, *J. Am. Chem. Soc.* **2005**, *127*, 11620–11621.

[14] M. P. Sarnowski, J. R. Del Valle, *Org. Biomol. Chem.* **2020**, *18*, 3690–3696.

[15] A. K. Sanki, R. S. Talan, S. J. Sucheck, *J. Org. Chem.* **2009**, *74*, 1886–1896.

[16] H. Miyabe, K. Yoshida, A. Matsumura, M. Yamauchi, Y. Takemoto, *Synlett* **2003**, *2003*, 0567–0569.

[17] G. Wu, J. R. Nielson, R. T. Peterson, J. M. Winter, *Mar. Drugs* **2017**, *15*, 195.
